# A photochemical strategy for pyrazole to imidazole conversion

**DOI:** 10.1039/d6sc03470e

**Published:** 2026-05-21

**Authors:** Youhao Wei, Kengo Kasama, Antòn Igartua, Dilara Berna Yildiz, Aurore Ceuninck, Thiago dos Santos, Cornelia Büttner, Damien Thevenet, Martin Bossart, Volker Derdau, María Méndez, Philippe Jubault, Thomas Poisson, Baptiste Roure, Daniele Leonori

**Affiliations:** a Institute of Organic Chemistry, RWTH Aachen University Landoltweg 1 52054 Aachen Germany b.roure@rug.nl daniele.leonori@rwth-aachen.de; b Faculty of Pharmaceutical Sciences, University of Toyama Sugitani Toyama 930-0194 Japan; c Department of Chemistry, Faculty of Science, Gazi University Teknikokullar Turkey; d INSA Rouen Normandie, Univ. Rouen Normandie, Univ. Caen Normandie, ENSICAEN, CNRS, Institut CARMeN UMR 6064 F-76000 Rouen France thomas.poisson@insa-rouen.fr; e Minakem Recherche 145 Chemin des Lilas 59310 Beuvry-La-Forêt France; f Integrated Drug Discovery, R&D, Sanofi Germany Industriepark Hoechst 65926 Frankfurt am Main Germany

## Abstract

Heteroaromatic scaffolds are central to modern medicinal chemistry. Methods that can reconfigure the core heterocycle of a molecule while preserving its substitution pattern would greatly streamline analogue synthesis and bioisosteric replacement. Yet, direct heterocycle-to-heterocycle interconversions remain rare. Here we report a photochemical strategy that directly converts pyrazoles into imidazoles with broad functional-group tolerance and full retention of peripheral substitution. The reaction is effective across densely substituted and bicyclic systems and extends to pyrazolo[1,5-*a*]azines, a class of high-value heteroaromatics that have never previously been reconfigured. This photochemical strategy is readily translated to continuous flow, confirming its potential for scalable applications. Our mechanistic studies support an N–N bond homolysis pathway in which solvent-dependent conformational changes govern the reactivity of the ensuing bi-nitrogen-radical intermediates. Overall, this work establishes a practical platform for direct core reconfiguration, providing modular access to imidazole analogues of pyrazoles that are otherwise difficult to prepare or very expensive.

## Abstract 2

We report a photochemical strategy that directly converts pyrazoles into imidazoles with broad functional-group tolerance and full retention of peripheral substitution, providing a modular access to imidazole analogues of pyrazoles.

## Graphical abstract

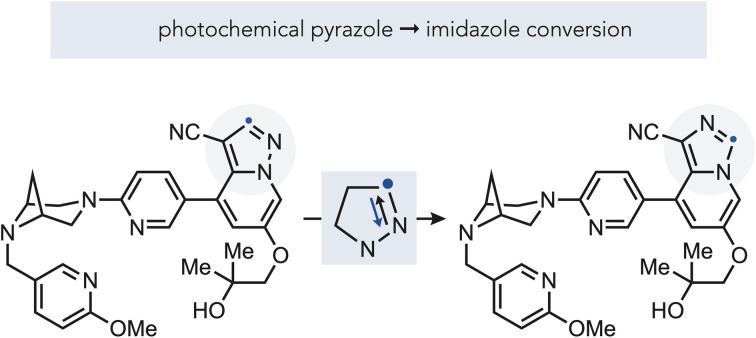

## Introduction

Heteroaromatic scaffolds are ubiquitous in bioactive molecules, where the identity and spatial arrangement of their heteroatoms and ring-substituents play a decisive role in governing binding interactions, physicochemical properties, and metabolic stability.^1^ As a result, medicinal chemists routinely explore closely related heterocyclic cores to fine-tune biological activity and selectivity.^2^ However, modifying a heteroaromatic framework while preserving its substitution pattern remains a major synthetic challenge. In practice, such transformations typically require complete *de novo* synthesis, slowing structure–activity relationship (SAR) campaigns and limiting access to promising regions of chemical space.^3^ Direct heterocycle-to-heterocycle interconversions would provide a powerful solution, provided they can operate with broad functional group tolerance and high fidelity in retaining substitution patterns.^4^

**Scheme 1:**
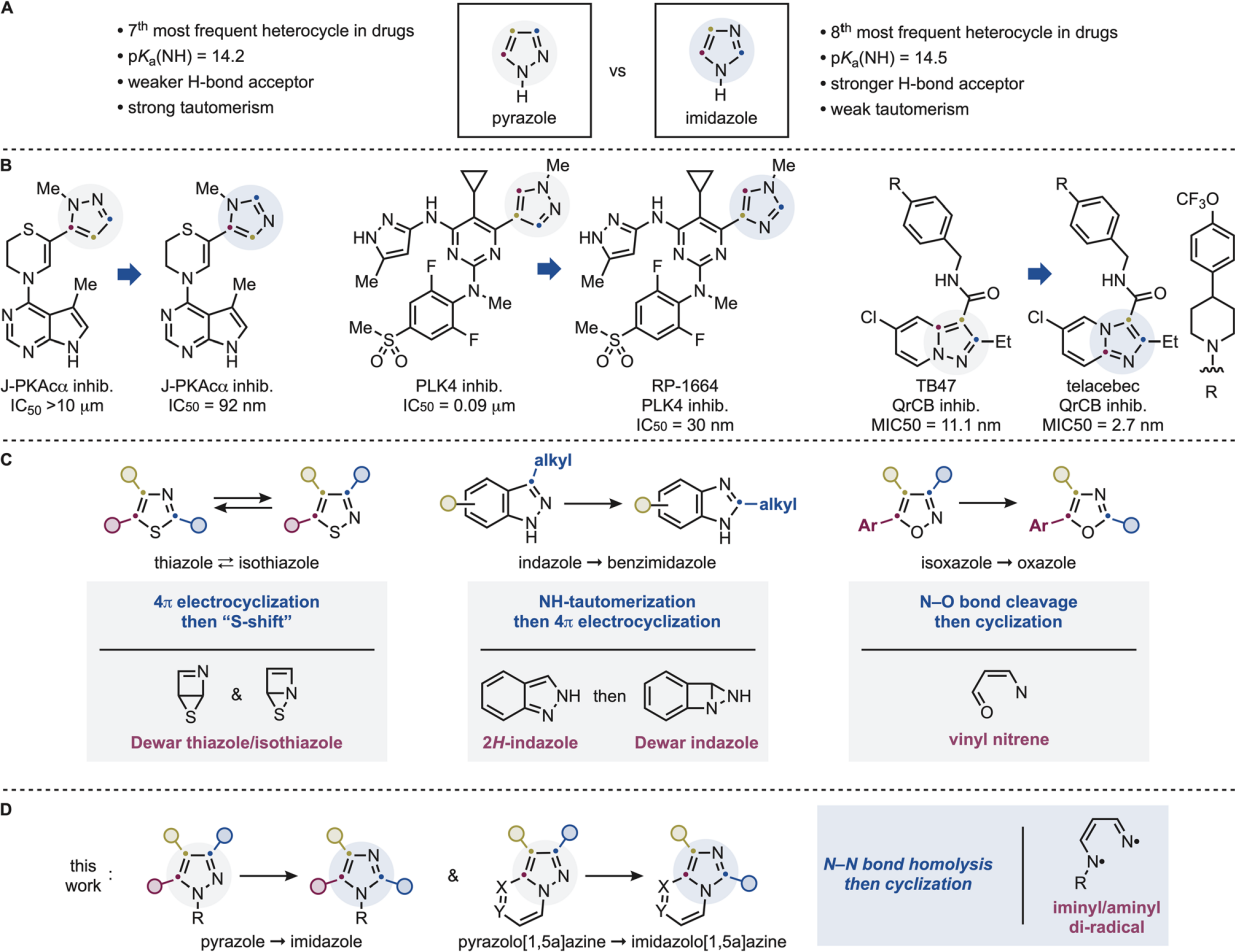
(A) Pyrazole and imidazole are two relevant *N*-heteroaromatics, but their *N*-atom dispositions provide key chemical difference. (B) Examples of pyrazole and imidazole conversions leading to improved bioactive molecules. (C) Previous permutation work on other azoles features mechanistically distinct pathways and intermediates. (D) This works demonstrates a direct pyrazole and imidazole conversion that can be applied to pyrazolo[1,5-*a*]azines.

Nitrogen-containing heteroaromatics are among the most prevalent motifs in drug discovery. In particular, pyrazoles and imidazoles rank among the most frequently encountered five-membered heterocycles in approved pharmaceuticals (Scheme 1A).^5^ Although they share a common ring size and contain two nitrogen atoms, their electronic structures and physicochemical properties differ substantially. Imidazoles possess a pyridine-like nitrogen that confers higher basicity and stronger hydrogen-bond acceptor ability, whereas the adjacent nitrogen arrangement in pyrazoles results in lower basicity and pronounced tautomerism.^6^ These differences translate into distinct binding modes, pharmacokinetic profiles, and biological activities.^7^ Consequently, strategic pyrazole ⇆ imidazole replacement is a common medicinal chemistry tactic to fine-tune biological properties (Scheme 1B). Prominent recent examples include the development of aplithianines, chimeric J-PKAca kinase inhibitors with potential applications in carcinoma treatment,^8^ and RP-1664, the first-in-class orally available PLK4 inhibitor.^9^ Importantly, such scaffold modifications are not limited to isolated pyrazole/imidazole rings but also extend to annulated systems, such as pyrazolo[1,5-*a*]pyridine ⇆ imidazo[1,2-*a*]pyridine transformations,^10^ which, for example, was crucial to the development of the anti-tuberculosis drug telacebec.^11^

Despite their close structural relationship, no general and practical method exists for the direct interconversion of pyrazoles into imidazoles. Early studies by Barltrop, Pavlik, and co-workers established that UV irradiation of simple pyrazoles can induce rearrangement processes.^12^ These seminal investigations provided important mechanistic insight into the photochemical behaviour of azoles, but the reactions typically resulted in complex mixtures, low conversions, and significant decomposition, with product formation often inferred indirectly from mass balance rather than isolation. Consequently, these transformations have not been adopted as synthetic methods, and current approaches to access imidazoles from pyrazole precursors rely instead on stepwise construction from acyclic building blocks.

Our group has recently started to work on establishing the concept of photochemical permutation as a potential strategy to streamline heteroatomatic synthesis and bioactive-molecule diversification.^13^ In this approach, selective excitation of a heteroaromatic framework enables direct reconfiguration into a different heteroaromatic while preserving its substitution pattern. Importantly, these transformations do not operate through a unified mechanism but instead proceed through distinct excited-state manifolds that depend on the heterocycle type. For example, thiazoles & isothiazoles undergo reversible rearrangement *via* 4π-electrocyclization to Dewar intermediates followed by “sulfur-shift”.^13*a*^ In contrast, indazoles require excited-state tautomerization prior to 4π-electrocyclization, imposing strict constraints on substitution patterns as only NH-derivatives with C3 alkyl groups can be engaged.^13*c*,14^ Isoxazoles proceed through N–O bond cleavage leading to vinylnitrene intermediates that display strong dependence on further chromophore-controlled excitation for oxazole formation whereby a C5 aromatic group is necessary in order to observe high reactivity.^15^ These findings highlight that photochemical heterocycle permutation is governed by multiple, mechanistically distinct regimes rather than a single general pathway. Hence, no general framework can be used to generalize reactivity, scope and mechanism but rather a case-by-case study is required to inform on all these aspects.

In this context, we questioned whether pyrazoles would follow any of the previously identified paradigms or instead access a distinct reactivity mode. In this full paper, we report a general and operationally simple photochemical strategy for the direct conversion of pyrazoles into imidazoles (Scheme 1D). The transformation proceeds under mild, additive-free conditions and accommodates a wide range of substitution patterns, including mono-, di-, and trisubstituted pyrazoles as well as diverse annulated systems. Notably, the method enables the first direct reconfiguration of pyrazolo[1,5-*a*]azines, despite the presence of competing chromophores that would be expected to dominate photoexcitation. Mechanistically, this pyrazole → imidazole conversion requires a fine interplay between excited and high-energy intermediates. Our studies revealed the process proceeds *via* N–N bond homolysis to an iminyl/aminyl diradical species followed by conformationally controlled radical cyclization, with solvent polarity and H-bonding playing decisive roles in product selectivity. Such mechanism is intrinsically irreversible and, in contrast to the indazole → benzimidazole case, does not require tautomerization, thus allowing broader tolerance of *N*-substitution patterns and expanded substrate scope. The reaction can be translated to continuous-flow conditions, underscoring its practical potential for scalable applications. Collectively, these findings establish a distinct mechanistic regime for heterocycle permutation and provide a direct entry to imidazole analogues that are otherwise difficult to access.

## Results

### Reaction development

**Scheme 2:**
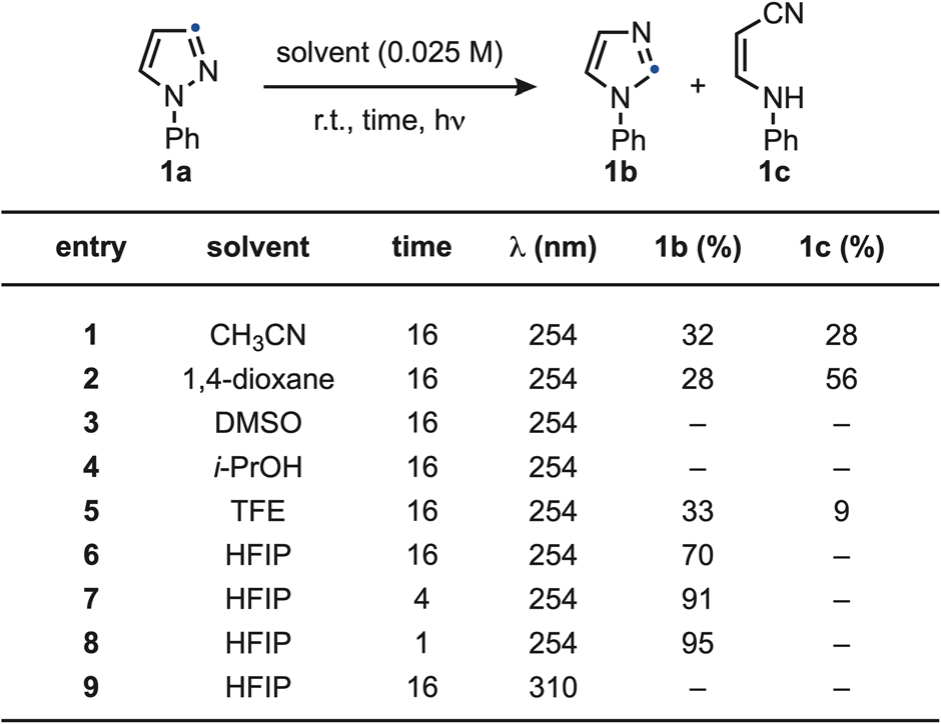
Optimization of the pyrazole-to-imidazole conversion using 1a.

We initiated our studies by examining the photochemical conversion of *N*-Ph-pyrazole 1a into imidazole 1b, which can be visualized as a direct “positional swap” between the *N* atom and the C2 unit (Scheme 2). All reactions were carried out at room temperature under dilute conditions (0.025 M) using *λ* = 254 nm as the irradiation source. Notably, the reaction displayed a pronounced solvent dependence, providing early evidence for the involvement of high-energy intermediates whose fate is strongly influenced by the reaction medium. Specifically, the reaction proceeded in polar solvents such as CH_3_CN (entry 1) and 1,4-dioxane (entry 2), affording 1b in moderate yield but along the ring-opened by-product 1c. When DMSO (entry 3) or *i*-PrOH (entry 4) were employed, complete material decomposition was observed. A notable shift in product distribution was achieved using the more Brønsted acidic solvent TFE (entry 5), which favored the formation of 1b over 1c, albeit in moderate efficiency. This effect was further amplified with HFIP (entry 6), that provided 1b in 70% yield with no detectable formation of 1c. This solvent effect can be potentially rationalized with stabilization of reactive intermediates through H-bonding interactions. The reaction efficiency improved significantly upon reducing the irradiation time (entries 7 and 8), with 1 h being optimum. In contrast, irradiation at *λ* = 310 nm (entry 9) resulted in no product formation, likely due to insufficient absorption at this wavelength. A comprehensive summary of the solvent screening and additional optimization studies is provided in the SI.

### Reaction scope

**Scheme 3:**
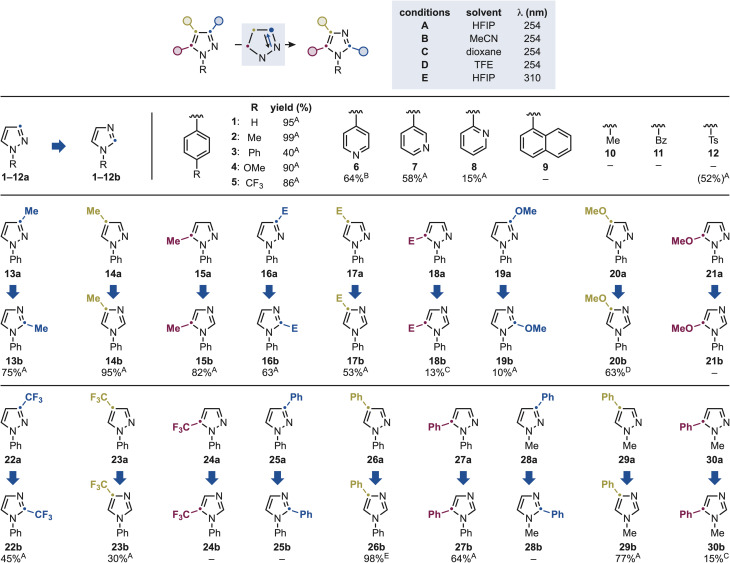
Substrate scope for the pyrazole-to-imidazole conversion using mono-substituted derivatives. E = CO_2_Me. The yield for substrate 12 corresponds to the detosylated product.

With optimized conditions in hand, we examined the influence of substitution on the pyrazole scaffold to delineate the structural features governing reactivity (Scheme 3). For each substrate, we screened five different conditions that varied in solvent composition (A–D) and irradiation wavelength (*E*). UV/vis absorption spectroscopy studies revealed that nearly all substrates absorb in the UV-B region, so we performed all reactions at room temperature under *λ* = 254 and 310 nm irradiation. In general, HFIP (conditions A and *E*) was identified as the optimum medium for various of these examples, albeit specific substrates performed better in other solvents.

*N*-aryl pyrazoles (1a–5a) showed fast and efficient reactivity under these conditions. The conversion of 2a into 2b converts a cheap material (2a: 61 $ per g) in a rather expensive building block (2b: 426 $ per g). Interestingly, the *para*-Ph derivative (3a) gave significantly lower yield, which we attribute to competitive excitation of the extended aromatic system. Given the frequent presence of azine-*N*-substituted azoles in bioactive molecules, we next examined substrates bearing C4, C3, and C2 pyridine substituents (6a–8a). These were successfully engaged in the permutation reaction giving 6b–8b, albeit with moderate to low efficiency.

**Scheme 4:**
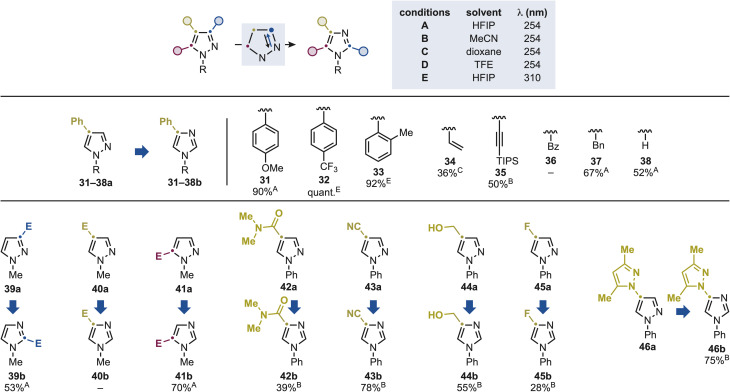
Substrate scope for the pyrazole-to-imidazole conversion using mono-substituted derivatives. E = CO_2_Me.

We also identified several *N*-groups incompatible with the permutation chemistry. In general, we believe reactivity to be governed by the ability of the pyrazole core to compete for photoexcitation with other chromophores present. Indeed, the strongly absorbing *N*-1-naphthyl unit (9a) led to full starting material recovery due to its dominant light absorption, which outcompetes the pyrazole core. *N*-alkyl (*e.g.*, Me, 10a), *N*-Bz (11a), and *N*-Ts (12a) substituents resulted in no reactivity and significant *N*-deprotection in the case of 12a. As we will discuss below, *N*-alkyl pyrazoles can be transformed into *N*-alkyl imidazoles upon simple substitution on the azole nucleus (see Scheme 4). We therefore attribute the lack of reactivity of 10a primarily to its insufficient absorption in the irradiation range employed.

We next investigated the influence of different substituents on the *N-*Ph pyrazole scaffold. Introduction of Me (13a–15a) and ester (16a–18a) groups at the C3, C4, and C5 positions afforded the corresponding imidazoles (13b–17b) in good yields, except for 18a, which gave 18b with low efficiency. Electron-donating OMe (19–21a) and electron-withdrawing CF_3_ (22–24a) groups were also examined. Imidazole formation was successfully achieved for the C3 (19a and 22a) and C4 (20a and 23a) isomers, providing the desired products (19b, 20b, 22b and 23b) in good to moderate yields. In contrast, C5-substituted derivatives (21a and 24a) proved unreactive.

Introduction of a Ph group on the pyrazole core induced a bathochromic shift in the absorption profile, and these reactions were therefore conducted using *λ* = 310 nm irradiation. Under these conditions, a marked positional effect was observed. Specifically, C3-Ph substitution (25a) resulted in full mass recovery with no product formation, whereas C4-Ph (26a) and C5-Ph (27a) derivatives afforded the corresponding imidazoles (26b and 27b) in excellent and moderate yields, respectively. These observations underscore the strong sensitivity of the reaction to structural changes in the heteroaromatic that have strong impact in excited-state dynamics. This reinforces the need for experimental guidelines as *a priori* generalization of reaction scope can be challenging.

Interestingly, the presence of the Ph group on the pyrazole core enabled reaction on some *N*–Me substituted systems. Specifically, while 28a did not lead to productive rearrangement (which is in line with 25a), 29a and 30a gave 29b and 30b in high and low yields respectively.

Hence, using the C4-Ph substitution pattern as a benchmark, we re-evaluated the compatibility of various *N*-substituents in the permutation reaction (Scheme 4). Both *para*-substituted electron-rich (31a) and electron-poor (32a) aryl groups, as well as *ortho*-substituted variants (33a), were well tolerated, delivering products 31–33b in high yields. Notably, *N*-vinyl (34a) and *N*-alkynyl (35a) substrates also participated, affording 34b and 35b in moderate and good yields, respectively. *N*-Bz pyrazole (36a) led to complete mass recovery, but both *N*-Bn and NH derivatives (37a and 38a) were successfully engaged in the reaction, giving the corresponding imidazoles (37b and 38b).

Building on these examples, we next examined the effect the ester substituents on the reactivity of *N*–Me pyrazole core. In this case, a different trend was observed as both the C3 (39a) and C5 (41a) derivatives underwent efficient transformation, providing the 39b and 41b in high yields, while the C4 derivative (40a) was unreactive.

Finally, we assessed the impact of other types of substituents using *N*-Ph substituted pyrazoles. In this case, we successfully demonstrated compatibility with a series of polar functionalities spanning C4-amide (42a), -nitrile (43a), hydroxymethyl (44a) and -fluorine (45a). Interestingly, substrate 46a features two pyrazoles unit and lead exclusively to the rearrangement of the *N*-Ph substituted fragment providing 46b in high yield. We believe this isomerization selectivity to be influenced by the *N*-Ph substituents that likely modulates the photochemical behavior of the attached pyrazole core (see below for comparison the high reactivity of 51a).

Importantly, most pyrazoles included in this study are commercially available and inexpensive, whereas the corresponding imidazoles typically require multi-step synthesis and are often unavailable from vendors (see SI). The transformation therefore represents a direct and practical entry to valuable heterocycles that would otherwise demand significant synthetic and/or economic investment.

**Scheme 5:**
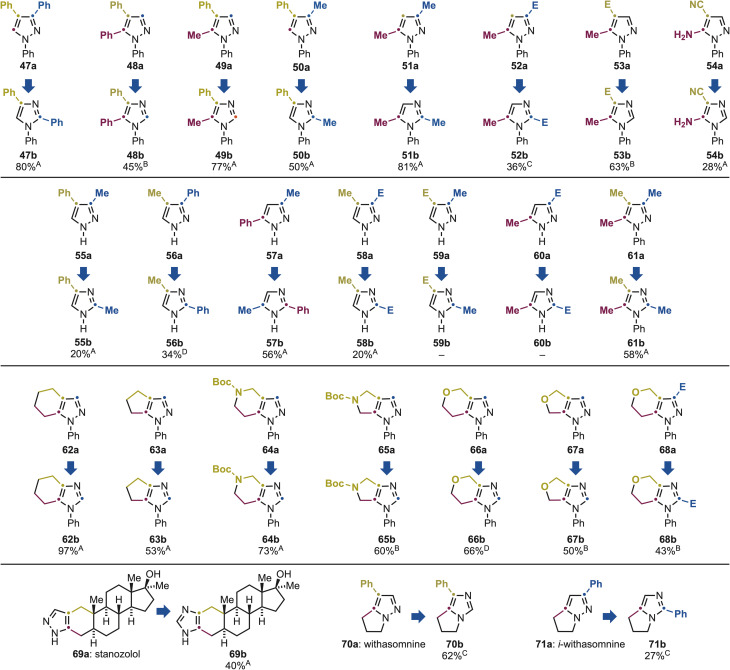
Substrate scope for the pyrazole and imidazole conversion using di- and tri-substituted derivatives. ^A–D^ See Scheme 3 for the legend with the specific conditions used.

We next examined the scope of the transformation on disubstituted pyrazoles to assess its compatibility with increased substitution density (Scheme 5). Using *N*-Ph substitution pattern, we investigated a series of derivatives bearing either two Ph groups at C3,C4 (47a) and C4,C5 (48a), mixed Ph and Me substituents (49a and 50a), two Me groups (51a), or Me and ester functionalities (52a and 53a). In all cases, the desired disubstituted imidazoles (47b–53b) were obtained in good to excellent yields. Notably, the method was also applicable to a substrate push–pull system based on a C4 nitrile and a C5 free amine functionality (54a), affording 54b in moderate yield.

We then turned our attention to NH-pyrazoles to probe the influence of substituents in the absence of *N*-protection or substitution. Here, substrate availability was more restricted because certain isomers cannot be isolated due to rapid tautomerization.^5*a*,7*a*^ Among the accessible derivatives (55a–60a), both Me- and Ph-substituted pyrazoles underwent efficient conversion, delivering the corresponding imidazoles 55b–57b in good yields. In contrast, among the ester-containing substrates, only the C3-ester/C4-methyl pyrazole 58a afforded the desired product 58b, while the remaining analogues 59a and 60a were unreactive. Finally, we demonstrated that the method tolerates further substitution as trisubstituted *N*–Me pyrazole 61a underwent smooth rearrangement to give 61b in good yield.

Having established the generality of the process on mono-, di-, and trisubstituted systems, we next explored its applicability to annulated pyrazoles, which are easily accessible through condensation chemistry.^16^ These substrates are synthetically valuable, as the corresponding imidazoles are typically not commercial and more challenging to prepare. Hence, their direct conversion would offer a strategic shortcut to privileged building blocks suitable for library synthesis and high-throughput screening. Pleasingly, the methodology proved compatible with a variety of bicyclic frameworks annulated at C4 and C5. Successful examples included both carbocyclic systems (62a and 63a) and heterocycles based on *N*-Boc-piperidine (64a), *N*-Boc-pyrrolidine (65a), tetrahydropyran (66a), and tetrahydrofuran (67a) that gave 62b–67b in high yields. The method was also effective for the synthesis of bicyclic trisubstituted imidazoles, as exemplified by the conversion of 68a into 68b in good yield.

Finally, we assessed the applicability of the transformation to complex, bioactive molecules. Stanozolol (69a), a drug used clinically for the treatment of hereditary angioedema, contains a pyrazole core annulated across C3 and C4 onto a steroidal framework. Under our optimized conditions, it was directly and efficiently converted into 69b in 40% yield. This compound had previously required a four-step sequence during the drug development campaign,^17^ an effort now bypassed in a single step by our methodology. Furthermore, withasomnine (70a) and *iso*-withasomnine (71a), two natural alkaloids isolated from the roots of the ashwagandha plant,^18^ feature a bicyclic linkage between C5 and the pyrazole nitrogen. Both substrates were smoothly converted to the corresponding imidazoles (70b and 71b) in good and moderate yields, respectively, thus enabling the fast preparation of natural product-like isomers.

Also in this case, the methodology enabled direct access to building blocks that are either extremely expensive (*e.g.*54a: 10 $ per g while 54b: 2370 $ per g) or currently not commercially available and require individual synthesis (see supplementary material for more information).

The successful conversion of pyrazoles into imidazoles across bicyclic systems prompted us to evaluate whether this reactivity could be extended to more complex heterocyclic frameworks. In particular, we targeted pyrazolo[1,5-*a*]azines, a class of motifs frequently embedded in bioactive molecules and blockbuster drugs.^10*b*^ However, these systems present a different photochemical scenario compared to simple pyrazoles. The presence of an annulated azine introduces a competing chromophore, which typically displays stronger absorption and would be expected to dominate photoexcitation. Moreover, such substrates have not been previously investigated in the context of photochemical permutation, raising the possibility that they may follow entirely different excited-state pathways or fail to undergo productive rearrangement altogether. We therefore questioned whether selective excitation of the pyrazole unit could still be achieved in these systems, or whether the intrinsic photochemical behaviour of the azine would suppress or divert reactivity.

**Scheme 6:**
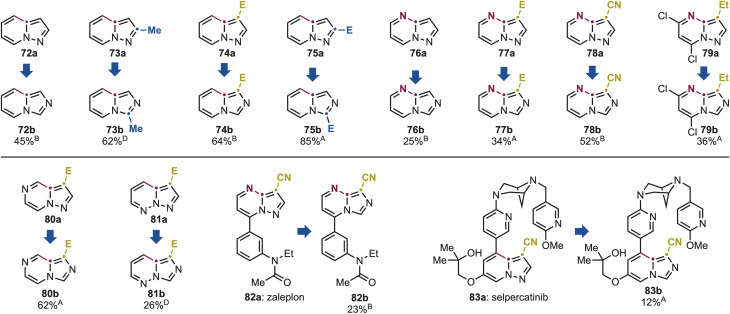
Substrate scope for the pyrazole and imidazole conversion using pyrazolo[1,5*a*]azine derivatives. ^A–D^ See Scheme 3 for the legend with the specific conditions used.

We initiated our investigation with a pyridine derivative 72a, which underwent smooth conversion under standard conditions B to the corresponding 72b in good yield (Scheme 6). Notably, the reaction tolerated various substitution patterns, including a C2-methyl group (73a) and C2- and C3-ester (74a and 75a), affording 72b–75b in high to excellent yields.

The rearrangement was next applied to pyrimidine derivatives 76a and 77a, bearing C2- and C3-ester as well as nitrile substituents (78a), which gave 76b–78b in moderate yields. Pyrazolo[1,5-*a*]pyrimidines are common scaffolds in bioactive molecules. Notably, 79a is used in the manufacture of the anticancer agents dinaciclib *via* S_N_Ar chemistry at the activated aryl chloride functionalities.^19^ Pleasingly, these groups were fully tolerated under our conditions, delivering 79b in useful yield, which can be integrated into existing synthetic routes for the preparation of drug analogues. We further examined the scope of the reaction with pyrazine and pyridazine derivatives 80a and 81a, which afforded 80b and 81b in high and moderate yields, respectively. It is important to note that also cross these azine classes, permutation provided direct access to imidazo[1,5-*a*]azine scaffolds that are either prohibitively expensive or entirely unavailable commercially (*e.g.*, 76a: 78 $ per g, while 76b: 1962 $ per g). This underscores the practical value of the method that converts widely available building blocks into elusive analogues in a single step.

Given the prevalence of pyrazolo[1,5-*a*]azine motifs in bioactive molecules, we next evaluated the potential of this method for late-stage ring reconfiguration of complex substrates. Zaleplon (82a), a hypnotic agent used for the treatment of severe insomnia, features a C3-nitrile-substituted pyrazolo[1,5-*a*]pyrimidine core with a pendant amide-substituted benzene ring. Under our photochemical conditions, we successfully obtained the corresponding imidazole derivative 82b in 23% yield. Furthermore, the structurally complex anticancer drug selpercatinib 83a underwent selective isomerization of its pyrazolo[1,5-*a*]pyridine core, delivering the rearranged product 83b in 12% yield. Although the conversions in these two cases are currently moderate, they demonstrate the ability of this methodology to reconfigure core heterocyclic frameworks in highly functionalized molecules in a single step, thus bypassing the need for lengthy and resource-intensive total syntheses.

### Reaction scalability

**Scheme 7:**
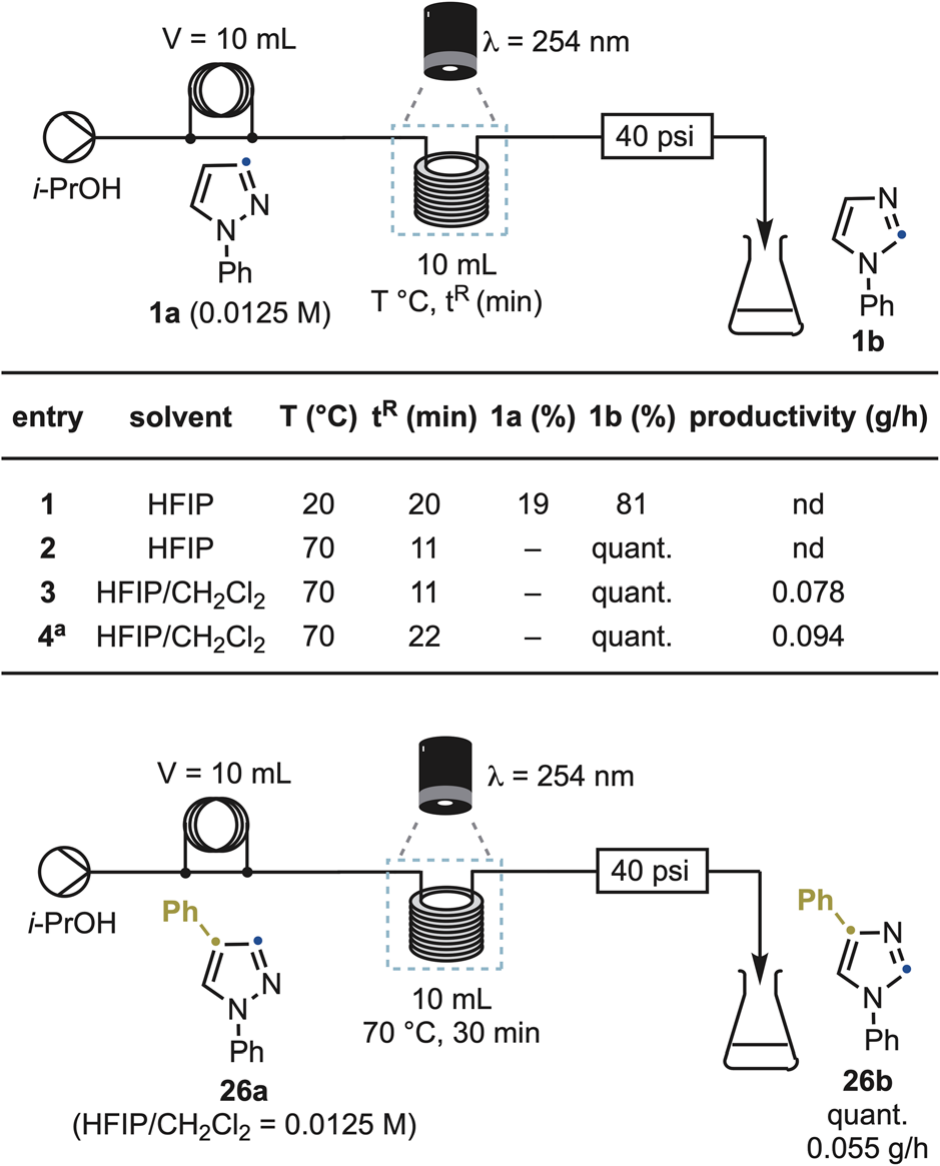
Reaction scale-up using photo-flow equipment. ^*a*^ Reaction conducted at 0.025 M instead of 0.0125 M. nd: not determined.

To demonstrate that the rearrangement is not limited to small-scale batch photolysis, we translated the reaction into a continuous-flow photochemical process (Scheme 7). Using 1a as the benchmark substrate, initial settings closely mirrored the batch setup (HFIP, *λ* = 254 nm, 0.0125 M). Under these conditions, a 10 mL UV reactor afforded 1b in 81% yield with 19% recovered 1a at 20 °C and a 20 min residence time (entry 1). Optimization revealed that reaction temperature was the key determinant of throughput. Increasing the reactor temperature to 70 °C while reducing the residence time to 11 min delivered complete conversion (entry 2). This temperature effect was fully consistent with the reaction's photochemical nature and not attributable to thermal activation. To reduce solvent cost while maintaining efficiency, we replaced HFIP with a 1 : 1 HFIP/CH_2_Cl_2_ mixture, which delivered the same conversion and identical quantum yields (*Φ* = 0.064 *vs.* 0.063),^20^ and afforded 1b in 79% yield and a productivity of 0.078 g h^−1^ (entry 3). Reactivity was maintained upon doubling the substrate concentration (96% yield, 0.094 g h^−1^ productivity), albeit at a slightly longer residence time (22 min), demonstrating that the transformation is not inherently limited by light penetration under these conditions (entry 4).

A second representative substrate, 26a, also reached full conversion to the imidazole product 26b under similar conditions, with a 30 min residence time (productivity 0.055 g h^−1^). The robustness of the method across substrates confirms that the rearrangement is flow-compatible, scalable, and operationally simple, suitable for both library synthesis and gram-scale analogue production.

### Mechanistic proposal

**Scheme 8:**
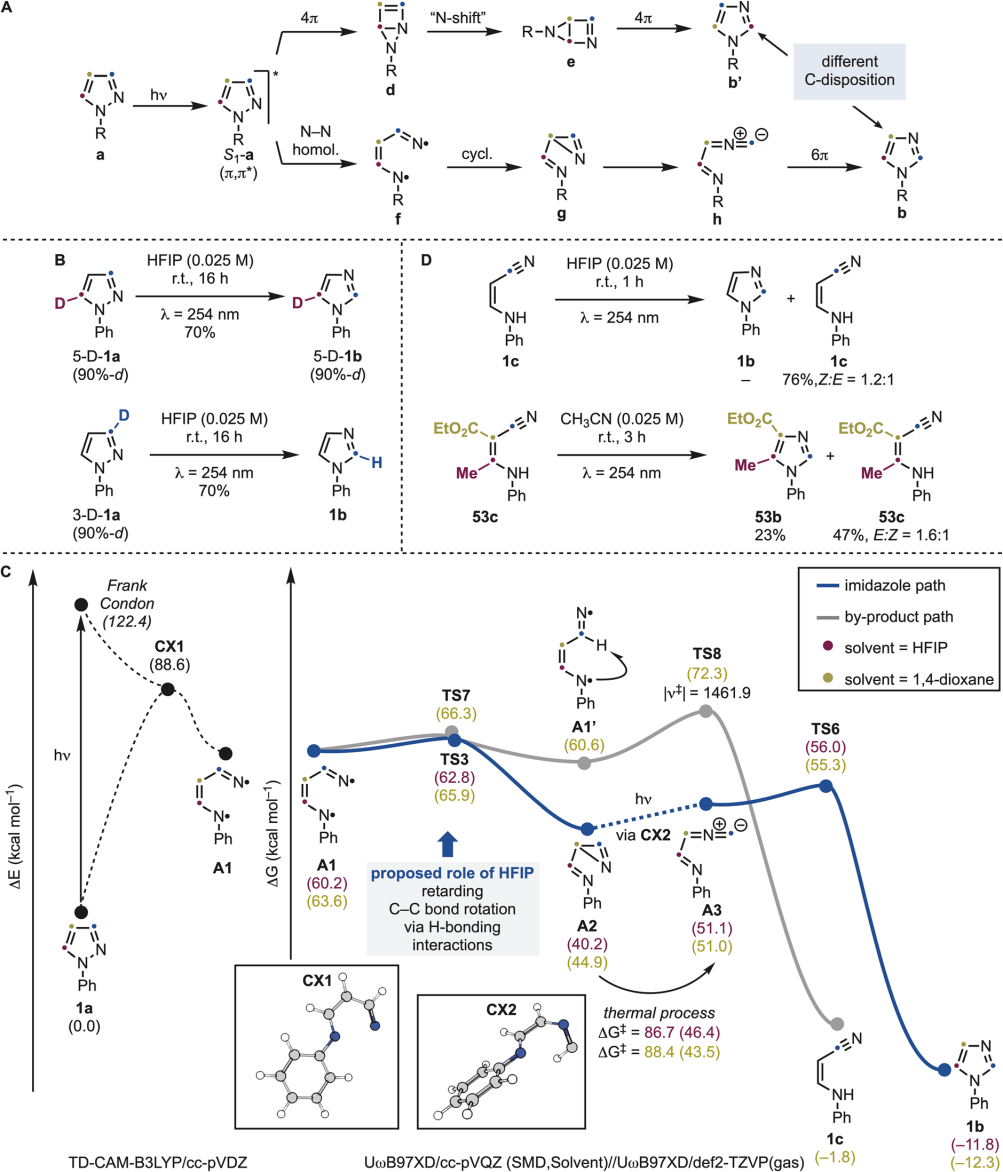
(A) Proposed mechanistic pathway for the pyrazole and imidazole conversion and computational studies. (B) Experiments on deuterated 1a. (C) Computational profile for the conversion of 1a into 1b and 1c. All energy values are in kcal mol^−1^. (D) Photochemical experiments on the reactivity of enamine nitriles by-products.

In principle, two mechanistic pathways, both originating from the *S*_1_-excited pyrazole (S_1_-a), can account for the observed transformation (Scheme 8a). By analogy with our previous work on thiazole/isothiazole and indazole/benzimidazole interconversions,^13*a*,*c*^ an excited-state 4π electrocyclization could form a Dewar intermediate d, which after an *N*-shift would furnish intermediate *e* and ultimately the imidazole b′. Alternatively, direct N–N bond homolysis^21^ could generate a di-*N*-radical f. This species would afford, upon cyclization, the imine–azirine intermediate g and, subsequently, the methanideiminium species h, capable of delivering b by 6π electrocylic ring closure.

Although both pathways converge to the same imidazole framework, they involve fundamentally different bond rearrangements with clear structural consequences. The radical pathway leading to b can be viewed as an “N–C3 swap,” preserving the original C4–C5 connectivity. In contrast, formation of a Dewar intermediate d would entail complete positional reorganization of the carbon framework in b′. Our experimental results show no evidence of positional scrambling, providing compelling support that the reaction proceeds through N–N bond cleavage rather than Dewar-type electrocyclization. To further support the proposed homolytic mechanism experimentally, C5- and C3-deuterated 1a were prepared and subjected to the photochemical conditions (Scheme 8b). In both cases, 1b was obtained in 70% yield. However, complete deuterium retention was observed for C5-labelled 1a, whereas C3-labelled 1a furnished non-deuterated 1b. This latter outcome is consistent with the intermediacy of methaniminium species h, from which C3-deuterium labeling is lost.

To obtain further evidence on the reaction mechanism we conducted computational studies using *N*-Ph pyrazole 1a as the model (Scheme 8b). Upon photoexcitation this species populates the S_1_ state (*E* = 122.4 kcal mol^−1^), which has π,π* character. From this excited state, a conical intersection (CX1) was identified at *E* = 88.6 kcal mol^−1^. This structure features a complete breakage of the N–N bond and therefore funnels the system into N–N bond homolysis pathway, generating the diradical intermediate A1. In HFIP solvent, this intermediate can cyclize to A2 (Δ*G*° = −20.0 kcal mol^−1^) *via* a shallow transition state (Δ*G*^‡^ = 2.8 kcal mol^−1^). We initially hypothesized that A2 might thermally interconvert into A3. However, our calculations revealed that this pathway is associated with a prohibitively high kinetic barrier (Δ*G*^‡^ = 46.4 kcal mol^−1^), rendering it inaccessible under the experimental conditions. Instead, time-dependent DFT studies demonstrated that A2 absorbs light in the same wavelength range as our irradiation source. Hence, we propose that a second photochemical event, involving A2 could populate the excited state S_1_-A2 (*E* = 104.9 kcal mol^−1^), which subsequently evolves through a conical intersection (CX2, *E* = 68.6 kcal mol^−1^, see SI) to form A3. From this intermediate, a 6π electrocyclization would deliver 1b in a kinetically facile and exothermic manner (Δ*G*^‡^ = 4.9 kcal mol^−1^; Δ*G*° = −62.9 kcal mol^−1^). Thus, in contrast to our previous work on thiazoles and indazoles, which proceed *via* pericyclic excited-state pathways, or isoxazoles, which proceed through the formation of a nitrene intermediate, the present transformation operates through N–N bond homolysis *via* di-*N*-radical species, establishing a distinct mechanistic regime for this heterocycle.

We then became interested in understanding the crucial role of HFIP in selectively providing access to the desired imidazole product. Indeed, as discussed above, when switching from H-bond-donating HFIP to 1,4-dioxane, the nitrile by-product 1c was formed in a 1 : 1 ratio with 1b (see Scheme 2). We propose this solvent dependence can be rationalized by conformational effects on A1. In HFIP, A1 should be stabilized by H-bonding interactions in an s-*cis* conformation that can readily cyclize to A2. In contrast, in 1,4-dioxane, a C–C bond rotation leads to a nearly isoenergetic s-*trans* conformer A1′. Crucially, this isomerization has the same kinetic barrier of the cyclization to A2, so we propose can compete with it. Species A1′ is unable to cyclize and instead can undergo 1,4-HAT between the iminyl-type radical and the acyl-type C(sp^2^)–H bond to give by-product 1c. Although this HAT step exhibits a high calculated barrier, it is characterized by a large imaginary frequency, indicative of significant quantum tunnelling,^22^ which we propose can enable the process to compete under the reaction conditions. Overall, it is noteworthy that the striking solvent effect exerted by HFIP in both the imidazole → benzimidazole and pyrazole → imidazole permutations arises from fundamentally different mechanistic origins. In the case of NH-indazoles, the acidity of HFIP is required to promote excited-state tautomerization,^13*c*^ whereas for pyrazoles it plays a key role in locking the conformation of the resulting di–N radical species A1.

The formation of by-product 1c warrants further discussion. According to our mechanistic proposal, this species arises from a parasitic pathway that diminishes the efficiency of the desired pyrazole → imidazole conversion when CH_3_CN solvent is used. Consistent with this, irradiation of an authentic sample of 1c did not yield 1b, but instead led to partial *Z* → *E* olefin isomerization (Scheme 8d). However, the substitution pattern of this species can significantly influence its photochemical behaviour. This was demonstrated by the preparation of 53c*via* irradiation of 53a in CH_3_CN, which afforded 53b (22%) and 53c (44%). Further irradiation of 53c produced 53b in 23% yield, accompanied by olefin isomerization. Therefore, we cannot exclude that, in certain cases, the pathway leading to the enamine–nitrile by-products may also positively contribute to the overall productivity of the process. These observations reinforce the notion that photochemical permutation mechanisms are highly sensitive to heteroaromatic substitution patterns. Such behavior is difficult to rationalize based on current structure–reactivity paradigms and highlights a gap in our understanding of the excited-state behavior of these heteroaromatic systems.

## Conclusions

We have developed a general photochemical strategy that transforms pyrazoles into imidazoles in a single step and fully preserve the substitution pattern of the parent heterocycle. The method tolerates a wide spectrum of electronic and steric environments, operates reliably across densely substituted and annulated systems, and, critically, enables the first direct reconfiguration of pyrazolo[1,5-*a*]azines. The successful translation of the reaction to continuous flow underscores its practical potential for scalable analogue generation. Our mechanistic studies suggest the heterocycle rearrangement is initiated by N–N bond homolysis and controlled by H-bonding interactions with the solvent. Taken together, these findings establish a blueprint for direct core reconfiguration as a versatile tool for accessing otherwise inaccessible heterocycles and accelerating bioisosteric exploration in medicinal chemistry.

## Author contributions

B. R. and D. L. designed the project. Y. W., K. K., A. I., T. d. S. and C. B. performed all synthetic experiments. D. B. Y. performed all computational studies. T. P. designed the flow experiments and quantum yield determinations. A. C. performed all flow experiments. All authors analysed the results and wrote the manuscript.

## Conflicts of interest

M. B., V. D. and M. M. are employees of Sanofi and might have shares and/or stock options in the company. The other authors do not declare competing interests.

## Supplementary Material

SC-017-D6SC03470E-s001

SC-017-D6SC03470E-s002

## Data Availability

All data is available in the supporting information (SI). Supplementary information is available. See DOI: https://doi.org/10.1039/d6sc03470e.
