# Supplementary material for: A photochemical strategy for pyrazole to imidazole conversion
Source: Chem Sci. 2026 May 21;17(26):12860–70. doi: 10.1039/d6sc03470e (PMC13213477; doi:10.1039/d6sc03470e)
Supplement: SC-017-D6SC03470E-s002 [file SC-017-D6SC03470E-s002.pdf]

Supplementary Information (SI) for Chemical Science.
This journal is © The Royal Society of Chemistry 2026

A photochemical strategy for pyrazole to imidazole conversion

Youhao Wei,[1#] Kengo Kasama,[1,2#] Antòn Igartua,[1] Dilara Berna Yildiz,[1,3] Aurore Ceuninck,[4] Thiago dos Santos,[1] Cornelia Büttner,[1] Damien Thevenet,[5] Martin Bossart,[6] Volker Derdau,[6] María Méndez,[6] Philippe Jubault,[4] Thomas Poisson,[4*] Baptiste Roure,[1*] Daniele Leonori[1*]

[1]*Institute of Organic Chemistry, RWTH Aachen University, Landoltweg 1, 52054 Aachen, Germany.*

[2] *Faculty of Pharmaceutical Sciences, University of Toyama, Sugitani, Toyama 930-0194, Japan.*

[3] *Department of Chemistry, Faculty of Science, Gazi University, Teknikokullar, Türkiye*

[4] *INSA Rouen Normandie, Univ. Rouen Normandie, Univ. Caen Normandie, ENSICAEN, CNRS, Institut CARMeN UMR 6064, F-76000 Rouen, France*

[5] *Minakem Recherche, 145 Chemin des Lilas, 59310 Beuvry-La-Forêt, France*

[6] *Integrated Drug Discovery, R&D, Sanofi Germany, Industriepark Hoechst, 65926 Frankfurt am Main, Germany*

thomas.poisson@insa-rouen.fr; b.roure@rug.nl; daniele.leonori@rwth-aachen.de

[#] These authors contributed equally to this work

1 General Experimental Details **3**

2 General Procedures **4**

3 Starting Material Synthesis **8**

4 Reaction Optimization **30**

4.1 Permutations of 1-Phenyl-1H-pyrazole (**1a**) 30

4.2 Permutations of 1,4-Diphenyl-1*H*-pyrazole (**26a**) 33

5 Pictures of Reaction Set-up **35**

6 UV-Vis Spectra **36**

7 Substrate Scope **38**

8 Flow Chemistry Experiments **64**

8.1 Optimisation of the Reaction Conditions for **1a** 64

8.2 Optimisation of the Reaction Conditions for **26a** 65

8.3 Scale-up Reaction 66

9 Mechanistic Studies **67**

9.1 Light On/Off Experiments 67

9.2 Study on the azirine intermediate 67

9.3 Kinetic Studies 69

9.4 Quantum yield measurement 72

9.4.1 Determination of the Photon Flux – (*E*)-Azobenzene 72

9.4.2 Quantum Yield Determinations 75

10 Computational Studies 76
10.1 Computational Details 76
10.2 Excited State Calculations of Pyrazoles 77
10.3 Electronic Structure of Biradical Intermediates (A1 and A1’) 78
10.4 Rotational Interconversion of A1 and A1’ 80
10.5 Reaction Profile 81
10.6 Tunneling Calculations 83
11 Comparison Between Permutation Reactivity and Current Synthetic Approaches for Imidazole Synthesis 87
12 NMR Spectra 89
13 References 149

1 General Experimental Details

All required fine chemicals were used directly without purification unless stated otherwise. All air and moisture sensitive reactions were carried out under an argon atmosphere using standard Schlenk manifold technique. All solvents were bought from Acros as 99.8% purity and degassed by Ar bubbling. ^{1}H and ^{13}C Nuclear Magnetic Resonance (NMR) spectra were acquired at various field strengths as indicated and were referenced to $CDCl_3$ (7.26 and 77.16 ppm for ^{1}H and ^{13}C respectively). ^{1}H NMR coupling constants are reported in Hertz and refer to apparent multiplicities and not true coupling constants. Data are reported as follows: chemical shift, integration, multiplicity (s = singlet, br s = broad singlet, d = doublet, t = triplet, q = quartet, p = quintet, sx = sextet, hp = heptet, m = multiplet, dd = doublet of doublets, etc.), proton assignment (determined by 2D NMR experiments: NOESY, HSQC and HMBC) where possible. ^{19}F NMR spectra were recorded and reported unreferenced. High-resolution mass spectra were obtained using a JEOL JMS-700 spectrometer or a Fissions VG Trio 2000 quadrupole mass spectrometer. Spectra were obtained using electron impact ionization (EI) and chemical ionization (CI) techniques, or positive electrospray (ES). Analytical TLC: aluminum backed plates pre-coated (0.25 mm) with Merck Silica Gel 60 F254. Compounds were visualized by exposure to UV-light or by dipping the plates in permanganate ($KMnO_4$) stain followed by heating. Flash column chromatography was performed using Merck Silica Gel 60 (40–63 μm). Absorption and emission spectra were obtained using an Horiba Duetta spectrometer and 1 mm High Precision Cell made of quartz from Hellma Analytics. All mixed solvent eluents are reported as v/v solutions. Reactions irradiated at 254 nm, 300 nm and 310 nm were carried out in a Photochemical Multirays Reactor from Helios Quartz equipped with the corresponding lamps. All the reactions were conducted in CEM 10 mL glass microwave tubes and EN09 quartz glass tubes.

2 General Procedures

General Procedure for the Arylation of Pyrazoles via Ullmann Coupling − GP1

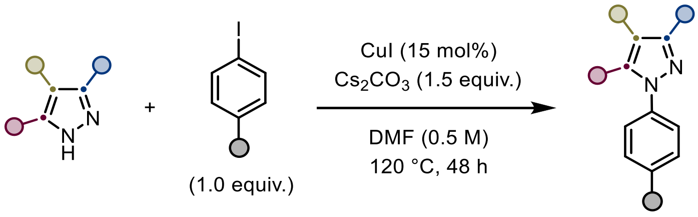

A round-bottom flask equipped with a stirring bar was charged with the pyrazole (1.5 mmol, 1.0 equiv.), the iodobenzene (1.5 mmol, 1.0 equiv.), CuI (43 mg, 0.225 mmol, 15 mol%) and Cs_2CO_3 (733 mg, 2.25 mmol, 1.5 equiv.). The flask was evacuated and refilled with Ar (x 3). DMF (0.5 M) was added. The reaction was stirred at r.t. for 30 min and then at 120 °C for 48 h. After completion of the reaction as monitored by TLC, the mixture was cooled to r.t. and diluted with EtOAc (5 mL) and NH_4Cl(sat.) (5 mL). The layers were separated, and the aqueous layer was extracted with EtOAc (5 mL x 3). The combined organic layers were washed with brine (15 mL), dried (Na_2SO_4) and filtered. The solvent was evaporated, and the residue was purified by column chromatography on silica gel to give the desired product.

General Procedure for the Synthesis of Pyrazoles via Knorr Pyrazole Synthesis – GP2

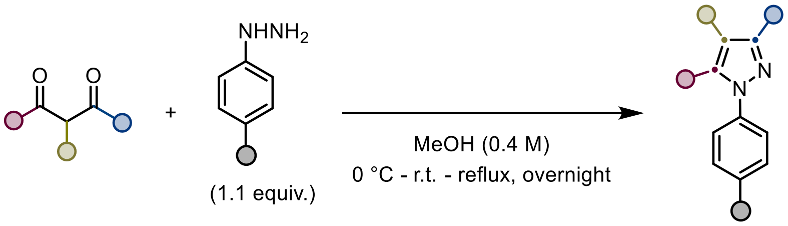

A round-bottom flask equipped with a stirring bar was evacuated and refilled with Ar (x 3). The 1,3-diketone (2 mmol, 1.0 equiv.), phenyl hydrazine (2.2 mmol, 1.1 equiv.) and dry MeOH (0.4 M) were added at 0 °C. The reaction was stirred at r.t. for 1 h and then under reflux overnight. After completion of the reaction as monitored by TLC, the solvent was evaporated, and the residue was directly purified by column chromatography on silica gel to give the desired product.

General Procedure for the Synthesis of Pyrazoles via Condensation Reaction – GP3

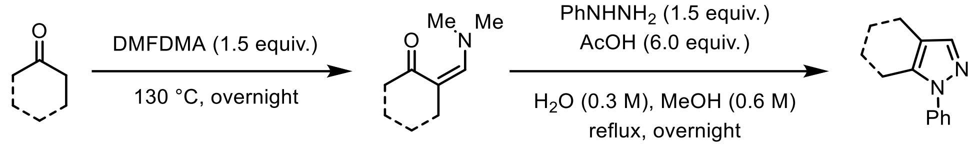

Step 1: A round-bottom flask equipped with a stirring bar was charged with the ketone (5.0 mmol, 1.0 equiv.) and *N*,*N*-dimethylformamide dimethyl acetal (DMFDMA) (1.0 mL, 7.5 mmol, 1.5 equiv.). The flask was evacuated and refilled with Ar (x 3). The reaction was stirred at 130 °C overnight. After completion of the reaction as monitored by TLC, the mixture was cooled to r.t. The solvent was evaporated, and the crude product was used in the next reaction without further purification.

Step 2: A round-bottom flask equipped with a stirring bar was charged with crude from the previous step (5 mmol, 1.0 equiv.). The flask was evacuated and refilled with Ar (x 3). MeOH (13.0 mL), AcOH (3.0 mL), H_2O (26.0 mL) and $PhNHNH_2$ (0.74 mL, 7.5 mmol, 1.5 equiv.) were added. The reaction was stirred under reflux overnight. After completion of the reaction as monitored by TLC, the mixture was cooled to r.t. and evaporated to approx. half volume. Then mixture was extracted with CH_2Cl_2 (10 mL x 3). The combined organic layers were washed with brine (15 mL x 1), dried (Na_2SO_4) and filtered. The solvent was evaporated, and the residue was purified by column chromatography on silica gel to give the desired product.

General Procedure for the Synthesis of Pyrazoles via Fischer Esterification – GP4a

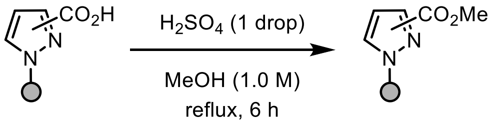

A round-bottom flask equipped with a stirring bar was charged with the pyrazole-carboxylic acid (1.5 mmol, 1.0 equiv.). The flask was evacuated and refilled with Ar (x 3). MeOH (1.5 mL) and concentrated H_2SO_4 (1 drop) were added. The reaction was stirred under reflux for 6 h. After completion of the reaction as monitored by TLC, the mixture was cooled to r.t. and $NaHCO_3$(sat.) (5 mL) was added. The layers were separated, and the aqueous layer was extracted with EtOAc (10 mL x 3). The combined organic layers were washed with brine (15 mL x 1), dried with Na_2SO_4 and filtered. The solvent was evaporated, and the residue was purified by column chromatography on silica gel to give the desired product.

General Procedure for the Esterification of Carboxylic Acid Pyrazole – GP4b

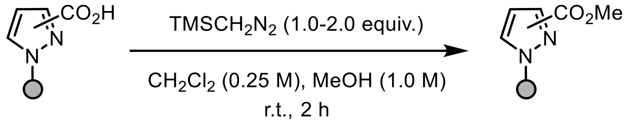

A round-bottom flask equipped with a stirring bar was charged with corresponding pyrazole-carboxylic acid (1.0 mmol, 1.0 equiv.). The flask was evacuated and refilled with Ar (x 3). CH_2Cl_2 (4.0 mL), MeOH (1.0 mL) and $TMSCH_2N_2$ (2 M in hexane, 1.0-2.0 mmol, 1.0-2.0 equiv.) were added. The reaction was stirred at r.t. for 2 h. After completion of the reaction as monitored by TLC, $NaHCO_3$(sat.) (5 mL) was added. The layers were separated, and the aqueous layer was extracted with CH_2Cl_2 (10 mL x 3). The combined organic layers were washed with brine (15 mL x 1), dried Na_2SO_4) and filtered. The solvent was evaporated, and the residue was purified by column chromatography on silica gel to give the desired product.

General Procedure for the Synthesis of Bicyclic Pyrazoles – GP5

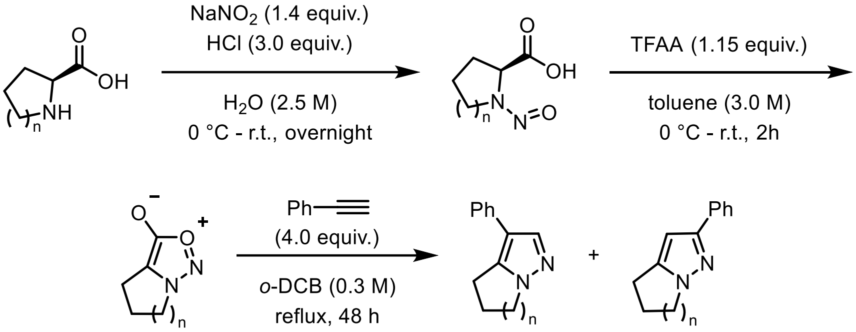

Step 1: A round-bottom flask equipped with a stirring bar was charged with $NaNO_2$ (966 mg, 14 mmol, 1.4 equiv.) and the amino acid (10 mmol, 1.0 equiv.). The flask was evacuated and refilled with Ar (x 3). The flask was cooled to 0 ºC. H_2O (4.0 mL) and HCl (2.6 mL, 35%, 30 mmol, 3.0 equiv.) were added. The reaction was then warmed to r.t. and stirred for 16 h. The reaction mixture was diluted with MTBE (10 mL) and the aqueous layer was extracted with MTBE (10 mL x 2). The combined organic layers were washed with brine (15 mL x 1), dried (Na_2SO_4) and filtered. The solvent was evaporated below 35 °C, and the crude product was used in the next reaction without further purification.

Step 2: A flask equipped with a stirring bar was charged with the crude intermediate (7.6 mmol, 1.0 equiv.). toluene (2.5 mL) and TFAA (1.6 mL, 11.5 mmol, 1.5 equiv.) were added at 0 °C. The reaction was then warmed to r.t. and stirred for 2 h. The reaction mixture was added into a stirred mixture of

K_2CO_3 (1.7 g, 12 mmol, 1.6 equiv.), CH_2Cl_2 (4.0 mL), and H_2O (2.0 mL) at 0 °C. The layers were separated, and the aqueous layer was extracted with CH_2Cl_2 (10 mL x 3). The combined organic layers were washed with brine (15 mL x 1), dried (Na_2SO_4) and filtered. The solvent was evaporated below 35 °C, and the crude product can be used in the next reaction without further purification.

Step 3: A flask equipped with a stirring bar was charged with sydnone (3 mmol, 1.0 equiv.). The flask was evacuated and refilled with Ar (x 3). Phenylacetylene (1.32 mL, 12 mmol, 4.0 equiv.) and *o*-dichlorobenzene (10 mL) were added. The reaction was then stirred under reflux for 48 h. After completion of the reaction as monitored by TLC, the mixture was cooled to r.t., the solvent was evaporated, and the residue was purified by column chromatography on silica gel to give the desired product.

General Procedure for the Permutation Reaction – GP*

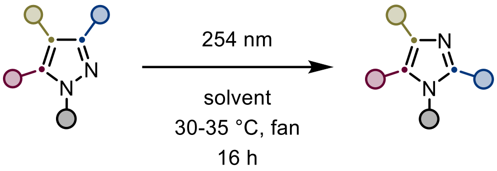

A dry quartz tube equipped with a stirring bar was charged with the pyrazole (0.1 mmol). The tube was capped with a rubber cap, evacuated and refilled with Ar (x 3). The corresponding degassed solvent was added. The tube was placed into a Helios Quartz photoreactor equipped with 254 nm lamps. The photoreactor and a fan were switched on and the mixture was stirred under irradiation for 16 h. The solvent was evaporated, and the residue was purified by column chromatography on silica gel to give the desired product.

Table S1. Main solvent variations used during the permutation reactions

GP	Solvent
**GP*1**	HFIP (0.025 M)
**GP*2**	CH_3CN (0.025 M)
**GP*3**	1,4-dioxane (0.025 M)
**GP*4**	TFE (0.025 M)

3 Starting Material Synthesis

Starting material **1a**, **2a**, **4a**, **8a**, **13a**, **17a**, **30a**, **39a**, **41a**, **44a**, **45a**, **51a**, **54a**, **55a**, **57a**, **69a**, **72a**, **76-80a**, **83a** are commercially available.

1-([1,1'-Biphenyl]-4-yl)-1*H*-pyrazole (3a)

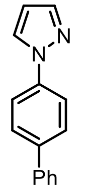

Following **GP1**, 1-*H*-pyrazole (102 mg, 1.5 mmol, 1.0 equiv.) and 4-iodobiphenyl (420 mg, 1.5 mmol, 1.0 equiv.) gave **3a** (271 mg, 82%) as a white solid. ^{1}H NMR (600 MHz, $CDCl_3$) δ 7.97 (1H, d, J = 2.3 Hz), 7.81-7.74 (3H, m), 7.71-7.66 (2H, m), 7.65-7.60 (2H, m), 7.47 (2H, t, J = 7.5 Hz), 7.37 (1H, t, J = 7.4 Hz), 6.50 (1H, t, J = 2.1 Hz); ^{13}C NMR (151 MHz, $CDCl_3$) δ 141.3, 140.2, 139.5, 139.5, 129.0, 128.2, 127.6, 127.1, 126.8, 119.6, 107.8. Data in accordance with the literature.[1]

1-(4-(Trifluoromethyl)phenyl)-1*H*-pyrazole (5a)

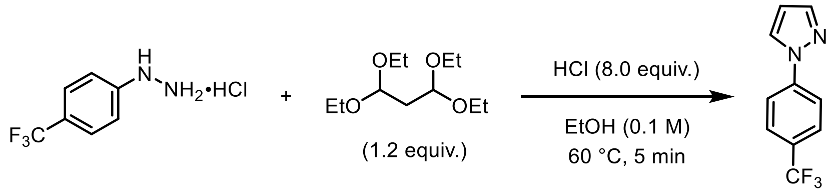

A flask equipped with a stirring bar was charged with (4-(trifluoromethyl)phenyl)hydrazine hydrochloride (638 mg, 3.0 mmol, 1.0 equiv.). The flask was evacuated and refilled with Ar (x 3). 1,1,3,3-tetraethoxypropan (0.9 mL, 3.6 mmol, 1.2 equiv.), HCl (35%, 2.1 mL, 24.0 mmol, 8.0 equiv.) and EtOH (30 mL) were added. The reaction was then stirred at 60 °C for 5 min. After completion of the reaction as monitored by TLC, the mixture was diluted with CH_2Cl_2 (15 mL) and H_2O (20 mL). The layers were separated, and the aqueous layer was extracted with CH_2Cl_2 (15 mL x 3). The combined organic layers were washed with brine (15 mL x 1), dried (Na_2SO_4) and filtered. The solvent was evaporated, and the residue was purified by column chromatography on silica gel to give **5a** (530 mg, 83%) as a white solid. ^{1}H NMR (600 MHz, $CDCl_3$) δ 7.98 (1H, d, J = 2.5 Hz), 7.83 (2H, d, J = 8.4 Hz), 7.76 (1H, d, J = 1.7 Hz), 7.71 (2H, d, J = 8.5 Hz), 6.51 (1H, dd, J = 2.5, 1.7 Hz); ^{13}C NMR (151 MHz,

CDCl3) δ 142.7, 142.1, 128.4 (q, J = 32.9 Hz), 126.9, 126.9 (q, J = 3.8 Hz), 124.1 (q, J = 271.8 Hz), 118.9, 108.6; ^{19}F NMR (565 MHz, $CDCl_3$) δ –62.3. Data in accordance with the literature.[2]

4-(1*H*-Pyrazol-1-yl)pyridine (6a)

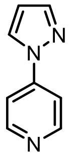

Following **GP1**, 1-*H*-pyrazole (204 mg, 3.0 mmol, 2.0 equiv.) and 4-iodopyridine (495 mg, 1.5 mmol, 1.0 equiv.) gave **6a** (163 mg, 75%) as a white solid. ^{1}H NMR (600 MHz, $CDCl_3$) δ 8.67-8.63 (2H, m), 8.04 (1H, d, J = 2.6 Hz), 7.78 (1H, d, J = 1.7 Hz), 7.67-7.62 (2H, m), 6.53 (1H, dd, J = 2.6, 1.7 Hz); ^{13}C NMR (151 MHz, $CDCl_3$) δ 151.4, 146.1, 142.7, 126.7, 112.6, 109.2. Data in accordance with the literature.[3]

3-(1*H*-Pyrazol-1-yl)pyridine (7a)

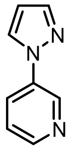

Following **GP1**, 1-*H*-pyrazole (102 mg, 1.5 mmol, 1.0 equiv.) and 3-iodopyridine (308 mg, 1.5 mmol, 1.0 equiv.) gave **7a** (169 mg, 78%) as a white solid. ^{1}H NMR (600 MHz, $CDCl_3$) δ 8.99 (1H, d, J = 2.7 Hz), 8.54 (1H, d, J = 4.7 Hz), 8.05 (1H, ddd, J = 8.3, 2.7, 1.4 Hz), 7.96 (1H, d, J = 2.5 Hz), 7.77 (1H, d, J = 1.8 Hz), 7.40 (1H, dd, J = 8.3, 4.7 Hz), 6.51 (1H, t, J = 2.1 Hz); ^{13}C NMR (151 MHz, $CDCl_3$) δ 147.7, 142.1, 140.7, 136.7, 126.9, 126.7, 124.1, 108.5. Data in accordance with the literature.[4]

4-Methyl-1-phenyl-1*H*-pyrazole (14a)

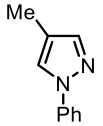

Following **GP1**, 4-methyl-1*H*-pyrazole (123 mg, 1.5 mmol, 1.0 equiv.) and iodobenzene (306 mg, 1.5 mmol, 1.0 equiv.) gave **14a** (202 mg, 85%) as a white solid. ^{1}H NMR (600 MHz, $CDCl_3$) δ 7.70 (1H, t, J = 0.9 Hz), 7.68-7.63 (2H, m), 7.53 (1H, s), 7.43 (2H, t, J = 8.0 Hz), 7.25 (1H, t, J = 7.1 Hz), 2.16 (3H,

s); ^{13}C NMR (151 MHz, $CDCl_3$) δ 141.9, 140.4, 129.5, 126.1, 125.5, 118.9, 118.3, 9.1. Data in accordance with the literature.[5]

5-Methyl-1-phenyl-1*H*-pyrazole (15a)

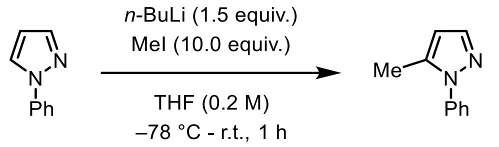

A round-bottom flask equipped with a stirring bar was charged with 1-phenyl-1*H*-pyrazole (433 mg, 3.0 mmol, 1.0 equiv.). The flask was evacuated and refilled with Ar (x 3). THF (15 mL) was added. The reaction was then cooled to –78 °C. *n*-BuLi (1.6 M in *n*-pentane, 2.8 mL, 4.5 mmol, 1.5 equiv.) was added dropwise and the mixture was maintained at this temperature for 1 h. MeI (1.8 mL, 30.0 mmol, 10.0 equiv.) was added and the resulting mixture was allowed to warm to r.t. and stirred at r.t. for 3 h. After completion of the reaction as monitored by TLC, NH_4Cl(sat.) (15 mL) was added into reaction. The layers were separated, and the aqueous layer was extracted with ether (15 mL x 3). The combined organic layers were washed with brine (20 mL x 1), dried (Na_2SO_4) and filtered. The solvent was evaporated, and the residue was purified by column chromatography on silica gel to give **15a** (190 mg, 88%) as an oil. R_f 0.19 [hexane:EtOAc (1:1)]; ^{1}H NMR (600 MHz, $CDCl_3$) δ 7.58 (1H, d, J = 1.8 Hz), 7.49-7.44 (4H, m), 7.38 (1H, tt, J = 6.4, 2.1 Hz), 6.20 (1H, s), 2.35 (3H, s); ^{13}C NMR (151 MHz, $CDCl_3$) δ 140.1, 140.0, 138.8, 129.2, 127.7, 125.0, 107.0, 12.5; HRMS (ESI): found MNa^+ 181.0736, $C_{10}H_{10}N_2Na$ requires 181.0736. Data in accordance with the literature.[6]

Methyl 1-Phenyl-1*H*-pyrazole-3-carboxylate (16a)

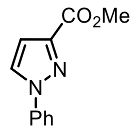

Following **GP1**, methyl pyrazole-3-carboxylate (189 mg, 1.5 mmol, 1.0 equiv.) and iodobenzene (306 mg, 1.5 mmol, 1.0 equiv.) gave **16a** (88 mg, 29%) as a white solid. ^{1}H NMR (600 MHz, $CDCl_3$) δ 7.94 (1H, dd, J = 2.5, 0.8 Hz), 7.76-7.71 (2H, m), 7.47 (2H, t, J = 8.0 Hz), 7.36 (1H, t, J = 7.4 Hz), 7.01 (1H, d, J = 2.5 Hz), 3.97 (3H, s); ^{13}C NMR (151 MHz, $CDCl_3$) δ 162.8, 145.0, 139.8, 129.6, 128.6, 127.8, 120.3, 110.6, 52.3. Data in accordance with the literature.[7]

Methyl 1-Phenyl-1*H*-pyrazole-5-carboxylate (18a)

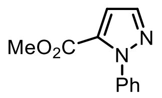

Following **GP4b**, 1-phenyl-1*H*-pyrazole-5-carboxylic acid (376 mg, 2 mmol, 1.0 equiv.) gave **18a** (217 mg, 54%) as a white solid. ^{1}H NMR (400 MHz, $CDCl_3$) δ 7.69 (1H, d, *J* = 2.0 Hz), 7.51-7.39 (5H, m), 7.02 (1H, d, *J* = 2.1 Hz), 3.79 (3H, s); ^{13}C NMR (101 MHz, $CDCl_3$) δ 159.7, 140.4, 139.8, 133.1, 128.8, 128.7, 126.1, 112.7, 52.1. Data in accordance with the literature.[8]

3-Methoxy-1-phenyl-1*H*-pyrazole (19a)

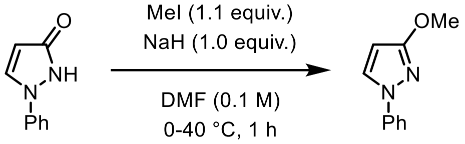

A flask equipped with a stirring bar was charged with 1-phenyl-1*H*-pyrazol-3-one (320 mg, 2.0 mmol, 1.0 equiv.). The flask was evacuated and refilled with Ar (x 3). Dry DMF (20 mL) and NaH (as a 60% dispersion in mineral oil, 80 mg, 2.0 mmol, 1.0 equiv.) were added at 0 °C. The reaction was stirred at 0 °C for 15 min. MeI (0.14 mL, 2.2 mmol, 1.1 equiv.) was added dropwise. The reaction was stirred at 40 °C for 1 h. After completion of the reaction as monitored by TLC, the mixture was diluted with H_2O (15 mL). The layers were separated, and the aqueous layer was extracted with EtOAc (20 mL x 3). The combined organic layers were washed with brine (20 mL x 1), dried (Na_2SO_4) and filtered. The solvent was evaporated, and the residue was purified by column chromatography on silica gel to give **19a** (282 mg, 81%) as a yellow oil. ^{1}H NMR (600 MHz, $CDCl_3$) δ 7.73 (1H, d, *J* = 2.6 Hz), 7.61 (2H, dd, *J* = 8.7, 1.1 Hz), 7.41 (2H, dd, *J* = 8.6, 7.4 Hz), 7.20 (1H, tt, *J* = 7.4, 1.2 Hz), 5.89 (1H, d, *J* = 2.6 Hz), 3.99 (3H, s); ^{13}C NMR (151 MHz, $CDCl_3$) δ 165.2, 140.4, 129.5, 127.9, 125.4, 118.0, 93.6, 56.5. Data in accordance with the literature.[9]

4-Methoxy-1-phenyl-1*H*-pyrazole (20a)

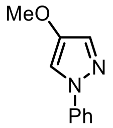

Following **GP1**, 4-methoxy-1*H*-pyrazole (147 mg, 1.5 mmol, 1.0 equiv.) and iodobenzene (306 mg, 1.5 mmol, 1.0 equiv.) gave **20a** (219 mg, 84%) as a colourless oil. R_f 0.27 [hexane:EtOAc (6:1)]; ^{1}H NMR (600 MHz, $CDCl_3$) δ 7.63 (2H, dd, *J* = 8.7, 1.1 Hz), 7.58 (1H, s), 7.48 (1H, s), 7.46-7.40 (2H, m), 7.25 (1H, t, *J* = 7.4 Hz), 3.82 (3H, s); ^{13}C NMR (151 MHz, $CDCl_3$) δ 148.7, 140.6, 129.6, 129.5, 126.1, 118.5, 111.1, 59.0; HRMS (EI): found M^+ 174.0787, $C_{10}H_{10}N_2O$ requires 174.0788.

1-Phenyl-3-(trifluoromethyl)-1*H*-pyrazole (22a)

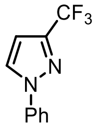

Following **GP1**, 3-(trifluormethyl)-1*H*-pyrazole (204 mg, 1.5 mmol, 1.0 equiv.) and iodobenzene (306 mg, 1.5 mmol, 1.0 equiv.) gave **22a** (252 mg, 79%) as a yellow oil. ^{1}H NMR (600 MHz, $CDCl_3$) δ 7.94 (1H, s), 7.70 (2H, d, *J* = 8.4 Hz), 7.48 (2H, t, *J* = 7.6 Hz), 7.37 (1H, t, *J* = 7.3 Hz), 6.72 (1H, d, *J* = 2.5 Hz); ^{13}C NMR (151 MHz, $CDCl_3$) δ 144.1 (q, *J* = 38.3 Hz), 139.5, 129.7, 128.5, 127.9, 121.4 (q, *J* = 268.5 Hz), 120.0, 106.1 (q, *J* = 2.1 Hz); ^{19}F NMR (565 MHz, $CDCl_3$) δ –62.0. Data in accordance with the literature.[10]

1-Phenyl-4-(trifluoromethyl)-1*H*-pyrazole (23a)

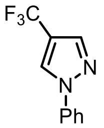

Following **GP1**, 4-(trifluoromethyl)-1*H*-pyrazole (204 mg, 1.5 mmol, 1.0 equiv.) and iodobenzene (306 mg, 1.5 mmol, 1.0 equiv.) gave **23a** (254 mg, 80%) as a white solid. ^{1}H NMR (600 MHz, $CDCl_3$) δ 8.18 (1H, s), 7.91 (1H, s), 7.68 (2H, dd, *J* = 8.6, 1.2 Hz), 7.49 (2H, t, *J* = 7.9 Hz), 7.37 (1H, t, *J* = 7.4 Hz); ^{13}C NMR (151 MHz, $CDCl_3$) δ 139.5, 138.3 (q, *J* = 2.7 Hz), 129.8, 127.9, 126.4 (q, *J* = 3.7 Hz), 122.6 (q, *J* = 267.3 Hz), 119.9, 115.6 (q, *J* = 38.5 Hz); ^{19}F NMR (565 MHz, $CDCl_3$) δ –56.7. Data in accordance with the literature.[11]

1,4-Diphenyl-1*H*-pyrazole (26a)

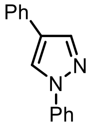

Following **GP1**, 4-phenyl-pyrazole (216 mg, 1.5 mmol, 1.0 equiv.) and iodobenzene (306 mg, 1.5 mmol, 1.0 equiv.) gave **26a** (176 mg, 53%) as a yellow solid. ^{1}H NMR (600 MHz, $CDCl_3$) δ 8.16 (1H, s), 8.01 (1H, s), 7.75 (2H, d, *J* = 7.5 Hz), 7.57 (2H, d, *J* = 6.9 Hz), 7.48 (2H, t, *J* = 8.0 Hz), 7.41 (2H, t, *J* = 7.6 Hz), 7.32 (1H, t, *J* = 7.3 Hz), 7.29 (1H, t, *J* = 7.4 Hz); ^{13}C NMR (151 MHz, $CDCl_3$) δ 140.2, 138.9, 132.2, 129.6, 129.1, 127.0, 126.7, 125.8, 125.0, 123.4, 119.2. Data in accordance with the literature.[12]

1,5-Diphenyl-1*H*-pyrazole (27a)

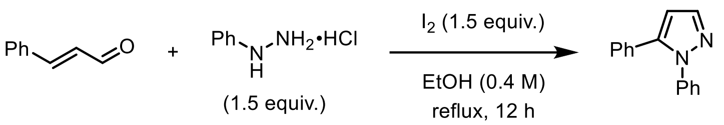

A flask equipped with a stirring bar was charged with *trans*-cinnamaldehyde (264 mg, 2.0 mmol, 1.0 equiv.), phenylhydrazine hydrochloride (434 mg, 3.0 mmol, 1.5 equiv.) and I_2 (761 mg, 3.0 mmol, 1.5 equiv.). The flask was evacuated and refilled with Ar (x 3). EtOH (5 mL) was added. The reaction was stirred under reflux for 12 h. After completion of the reaction as monitored by TLC, the mixture was quenched with 5% $Na_2S_2O_3$ (10 mL). The layers were separated, and the aqueous layer was extracted with EtOAc (10 mL x 3). The combined organic layers were washed with brine (15 mL x 1), dried (Na_2SO_4) and filtered. The solvent was evaporated, and the residue was purified by column chromatography on silica gel to give **27a** (366 mg, 55%) as a yellow oil. ^{1}H NMR (600 MHz, $CDCl_3$) δ 7.73 (1H, d, *J* =1.8 Hz), 7.36-7.28 (8H, m), 7.25-7.22 (2H, m), 6.52 (1H, d, *J* = 1.8 Hz); ^{13}C NMR (151 MHz, $CDCl_3$) δ 143.1, 140.4, 140.3, 130.8, 129.0, 128.9, 128.6, 128.3, 127.5, 125.3, 108.0. Data in accordance with the literature.[13]

1-Methyl-4-phenyl-1*H*-pyrazole (29a)

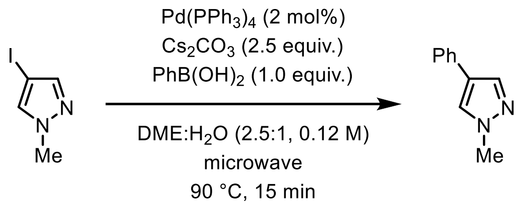

A microwave vial equipped with a stirring bar was charged with 4-iodo-1-methyl-1*H*-pyrazole (416 mg, 2.0 mmol, 1.0 equiv.), phenylboronic acid (244 mg, 2.0 mmol, 1.0 equiv.), $Pd(PPh_3)_4$ (46 mg, 0.04 mmol, 2 mol%) and Cs_2CO_3 (1.7 g, 5.0 mmol, 2.5 equiv.). The vial was evacuated and refilled with Ar (x 3). DME (12 mL) and H_2O (5 mL) were added. The reaction was irradiated in a microwave apparatus at 90 °C for 15 min. After completion of the reaction as monitored by TLC, the mixture was cooled to r.t. and the solvent was evaporated. The residue was purified by column chromatography on silica gel to give **29a** (92 mg, 29%) as a white solid. ^{1}H NMR (600 MHz, $CDCl_3$) δ 7.76 (1H, s), 7.61 (1H, s), 7.48-7.46 (2H, m), 7.36 (2H, t, J = 7.8 Hz), 7.24-7.20 (1H, m), 3.95 (3H, s); ^{13}C NMR (101 MHz, $CDCl_3$) δ 136.9, 132.8, 129.0, 127.0, 126.5, 125.7, 123.4, 39.2. Data in accordance with the literature.[14]

1-Methyl-5-phenyl-1*H*-pyrazole (30a)

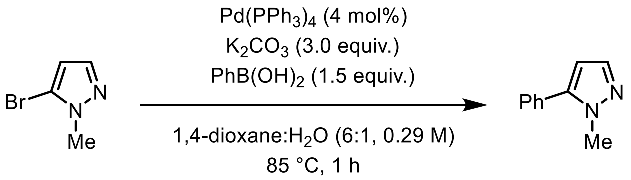

A flask equipped with a stirring bar was charged with 5-bromo-1-methyl-1*H*-pyrazole (322 mg, 2.0 mmol, 1.0 equiv.), phenylboronic (366 mg, 3.0 mmol, 1.5 equiv.), $Pd(PPh_3)_4$ (92 mg, 0.08 mmol, 4 mol%) and K_2CO_3 (829 mg, 6.0 mmol, 3.0 equiv.). The flask was evacuated and refilled with Ar (x 3). 1,4-dioxnae (6 mL) and H_2O (1 mL) were added. The reaction was then stirred at 85 °C for 1 h. After completion of the reaction as monitored by TLC, the mixture was cooled to r.t. and the solvent was evaporated. The residue was purified by column chromatography on silica gel to give **30a** (235 mg, 74%) as a yellow oil. ^{1}H NMR (600 MHz, $CDCl_3$) δ 7.52 (1H, d, J = 1.9 Hz), 7.48-7.44 (2H, m), 7.43-7.39 (3H, m), 6.31 (1H, d, J = 1.9 Hz), 3.90 (3H, s); ^{13}C NMR (101 MHz, $CDCl_3$) δ 143.7, 138.7, 130.9, 128.9, 128.8, 128.5, 106.2, 37.6. Data in accordance with the literature.[15]

1-(4-Methoxyphenyl)-4-phenyl-1*H*-pyrazole (31a)

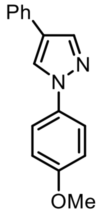

Following **GP2**, 2-phenylmalondialdehyde (296 mg, 2.0 mmol, 1.0 equiv.) and 4-methoxy phenylhydrazine hydrochloride (384 mg, 2.2 mmol, 1.1 equiv.) gave **31a** (648 mg, 80%) as a yellow solid. ^{1}H NMR (600 MHz, $CDCl_3$) δ 8.06 (1H, s), 7.97 (1H, s), 7.63 (2H, d, *J* = 9.0 Hz), 7.55 (2H, d, *J* = 7.1 Hz), 7.40 (2H, t, *J* = 7.8 Hz), 7.27 (1H, t, *J* = 7.2 Hz), 6.99 (2H, d, *J* = 8.9 Hz), 3.86 (3H, s); ^{13}C NMR (151 MHz, $CDCl_3$) δ 158.5, 138.4, 134.0, 132.4, 129.1, 126.9, 125.8, 124.7, 123.6, 120.9, 114.7, 55.7. Data in accordance with the literature.[16]

4-Phenyl-1-(4-(trifluoromethyl)phenyl)-1*H*-pyrazole (32a)

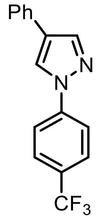

Following **GP2**, 2-phenylmalondialdehyde (296 mg, 2.0 mmol, 1.0 equiv.) and 4-(trifluoromethyl) phenylhydrazine hydrochloride (468 mg, 2.2 mmol, 1.1 equiv.) gave **32a** (478 mg, 83%) as a yellow solid, m.p. 140-141 °C. R_f 0.61 [hexane:EtOAc (19:1)]; ^{1}H NMR (600 MHz, $CDCl_3$) δ 8.20 (1H, s), 8.04 (1H, s), 7.86 (2H, d, *J* = 8.4 Hz), 7.73 (2H, d, *J* = 8.4 Hz), 7.57 (2H, d, *J* = 6.8 Hz), 7.42 (2H, t, *J* = 7.8 Hz), 7.31 (1H, t, *J* = 7.4 Hz); ^{13}C NMR (151 MHz, $CDCl_3$) δ 142.5, 139.9, 131.7, 129.2, 128.5 (q, *J* = 32.8 Hz), 127.4, 126.9 (q, *J* = 3.9 Hz), 125.9, 125.9, 124.1 (q, *J* = 271.8 Hz), 123.3, 118.8; ^{19}F NMR (565 MHz, $CDCl_3$) δ –62.2; HRMS (EI): found M^+ 288.0864, $C_{16}H_9F_3N_2$ requires 288.0869.

4-Phenyl-1-(*o*-tolyl)-1*H*-pyrazole (33a)

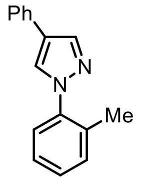

Following **GP2**, 2-phenylmalondialdehyde (296 mg, 2.0 mmol, 1.0 equiv.) and *o*-tolylhydrazine hydrochloride (349 mg, 2.2 mmol, 1.1 equiv.) gave **33a** (500 mg, 73%) as a yellow solid, m.p. 106-107 °C. R_f 0.45 [hexane:EtOAc (19:1)]; ^{1}H NMR (600 MHz, $CDCl_3$) δ 8.01 (1H, s), 7.85 (1H, s), 7.56 (2H, d, J = 7.6 Hz), 7.42-7.37 (3H, m), 7.35-7.29 (3H, m), 7.27 (1H, t, J = 7.4 Hz), 2.32 (3H, s); ^{13}C NMR (151 MHz, $CDCl_3$) δ 140.0, 138.0, 133.8, 132.5, 131.5, 129.1, 128.7, 127.5, 126.8, 126.8, 126.2, 125.8, 123.8, 18.3; HRMS (ESI): found MNa^+ 257.1047, $C_{16}H_{14}N_2Na$ requires 257.1049.

4-Phenyl-1-vinyl-1*H*-pyrazole (34a)

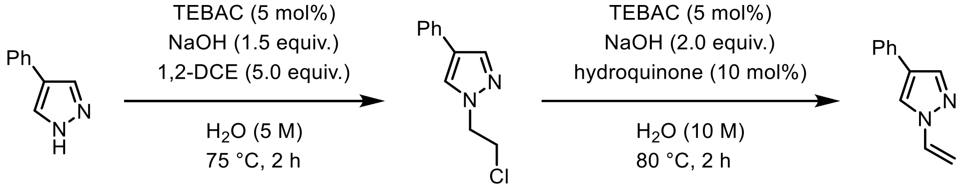

Step 1: A flask equipped with a stirring bar was charged with 4-phenyl-1*H*-pyrazole (288 mg, 2.0 mmol, 1.0 equiv.), NaOH (120 mg, 3.0 mmol, 1.5 equiv.) and benzyltriethylammonium chloride (TEBAC) (23 mg, 0.1 mmol, 5 mol%). The flask was evacuated and refilled with Ar (x 3). 1,2-DCE (0.8 mL, 15 mmol, 5.0 equiv.) and H_2O (0.2 mL) were added. The reaction was stirred at 75 °C for 2 h. After completion of the reaction as monitored by TLC, the mixture was cooled to 20 °C and H_2O (10 mL) was added. The layers were separated, and the aqueous layer was extracted with Et_2O (10 mL x 3). The combined organic layers were washed with brine (15 mL x 1), dried ($MgSO_4$) and filtered. The solvent was evaporated, and the crude product was used in the next reaction without further purification.

Step 2: A flask equipped with a stirring bar was charged with 1-(2-chloroethyl)-4-phenyl-1*H*-pyrazole (251 mg, 1.2 mmol, 1.0 equiv.), NaOH (96 mg, 2.4 mmol, 2.0 equiv.), TEBAC (14 mg, 0.06 mmol, 5 mol%) and hydroquinone (13 mg, 0.12 mmol, 10 mol%). The flask was evacuated and refilled with Ar (x 3). H_2O (0.1 mL) was added. The reaction was warmed to 80 °C and stirred for 2 h. After completion of the reaction as monitored by TLC, the mixture was cooled to 20 °C and H_2O (10 mL) was added into reaction. The layers were separated, and the aqueous layer was extracted with Et_2O (10 mL x 3). The

combined organic layers were washed with brine (15 mL x 1), dried ($MgSO_4$) and filtered. The solvent was evaporated, and the residue was purified by column chromatography on silica gel to give **34a** (135 mg, 40%) as a white solid, m.p. 51-52 °C. R_f 0.42 [hexane:EtOAc (9:1)]; ^1H NMR (600 MHz, $CDCl_3$) δ 7.89 (1H, s), 7.85 (1H, s), 7.53-7.48 (2H, m), 7.38 (2H, t, J = 7.8 Hz), 7.29-7.23 (1H, m), 7.07 (1H, dd, J = 15.8, 8.9 Hz), 5.53 (1H, dd, J = 15.7, 1.5 Hz), 4.87 (1H, dd, J = 8.9, 1.5 Hz); ^{13}C NMR (151 MHz, $CDCl_3$) δ 138.8, 133.2, 132.0, 129.1, 127.1, 125.9, 124.6, 123.7, 99.9; HRMS (EI): found M^+ 170.0839, $C_{11}H_{10}N_2$ requires 170.0844.

4-Phenyl-1-((triisopropylsilyl)ethynyl)-1*H*-pyrazole (35a)

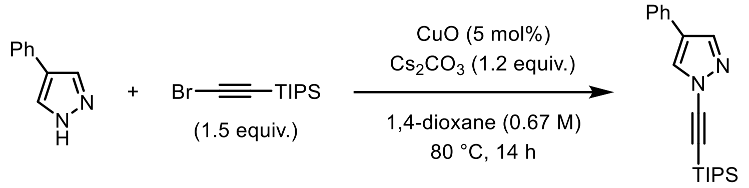

A flask equipped with a stirring bar was charged with 4-phenyl-1H-pyrazole (288 mg, 2.0 mmol, 1.0 equiv.), CuO (19 mg, 0.1 mmol, 5 mol%) and Cs_2CO_3 (782 mg, 2.4 mmol, 1.2 equiv.). The flask was evacuated and refilled with Ar (x 3). (Bromoethynyl)triisopropylsilane (4.0 mmol, 1.0 mL, 2.0 equiv.) and 1,4-dioxane (3 mL) were added. The reaction was warmed to 80 °C and stirred for 14 h. After completion of the reaction as monitored by TLC, the mixture was cooled to 20 °C and H_2O (5 mL) was added. The layers were separated, and the aqueous layer was extracted with EtOAc (10 mL x 3). The combined organic layers were washed with brine (10 mL x 1), dried (Na_2SO_4) and filtered. The solvent was evaporated, and the residue was purified by column chromatography on silica gel to give **35a** (135 mg, 40%) as a yellow oil. R_f 0.42 [hexane:EtOAc (9:1)]; ^1H NMR (600 MHz, $CDCl_3$) δ 7.92-7.88 (2H, m), 7.51-7.47 (2H, m), 7.39 (2H, t, J = 7.8 Hz), 7.29 (1H, tt, J = 7.2, 1.2 Hz), 1.17-1.13 (21H, m); ^{13}C NMR (151 MHz, $CDCl_3$) δ 140.4, 131.0, 130.7, 129.2, 127.5, 126.1, 124.1, 95.0, 70.2, 18.8, 11.4; HRMS (ESI): found MNa^+ 347.1907, $C_{20}H_{28}N_2NaSi$ requires 347.1914.

1-Benzyl-4-phenyl-1*H*-pyrazole (37a)

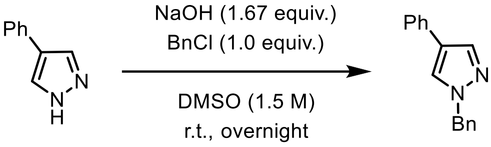

A flask equipped with a stirring bar was charged with 4-phenyl-1*H*-pyrazole (216 mg, 1.5 mmol, 1.0 equiv.), benzyl chloride (190 mg, 1.5 mmol, 1.0 equiv.) and NaOH (100 mg, 2.5 mmol, 1.7 equiv.). The flask was evacuated and refilled with Ar (x 3). DMSO (1 mL) was added. The reaction was stirred at r.t. overnight. After completion of the reaction as monitored by TLC, H_2O (5 mL) was added. The layers were separated, and the aqueous layer was extracted with EtOAc (10 mL x 3). The combined organic layers were washed with brine (15 mL x 1) and $NaHCO_3$(sat.) (10 mL x 1), dried (Na_2SO_4) and filtered. The solvent was evaporated, and the residue was purified by column chromatography on silica gel to give **37a** (275 mg, 78%) as a white solid. ^1H NMR (600 MHz, $CDCl_3$) δ 7.83 (1H, s), 7.62 (1H, s), 7.48-7.45 (2H, m), 7.38-7.30 (5H, m), 7.27 (2H, d, J = 7.5 Hz), 7.21 (1H, t, J = 7.4 Hz), 5.34 (2H, s); ^{13}C NMR (151 MHz, $CDCl_3$) δ 137.2, 136.5, 132.7, 129.0, 129.0, 128.3, 127.9, 126.5, 126.3, 125.7, 123.7, 56.4. Data in accordance with the literature.[17]

***N*,*N*-Dimethyl-1-phenyl-1*H*-pyrazole-4-carboxamide (42a)**

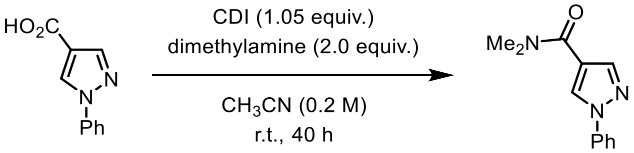

A flask equipped with a stirring bar was charged with 1-phenyl-1*H*-pyrazole-4-carboxylic acid (188 mg, 1.0 mmol, 1.0 equiv.) and 1,1'-carbonyldiimidazole (170 mg, 1.05 mmol, 1.05 equiv.). The flask was evacuated and refilled with Ar (x 3). CH_3CN (5 mL) was added. The reaction was then stirred at r.t. for 2 h. Then dimethylamine (2.0 M in THF, 1.0 mL, 2.0 mmol, 2.0 equiv.) was added and the mixture was stirred at r.t. for 40 h. After completion of the reaction as monitored by TLC, H_2O (10 mL) was added. The layers were separated, and the aqueous layer was extracted with EtOAc (10 mL x 3). The combined organic layers were washed with brine (15 mL x 1), dried (Na_2SO_4) and filtered. The solvent was evaporated, and the residue was purified by column chromatography on silica gel to give **42a** (190 mg, 88%) as a white solid, m.p. 132-133 °C. R_f 0.19 [hexane:EtOAc (1:1)]; ^1H NMR (600 MHz, $CDCl_3$) δ 8.28 (1H, s), 7.90 (1H, s), 7.69 (2H, d, J = 8.0 Hz), 7.46 (2H, t, J = 7.8 Hz), 7.33 (1H, t, J = 7.4 Hz),

3.26 (3H, s), 3.11 (3H, s); ^{13}C NMR (151 MHz, $CDCl_3$) δ 164.4, 140.7, 139.6, 129.7, 129.4, 127.4, 119.6, 119.6, 39.2, 36.1; HRMS (ESI): found MNa^+ 238.0946, $C_{12}H_{13}N_3ONa$ requires 238.0951.

4-Fluoro-1-phenyl-1*H*-pyrazole (45a)

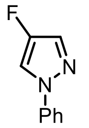

Following **GP1**, 4-Fluoro-1-*H*-pyrazole (129 mg, 1.5 mmol, 1.0 equiv.) and iodobenzene (306 mg, 1.5 mmol, 1.0 equiv.) gave **45a** (210 mg, 86%) as a white solid. ^{1}H NMR (600 MHz, $CDCl_3$) δ 7.80 (1H, dd, J = 4.8, 0.8 Hz), 7.66-7.61 (2H, m), 7.58 (1H, d, J = 4.2 Hz), 7.48-7.39 (2H, m), 7.30 (1H, tt, J = 7.1, 1.2 Hz); ^{13}C NMR (151 MHz, $CDCl_3$) δ 151.2 (d, J = 249.0 Hz), 140.1, 129.5, 128.5 (d, J = 13.8 Hz), 126.7, 118.7, 113.0 (d, J = 28.3 Hz); ^{19}F NMR (565 MHz, $CDCl_3$) δ –175.1 (t, J = 4.5 Hz). Data in accordance with the literature.[18]

3,5-Dimethyl-1'-phenyl-1'*H*-1,4'-bipyrazole (46a)

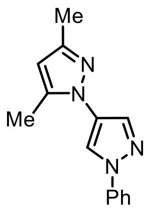

Following **GP1**, 3,5-dimethyl-1*H*-pyrazole (96 mg, 1.0 mmol, 1.0 equiv.) and 4-iodo-1-phenyl-1*H*-pyrazole (270 mg, 1.0 mmol, 1.0 equiv.) gave **46a** (105 mg, 44%) as a white solid, m.p. 92-93 °C. R_f 0.15 [hexane:EtOAc (6:1)]; ^{1}H NMR (600 MHz, $CDCl_3$) δ 8.15 (1H, s), 7.85 (1H, s), 7.71 (2H, d, J = 7.8 Hz), 7.47 (2H, t, J = 7.8 Hz), 7.32 (1H, t, J = 7.4 Hz), 5.97 (1H, s), 2.35 (3H, s), 2.29 (3H, s); ^{13}C NMR (151 MHz, $CDCl_3$) δ 149.4, 140.0, 140.0, 135.2, 129.7, 127.1, 126.2, 121.1, 119.2, 106.8, 13.6, 12.2; HRMS (ESI): found MNa^+ 261.1107, $C_{14}H_{14}N_4Na$ requires 261.1111.

1,3,4-Triphenyl-1*H*-pyrazole (47a)

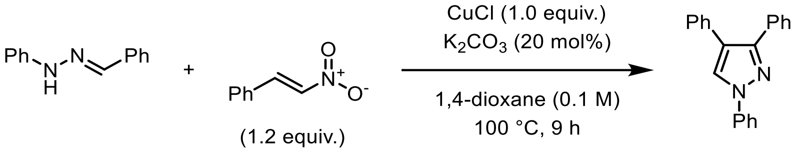

A flask equipped with a stirring bar was charged with (*E*)-(2-nitrovinyl)benzene (358 mg, 2.4 mmol, 1.2 equiv.), benzaldehyde phenylhydrazone (393 mg, 2.0 mmol, 1.0 equiv.), CuCl (198 mg, 2.0 mmol, 1.0 equiv.) and K_2CO_3 (55 mg, 0.2 mmol, 20 mol%). The flask was evacuated and refilled with Ar (x 3) and 1,4-dioxane (20 mL) was added. The reaction was warmed to 90 °C and stirred for 9 h. After completion of the reaction as monitored by TLC, the mixture was cooled to r.t. and H_2O (15 mL) was added into reaction. The layers were separated, and the aqueous layer was extracted with EtOAc (15 mL x 3). The combined organic layers were washed with brine (20 mL x 1), dried (Na_2SO_4) and filtered. The solvent was evaporated, and the residue was purified by column chromatography on silica gel to give **47a** (166 mg, 28%) as a yellow solid. ^{1}H NMR (600 MHz, $CDCl_3$) δ 8.03 (1H, s), 7.81 (2H, d, *J*=8.6 Hz), 7.61 (2H, dt, *J* = 7.6, 1.5 Hz), 7.48 (2H, t, *J* = 8.0 Hz), 7.38-7.28 (9H, m); ^{13}C NMR (151 MHz, $CDCl_3$) δ 150.6, 140.1, 133.3, 133.0, 129.6, 128.8, 128.7, 128.6, 128.4, 128.0, 127.1, 126.8, 126.6, 123.1, 119.1. Data in accordance with the literature.[19]

1,4,5-Triphenyl-1*H*-pyrazole (48a)

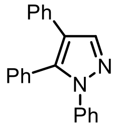

Following **GP3**, 2-phenylacetophenone (981 mg, 5.0 mmol, 1.0 equiv.) gave **48a** (595 mg, 40%) as a white solid. ^{1}H NMR (600 MHz, $CDCl_3$) δ 7.94 (1H, s), 7.36-7.20 (13H, m), 7.19-7.16 (2H, m); ^{13}C NMR (151 MHz, $CDCl_3$) δ 140.1, 139.9, 139.4, 132.9, 130.6, 130.4, 128.8, 128.7, 128.6, 128.6, 128.1, 127.3, 126.5, 125.3, 122.6. Data in accordance with the literature.[20]

5-Methyl-1,4-diphenyl-1*H*-imidazole (49a)

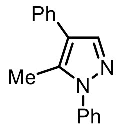

Following **GP1**, 3-methyl-4-phenyl-1*H*-pyrazole (237 mg, 1.5 mmol, 1.0 equiv.) and iodobenzene (306 mg, 1.5 mmol, 1.0 equiv.) gave **49a** (81 mg, 23%) as a white solid. ^{1}H NMR (600 MHz, $CDCl_3$) δ 7.80 (1H, s), 7.52-7.50 (4H, m), 7.47-7.40 (5H, m), 7.30 (1H, tt, J = 6.8, 1.8 Hz), 2.44 (3H, s); ^{13}C NMR (151 MHz, $CDCl_3$) δ 140.0, 139.4, 135.4, 133.7, 129.2, 128.8, 128.0, 127.9, 126.5, 125.3, 122.2, 12.1. Data in accordance with the literature.[20]

3-Methyl-1,4-diphenyl-1*H*-pyrazole (50a)

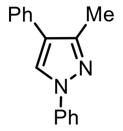

Following **GP1**, 3-methyl-4-phenyl-1*H*-pyrazole (237 mg, 1.5 mmol, 1.0 equiv.) and iodobenzene (306 mg, 1.5 mmol, 1.0 equiv.) gave **50a** (194 mg, 55%) as a colourless oil. R_f 0.51 [hexane:EtOAc (9:1)]; ^{1}H NMR (600 MHz, $CDCl_3$) δ 7.98 (1H, s), 7.71 (2H, d, J = 7.5 Hz), 7.51-7.38 (6H, m), 7.31 (1H, t, J = 7.3 Hz), 7.27 (1H, t, J = 7.5 Hz), 2.51 (3H, s); ^{13}C NMR (151 MHz, $CDCl_3$) δ 148.1, 140.1, 133.2, 129.5, 128.8, 127.8, 126.7, 126.2, 125.3, 123.5, 118.8, 13.5; HRMS (ESI): found MNa^+ 257.1046, $C_{16}H_{14}N_2Na$ requires 257.1049.

Methyl 5-Methyl-1-phenyl-1*H*-pyrazole-3-carboxylate (52a)

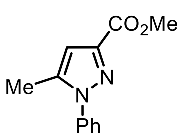

Following **GP4a**, 5-methyl-1-phenyl-1*H*-pyrazole-3-carboxylic acid (303 mg, 1.5 mmol, 1.0 equiv.) gave **52a** (236 mg, 73%) as a yellow oil. ^{1}H NMR (600 MHz, $CDCl_3$) δ 7.49-7.38 (5H, m), 6.73 (1H, d, J = 0.8 Hz), 3.91 (3H, s), 2.32 (3H, d, J = 0.8 Hz); ^{13}C NMR (151 MHz, $CDCl_3$) δ 163.1, 143.6, 140.7, 139.2, 129.2, 128.7, 125.6, 109.3, 52.1, 12.4; HRMS (ESI): found MNa^+ 239.0792, $C_{12}H_{12}N_2O_2Na$ requires 239.0791. Data in accordance with the literature.[21]

Methyl 5-Methyl-1-phenyl-1H-pyrazole-4-carboxylate (53a)

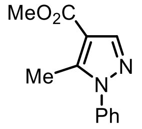

Following **GP4a**, 3-methyl-2-phenyl-4-pyrazolecarboxylic acid (404 mg, 2 mmol, 1.0 equiv.) gave **53a** (367 mg, 85%) as a white solid, m.p. 70-71 °C. R_f 0.31 [hexane:EtOAc (9:1)]; ^{1}H NMR (600 MHz, $CDCl_3$) δ 8.03 (1H, s), 7.51 (2H, t, J = 7.6 Hz), 7.47-7.41 (3H, m), 3.86 (3H, s), 2.57 (3H, s); ^{13}C NMR (151 MHz, $CDCl_3$) δ 164.4, 143.9, 142.0, 138.9, 129.4, 128.9, 125.7, 112.8, 51.4, 12.1; HRMS (EI): found M^+ 216.0893, $C_{12}H_{12}N_2O_2$ requires 216.0893.

4-Methyl-3-phenyl-1*H*-pyrazole (56a)

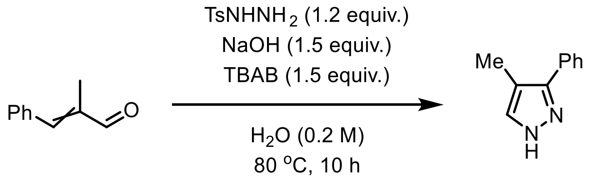

A flask equipped with a stirring bar was charged with α-methylcinnamaldehyde (439 mg, 3.0 mmol, 1.0 equiv.), 4-methylbenzenesulfonhydrazide (620 mg, 3.6 mmol, 1.2 equiv.), NaOH (180 mg, 4.5 mmol, 1.5 equiv.) and TBAB (1451 mg, 4.5 mmol, 1.5 equiv.). The flask was evacuated and refilled with Ar (x 3). H_2O (15 mL) was added. The reaction was warmed to 80 °C and stirred for 10 h. After completion of the reaction as monitored by TLC, the mixture was cooled to r.t. and extracted with EtOAc (20 mL x 3). The combined organic layers were washed with brine (20 mL x 1), dried with Na_2SO_4 and filtered. The solvent was evaporated, and the residue was purified by column chromatography on silica gel to give **56a** (434 mg, 92%) as a white solid. ^{1}H NMR (600 MHz, $CDCl_3$) δ 11.55 (1H, br s), 7.60 (2H, d, J = 7.6 Hz), 7.43 (2H, t, J = 7.7 Hz), 7.40 (1H, s), 7.35 (1H, tt, J = 6.7, 1.3 Hz), 2.24 (3H, s); ^{13}C NMR (151 MHz, $CDCl_3$) δ 128.8, 127.8, 127.5, 113.2, 9.8. Data in accordance with the literature.[22]

Methyl 4-Methyl-1*H*-pyrazole-3-carboxylate (58a)

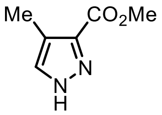

Following **GP4a**, 4-methyl-1*H*-pyrazole-3-carboxylic acid (631 mg, 2 mmol, 1.0 equiv.) gave **58a** (254 mg, 36%) as a white solid. ^{1}H NMR (400 MHz, $CDCl_3$) δ 11.09 (1H, br s), 7.47 (1H, s), 3.93 (3H, s),

2.31 (3H, s); ^{13}C NMR (151 MHz, $CDCl_3$) δ 162.2, 120.6, 51.9, 31.1, 9.7. Data in accordance with the literature.[23]

3,4,5-Trimethyl-1-phenyl-1*H*-pyrazole (61a)

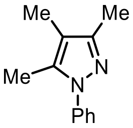

Following **GP2**, 3-methylacetylaceton (0.23 mL, 2.0 mmol, 1.0 equiv.) and phenylhydrazine (238 mg, 2.2 mmol, 1.1 equiv.) gave **61a** (350 mg, 94%) as a brown oil. ^{1}H NMR (600 MHz, $CDCl_3$) δ 7.45-7.39 (4H, m), 7.31 (1H, tt, *J* = 6.6, 1.6 Hz), 2.25 (3H, s), 2.22 (3H, s), 1.98 (3H, s); ^{13}C NMR (151 MHz, $CDCl_3$) δ 148.2, 140.4, 136.1, 129.1, 127.0, 124.8, 113.4, 12.0, 11.1, 8.3. Data in accordance with the literature.[24]

1-Phenyl-4,5,6,7-tetrahydro-1H-indazole (62a)

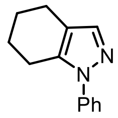

Following **GP1**, 4,5,6,7-tetrahydro-1*H*-indazole (183 mg, 1.5 mmol, 1.0 equiv.) and iodobenzene (306 mg, 1.5 mmol, 1.0 equiv.) gave 62a (89 mg, 30%) as a yellow oil. ^{1}H NMR (600 MHz, $CDCl_3$) δ 7.52-7.47 (2H, m), 7.47 (1H, s), 7.44 (2H, t, *J* = 7.9 Hz), 7.30 (1H, t, *J* = 7.4 Hz), 2.73 (2H, t, *J* = 5.8 Hz), 2.59 (2H, t, *J* = 5.8 Hz), 1.84-1.76 (4H, m); ^{13}C NMR (151 MHz, $CDCl_3$) δ 140.2, 138.8, 138.2, 129.0, 126.6, 123.0, 117.8, 23.7, 23.2, 22.8, 20.7. Data in accordance with the literature.[25]

1-Phenyl-1,4,5,6-tetrahydrocyclopenta[*c*]pyrazole (63a)

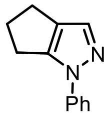

Following **GP1**, 1,4,5,6-tetrahydrocyclopenta[*c*]pyrazole (216 mg, 2.0 mmol, 1.0 equiv.) and iodobenzene (408 mg, 2.0 mmol, 1.0 equiv.) gave **63a** (76 mg, 21%) as a white solid. ^{1}H NMR (600 MHz, $CDCl_3$) δ 7.66-7.63 (2H, m), 7.44-7.40 (2H, m), 7.38 (1H, s), 7.23 (1H, t, *J* = 7.4 Hz), 3.02-2.98 (2H, m), 2.70-2.66 (2H, m), 2.62 (2H, p, *J* = 7.2 Hz); ^{13}C NMR (151 MHz, $CDCl_3$) δ 148.6, 140.6, 135.0,

129.5, 129.4, 125.7, 119.2, 31.1, 26.6, 22.9; HRMS (ESI): found MNa^+ 207.0889, $C_{12}H_{12}N_2Na$ requires 207.0893. Data in accordance with the literature.[26]

***tert*-Butyl 1-Phenyl-1,4,6,7-tetrahydro-5*H*-pyrazolo[4,3-*c*]pyridine-5-carboxylate (64a)**

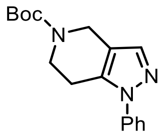

Following **GP1**, *tert*-butyl 6,7-dihydro-1H-pyrazolo[4,3-*c*]pyridine-5(4*H*)-carboxylate (335 mg, 1.5 mmol, 1.0 equiv.) and iodobenzene (306 mg, 1.5 mmol, 1.0 equiv.) gave **64a** (105 mg, 23%) as a colourless oil. R_f 0.09 [hexane:EtOAc (6:1)]; ^{1}H NMR (600 MHz, $CDCl_3$) δ 7.51 (1H, s), 7.50-7.43 (4H, m), 7.32 (1H, t, *J* = 7.0 Hz), 4.51 (2H, s), 3.69 (2H, t, *J* = 7.6 Hz), 2.84 (2H, t, *J* = 5.5 Hz), 1.50 (9H, s); ^{13}C NMR (151 MHz, $CDCl_3$) δ 155.1, 139.7, 136.7, 129.3, 127.1, 122.8, 115.7, 80.2, 60.5, 40.7, 28.6, 24.3, 14.3; HRMS (ESI): found MNa^+ 322.1523, $C_{17}H_{21}N_3O_2Na$ requires 322.1526.

***tert*-Butyl 1-Phenyl-4,6-dihydropyrrolo[3,4-*c*]pyrazole-5(1*H*)-carboxylate (65a)**

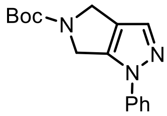

Following **GP1**, *tert*-butyl 4,6-dihydropyrrolo[3,4-*c*]pyrazole-5(1*H*)-carboxylate (419 mg, 2.0 mmol, 1.0 equiv.) and iodobenzene (408 mg, 2.0 mmol, 1.0 equiv.) gave **65a** (70 mg, 12%) as a white solid, m.p. 126-127 °C. R_f 0.23 [hexane:EtOAc (6:1)]; ^{1}H NMR (600 MHz, $CDCl_3$, rotamers) δ 7.56 (2H, t, *J* = 8.4 Hz), 7.50-7.43 (3H, m), 7.30-7.27 (1H, m), 4.79 (1H, t, *J* = 2.5 Hz), 4.73 (1H, t, *J* = 2.4 Hz), 4.49 (1H, t, *J* = 2.5 Hz), 4.45 (1H, t, *J* = 2.5 Hz), 1.53 (9H, d, *J* = 6.2 Hz); ^{13}C NMR (151 MHz, $CDCl_3$, rotamers) δ 154.6, 154.3, 141.7, 141.5, 139.8, 139.7, 133.8, 133.5, 129.7, 126.5, 126.4, 123.5, 123.2, 118.8, 118.6, 80.4, 80.3, 47.1, 47.0, 45.7, 45.3, 28.7, 28.6; HRMS (ESI): found MNa^+ 308.1373, $C_{16}H_{19}N_3O_2Na$ requires 308.1370.

1-Phenyl-1,4,6,7-tetrahydropyrano[4,3-*c*]pyrazole (66a)

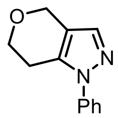

Following **GP3**, tetrahydro-4*H*-pyran-4-one (300 mg, 3.0 mmol, 1.0 equiv.) gave **66a** (142 mg, 24%) as a yellow solid, m.p. 82-83 °C. R_f 0.21 [hexane:EtOAc (6:1)]; ^{1}H NMR (600 MHz, $CDCl_3$) δ 7.51 (2H, d, J = 8.1 Hz), 7.48-7.43 (3H, m), 7.33 (1H, t, J = 7.3 Hz), 4.76 (2H, s), 3.91 (2H, t, J = 5.3 Hz), 2.89 (2H, t, J = 5.2 Hz); ^{13}C NMR (151 MHz, $CDCl_3$) δ 139.7, 135.8, 135.3, 129.4, 127.1, 122.7, 116.5, 64.3, 63.6, 25.0; HRMS (ESI): found MNa^+ 223.0839, $C_{12}H_{12}N_2O_2Na$ requires 223.0842.

1-Phenyl-4,6-dihydro-1*H*-furo[3,4-*c*]pyrazole (67a)

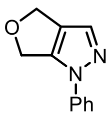

Following **GP3**, but in step 1, DMFDMA (5 mL, 37.3 mmol, 9.3 equiv.) was used and the reaction was warmed to 110 ºC and stirred for 3 h; in step 2, only AcOH (8 mL) and $PhNHNH_2$ (0.5 mL, 5.0 mmol, 1.25 equiv.) were added and the reaction was stirred at r.t. for 48 h. Dihydrofuran-3(2*H*)-one (344 mg, 4.0 mmol, 1.0 equiv.) gave **67a** (613 mg, 82%) as a red solid, m.p. 82-83 °C. R_f 0.58 [hexane:EtOAc (3:1)]; ^{1}H NMR (600 MHz, $CDCl_3$) δ 7.50-7.47 (2H, m), 7.46-7.41 (3H, m), 7.26 (1H, tt, J = 7.2, 1.3 Hz), 5.15 (2H, t, J = 2.8 Hz), 4.95 (2H, t, J = 2.8 Hz); ^{13}C NMR (151 MHz, $CDCl_3$) δ 145.8, 139.7, 132.6, 129.7, 127.5, 126.3, 118.3, 67.1, 66.7; HRMS (ESI): found MNa^+ 209.0686, $C_{11}H_{10}N_2ONa$ requires 209.0685.

Ethyl 1-Phenyl-1,4,6,7-tetrahydropyrano[4,3-*c*]pyrazole-3-carboxylate (68a)

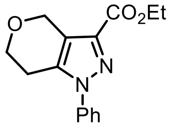

Following **GP1**, ethyl 1,4,6,7-tetrahydropyrano[4,3-*c*]pyrazole-3-carboxylate (589 mg, 3.0 mmol, 1.0 equiv.) and iodobenzene (612 mg, 3.0 mmol, 1.0 equiv.) gave **68a** (219 mg, 27%) as a white solid, m.p. 104-105 °C. R_f 0.12 [hexane:EtOAc (6:1)]; ^{1}H NMR (600 MHz, $CDCl_3$) δ 7.56-7.52 (2H, m), 7.48 (2H, t, J = 7.9 Hz), 7.39 (1H, tt, J = 7.2, 1.2 Hz), 4.93 (2H, s), 4.41 (2H, q, J = 7.1 Hz), 3.91 (2H, t, J = 5.4 Hz), 2.86 (2H, tt, J = 5.4, 1.4 Hz), 1.40 (3H, t, J = 7.1 Hz); ^{13}C NMR (151 MHz, $CDCl_3$) δ 162.7, 139.6,

138.9, 137.5, 129.4, 128.3, 123.7, 119.8, 64.3, 63.9, 61.1, 24.8, 14.5; HRMS (ESI): found MNa$^+$ 295.1045, $C_{15}H_{16}N_2O_3Na$ requires 295.1053.

3-Phenyl-5,6-dihydro-4*H*-pyrrolo[1,2-*b*]pyrazole (70a)

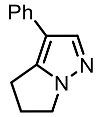

Following **GP5**, *L*-proline (1151 mg, 10.0 mmol, 1.0 equiv.) gave **70a** (108 mg, 6%) as a white solid. ^{1}H NMR (600 MHz, $CDCl_3$) δ 7.81 (1H, s), 7.45 (2H, d, *J* = 8.4 Hz), 7.36 (2H, t, *J* = 7.6 Hz), 7.19 (1H, t, *J* = 7.9 Hz), 4.18 (2H, t, *J* = 7.3 Hz), 3.10 (2H, t, *J* = 7.3 Hz), 2.69 (2H, p, *J* = 7.3 Hz); ^{13}C NMR (151 MHz, $CDCl_3$) δ 142.8, 141.1, 133.6, 129.0, 125.8, 125.2, 115.5, 47.7, 26.6, 24.0. Data in accordance with the literature.[12]

2-Phenyl-5,6-dihydro-4*H*-pyrrolo[1,2-*b*]pyrazole (71a)

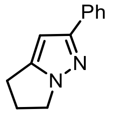

Following **GP5**, *L*-proline (1151 mg, 10.0 mmol, 1.0 equiv.) gave **71a** (423 mg, 23%) as a white solid, m.p. 82-83 °C. R_f 0.20 [hexane:EtOAc (3:1)]; ^{1}H NMR (600 MHz, $CDCl_3$) δ 7.78 (2H, dd, *J* = 7.3, 1.1 Hz), 7.38 (2H, t, *J* = 7.8 Hz), 7.30-7.24 (1H, m), 6.28 (1H, s), 4.19 (2H, t, *J* = 7.2 Hz), 2.92 (2H, t, *J* = 7.4 Hz), 2.61 (2H, p, *J* = 7.3 Hz); ^{13}C NMR (151 MHz, $CDCl_3$) δ 156.3, 147.0, 134.5, 128.7, 127.5, 125.5, 96.0, 47.9, 26.2, 23.3; HRMS (ESI): found MNa$^+$ 207.0892, $C_{12}H_{12}N_2Na$ requires 207.0893.

2-Methylpyrazolo[1,5-*a*]pyridine (73a)

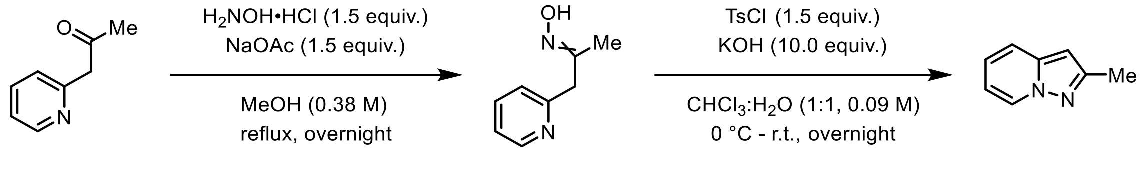

Step 1: A flask equipped with a stirring bar was charged with 1-(pyridin-2-yl)propan-2-one (507 mg, 3.75 mmol, 1.0 equiv.), NaOAc (462 mg, 5.63 mmol, 1.5 equiv.) and hydroxylamine hydrochloride (391 mg, 5.63 mmol, 1.5 equiv.). Dry MeOH (10 mL) was added. The reaction was then stirred under reflux overnight. After completion of the reaction as monitored by TLC, the mixture was cooled to 20°C, concentrated and H_2O (10 mL) was added. The layers were separated, and the aqueous layer was

extracted with EtOAc (15 mL x 3). The combined organic layers were washed with brine (15 mL x 1), dried (Na_2SO_4) and filtered. The solvent was evaporated, and the crude product can be used in the next reaction without further purification.

Step 2: A flask equipped with a stirring bar was charged with 1-(pyridin-2-yl)propan-2-one oxime (563 mg, 3.75 mmol, 1.0 equiv.) and KOH (2103 mg, 37.5 mmol, 10.0 equiv.). The flask was evacuated and refilled with Ar (x 3). $CHCl_3$ (20 mL) and H_2O (20 mL) were added. The reaction was stirred at 0 °C for 10 min. After that, TsCl (1.07 mL, 5.63 mmol, 1.5 equiv.) was added and the reaction was stirred at r.t. overnight. After completion of the reaction as monitored by TLC, the layers were separated, and the aqueous layer was extracted with $CHCl_3$ (20 mL x 3). The combined organic layers were washed with brine (15 mL x 1), dried (Na_2SO_4) and filtered. The solvent was evaporated, and the residue was purified by column chromatography on silica gel to give **73a** (110 mg, 22%) as a pale-yellow oil. R_f 0.24 [hexane:EtOAc (6:1)]; ^{1}H NMR (400 MHz, $CDCl_3$) δ 8.39-8.31 (1H, m), 7.43-7.36 (1H, m), 7.08-6.98 (1H, m), 6.64 (1H, ddd, *J* = 8.6, 5.1, 1.6 Hz), 6.26 (1H, s), 2.48 (3H, s); ^{13}C NMR (101 MHz, $CDCl_3$) δ 151.8, 141.3, 128.2, 123.3, 117.3, 110.8, 96.2, 14.0; HRMS (ESI): found MH^+ 133.0763, $C_8H_9N_2$ requires 133.0760.

Methyl Pyrazolo[1,5-*a*]pyridine-3-carboxylate (74a)

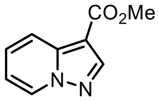

Following **GP4b**, pyrazolo[1,5-*a*]pyridine-3-carboxylic acid (162 mg, 1 mmol, 1.0 equiv.) gave **75a** (156 mg, 89%) as a white solid. ^{1}H NMR (600 MHz, $CDCl_3$) δ 8.53 (1H, dd, *J* = 6.9, 1.1 Hz), 8.40 (1H, s), 8.17 (1H, dd, *J* = 8.8, 1.3 Hz), 7.41 (1H, ddd, *J* = 9.0, 6.8, 1.2 Hz), 6.96 (1H, td, *J* = 6.8, 1.4 Hz), 3.92 (3H, s); ^{13}C NMR (151 MHz, $CDCl_3$) δ 164.0, 145.0, 141.1, 129.5, 127.5, 119.2, 113.9, 103.8, 51.4. Data in accordance with the literature.[27]

Methyl Pyrazolo[1,5-*a*]pyridine-2-carboxylate (75a)

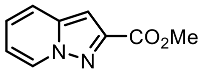

Following **GP4b**, pyrazolo[1,5-*a*]pyridine-2-carboxylic acid (162 mg, 1 mmol, 1.0 equiv.) gave **74a** (156 mg, 89%) as a white solid. ^{1}H NMR (400 MHz, $CDCl_3$) δ 8.51 (1H, dd, *J* = 7.1, 1.2 Hz), 7.59 (1H,

dd, J = 9.0, 1.3 Hz), 7.17 (1H, ddd, J = 9.0, 6.7, 1.1 Hz), 7.07 (1H, s), 6.89 (1H, ddd, J = 6.9, 6.9, 1.4 Hz), 3.99 (3H, s); ^{13}C NMR (101 MHz, $CDCl_3$) δ 163.3, 144.8, 141.0, 129.0, 124.2, 119.4, 114.3, 100.3, 52.5. Data in accordance with the literature.[28]

Methyl pyrazolo[1,5-*b*]pyridazine-3-carboxylate (81a)

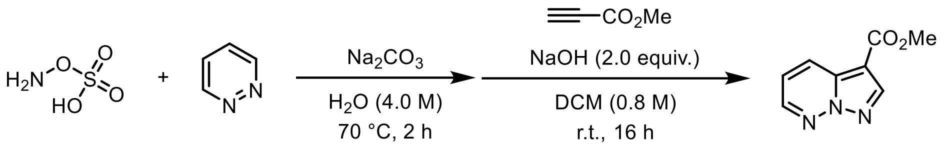

Step 1: A flask equipped with a stirring bar was charged with (aminooxy)sulfonic acid (1.36 g, 12.0 mmol, 6.0 equiv.) and H_2O (0.5 mL). Na_2CO_3(sat) was added to the solution until pH = 6. The reaction was warmed to 70 °C and pyridazine (0.58 mL, 8.0 mmol, 4.0 equiv.) was added dropwise. The reaction was then stirred at the same temperature for 2 h.

Step 2: After completion of the reaction, the reaction was cooled to r.t. and then neutralized with Na_2CO_3(sat.). A solution of methyl propiolate (0.18 mL, 2.0 mmol, 1.0 equiv.) in CH_2Cl_2 (2.5 mL) was added followed by NaOH (160 mg, 4.0 mmol, 2.0 equiv.). The reaction was stirred at r.t. for 16 h. After completion of the reaction, the reaction mixture was diluted with CH_2Cl_2 (20 mL), washed with H_2O (10 mL x 1), brine (10 mL x 1), dried ($MgSO_4$) and filtered. The solvent was evaporated, and the residue was purified by column chromatography on silica gel to give **81a** (200 mg, 56%) as a pale-brown solid. ^{1}H NMR (400 MHz, $CDCl_3$) δ 8.54 (1H, dd, J = 9.0, 2.0 Hz), 8.47 (1H, s), 8.43 (1H, dd, J = 4.4, 2.0 Hz), 7.26 (1H, dd, J = 9.0, 4.5 Hz), 3.93 (3H, s); ^{13}C NMR (101 MHz, $CDCl_3$) δ 163.2, 143.3, 142.5, 135.2, 128.2, 119.3, 104.4, 51.7. Data in accordance with the literature.[29]

N-(3-(3-Cyanopyrazolo[1,5-*a*]pyrimidin-7-yl)phenyl)-*N*-ethylacetamide (82a)

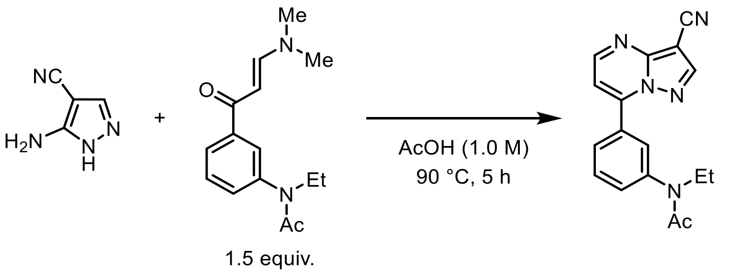

A flask equipped with a stirring bar was charged with *N*-ethyl-*N*-3-((3-dimethylamino-1-oxo-2-propenyl)phenyl)acetamide (1953 mg, 5.0 mmol, 1.0 equiv.) and 3-amino-4-pyrazolecarbonitrile (541

mg, 7.5 mmol, 1.5 equiv.). The flask was evacuated and refilled with Ar (x 3). AcOH (5 mL) was added. The reaction was warmed to 90 °C and stirred for 5.0 h and then cooled to r.t. After completion of the reaction as monitored by TLC, H_2O (10 mL) was added. The layers were separated, and the aqueous layer was extracted with CH_2Cl_2 (10 mL x 3). The combined organic layers were washed with Na_2CO_3 (sat.) (15 mL x 1), dried ($MgSO_4$) and filtered. The solvent was evaporated, and the residue was purified by preparative HPLC to give **82a** (157 mg, 42%) as a pale-yellow solid. ^{1}H NMR (600 MHz, $CDCl_3$) δ 8.81 (1H, s), 8.43 (1H, s), 7.97 (1H, d, *J* = 7.8 Hz), 7.94 (1H, s), 7.68 (1H, t, *J* = 8.0 Hz), 7.45 (1H, d, *J* = 8.0 Hz), 7.20 (1H, d, *J* = 3.9 Hz), 3.82 (2H, q, *J* = 7.6 Hz), 1.93 (3H, s), 1.16 (3H, t, *J* = 7.0 Hz); ^{13}C NMR (151 MHz, $CDCl_3$) δ 169.8, 152.7, 151.4, 147.2, 147.0, 143.5, 131.7, 131.1, 130.5, 129.7, 128.8, 112.7, 110.0, 83.7, 44.1, 23.1, 13.2. Data in accordance with the literature.[30]

4 Reaction Optimization

General Procedure for the Reaction Optimization – GP6

A dry quartz tube equipped with a stirring bar was charged with the pyrazole (0.1 mmol, 1.0 equiv.). The tube was capped with a rubber seal, evacuated and refilled with Ar (x 3). The corresponding degassed solvent was added. The tube was placed into a Helios Quartz photoreactor equipped with 254 nm, 300 nm or 310 nm lamps and a fan. The photoreactor and the fan were switched on and the mixture was stirred under irradiation. The photoreactor and the fan were switched off and 1.0 mL of a stock solution of 1,3-dinitrobenzene in EtOAc (0.1 M) was added. After stirring the new mixture, the solvent was evaporated, and the residue was diluted with $CDCl_3$ (0.5 mL) and analysed by ^{1}H NMR spectroscopy to determine the NMR yield.

4.1 Permutations of 1-Phenyl-1H-pyrazole (1a)

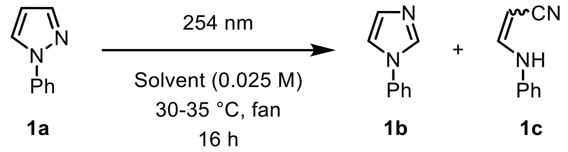

All the reaction parameters were optimized following **GP6** using **1a** (14 mg, 0.1 mmol, 1.0 equiv.).

Table S2. Screening of solvents.

entry	solvent (0.025 M)	1b (%)	1c (%)	1a (%)
**1**	HFIP	**70**	–	–
**2**	CH_3CN	32	28	–
**3**	TFE	33	9	–
**4**	1,4-dioxane	33	40	–
**5**	*i*-PrOH	–	–	trace
**6**	MeOH	26	6	–
**7**	EtOH	20	18	–
**8**	*t*-BuOH	17	10	5
**9**	CH_2Cl_2	–	trace	13
**10**	DCE	–	–	–
**11**	$CHCl_3$	10	–	20

entry	solvent (0.025 M)	1b (%)	1c (%)	1a (%)
**12**	EtOAc	15	23	–
**13**	THF	16	30	14
**14**	Et_2O	11	12	31
**15**	$PhCF_3$	25	10	13
**16**	hexane	7	4	55
**17**	DMF	–	–	–
**18**	DMSO	–	–	–

Table S3. Screening of time using **HFIP** as solvent.

entry	time (h)	1b (%)	1c (%)
**1**	1	**95**	–
**2**	2	93	–
**3**	4	91	–
**4**	8	84	–
**5**	24	68	–

Table S4. Screening of concentration and time using **HFIP** as solvent.

entry	concentration (M)	time (h)	1b (%)	1c (%)
**1**	0.1	**1**	**52**	39
**2**		2	87	2
**3**		3	76	–
**4**	0.05	1	84	–
**5**		1.5	68	–
**6**		2	81	–
**7**	0.025	1	**95**	–
**8**	0.017	0.5	80	18
**9**		1	90	–

Table S5. Screening of additives using **1,4-dioxane** as solvent.

entry	additive (1.0 equiv.)	1b (%)	1c (%)	1a (%)
**1**	none	28	56	–
**2**	AcOH	32	28	–
**3**	TFA	21	29	18
**4**	HFIP	10	15	7
**5**	Urea	26	32	20
**6**	Thiourea	25	30	22
**7**	BzOH	12	16	10

Table S6. Screening of solvents under **300 nm**.

entry	solvent (0.025 M)	1b (%)	1c (%)	1a (%)
**1**	HFIP	–	–	100
**2**	CH3CN	–	–	100
**3**	TFE	–	–	100
**4**	1,4-dioxane	–	–	100

Table S7. Screening of additives using **HFIP** as solvent under **310 nm**.

entry	additive (1.0 equiv.)	Time (h)	1b (%)	1a (%)
**1**	none	16	–	100
**2**	TEA	2	–	100
**3**	LiI	2	–	100
**4**	BzOH	2	–	100
**5**	*N*,*N'*-dimethyl thiourea	2	–	100
**6**	$BF_3 \cdot OEt_2$	2	–	100
**7**	$MgCl_2$	2	–	100

4.2 Permutations of 1,4-Diphenyl-1*H*-pyrazole (26a)

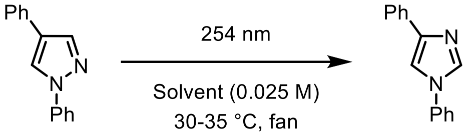

26a → **26b**

All the reaction parameters were optimized following **GP6** using **26a** (22 mg, 0.1 mmol, 1.0 equiv.).

Table S8. Screening of time.

entry	time (h)	26b (%)	26a (%)
**1**	0.5	40	54
**2**	1	75	17
**3**	2	77	–
**4**	3	**78**	–

Table S9. Screening of additives.

entry	additive (1.0 equiv.)	26b (%)	26a (%)
**1**	None	**77**	–
**2**	TEA	73	2
**3**	LiI	40	–
**4**	BzOH	65	5
**5**	Thiourea	73	2
**6**	**A**	71	2
**7**	**B**	41	3
**8**	**C**	81	2
**9**	**D**	73	14
**10**	**E**	50	10
**11**	**F**	71	7
**12**	$BF_3{\cdot}OEt_2$	–	8
**13**	$MgCl_2$	70	4

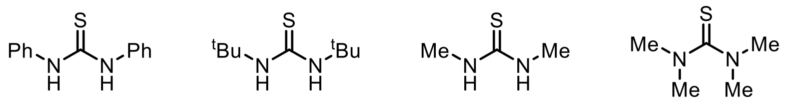

A B C D

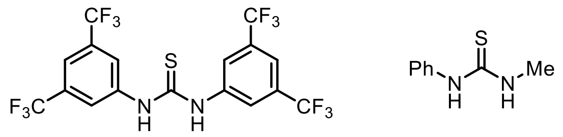

E F

Table S10. Screening of wavelength under 3 h.

entry	wavelength (nm)	26b (%)	26a (%)
**1**	254	78	–
**2**	310	**98**	–

5 Pictures of Reaction Set-up

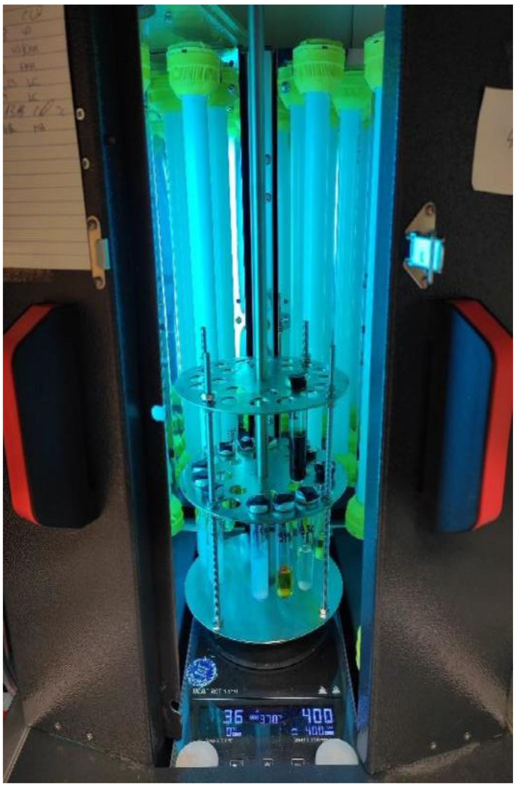

Figure S1. Set-up for 0.1 mmol scale reactions

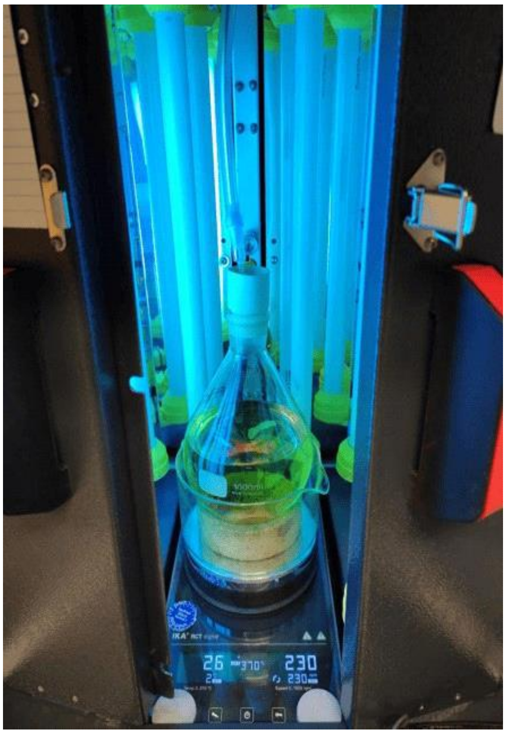

Figure S2. Set-up for 1.0 mmol scale reactions

6 UV-Vis Spectra

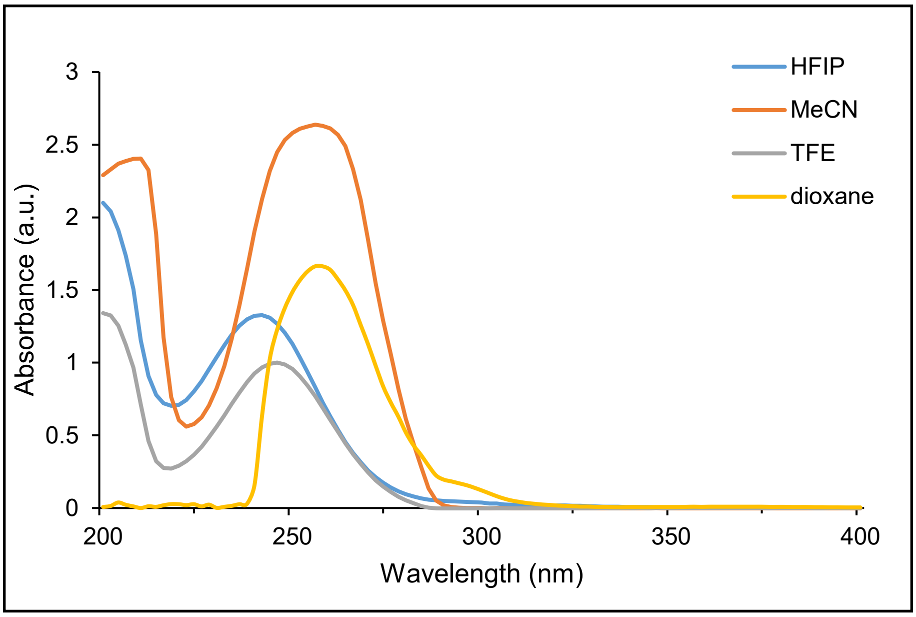

Figure S3. 1a in different solvents (c = 1×10^{-4} M)

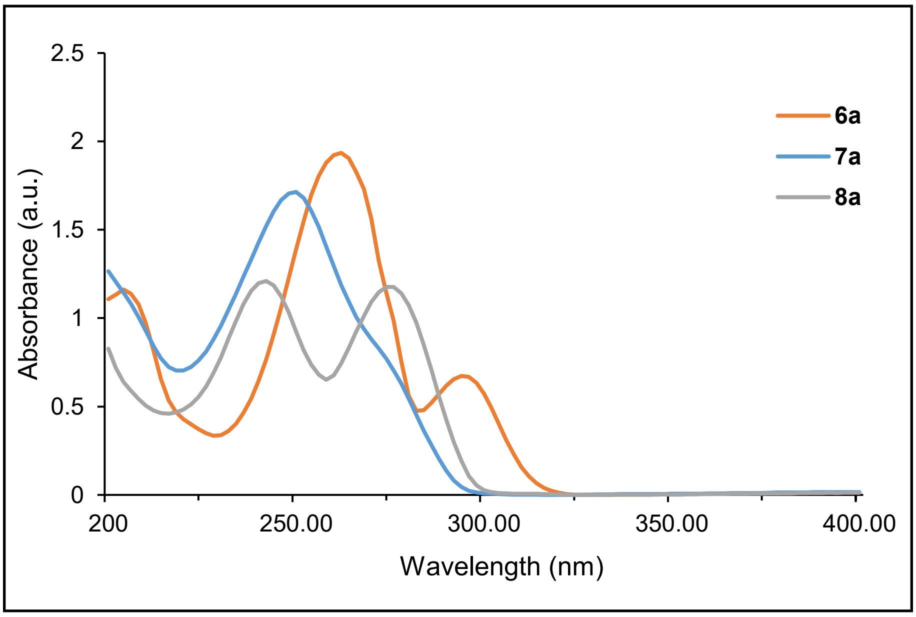

Figure S4. 6a, 7a and **8a** in HFIP (c = 1×10^{-4} M)

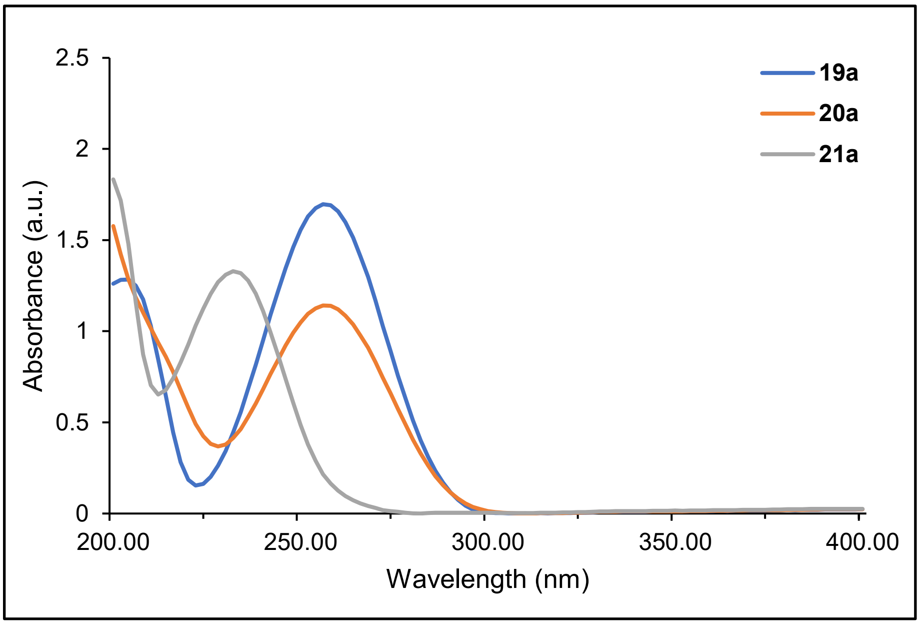

Figure S5. 19a, 20a and **21a** in HFIP (c = 1×10^{-4} M)

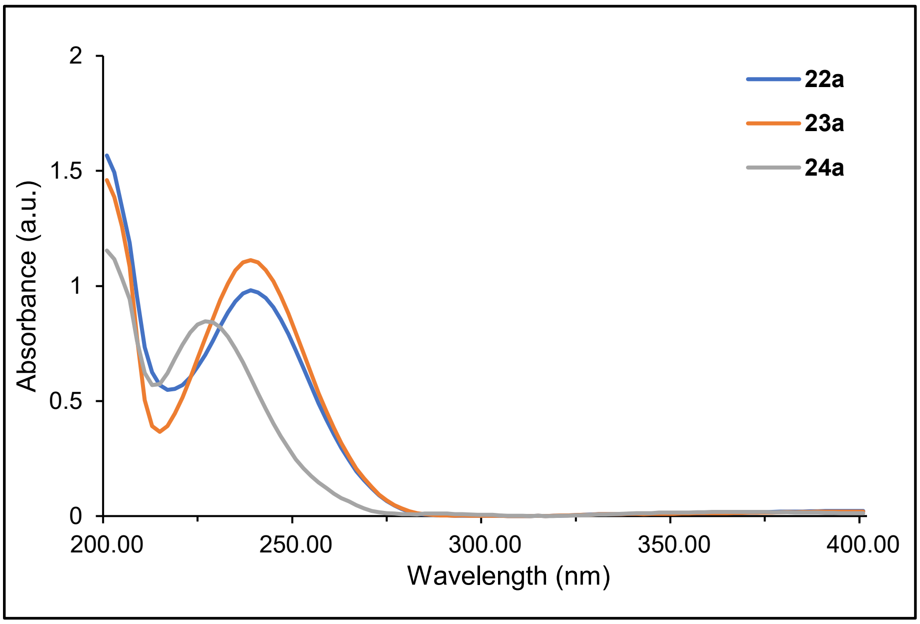

Figure S6. 22a, 23a and **24a** in HFIP (c = 1×10^{-4} M)

7 Substrate Scope

1-Phenyl-1*H*-imidazole (1b)

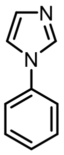

Following **GP*1**, but irradiating the reaction for 1 h, **1a** (14 mg, 0.1 mmol, 1.0 equiv.) gave **1b** (95%) as a yellow oil. ^{1}H NMR (600 MHz, $CDCl_3$) δ 7.85 (1H, s), 7.48 (2H, t, J = 7.5 Hz), 7.41-7.34 (3H, m), 7.28 (1H, s), 7.21 (1H, s); ^{13}C NMR (151 MHz, $CDCl_3$) δ 137.5, 135.7, 130.6, 130.0, 127.6, 121.6, 118.4. Data in accordance with the literature.[31]

Scale-up Reaction: Following **GP*1**, but concentration of 0.10 M and irradiating the reaction for 5 h, **1a** (721 mg, 5 mmol) gave **1b** (577 mg, 80%) as a yellow oil.

1-(*p*-Tolyl)-1*H*-imidazole (2b)

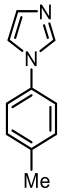

Following **GP*1**, but irradiating the reaction for 3 h, **2a** (16 mg, 0.1 mmol, 1.0 equiv.) gave **2b** (99%) as a yellow oil. ^{1}H NMR (600 MHz, $CDCl_3$) δ 7.81 (1H, t, J = 1.1 Hz), 7.27 (4H, s), 7.24 (1H, t, J = 1.3 Hz), 7.19 (1H, t, J = 1.2 Hz), 2.40 (3H, s); ^{13}C NMR (151 MHz, $CDCl_3$) δ 137.6, 135.8, 135.1, 130.5, 130.4, 121.6, 118.5, 21.1. Data in accordance with the literature.[32]

1-([1,1'-Biphenyl]-4-yl)-1*H*-imidazole (3b)

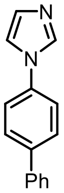

Following **GP*1**, but irradiating the reaction for 60 h, **3a** (22 mg, 0.1 mmol, 1.0 equiv.) gave **3b** (40%) as a white solid and 58% of **3a** was recovered. ^{1}H NMR (400 MHz, $CDCl_3$) δ 7.89 (1H, s), 7.68 (2H, d, J = 8.3 Hz), 7.60 (2H, d, J = 7.1 Hz), 7.51-7.42 (4H, m), 7.38 (1H, tt, J = 6.4, 1.4 Hz), 7.31 (1H, s), 7.23

(1H, s); ^{13}C NMR (101 MHz, $CDCl_3$) δ 140.6, 139.8, 136.6, 135.6, 130.6, 129.0, 128.6, 127.9, 127.1, 121.8, 118.2. Data in accordance with the literature.[33]

1-(4-Methoxyphenyl)-1*H*-imidazole (4b)

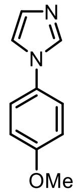

Following **GP*1**, but irradiating the reaction for 24 h, **4a** (17 mg, 0.1 mmol, 1.0 equiv.) gave **4b** (90%) as a brown oil. ^{1}H NMR (600 MHz, $CDCl_3$) δ 7.76 (1H, s), 7.31-7.28 (2H, m), 7.19 (2H, dt, *J* = 11.8, 1.2 Hz), 7.00-6.95 (2H, m), 3.85 (3H, s); ^{13}C NMR (151 MHz, $CDCl_3$) δ 159.1, 136.0, 130.9, 130.2, 123.4, 118.9, 115.0, 55.7. Data in accordance with the literature.[32]

1-(4-(Trifluoromethyl)phenyl)-1*H*-imidazole (5b)

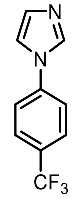

Following **GP*1**, but irradiating the reaction for 2 h, **5a** (21 mg, 0.1 mmol, 1.0 equiv.) gave **5b** (86%) as a white solid. ^{1}H NMR (600 MHz, $CDCl_3$) δ 7.89 (1H, s), 7.72 (2H, d, *J* = 7.7 Hz), 7.50 (2H, d, *J* = 8.2 Hz), 7.30 (1H, s), 7.21 (1H, s); ^{13}C NMR (151 MHz, $CDCl_3$) δ 140.1, 135.5, 131.2, 129.6 (q, *J* = 32.9 Hz), 127.3 (q, *J* = 3.8 Hz), 123.7 (q, *J* = 272.1 Hz), 121.3, 117.9; ^{19}F NMR (565 MHz, $CDCl_3$) δ – 62.5. Data in accordance with the literature.[32]

4-(1*H*-imidazol-1-yl)pyridine (6b)

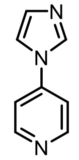

Following **GP*2**, but irradiating the reaction for 24 h, **6a** (15 mg, 0.1 mmol, 1.0 equiv.) gave **6b** (64%) as a white solid and 18% of **6a** was recovered. ^{1}H NMR (600 MHz, $CDCl_3$) δ 8.65-8.61 (2H, m), 7.98

(1H, t, J = 1.1 Hz), 7.34 (1H, t, J = 1.5 Hz), 7.30-7.27 (2H, m), 7.18 (1H, t, J = 1.1 Hz); ^{13}C NMR (151 MHz, $CDCl_3$) δ 151.7, 143.5, 134.9, 131.5, 116.8, 114.4. Data in accordance with the literature.[34]

Following **GP*1**, but irradiating the reaction for 24 h, **6a** (15 mg, 0.1 mmol, 1.0 equiv.) gave **6b** (34%) as a white solid

3-(1*H*-imidazol-1-yl)pyridine (7b)

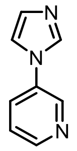

Following **GP*1**, but irradiating the reaction for 24 h, **7a** (15 mg, 0.1 mmol, 1.0 equiv.) gave **7b** (58%) as a white solid. ^{1}H NMR (600 MHz, $CDCl_3$) δ 8.73 (1H, d, J = 2.6 Hz), 8.62 (1H, dd, J = 4.8, 1.4 Hz), 7.86 (1H, s), 7.71 (1H, ddd, J = 8.2, 2.7, 1.5 Hz), 7.43 (1H, dd, J = 8.2, 4.8 Hz), 7.29 (1H, s), 7.24 (1H, s); ^{13}C NMR (151 MHz, $CDCl_3$) δ 148.9, 143.0, 135.6, 134.0, 131.2, 128.9, 124.4, 118.2. Data in accordance with the literature.[4]

2-(1*H*-Imidazol-1-yl)pyridine (8b)

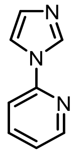

Following **GP*1**, but irradiating the reaction for 66 h, **8a** (20 mg, 0.1 mmol, 1.0 equiv.) gave **8b** (15%) as a white solid and 25% of **8a** was recovered. ^{1}H NMR (600 MHz, $CDCl_3$) δ 8.49 (1H, ddd, J = 4.9, 1.9, 0.9 Hz), 8.35 (1H, t, J = 1.1 Hz), 7.83 (1H, ddd, J = 8.2, 7.5, 1.9 Hz), 7.65 (1H, t, J = 1.4 Hz), 7.36 (1H, dt, J = 8.2, 0.9 Hz), 7.25 (1H, ddd, J = 7.4, 4.8, 0.9 Hz), 7.20 (1H, t, J = 1.2 Hz); ^{13}C NMR (151 MHz, $CDCl_3$) δ 149.4, 139.1, 135.1, 130.9, 122.1, 116.3, 112.5. Data in accordance with the literature.[35]

2-Methyl-1-phenyl-1*H*-imidazole (13b)

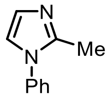

Following **GP*1**, but irradiating the reaction for 8 h, **13a** (16 mg, 0.1 mmol, 1.0 equiv.) gave **13b** (75%) as a brown oil. ^{1}H NMR (600 MHz, $CDCl_3$) δ 7.48 (2H, t, J = 7.6 Hz), 7.42 (1H, t, J = 7.4 Hz), 7.30-

7.28 (2H, m), 7.03 (1H, s), 7.01 (1H, d, *J* = 1.2 Hz), 2.37 (3H, s); ^{13}C NMR (151 MHz, $CDCl_3$) δ 144.9, 138.2, 129.6, 128.3, 127.8, 125.7, 120.8, 13.9. Data in accordance with the literature.[36]

4-Methyl-1-phenyl-1*H*-imidazole (14b)

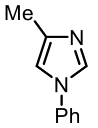

Following **GP*1**, but irradiating the reaction for 3 h, **14a** (16 mg, 0.1 mmol, 1.0 equiv.) gave **14b** (95%) as a yellow solid. ^{1}H NMR (600 MHz, $CDCl_3$) δ 7.75 (1H, s), 7.45 (2H, t, *J* = 7.7 Hz), 7.37-7.30 (3H, m), 7.00 (1H, s), 2.30 (3H, s); ^{13}C NMR (151 MHz, $CDCl_3$) δ 139.7, 137.7, 134.7, 129.9, 127.2, 121.2, 114.7, 13.9. Data in accordance with the literature.[37]

5-Methyl-1-phenyl-1*H*-imidazole (15b)

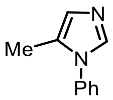

Following **GP*1**, but irradiating the reaction for 6 h, **15a** (16 mg, 0.1 mmol, 1.0 equiv.) gave **15b** (82%) as a yellow oil. R_f 0.11 [hexane:EtOAc (1:1)]; ^{1}H NMR (600 MHz, $CDCl_3$) δ 7.54 (1H, s), 7.50-7.46 (2H, m), 7.42 (1H, tt, *J* = 6.5, 1.3 Hz), 7.30-7.26 (2H, m), 6.89 (1H, s), 2.16 (3H, d, *J* = 1.1 Hz); ^{13}C NMR (151 MHz, $CDCl_3$) δ 137.1, 136.7, 129.6, 128.4, 128.0, 127.5, 125.7, 10.0; HRMS (ESI): found MH^+ 159.0915, $C_{10}H_{11}N_2$ requires 159.0917.

Methyl 1-Phenyl-1*H*-imidazole-2-carboxylate (16b)

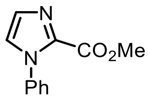

Following **GP*1**, but irradiating the reaction for 60 h, **16a** (14 mg, 0.1 mmol, 1.0 equiv.) gave **16b** (63%) as a pale-yellow solid. R_f 0.20 [hexane:EtOAc (1:1)]; ^{1}H NMR (600 MHz, $CDCl_3$) δ 7.50-7.46 (3H, m), 7.34-7.31 (2H, m), 7.28 (1H, d, *J* = 1.1 Hz), 7.19 (1H, d, *J* = 1.0 Hz), 3.84 (3H, s); ^{13}C NMR (151 MHz, $CDCl_3$) δ 158.9, 138.1, 136.9, 130.1, 129.2, 129.1, 126.7, 126.2, 52.4; HRMS (ESI): found MNa^+ 225.0632, $C_{11}H_{10}N_1O_2Na$ requires 225.0635.

Methyl 1-Phenyl-1*H*-imidazole-4-carboxylate (17b)

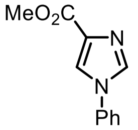

Following **GP*1**, but irradiating the reaction for 115 h, **17a** (20 mg, 0.1 mmol, 1.0 equiv.) gave **17b** (53%) as a yellow solid, m.p. 106-107 °C. R_f 0.42 [EtOAc] ^{1}H NMR (400 MHz, $CDCl_3$) δ 7.96 (1H, d, *J* = 1.5 Hz), 7.86 (1H, d, *J* = 1.4 Hz), 7.57-7.47 (2H, m), 7.46-7.38 (3H, m), 3.93 (3H, s); ^{13}C NMR (101 MHz, $CDCl_3$) δ 163.3, 136.6, 136.5, 135.0, 130.3, 128.6, 124.2, 121.9, 52.0; HRMS (EI): found M^+ 202.0738, $C_{11}H_{10}N_2O_2$ requires 202.0737.

Methyl 1-Phenyl-1*H*-imidazole-5-carboxylate (18b)

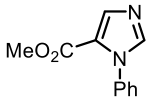

Following **GP*3**, but irradiating the reaction for 36 h, **18a** (20 mg, 0.1 mmol, 1.0 equiv.) gave **18b** (13%) as a yellow solid, m.p. 99-100 °C. R_f 0.19 [hexane:EtOAc (1:1)]; ^{1}H NMR (600 MHz, $CDCl_3$) δ 7.86 (1H, d, *J* = 1.0 Hz), 7.69 (1H, d, *J* = 1.0 Hz), 7.50-7.47 (3H, m), 7.35-7.31 (2H, m), 3.76 (3H, s); ^{13}C NMR (151 MHz, $CDCl_3$) δ 159.9, 142.4, 138.0, 136.2, 129.1, 129.0, 126.2, 123.8, 51.6; HRMS (ESI): found MH^+ 203.0814, $C_{11}H_{11}N_2O_2$ requires 203.0815.

Following **GP*1**, but irradiating the reaction for 36 h, **18a** (20 mg, 0.1 mmol, 1.0 equiv.) gave **18b** (9%) as a yellow solid and 34% of **18a** was recovered.

2-Methoxy-1-phenyl-1*H*-imidazole (19b)

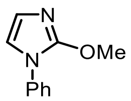

Following **GP*1**, **19a** (17 mg, 0.1 mmol, 1.0 equiv.) gave **19b** (10%) as a brown oil and 29% of **19a** was recovered. R_f 0.20 [hexane:EtOAc (3:1)]; ^{1}H NMR (600 MHz, $CDCl_3$) δ 7.45-7.39 (4H, m), 7.31 (1H, t, *J* = 7.4 Hz), 6.83 (1H, s), 6.75 (1H, s), 4.06 (3H, s); ^{13}C NMR (151 MHz, $CDCl_3$) δ 152.4, 136.6, 129.3, 127.0, 123.9, 123.6, 115.9, 57.0; HRMS (EI): found M^+ 174.0787, $C_{10}H_{10}N_2O$ requires 174.0788.

4-Methoxy-1-phenyl-1*H*-imidazole (20b)

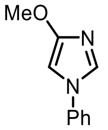

Following **GP*4**, but irradiating the reaction for 3 h, **20a** (17 mg, 0.1 mmol, 1.0 equiv.) gave **20b** (63%) as a dark oil. R_f 0.11 [hexane:EtOAc (3:1)]; ^{1}H NMR (600 MHz, $CDCl_3$) δ 7.50 (1H, d, *J* = 1.7 Hz), 7.47-7.42 (2H, m), 7.37-7.31 (3H, m), 6.60 (1H, d, *J* = 1.7 Hz), 3.86 (3H, s); ^{13}C NMR (151 MHz, $CDCl_3$) δ 158.3, 137.7, 130.3, 129.9, 127.3, 120.9, 96.0, 57.0; HRMS (ESI): found MH^+ 175.0864, $C_{10}H_{11}N_2O$ requires 175.0866.

Following **GP*1**, **20a** (17 mg, 0.1 mmol, 1.0 equiv.) gave **20b** (34%) as a dark oil.

1-Phenyl-2-(trifluoromethyl)-1*H*-imidazole (22b)

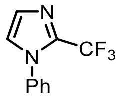

Following **GP*1**, but irradiating the reaction for 6 h, **22a** (21 mg, 0.1 mmol, 1.0 equiv.) gave **22b** (45%) as a brown solid. ^{1}H NMR (600 MHz, $CDCl_3$) δ 7.51-7.46 (3H, m), 7.37-7.34 (2H, m), 7.21 (1H, d, *J* = 1.2 Hz), 7.15 (1H, d, *J* = 1.2 Hz); ^{13}C NMR (151 MHz, $CDCl_3$) δ 136.4, 136.2 (q, *J* = 39.0 Hz), 129.7, 129.5, 128.9, 126.2, 125.2, 118.8 (q, *J* = 270.0 Hz); ^{19}F NMR (565 MHz, $CDCl_3$) δ –59.6. Data in accordance with the literature.[38]

1-Phenyl-4-(trifluoromethyl)-1*H*-imidazole (23b)

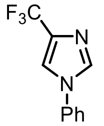

Following **GP*1**, but irradiating the reaction for 3 h, **23a** (21 mg, 0.1 mmol, 1.0 equiv.) gave **23b** (30%) as a brown solid, m.p. 80-81 °C. R_f 0.28 [hexane:EtOAc (6:1)]; ^{1}H NMR (600 MHz, $CDCl_3$) δ 7.85 (1H, s), 7.60 (1H, s), 7.52 (2H, t, *J* = 7.8 Hz), 7.44 (1H, t, *J* = 7.5 Hz), 7.39 (2H, d, *J* = 7.9 Hz); ^{13}C NMR (151 MHz, $CDCl_3$) δ 136.7, 136.4, 133.8 (q, *J* = 39.0 Hz), 130.3, 128.7, 122.0, 121.6 (q, *J* = 267.0 Hz), 118.5 (q, *J* = 3.7 Hz); ^{19}F NMR (565 MHz, $CDCl_3$) δ –62.9; HRMS (EI): found M^+ 212.0555, $C_{10}H_6N_2F_3$ requires 212.0556.

1,4-Diphenyl-1*H*-imidazole (26b)

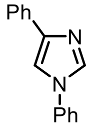

Following **GP*1**, but irradiating the reaction for 3 h and at λ = 310 nm, **26a** (22 mg, 0.1 mmol, 1.0 equiv.) gave **26b** (98%) as a yellow solid, m.p. 91-92 °C. R_f 0.72 [EtOAc] ^{1}H NMR (600 MHz, $CDCl_3$) δ 7.90 (1H, d, *J* = 1.4 Hz), 7.85 (2H, dd, *J* = 7.2, 1.3 Hz), 7.58 (1H, d, *J* = 1.4 Hz), 7.51 (2H, t, *J* = 7.9 Hz), 7.47-7.44 (2H, m), 7.43-7.37 (3H, m), 7.28 (1H, tt, *J* = 7.0, 1.3 Hz); ^{13}C NMR (151 MHz, $CDCl_3$) δ 143.4, 137.5, 135.9, 133.9, 130.1, 128.8, 127.7, 127.3, 125.1, 121.5, 113.9; HRMS (EI): found M^+ 220.0993, $C_{15}H_{12}N_2$ requires 220.0995.

Following **GP*1**, but irradiating the reaction for 3 h, **26a** (22 mg, 0.1 mmol, 1.0 equiv.) gave **26b** (78%) as a yellow solid.

1,5-Diphenyl-1*H*-imidazole (27b)

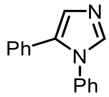

Following **GP*1**, but irradiating the reaction for 36 h, **27a** (22 mg, 0.1 mmol, 1.0 equiv.) gave **27b** (64%) as a yellow solid. ^{1}H NMR (600 MHz, $CDCl_3$) δ 7.71 (1H, s), 7.42-7.37 (3H, m), 7.29-7.23 (4H, m), 7.19 (2H, dd, *J* = 7.7, 2.0 Hz), 7.15 (2H, dd, *J* = 7.4, 2.3 Hz); ^{13}C NMR (151 MHz, $CDCl_3$) δ 139.0, 136.8, 133.1, 129.6, 129.6, 129.0, 128.5, 128.2, 128.2, 127.6, 125.7. Data in accordance with the literature.[39]

1-Methyl-4-phenyl-1*H*-imidazole (29b)

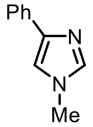

Following **GP*1**, but irradiating the reaction for 3 h, **29a** (16 mg, 0.1 mmol, 1.0 equiv.) gave **29b** (77%) as a yellow solid. ^{1}H NMR (600 MHz, $CDCl_3$) δ 7.76 (1H, s), 7.75 (1H, s), 7.47 (1H, s), 7.37 (2H, t, *J* = 7.7 Hz), 7.25-7.21 (1H, m), 7.17 (1H, s), 3.72 (3H, s); ^{13}C NMR (151 MHz, $CDCl_3$) δ 142.6, 138.2,

134.3, 128.7, 126.9, 124.9, 116.0, 33.7; HRMS (EI): found M^+ 158.0840, $C_{10}H_{11}N_2$ requires 158.0839. Data in accordance with the literature.[40]

1-Methyl-5-phenyl-1*H*-imidazole (30b)

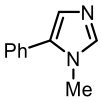

Following **GP*3**, **30a** (16 mg, 0.1 mmol, 1.0 equiv.) gave **30b** (15%) as a yellow solid. ^{1}H NMR (600 MHz, $CDCl_3$) δ 7.51 (1H, s), 7.43 (2H, t, *J* = 7.5 Hz), 7.40-7.34 (3H, m), 7.10 (1H, s), 3.66 (3H, s); ^{13}C NMR (151 MHz, $CDCl_3$) δ 139.2, 133.6, 130.0, 128.8, 128.6, 128.2, 128.0, 32.6. Data in accordance with the literature.[41]

1-(4-Methoxyphenyl)-4-phenyl-1*H*-imidazole (31b)

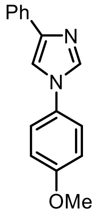

Following **GP*1**, **31a** (25 mg, 0.1 mmol, 1.0 equiv.) gave **31b** (90%) as a yellow solid, m.p. 99-100 °C. R_f 0.63 [EtOAc] ^{1}H NMR (600 MHz, $CDCl_3$) δ 7.84 (2H, dd, *J* = 8.2, 1.0 Hz), 7.80 (1H, d, *J* = 0.9 Hz), 7.49 (1H, d, *J* = 1.3 Hz), 7.40 (2H, t, *J* = 7.8 Hz), 7.37-7.33 (2H, m), 7.27 (1H, t, *J* = 7.3 Hz), 7.02-6.98 (2H, m), 3.86 (3H, s); ^{13}C NMR (151 MHz, $CDCl_3$) δ 159.1, 143.0, 136.1, 134.0, 130.7, 128.8, 127.1, 125.0, 123.2, 115.1, 114.5, 55.8; HRMS (EI): found M^+ 250.1098, $C_{16}H_{14}N_2O$ requires 250.1101.

4-Phenyl-1-(4-(trifluoromethyl)phenyl)-1*H*-imidazole (32b)

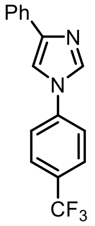

Following **GP*1**, but irradiating at λ = 310 nm, **32a** (29 mg, 0.1 mmol, 1.0 equiv.) gave **32b** (100%) as a white solid, m.p. 156-157 °C. R_f 0.27 [hexane:EtOAc (3:1)] ^{1}H NMR (600 MHz, $CDCl_3$) δ 7.96 (1H, d, *J* = 1.4 Hz), 7.85 (2H, dd, *J* = 8.3, 1.3 Hz), 7.78 (2H, d, *J* = 8.5 Hz), 7.61 (1H, d, *J* = 1.4 Hz), 7.58

(2H, d, $J = 8.4$ Hz), 7.42 (2H, t, $J = 7.8$ Hz), 7.30 (1H, tt, $J = 7.2$, 1.2 Hz); ^{13}C NMR (151 MHz, $CDCl_3$) δ 144.0, 139.9, 135.6, 133.4, 129.6 (q, $J = 33.2$ Hz), 128.8, 127.6, 127.4 (q, $J = 3.8$ Hz), 125.1, 123.8 (q, $J = 272.1$ Hz), 121.1, 113.2; ^{19}F NMR (564 MHz, $CDCl_3$) δ –62.5; HRMS (EI): found M^+ 288.0865, $C_{16}H_{11}N_2F_3$ requires 288.0869.

Following **GP*1**, **32a** (29 mg, 0.1 mmol, 1.0 equiv.) gave **32b** (93%) as a white solid.

4-Phenyl-1-(*o*-tolyl)-1*H*-imidazole (33b)

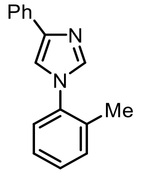

Following **GP*1**, but irradiating the reaction for 24 h and at λ = 310 nm, **33a** (23 mg, 0.1 mmol, 1.0 equiv.) gave **33b** (92%) as a yellow oil. R_f 0.28 [hexane:EtOAc (4:1)]; ^{1}H NMR (600 MHz, $CDCl_3$) δ 7.84 (2H, d, $J = 7.4$ Hz), 7.63 (1H, s), 7.41 (2H, t, $J = 7.7$ Hz), 7.38-7.34 (3H, m), 7.34-7.30 (1H, m), 7.30-7.26 (2H, m), 2.25 (3H, s); ^{13}C NMR (151 MHz, $CDCl_3$) δ 142.4, 137.9, 136.7, 134.0, 134.0, 131.5, 129.1, 128.8, 127.1, 127.1, 126.7, 125.0, 116.3, 17.9; HRMS (EI): found M^+ 234.1151, $C_{16}H_{14}N_2$ requires 234.1152.

Following **GP*1**, **33a** (23 mg, 0.1 mmol, 1.0 equiv.) gave **33b** (51%) as a yellow oil.

4-Phenyl-1-vinyl-1*H*-imidazole (34b)

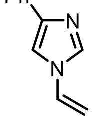

Following **GP*3**, **34a** (17 mg, 0.1 mmol, 1.0 equiv.) gave **34b** (36%) as a yellow oil. R_f 0.20 [hexane:EtOAc (3:1)]; ^{1}H NMR (600 MHz, $CDCl_3$) δ 7.80 (2H, d, $J = 7.1$ Hz), 7.68 (1H, s), 7.46 (1H, s), 7.39 (2H, t, $J = 7.8$ Hz), 7.27 (1H, t, $J = 6.0$ Hz), 6.90 (1H, dd, $J = 15.7$, 8.8 Hz), 5.33 (1H, d, $J = 15.8$ Hz), 4.92 (1H, d, $J = 8.8$ Hz); ^{13}C NMR (151 MHz, $CDCl_3$) δ 143.2, 136.5, 133.6, 129.4, 128.8, 127.4, 125.2, 111.1, 101.6; HRMS (ESI): found MH^+ 171.0916, $C_{11}H_{11}N_2$ requires 171.0917.

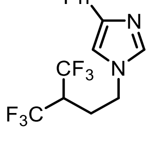

Following **GP*1**, **34a** (17 mg, 0.1 mmol, 1.0 equiv.) didn't give **34b** but a HFIP adding byproduct.

4-Phenyl-1-((triisopropylsilyl)ethynyl)-1*H*-imidazole (35b)

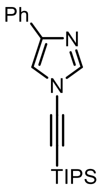

Following **GP*2**, but irradiating the reaction for 6 h, **35a** (33 mg, 0.1 mmol, 1.0 equiv.) gave **35b** (50%) as a colourless oil. ^{1}H NMR (600 MHz, $CDCl_3$) δ 7.79-7.76 (3H, m), 7.41-7.37 (3H, m), 7.29 (1H, t, *J* = 7.4 Hz), 1.15-1.12 (21H, m); ^{13}C NMR (151 MHz, $CDCl_3$) δ 141.9, 140.6, 132.9, 128.8, 127.7, 125.4, 117.1, 91.8, 70.4, 18.7, 11.4. Data in accordance with the literature.[42]

1-Benzyl-4-phenyl-1*H*-imidazole (37b)

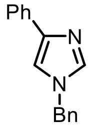

Following **GP*1**, but irradiating the reaction for 3 h, **37a** (23 mg, 0.1 mmol, 1.0 equiv.) gave **37b** (67%) as a yellow solid. ^{1}H NMR (400 MHz, $CDCl_3$) δ 7.76 (2H, dd, *J* = 8.2, 1.2 Hz), 7.58 (1H, s), 7.39-7.32 (5H, m), 7.25-7.16 (4H, m), 5.11 (2H, s); ^{13}C NMR (101 MHz, $CDCl_3$) δ 142.7, 137.7, 136.1, 134.2, 129.1, 128.7, 128.4, 127.4, 126.9, 124.9, 115.2, 51.1. Data in accordance with the literature.[43]

4-Phenyl-1*H*-imidazole (38b)

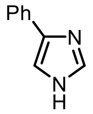

Following **GP*1**, but irradiating the reaction for 6 h, **38a** (14 mg, 0.1 mmol, 1.0 equiv.) gave **38b** (52%) as a white solid. ^{1}H NMR (600 MHz, $CDCl_3$) δ 7.72 (2H, d, *J* = 7.7 Hz), 7.67 (1H, s), 7.37 (2H, t, *J* = 7.6 Hz), 7.33 (1H, s), 7.25 (1H, t, *J* = 7.0 Hz), 6.91 (1H, br s); ^{13}C NMR (151 MHz, $CDCl_3$) δ 139.0, 135.6, 132.9, 128.9, 127.3, 125.1, 115.2. Data in accordance with the literature.[31]

Methyl 1-Methyl-1*H*-imidazole-2-carboxylate (39b)

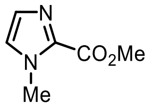

Following **GP*1**, but irradiating the reaction for 48 h, **39a** (14 mg, 0.1 mmol, 1.0 equiv.) gave **39b** (53%) as a yellow oil. ^{1}H NMR (600 MHz, $CDCl_3$) δ 7.14 (1H, s), 7.03 (1H, s), 4.01 (3H, s), 3.93 (3H, s); ^{13}C NMR (151 MHz, $CDCl_3$) δ 159.7, 136.6, 129.6, 126.4, 52.3, 35.9. Data in accordance with the literature.[44]

Methyl 1-Methyl-1*H*-imidazole-5-carboxylate (41b)

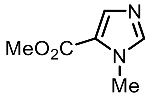

Following **GP*1**, **41a** (14 mg, 0.1 mmol, 1.0 equiv.) gave **41b** (70%) as a yellow oil. ^{1}H NMR (600 MHz, $CDCl_3$) δ 7.71 (1H, d, *J* = 1.1 Hz), 7.54 (1H, s), 3.90 (3H, s), 3.85 (3H, s); ^{13}C NMR (151 MHz, $CDCl_3$) δ 161.0, 142.6, 137.8, 123.1, 51.6, 34.2; HRMS (EI): found M^+ 140.0582, $C_6H_8N_2O_2$ requires 140.0580. Data in accordance with the literature.[45]

***N*,*N*-dimethyl-1-phenyl-1*H*-imidazole-4-carboxamide (42b)**

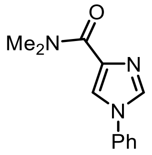

Following **GP*2**, but irradiating the reaction for 48 h, **42a** (22 mg, 0.1 mmol, 1.0 equiv.) gave **42b** (39%) as a yellow solid, m.p. 135-136 °C. R_f 0.72 [hexane:EtOAc (1:2)]; ^{1}H NMR (600 MHz, $CDCl_3$) δ 7.89 (1H, s), 7.78 (1H, s), 7.50 (2H, t, *J* = 7.7 Hz), 7.43-7.38 (3H, m), 3.52 (3H, s), 3.11 (3H, s); ^{13}C NMR (151 MHz, $CDCl_3$) δ 164.1, 139.5, 136.9, 134.3, 130.2, 128.2, 123.6, 121.7, 29.8; HRMS (ESI): found MNa^+ 238.0948, $C_{12}H_{13}N_3ONa$ requires 238.0951.

Following **GP*1**, but irradiating the reaction for 48 h, **42a** (22 mg, 0.1 mmol, 1.0 equiv.) gave **42b** (29%) as a yellow solid and 39% of **42a** was recovered.

1-Phenyl-1*H*-imidazole-4-carbonitrile (43b)

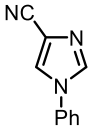

Following **GP*2**, but irradiating the reaction for 9 h, **43a** (17 mg, 0.1 mmol, 1.0 equiv.) gave **43b** (78%) as a colourless solid. ^{1}H NMR (600 MHz, $CDCl_3$) δ 7.85 (1H, d, *J* = 1.4 Hz), 7.81 (1H, d, *J* = 1.4 Hz), 7.54 (2H, t, *J* = 7.7 Hz), 7.47 (1H, t, *J* = 7.4 Hz), 7.41-7.37 (2H, m); ^{13}C NMR (151 MHz, $CDCl_3$) δ 137.2, 135.8, 130.4, 129.2, 126.8, 122.1, 115.7, 114.5. Data in accordance with the literature.[46]
Following **GP*1**, **43a** (17 mg, 0.1 mmol, 1.0 equiv.) gave **43b** (51%) as a colourless solid.

(1-Phenyl-1*H*-imidazol-4-yl)methanol (44b)

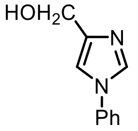

Following GP*2, but irradiating the reaction for 3 h, **44a** (16 mg, 0.1 mmol, 1.0 equiv.) gave **44b** (55%) as a yellow solid, m.p. 116-117 °C. Rf 0.12 [EtOAc] 1H NMR (600 MHz, CDCl3) δ 7.83 (1H, s), 7.48 (2H, t, *J* = 7.7 Hz), 7.40-7.34 (3H, m), 7.25 (1H, s), 4.69 (2H, s), 2.67 (1H, br s); ^{13}C NMR (151 MHz, $CDCl_3$) δ 143.6, 137.4, 135.6, 130.1, 127.7, 121.6, 115.7, 58.4; HRMS (ESI): found MNa^+ 197.0682, $C_{10}H_{10}N_2ONa$ requires 197.0685.
Following **GP*1**, but irradiating the reaction for 3 h, **44a** (8 mg, 0.1 mmol, 1.0 equiv.) gave **44b** (23%) as a yellow solid.

4-Fluoro-1-phenyl-1*H*-imidazole (45b)

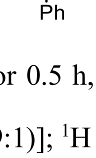

Following **GP*2**, but irradiating the reaction for 0.5 h, **45a** (16 mg, 0.1 mmol, 1.0 equiv.) gave **45b** (28%) as a yellow oil. R_f 0.11 [hexane:EtOAc (9:1)]; ^{1}H NMR (600 MHz, $CDCl_3$) δ 7.49 (2H, t, *J* = 7.8 Hz), 7.44 (1H, t, *J* = 1.6 Hz), 7.39 (1H, tt, *J* = 7.3, 1.2 Hz), 7.37 (2H, dd, *J* = 8.5, 1.2 Hz), 6.82 (1H, dd, *J* = 8.2, 1.8 Hz); ^{13}C NMR (151 MHz, $CDCl_3$) δ 158.1 (d, *J* = 238.6 Hz), 137.3, 130.1, 128.9 (d, *J* = 15.2

Hz), 128.1, 121.4, 97.3 (d, J = 38.9 Hz); ^{19}F NMR (565 MHz, $CDCl_3$) δ –134.2 (d, J = 8.0 Hz); HRMS (EI): found M^+ 162.0586, $C_9H_7N_2F$ requires 162.0588.

Following **GP*1**, but irradiating the reaction for 1 h, **45a** (16 mg, 0.1 mmol, 1.0 equiv.) gave **45b** (14%) as a yellow oil.

3,5-Dimethyl-1-(1-phenyl-1*H*-imidazol-4-yl)-1*H*-pyrazole (46b)

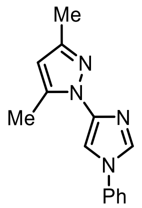

Following **GP*2**, but irradiating the reaction for 3 h, **46a** (24 mg, 0.1 mmol, 1.0 equiv.) gave **46b** (75%) as a brown solid, m.p. 79-80 °C. R_f 0.36 [hexane:EtOAc (1:1)]; ^{1}H NMR (600 MHz, $CDCl_3$) δ 7.74 (1H, s), 7.49 (2H, t, J = 7.5 Hz), 7.45-7.41 (3H, m), 7.39 (1H, t, J = 7.7 Hz), 5.95 (1H, s), 2.50 (3H, s), 2.28 (3H, s); ^{13}C NMR (151 MHz, $CDCl_3$) δ 149.4, 141.9, 140.7, 137.2, 132.7, 130.1, 127.9, 121.4, 109.2, 106.9, 13.6, 12.6; HRMS (ESI): found MNa^+ 261.1112, $C_{14}H_{14}N_4Na$ requires 261.1111.

Following **GP*1**, **46a** (24 mg, 0.1 mmol, 1.0 equiv.) gave **46b** (20%) as a brown solid.

1,2,4-Triphenyl-1*H*-imidazole (47b)

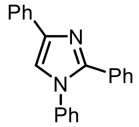

Following **GP*1**, but irradiating the reaction for 60 h, **47a** (30 mg, 0.1 mmol, 1.0 equiv.) gave **47b** (80%) as a yellow oil. ^{1}H NMR (600 MHz, $CDCl_3$) δ 7.91 (2H, d, J = 6.9 Hz), 7.49-7.44 (3H, m), 7.43-7.39 (5H, m), 7.30-7.27 (6H, m); ^{13}C NMR (151 MHz, $CDCl_3$) δ 147.1, 141.9, 138.7, 134.0, 130.5, 129.6, 129.0, 128.7, 128.6, 128.3, 128.3, 127.1, 126.0, 125.2, 118.7. Data in accordance with the literature.[47]

1,4,5-Triphenyl-1H-imidazole (48b)

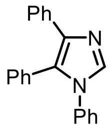

Following **GP*2**, but irradiating the reaction for 36 h, **48a** (30 mg, 0.1 mmol, 1.0 equiv.) gave **48b** (45%) as a pale-yellow solid. ^{1}H NMR (600 MHz, $CDCl_3$) δ 7.79 (1H, s), 7.56 (2H, d, J = 7.2 Hz), 7.34-7.31 (3H, m), 7.30-7.24 (5H, m), 7.20 (1H, t, J = 7.3 Hz), 7.18-7.15 (2H, m), 7.13-7.10 (2H, m); ^{13}C NMR (151 MHz, $CDCl_3$) δ 139.1, 137.5, 136.6, 134.6, 130.9, 130.3, 129.3, 128.8, 128.7, 128.3, 128.2, 128.1, 127.4, 126.8, 125.9. Data in accordance with the literature.[48]

Following **GP*1**, **48a** (30 mg, 0.1 mmol, 1.0 equiv.) gave **48b** (25%) as a pale-yellow solid.

5-Methyl-1,4-diphenyl-1H-imidazole (49b)

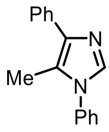

Following **GP*1**, but irradiating the reaction for 3 h, **49a** (23 mg, 0.1 mmol, 1.0 equiv.) gave **49b** (77%) as a colourless solid, m.p. 137-138 °C. ^{1}H NMR (600 MHz, $CDCl_3$) δ 7.74 (2H, d, J = 7.9 Hz), 7.65 (1H, s), 7.52 (2H, t, J = 7.7 Hz), 7.48-7.41 (3H, m), 7.36-7.33 (2H, m), 7.28 (1H, td, J = 7.4, 1.3 Hz), 2.36 (3H, s); ^{13}C NMR (151 MHz, $CDCl_3$) δ 138.6, 136.6, 136.4, 135.3, 129.7, 128.6, 128.5, 127.1, 126.5, 126.1, 124.1, 10.9; HRMS (ESI): found MH^+ 235.1228, $C_{16}H_{15}N_2$ requires 235.1230.

2-Methyl-1,4-diphenyl-1H-imidazole (50b)

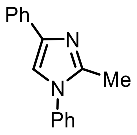

Following **GP*1**, **50a** (23 mg, 0.1 mmol, 1.0 equiv.) gave **50b** (50%) as a yellow solid. ^{1}H NMR (600 MHz, $CDCl_3$) δ 7.79 (2H, dd, J = 8.2, 1.3 Hz), 7.51 (2H, t, J = 7.6 Hz), 7.44 (1H, tt, J = 6.8, 1.3 Hz), 7.40-7.34 (4H, m), 7.30 (1H, s), 7.24 (1H, tt, J = 7.1, 1.3 Hz), 2.43 (3H, s); ^{13}C NMR (151 MHz, $CDCl_3$) δ 145.2, 140.7, 138.1, 134.1, 129.7, 128.7, 128.4, 126.9, 125.7, 124.9, 116.5, 14.0; HRMS (ESI): found MH^+ 235.1228, $C_{16}H_{15}N_2$ requires 235.1230. Data in accordance with the literature.[49]

2,5-Dimethyl-1-phenyl-1*H*-imidazole (51b)

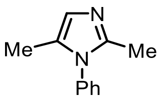

Following **GP*1**, **51a** (17 mg, 0.1 mmol, 1.0 equiv.) gave **51b** (81%) as a pale-yellow solid. ^{1}H NMR (600 MHz, $CDCl_3$) δ 7.50 (2H, t, J = 7.4 Hz), 7.49-7.44 (1H, m), 7.21-7.17 (2H, m), 6.75 (1H, s), 2.20 (3H, s), 1.99 (3H, s); ^{13}C NMR (151 MHz, $CDCl_3$) δ 144.7, 136.8, 129.7, 128.8, 128.4, 127.6, 124.6, 14.0, 10.3. Data in accordance with the literature.[49]

Methyl 5-Methyl-1-phenyl-1*H*-imidazole-2-carboxylate (52b)

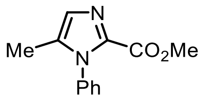

Following **GP*3**, but irradiating the reaction for 48 h, **52a** (22 mg, 0.1 mmol, 1.0 equiv.) gave **52b** (36%) as a yellow solid, m.p. 151-152 °C. R_f 0.17 [hexane:EtOAc (1:1)]; ^{1}H NMR (600 MHz, $CDCl_3$) δ 7.54-7.47 (3H, m), 7.24-7.17 (2H, m), 7.04 (1H, s), 3.78 (3H, s), 2.03 (3H, s); ^{13}C NMR (151 MHz, $CDCl_3$) δ 158.9, 137.0, 136.9, 134.7, 129.4, 129.3, 128.3, 127.2, 52.2, 10.2; HRMS (ESI): found MNa^+ 239.0791, $C_{12}H_{12}N_2O_2Na$ requires 239.0791.

Following **GP*1**, **52a** (22 mg, 0.1 mmol, 1.0 equiv.) gave **52b** (6%) as a yellow solid and 87% of **52a** was recovered.

Methyl 5-Methyl-1-phenyl-1*H*-imidazole-4-carboxylate (53b)

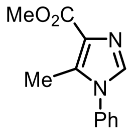

Following **GP*2**, **53a** (22 mg, 0.1 mmol, 1.0 equiv.) gave **53b** (63%) as a pale-yellow solid. ^{1}H NMR (600 MHz, $CDCl_3$) δ 7.55 (1H, s), 7.54-7.47 (3H, m), 7.30-7.26 (2H, m), 3.91 (3H, s), 2.46 (3H, s); ^{13}C NMR (151 MHz, $CDCl_3$) δ 164.3, 136.7, 136.4, 135.4, 129.9, 129.7, 129.4, 126.1, 51.6, 10.6. Data in accordance with the literature.[50]

5-Amino-1-phenyl-1*H*-imidazole-4-carbonitrile (54b)

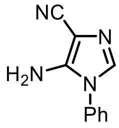

Following **GP*1**, but irradiating the reaction for 6 h, **54a** (18 mg, 0.1 mmol, 1.0 equiv.) gave **54b** (28%) as a pale-yellow solid and 42% of **54a** was recovered. ^{1}H NMR (600 MHz, DMSO-d$_6$) δ 7.56 (2H, t, *J* = 7.5 Hz), 7.50 (1H, tt, *J* = 6.6, 1.2 Hz), 7.47-7.44 (2H, m), 7.41 (1H, s), 6.14 (2H, s); ^{13}C NMR (151 MHz, DMSO-d$_6$) δ 147.7, 134.3, 133.1, 130.3, 129.2, 125.7, 117.5, 91.5; HRMS (ESI): found MNa$^+$ 207.0639, $C_{10}H_8N_4Na$ requires 207.0641. Data in accordance with the literature.[51]

2-Methyl-4-phenyl-1*H*-imidazole (55b) and 5-Methyl-4-phenyl-1*H*-imidazole (55c)

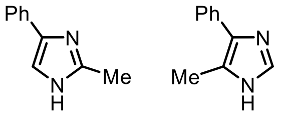

Following **GP*1**, **55a** (16 mg, 0.1 mmol, 1.0 equiv.) gave **55b** (20%) and **55c** (16%) as an inseparable mixture, as a brown oil. **55b**: **55c** = 1:0.8. R_f 0.12 [EtOAc].

Signals corresponding to **55b**: ^{1}H NMR (600 MHz, DMSO-d$_6$) δ 7.69 (2H, d, *J* = 7.5 Hz), 7.38 (1H, s), 7.31 (2H, t, *J* = 7.7 Hz), 7.15 (1H, t, *J* = 7.4 Hz), 2.31 (3H, s); ^{13}C NMR (151 MHz, DMSO-d$_6$) δ 144.6, 128.5, 125.9, 124.0, 40.1**.**

Signals corresponding to **55c**: ^{1}H NMR (600 MHz, DMSO-d$_6$) δ 11.99 (0.8H, s), 7.60 (1.6H, d, *J* = 7.7 Hz), 7.56 (0.8H, s), 7.38 (1.6H, t, *J* = 7.7 Hz), 7.20 (0.8H, t, *J* = 7.4 Hz), 2.36 (2.4H, s); ^{13}C NMR (151 MHz, DMSO-d$_6$) δ 133.9, 128.5, 125.9, 125.7, 13.9.

HRMS (ESI): found MH$^+$ 159.0919, $C_{10}H_{11}N_2$ requires 159.0917.

4-Methyl-2-phenyl-1*H*-imidazole (56b & 57b)

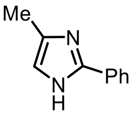

Start from **3-Phenyl-4-methyl-1*H*-pyrazole** (**56b**)

Following **GP*4**, **56a** (16 mg, 0.1 mmol, 1.0 equiv.) gave **56b** (34%) as a white solid, m.p. 167-168 °C, and 29% of **56a** was recovered. R_f 0.45 [EtOAc] ^{1}H NMR (600 MHz, $CDCl_3$) δ 7.85-7.81 (2H, m), 7.35-7.31 (2H, m), 7.28 (1H, tt, *J* = 6.5, 1.3 Hz), 6.82 (1H, t, *J* = 1.1 Hz), 2.26 (3H, d, *J* = 1.0 Hz); ^{13}C NMR

(151 MHz, $CDCl_3$) δ 146.3, 130.6, 128.9, 128.4, 125.2, 12.1; HRMS (ESI): found MH^+ 159.0919, $C_{10}H_{11}N_2$ requires 159.0917.

Following **GP*1**, but irradiating the reaction for 6 h, **56a** (16 mg, 0.1 mmol, 1.0 equiv.) gave **56b** (31%) as a white solid.

Start from **3-Methyl-5-phenyl-1*H*-pyrazole** (**57b**)

Following **GP*1**, but irradiating the reaction for 6 h, **57a** (16 mg, 0.1 mmol, 1.0 equiv.) gave **57b** (56%) as a white solid.

Methyl 4-Methyl-1*H*-imidazole-2-carboxylate (58b)

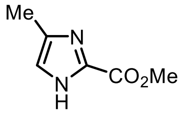

Following **GP*1**, but irradiating the reaction for 24 h, **58a** (14 mg, 0.1 mmol, 1.0 equiv.) gave **58b** (20%) as a pale-yellow solid, m.p. 127-128 °C. R_f 0.31 [hexane:EtOAc (1:1)]; ^1H NMR (600 MHz, $CDCl_3$) δ 10.54 (1H, br s), 6.96 (1H, s), 3.94 (3H, s), 2.32 (3H, s); ^{13}C NMR (151 MHz, $CDCl_3$) δ 159.7, 136.7, 52.6; HRMS (ESI): found MNa^+ 163.0478, $C_6H_8N_2O_2Na$ requires 163.0478.

2,4,5-Trimethyl-1-phenyl-1*H*-imidazole (61b)

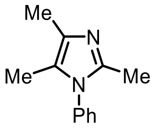

Following **GP*1**, but irradiating the reaction for 3 h, **61a** (19 mg, 0.1 mmol, 1.0 equiv.) gave **61b** (58%) as a yellow oil. ^1H NMR (600 MHz, $CDCl_3$) δ 7.46 (2H, t, J = 7.3 Hz), 7.41 (1H, t, J = 7.3 Hz), 7.14 (2H, d, J = 7.2 Hz), 2.15 (6H, s), 1.89 (3H, s); ^{13}C NMR (151 MHz, $CDCl_3$) δ 142.9, 137.3, 131.7, 129.5, 128.5, 127.6, 123.1, 13.8, 12.6, 9.4. Data in accordance with the literature.[52]

1-Phenyl-4,5,6,7-tetrahydro-1*H*-benzo[*d*]imidazole (62b)

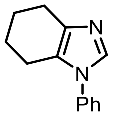

Following **GP*1**, but irradiating the reaction for 6 h, **62a** (20 mg, 0.1 mmol, 1.0 equiv.) gave **62b** (97%) as a brown oil. R_f 0.11 [hexane:EtOAc (1:1)]; ^1H NMR (600 MHz, $CDCl_3$) δ 7.54 (1H, s), 7.46 (2H, t, J

= 7.7 Hz), 7.37 (1H, t, *J* = 7.4 Hz), 7.28 (2H, d, *J* = 7.4 Hz), 2.67 (2H, t, *J* = 5.9 Hz), 2.53 (2H, t, *J* = 5.9 Hz), 1.87-1.77 (4H, m); ^{13}C NMR (151 MHz, $CDCl_3$) δ 137.8, 136.9, 135.1, 129.6, 127.6, 126.0, 124.3, 24.5, 23.3, 23.3, 22.0; HRMS (ESI): found MH^+ 199.1227, $C_{13}H_{15}N_2$ requires 199.1230.

1-Phenyl-1,4,5,6-tetrahydrocyclopenta[*d*]imidazole (63b)

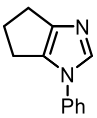

Following **GP*1**, but irradiating the reaction for 3 h, **63a** (18 mg, 0.1 mmol, 1.0 equiv.) gave **63b** (53%) as a brown solid, m.p. 83-84 °C. R_f 0.13 [hexane:EtOAc (1:1)]; ^{1}H NMR (600 MHz, $CDCl_3$) δ 7.68 (1H, s), 7.44 (2H, t, *J* = 7.9 Hz), 7.35 (2H, d, *J* = 7.7 Hz), 7.30 (1H, t, *J* = 7.3 Hz), 2.84 (2H, t, *J* = 7.2 Hz), 2.72 (2H, t, *J* = 6.9 Hz), 2.53 (2H, p, *J* = 7.4 Hz); ^{13}C NMR (151 MHz, $CDCl_3$) δ 148.8, 138.1, 137.7, 133.4, 129.9, 126.8, 120.9, 28.2, 24.8, 24.3; HRMS (ESI): found MH^+ 185.1072, $C_{12}H_{13}N_2$ requires 185.1073.

***tert*-Butyl 1-Phenyl-1,4,6,7-tetrahydro-5*H*-imidazo[4,5-*c*]pyridine-5-carboxylate (64b)**

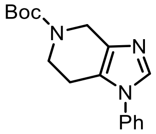

Following **GP*1**, but irradiating the reaction for 6 h, **64a** (30 mg, 0.1 mmol, 1.0 equiv.) gave **64b** (73%) as a yellow oil. R_f 0.12 [hexane:EtOAc (1:1)]; ^{1}H NMR (600 MHz, $CDCl_3$) δ 7.62 (1H, s), 7.49 (2H, t, *J* = 7.4 Hz), 7.40 (1H, t, *J* = 7.5 Hz), 7.29 (2H, d, *J* = 7.7 Hz), 4.54 (2H, s), 3.73 (2H, s), 2.66 (2H, s), 1.49 (9H, s); ^{13}C NMR (151 MHz, $CDCl_3$) δ 155.1, 136.2, 135.7, 135.1, 129.7, 127.9, 124.4, 123.8, 79.9, 43.7, 40.7, 28.4, 22.1; HRMS (ESI): found MNa^+ 322.1534, $C_{17}H_{21}N_3O_2Na$ requires 322.1526.

***tert*-Butyl 1-Phenyl-4,6-dihydropyrrolo[3,4-*d*]imidazole-5(1*H*)-carboxylate (65b)**

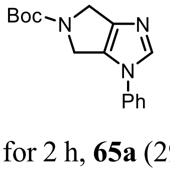

Following **GP*2**, but irradiating the reaction for 2 h, **65a** (29 mg, 0.1 mmol, 1.0 equiv.) gave **65b** (60%) as a brown solid, m.p. 126-127 °C. R_f 0.23 [hexane:EtOAc (1:1)]; ^{1}H NMR (600 MHz, $CDCl_3$, rotamers) δ 7.74 (1H, d, *J* = 9.4 Hz), 7.50-7.42 (2H, m), 7.37-7.32 (1H, m), 7.30 (2H, dd, *J* = 8.6, 1.2 Hz), 4.61

(2H, dt, J = 28.6, 2.8 Hz), 4.49-4.41 (2H, m), 1.49 (9H, d, J = 4.0 Hz); ^{13}C NMR (151 MHz, $CDCl_3$, rotamers) δ 154.8, 154.4, 142.8, 142.7, 138.6, 138.4, 136.7, 136.7, 130.2, 127.7, 127.6, 127.5, 127.5, 120.8, 120.4, 80.1, 80.1, 47.1, 46.7, 46.1, 45.9, 28.6, 28.6; HRMS (ESI): found MNa^+ 308.1372, $C_{16}H_{19}N_3O_2Na$ requires 308.1370.

Following **GP*1**, but irradiating the reaction for 6 h, **65a** (29 mg, 0.1 mmol, 1.0 equiv.) gave **65b** (14%) as a brown solid.

1-Phenyl-1,4,6,7-tetrahydropyrano[3,4-*d*]imidazole (66b)

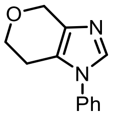

Following **GP*4**, but irradiating the reaction for 3 h, **66a** (20 mg, 0.1 mmol, 1.0 equiv.) gave **66b** (66%) as a yellow oil. R_f 0.29 [hexane:EtOAc (3:1)]; ^1H NMR (600 MHz, $CDCl_3$) δ 7.58 (1H, s), 7.46 (2H, t, J = 7.8 Hz), 7.38 (1H, t, J = 7.5 Hz), 7.30-7.26 (2H, m), 4.72 (2H, s), 3.93 (2H, t, J = 5.0 Hz), 2.69 (2H, t, J = 5.4 Hz); ^{13}C NMR (151 MHz, $CDCl_3$) δ 136.2, 136.0, 135.5, 129.8, 127.9, 123.8, 123.3, 65.8, 64.8, 23.3; HRMS (ESI): found MH^+ 201.1021, $C_{12}H_{13}N_2O$ requires 201.1022.

Following **GP*1**, **66a** (20 mg, 0.1 mmol, 1.0 equiv.) gave **66b** (38%) as a yellow solid.

1-Phenyl-4,6-dihydro-1*H*-furo[3,4-*d*]imidazole (67b)

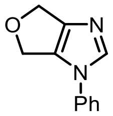

Following **GP*2**, but irradiating the reaction for 1 h, **67a** (19 mg, 0.1 mmol, 1.0 equiv.) gave **67b** (50%) as a brown solid, m.p. 77-78 °C. R_f 0.16 [hexane:EtOAc (1:1)]; ^1H NMR (600 MHz, $CDCl_3$) δ 7.79 (1H, s), 7.47 (2H, t, J = 7.8 Hz), 7.34 (1H, t, J = 7.5 Hz), 7.27 (2H, d, J = 7.9 Hz), 5.07 (2H, t, J = 3.5 Hz), 4.91 (2H, t, J = 3.5 Hz); ^{13}C NMR (151 MHz, $CDCl_3$) δ 146.8, 139.3, 136.8, 130.9, 130.2, 127.4, 120.2, 67.5, 66.1; HRMS (ESI): found MH^+ 187.0866, $C_{11}H_{11}N_2O$ requires 187.0866.

Following **GP*1**, but irradiating the reaction for 6 h, **67a** (19 mg, 0.1 mmol, 1.0 equiv.) gave **67b** (10%) as a brown solid.

Ethyl 1-Phenyl-1,4,6,7-tetrahydropyrano[3,4-*d*]imidazole-2-carboxylate (68b)

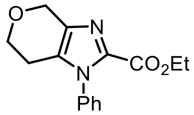

Following **GP*2**, but irradiating the reaction for 48 h, **68a** (27 mg, 0.1 mmol, 1.0 equiv.) gave **68b** (43%) as a yellow solid, m.p. 96-97 °C, and 28% of **68a** was recovered. R_f 0.39 [EtOAc] ^{1}H NMR (600 MHz, $CDCl_3$) δ 7.50-7.45 (3H, m), 7.25-7.21 (2H, m), 4.74 (2H, s), 4.25 (2H, q, *J* = 7.1 Hz), 3.93 (2H, t, *J* = 5.5 Hz), 2.47 (2H, t, *J* = 5.3 Hz), 1.26 (3H, t, *J* = 7.1 Hz); ^{13}C NMR (151 MHz, $CDCl_3$) δ 158.4, 136.6, 136.5, 136.0, 130.5, 129.3, 129.2, 126.7, 65.6, 64.6, 61.4, 22.9, 14.2; HRMS (ESI): found MNa^+ 295.1048, $C_{15}H_{16}N_2O_3Na$ requires 295.1053.

Following **GP*1**, but irradiating the reaction for 48 h, **68a** (27 mg, 0.1 mmol, 1.0 equiv.) gave **68b** (22%) as a yellow solid and 58% of **68a** was recovered.

(1*S*,3a*S*,3b*R*,10a*S*,10b*S*,12a*S*)-1,10a,12a-Trimethyl-1,2,3,3a,3b,4,5,5a,6,7,10,10a,10b,11,12,12a-hexadecahydrocyclopenta[7,8]phenanthro[2,3-*d*]imidazol-1-ol (69b)

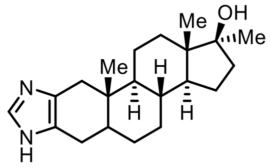

Following **GP*1**, but irradiating the reaction for 48 h, **69a** (33 mg, 0.1 mmol, 1.0 equiv.) gave **69b** (40%) as a pale-yellow solid. R_f 0.11 [CH_2Cl_2:MeOH (10:1)]; ^{1}H NMR (600 MHz, $CDCl_3$) δ 7.47 (1H, s), 2.59 (1H, d, *J* = 14.9 Hz), 2.50 (1H, dd, *J* = 14.8, 4.4 Hz), 2.27-2.18 (2H, m), 1.82 (1H, ddd, *J* = 15.1, 11.8, 3.6 Hz), 1.77-1.69 (2H, m), 1.66-1.51 (5H, m), 1.49-1.17 (7H, m), 1.22 (3H, s), 0.94-0.81 (3H, m), 0.88 (3H, s), 0.79 (3H, s); ^{13}C NMR (151 MHz, $CDCl_3$) δ 133.2, 81.9, 54.0, 50.7, 45.6, 43.1, 39.1, 37.3, 37.2, 36.7, 31.8, 31.7, 29.2, 27.5, 26.0, 23.5, 21.0, 14.1, 12.0; HRMS (ESI): found MH^+ 329.2586, $C_{21}H_{33}N_2O$ requires 329.2587. This compound was known in the literature,[53] but spectroscopic data was not provided.

1-Phenyl-6,7-dihydro-5*H*-pyrrolo[1,2-*c*]imidazole (70b)

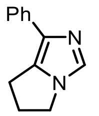

Following **GP*3**, but irradiating the reaction for 4 h, **70a** (18 mg, 0.1 mmol, 1.0 equiv.) gave **70b** (62%) as a white solid, m.p. 101-102 °C. R_f 0.15 [EtOAc] ^{1}H NMR (600 MHz, $CDCl_3$) δ 7.72 (2H, d, *J* = 8.1 Hz), 7.45 (1H, d, *J* = 2.4 Hz), 7.36 (2H, tt, *J* = 7.9, 1.4 Hz), 7.18 (1H, tt, *J* = 7.3, 1.3 Hz), 3.98 (2H, td, *J* = 7.4, 3.8 Hz), 3.02 (2H, td, *J* = 7.0, 3.2 Hz), 2.67 (2H, pd, *J* = 7.3, 3.3 Hz); ^{13}C NMR (151 MHz, $CDCl_3$) δ 135.1, 133.6, 131.6, 130.3, 128.6, 125.8, 124.6, 44.1, 29.5, 23.4; HRMS (ESI): found MH^+ 185.1074, $C_{12}H_{13}N_2$ requires 185.1073.

Following **GP*1**, but irradiating the reaction for 3 h, **70a** (18 mg, 0.1 mmol, 1.0 equiv.) gave **70b** (20%) as a white solid.

3-Phenyl-6,7-dihydro-5*H*-pyrrolo[1,2-*c*]imidazole (71b)

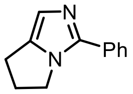

Following **GP*3**, but irradiating the reaction for 48 h, **71a** (18 mg, 0.1 mmol, 1.0 equiv.) gave **71b** (27%) as a pale-yellow solid. R_f 0.20 [EtOAc] ^{1}H NMR (600 MHz, $CDCl_3$) δ 7.74 (2H, d, *J* = 8.2 Hz), 7.35 (2H, t, *J* = 7.7 Hz), 7.21 (1H, t, *J* = 7.3 Hz), 7.17 (1H, s), 4.00 (2H, t, *J* = 7.1 Hz), 2.92 (2H, t, *J* = 7.6 Hz), 2.61 (2H, p, *J* = 7.4 Hz); ^{13}C NMR (101 MHz, $CDCl_3$) δ 155.0, 146.4, 135.0, 128.6, 126.6, 124.7, 110.4, 44.9, 26.1, 23.2; HRMS (ESI): found MH^+ 185.1073, $C_{12}H_{13}N_2$ requires 185.1073. Data in accordance with the literature.[54]

Imidazo[1,5-*a*]pyridine (72b)

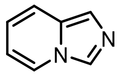

Following **GP*2**, **72a** (12 mg, 0.1 mmol, 1.0 equiv.) gave **72b** (45%) as a pale-yellow solid. ^{1}H NMR (600 MHz, $CDCl_3$) δ 8.08 (1H, s), 7.89 (1H, dd, *J* = 7.1, 1.2 Hz), 7.44-7.37 (2H, m), 6.67 (1H, ddd, *J* = 9.2, 6.4, 1.0 Hz), 6.52 (1H, ddd, *J* = 6.8, 6.5, 1.2 Hz); ^{13}C NMR (151 MHz, $CDCl_3$) δ 130.4, 127.7, 122.2, 120.0, 119.0, 118.5, 112.7. Data in accordance with the literature.[55]

3-Methylimidazo[1,5-*a*]pyridine (73b)

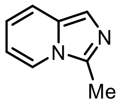

Following **GP*4**, **73a** (13 mg, 0.1 mmol, 1.0 equiv.) gave **73b** (62%) as a yellow oil. ^{1}H NMR (600 MHz, $CDCl_3$) δ 7.66 (1H, dd, *J* = 7.3, 1.2 Hz), 7.40 (1H, d, *J* = 9.1 Hz), 7.33 (1H, s), 6.64 (1H, ddd, *J* = 9.2, 6.3, 1.0 Hz), 6.54 (1H, ddd, *J* = 7.3, 6.3, 1.2 Hz), 2.64 (3H, s); ^{13}C NMR (151 MHz, $CDCl_3$) δ 135.1, 130.5, 120.7, 118.7, 118.4, 117.6, 112.3, 12.7. Data in accordance with the literature.[56]

Methyl Imidazo[1,5-*a*]pyridine-1-carboxylate (74b)

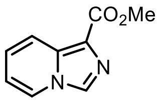

Following **GP*2**, **74a** (18 mg, 0.1 mmol, 1.0 equiv.) gave **74b** (64%) as a white solid, m.p. 143-144 °C. ^{1}H NMR (600 MHz, $CDCl_3$) δ 8.16 (1H, d, *J* = 9.2 Hz), 8.09 (1H, s), 8.05 (1H, d, *J* = 6.9 Hz), 7.11 (1H, ddd, *J* = 9.2, 6.5, 1.0 Hz), 6.79 (1H, td, *J* = 6.7, 1.1 Hz), 3.97 (3H, s); ^{13}C NMR (151 MHz, $CDCl_3$) δ 163.8, 134.5, 128.4, 124.6, 123.2, 121.6, 119.8, 114.5, 51.6; HRMS (ESI): found MNa^+ 199.0475, $C_9H_8N_2O_2Na$ required 199.0478.

Following **GP*1**, **74a** (18 mg, 0.1 mmol, 1.0 equiv.) gave **74b** (47%) as a white solid.

Methyl Imidazo[1,5-*a*]pyridine-3-carboxylate (75b)

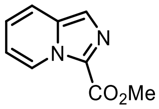

Following **GP*1**, but concentration of 0.020 M, **75a** (18 mg, 0.1 mmol, 1.0 equiv.) gave **75b** (85%) as a white solid, m.p. 70-71 °C. R_f 0.30 [hexane:EtOAc (1:1)]; ^{1}H NMR (600 MHz, $CDCl_3$) δ 9.31 (1H, dd, *J* = 7.2, 1.1 Hz), 7.62 (2H, d, *J* = 1.0 Hz), 7.06 (1H, ddd, *J* = 9.0, 6.6, 1.0 Hz), 6.91 (1H, ddd, *J* = 6.9, 6.8, 1.2 Hz), 4.01 (3H, s); ^{13}C NMR (151 MHz, $CDCl_3$) δ 160.1, 134.4, 127.3, 125.6, 122.9, 122.7, 118.3, 115.6, 52.2; HRMS (EI): found M^+ 176.0580, $C_9H_8N_2O_2$ requires 176.0588.

Imidazo[1,5-*a*]pyrimidine (76b)

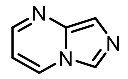

Following **GP*2**, **76a** (12 mg, 0.1 mmol, 1.0 equiv.) gave **76b** (25%) as a yellow solid and 24% of **76a** was recovered. ^{1}H NMR (600 MHz, $CDCl_3$) δ 8.21-8.15 (2H, m), 8.05 (1H, s), 7.65 (1H, s), 6.58 (1H, dd, J = 7.1, 3.7 Hz); ^{13}C NMR (151 MHz, $CDCl_3$) δ 146.2, 138.5, 129.2, 125.3, 119.9, 108.6. Data in accordance with the literature.[58]

Ethyl Imidazo[1,5-*a*]pyrimidine-8-carboxylate (77b)

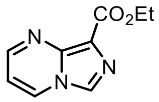

Following **GP*1**, but concentration of 0.020 M and irradiating the reaction for 48 h, **77a** (19 mg, 0.1 mmol, 1.0 equiv.) gave **77b** (34%) as a pale-yellow solid, m.p. 128-129 °C, and 55% of **77a** was recovered. R_f 0.05 [EtOAc]; ^{1}H NMR (600 MHz, $CDCl_3$) δ 8.58 (1H, dd, J = 3.8, 1.8 Hz), 8.33 (1H, dd, J = 7.1, 1.8 Hz), 8.08 (1H, s), 6.83 (1H, dd, J = 7.1, 3.8 Hz), 4.52 (2H, q, J = 7.2 Hz), 1.46 (3H, t, J = 7.1 Hz); ^{13}C NMR (151 MHz, $CDCl_3$) δ 162.4, 151.0, 140.3, 130.4, 125.5, 121.1, 110.0, 60.9, 14.7; HRMS (EI): found M^+ 191.0689, $C_9H_9N_3O_2$ requires 191.0689.

Imidazo[1,5-*a*]pyrimidine-8-carbonitrile (78b)

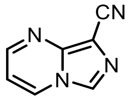

Following **GP*2**, **78a** (14 mg, 0.1 mmol, 1.0 equiv.) gave **78b** (52%) as a pale-yellow solid, m.p. 210-211 °C, and 24% of **78a** was recovered. R_f 0.10 [EtOAc]; ^{1}H NMR (600 MHz, CD_3OD) δ 8.81 (1H, dd, J = 7.1, 1.7 Hz), 8.57 (1H, dd, J = 3.9, 1.7 Hz), 8.37 (1H, s), 7.04 (1H, dd, J = 7.1, 3.9 Hz); ^{13}C NMR (151 MHz, CD_3OD) δ 153.8, 144.9, 133.9, 129.5, 115.2, 111.9, 102.4; HRMS (EI): found M^+ 144.0431, $C_7H_4N_4$ requires 144.0431.

Following **GP*1**, **78a** (14 mg, 0.1 mmol, 1.0 equiv.) gave **78b** (37%) as a pale-yellow solid and 50% of **78a** was recovered.

2,4-Dichloro-8-ethylimidazo[1,5-*a*]pyrimidine (79b)

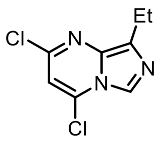

Following **GP*1**, but irradiating the reaction for 30 h, **79a** (22 mg, 0.1 mmol, 1.0 equiv.) gave **79b** (36%) as a brown solid, m.p. 64-65 °C, and 50% of **79a** was recovered. R_f 0.20 [EtOAc]; ^{1}H NMR (600 MHz, $CDCl_3$) δ 8.13 (1H, s), 6.64 (1H, s), 2.97 (2H, q, J = 7.6 Hz), 1.35 (3H, t, J = 7.6 Hz); ^{13}C NMR (151 MHz, $CDCl_3$) δ 143.1, 135.6, 134.1, 133.7, 122.9, 108.4, 20.0, 14.0; HRMS (ESI): found M$^+$ 215.0012, $C_8H_7Cl_2N_3$ requires 215.0012.

Methyl Imidazo[1,5-*a*]pyrazine-1-carboxylate (80b)

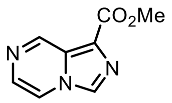

Following **GP*1**, **80a** (18 mg, 0.1 mmol, 1.0 equiv.) gave **80b** (62%) as a white solid, m.p. 205-206 °C. R_f 0.05 [EtOAc]; ^{1}H NMR (600 MHz, $CDCl_3$) δ 9.60 (1H, d, J = 1.7 Hz), 8.22 (1H, s), 7.95 (1H, dd, J = 4.9, 1.7 Hz), 7.81 (1H, d, J = 4.9 Hz), 4.02 (3H, s); ^{13}C NMR (151 MHz, $CDCl_3$) δ 162.7, 147.3, 130.5, 129.2, 128.1, 125.6, 115.6, 52.3; HRMS (EI): found M$^+$ 177.0534, $C_8H_7N_3O_2$ requires 177.0533.

Methyl Imidazo[1,5-*b*]pyridazine-5-carboxylate (81b)

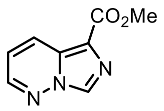

Following **GP*4**, but concentration of 0.020 M and irradiating the reaction for 48 h, **81a** (18 mg, 0.1 mmol, 1.0 equiv.) gave **81b** (26%) as a pale-yellow solid, m.p. 134-135 °C, and 63% of **81a** was recovered. R_f 0.30 [EtOAc]; ^{1}H NMR (600 MHz, $CDCl_3$) δ 8.49 (1H, ddd, J = 9.3, 1.7, 0.7 Hz), 8.44 (1H, d, J = 0.6 Hz), 8.26 (1H, dd, J = 4.3, 1.7 Hz), 6.93 (1H, dd, J = 9.3, 4.3 Hz), 4.00 (3H, s); ^{13}C NMR (151 MHz, $CDCl_3$) δ 163.2, 145.5, 132.0, 128.7, 128.3, 122.0, 116.7, 52.0; HRMS (EI): found M$^+$ 177.0533, $C_8H_7N_3O_2$ requires 177.0533.

Following **GP*1**, **81a** (18 mg, 0.1 mmol, 1.0 equiv.) gave **81b** (8%) as a pale-yellow solid and 57% of **81a** was recovered.

N-(3-(8-Cyanoimidazo[1,5-a]pyrimidin-4-yl)phenyl)-N-ethylacetamide (82b)

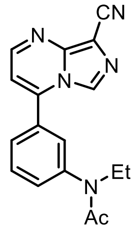

Following **GP*2**, but concentration of 0.020 M and irradiating the reaction for 48 h, **82a** (31 mg, 0.1 mmol, 1.0 equiv.) gave **82b** (23%) as a brown solid and 23% of **82a** was recovered. R_f 0.05 [CH_2Cl_2:MeOH (10:1)]; ^{1}H NMR (600 MHz, $CDCl_3$) δ 8.43 (1H, d, J = 7.4 Hz), 8.16 (1H, d, J = 7.8 Hz), 8.10 (1H, s), 8.00 (1H, s), 7.62 (1H, t, J = 7.8 Hz), 7.40-7.33 (2H, m), 3.83 (2H, q, J = 7.1 Hz), 1.89 (3H, s), 1.16 (3H, t, J = 7.4 Hz); ^{13}C NMR (151 MHz, $CDCl_3$) δ 169.8, 156.3, 144.2, 143.4, 137.5, 131.7, 131.0, 130.7, 127.4, 127.1, 126.5, 119.7, 114.4, 108.2, 44.2, 23.1, 13.3; HRMS (ESI): found MNa^+ 328.1170, $C_{17}H_{15}ON_5Na$ requires 328.1169. This compound was known in the literature,[58] but spectroscopic data was not provided.

Following **GP*1**, **82a** (31 mg, 0.1 mmol, 1.0 equiv.) didn't give **82b** and 68% of **82a** was recovered.

6-(2-Hydroxy-2-methylpropoxy)-8-(6-(6-((6-methoxypyridin-3-yl)methyl)-3,6-diazabicyclo[3.1.1]heptan-3-yl)pyridin-3-yl)imidazo[1,5-a]pyridine-1-carbonitrile (83b)

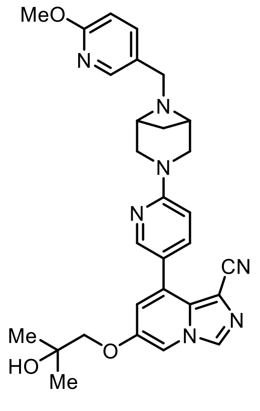

Following **GP*1**, but concentration of 0.020 M, **83a** (53 mg, 0.1 mmol, 1.0 equiv.) gave **83b** (12%) as a brown solid, m.p. 106-107 °C, and 54% of **83a** was recovered. R_f 0.45 [CH_2Cl_2:MeOH (9:1)]; ^{1}H NMR (600 MHz, $CDCl_3$) δ 8.41 (1H, d, J = 2.5 Hz), 8.11 (1H, d, J = 2.4 Hz), 8.10 (1H, s), 7.80 (1H, dd, J = 8.8, 2.5 Hz), 7.67-7.60 (1H, m), 7.59 (1H, d, J = 2.0 Hz), 6.87 (1H, d, J = 1.9 Hz), 6.72 (1H, d, J = 8.5 Hz), 6.68 (1H, d, J = 8.8 Hz), 3.92 (3H, s), 3.88-3.80 (4H, m), 3.80-3.74 (2H, m), 3.58 (3H, s), 2.69 (1H, s), 2.06 (1H, s), 1.66 (1H, d, J = 8.7 Hz), 1.39 (6H, s), 1.27-1.24 (1H, m); ^{13}C NMR (151 MHz, $CDCl_3$)

δ 163.6, 158.6, 150.1, 147.9, 146.5, 139.4, 137.8, 132.7, 131.0, 130.3, 126.5, 120.4, 119.2, 115.9, 110.8, 104.2, 104.1, 103.6, 70.1, 57.8, 53.5, 47.0, 44.3, 30.3, 26.4; HRMS (ESI): found MH^+ 526.2556, $C_{29}H_{32}N_7O_3$ requires 526.2561.

8 Flow Chemistry Experiments

General Procedure for the Flow-Reaction – GP7

A solution of pyrazole in HFIP or in a mixture of HFIP and CH_2Cl_2 was introduced via a 10 mL or a 2 mL injection loop. The mixture was propelled using isopropanol as the solvent delivered by a peristaltic pump at various flow rates. The reaction mixture was then passed through a 10 mL PFA reactor coil (ID = 1 mm, L = 12.7 m, tR = 1.1 min) and was irradiated with a low-pressure mercury UVC lamp (λ = 254 nm, 99% intensity). After reaching steady-state conditions, the outflow was collected, concentrated under reduced pressure, and analysed by 1H NMR spectroscopy.

8.1 Optimisation of the Reaction Conditions for 1a

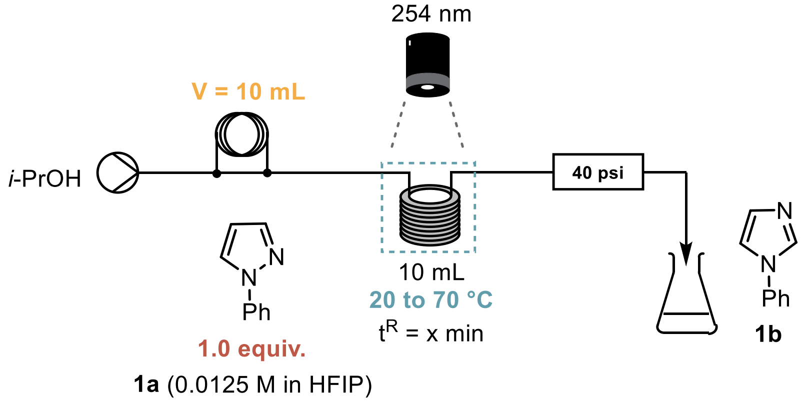

All the reaction parameters were optimized following **GP7** using **1a** (20 mg, 0.134 mmol, 0.0125 M) or (40 mg, 0.268 mmol, 0.025 M) in 11 mL solvent.

Table S11. Screening of temperature residence time and flow rate using 0.134 mmol **1a**.

entry	temperature (°C)	residence time (min)	flow rate ($mL.min^{-1}$)	1b (%)	1a (%)
**1**	20	20	0.5	79	21
**2**	40	25	0.4	99	1
**3**	60	10	1.667	97	3
**4**	70	7	1.428	90	10
**5**	70	11	0.909	**100**	–

Table S12. Screening of solvent mixture using 0.134 mmol **1a**,
when temperature is 70 °C, residence time is 11 min, flow rate is 0.909 mL.min^{-1}.

entry	ratio of HFIP (%)	ratio of CH_2Cl_2 (%)	1b (%)	1a (%)
**1**	80	20	100	–
**2**	50	50	100	–
**3**	25	75	100	–

Table S13. Screening of conditions using 0.268 mmol **1a**,
when temperature is 70 °C.

entry	ratio of HFIP (%)	ratio of CH_2Cl_2 (%)	residence time (min)	flow rate (mL.min^{-1})	1b (%)	1a (%)
**1**	80	20	11	0.909	80	20
**2**	50	50	11	0.909	74	26
**3**	50	50	22	0.455	98	2
**4**	25	75	22	0.455	87	13
**5**	10	90	22	0.455	52	48

Photochemical reactions under flow conditions are subject to diffusion limitations, resulting in a twofold increase in residence time when the concentration is doubled.

8.2 Optimisation of the Reaction Conditions for 26a

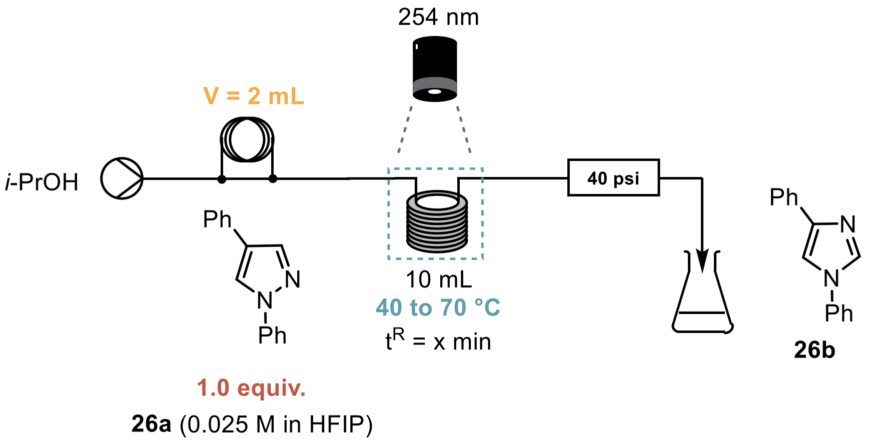

All the reaction parameters were optimized following **GP7** using **26a** (17 mg, 0.075 mmol, 0.025 M) in 3 mL HFIP.

Table S14. Screening of conditions using **26a**,

entry	temperature (°C)	residence time (min)	flow rate (mL.min^{-1})	26b (%)	26a (%)
**1**	40	30	0.333	75	25
**2**	40	45	0.222	94	6
**3**	60	15	0.667	67	33
**4**	70	15	0.667	74	26
**5**	70	25	0.4	93	7
**6**[a]	70	30	0.333	**94**	6

[a]: the reaction was also performed in a 1:1 mixture of HFIP and CH_2Cl_2, affording **26b** in 95% conversion.

8.3 Scale-up Reaction

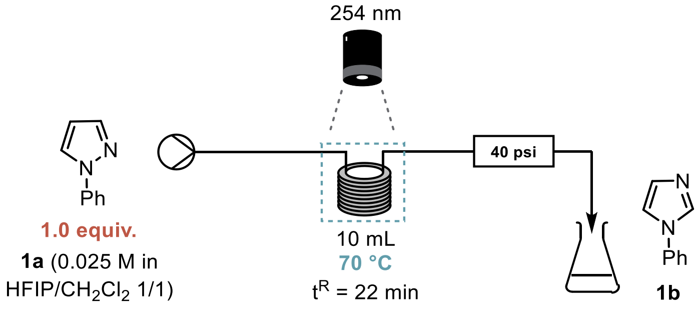

A solution of **1a** (180 mg, 1.25 mmol, 0.025 M) in a 1:1 mixture of HFIP/CH_2Cl_2 (50 mL) was pumped using a Vapourtec® R-Series flow reactor equipped with a peristaltic pump at a flow rate of 0.909 mL.min^{-1}, with isopropanol as the solvent. The reaction mixture was introduced into a 10 mL PFA reactor coil and irradiated at 254 nm for 22 min at 70 °C. After reaching steady-state conditions, the outflow was collected, concentrated under reduced pressure, and analysed by ^{1}H NMR spectroscopy. The crude mixture was purified by column chromatography on silica gel (eluent: 5:95 MeOH/CH_2Cl_2 + 0.5% Et_3N) to afford **1b** (131 mg, 96% yield) as a yellowish oil.

The reaction was conducted using a solution of **1a** (180 mg, 1.25 mmol, 0.0125 M) in a 1:1 mixture of HFIP/CH_2Cl_2 (100 mL). The reaction mixture was irradiated at 254 nm for 11 min at 70 °C. Following purification by silica gel column chromatography, **1b** was isolated as a yellowish oil (108 mg, 79% yield).

9 Mechanistic Studies

9.1 Light On/Off Experiments

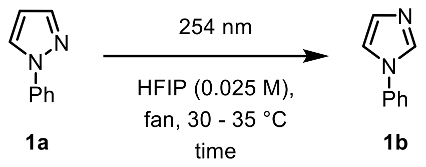

Following **GP*1**, but irradiating the reaction for 10 min and then stopping irradiation for 10 min, performing six cycles, **1a** (14 mg, 0.1 mmol, 1.0 equiv.) gave product **1b**, determined by NMR with using 1,3-dinitrobenzene as an internal standard.

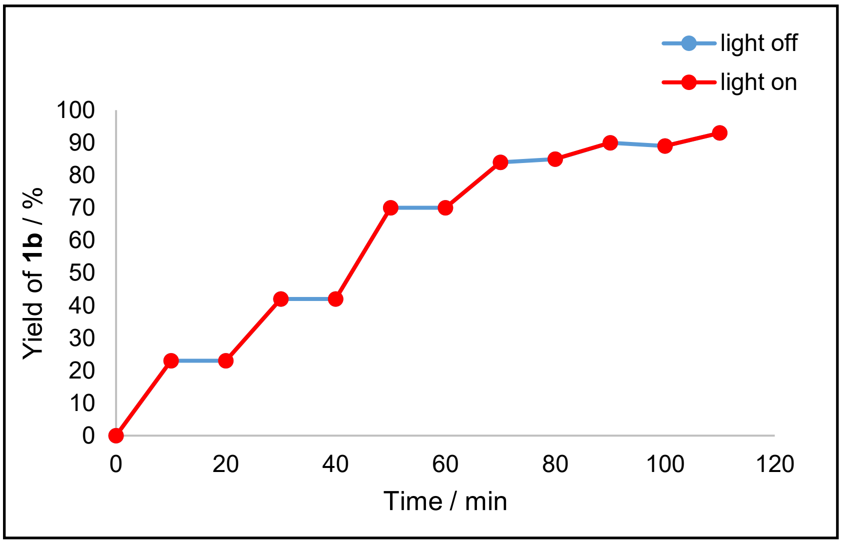

Figure S7. light on & off experiment of **1a**

These experiments demonstrate the reaction requires continuous irradiation to proceed.

9.2 Study on the azirine intermediate

Following **GP*1**, but concentration of 0.020 M, **75a** (18 mg, 0.1 mmol, 1.0 equiv.) gave **75b** (85%) as a white solid.

Ethyl 2-(Pyridin-2-yl)-2*H*-azirine-3-carboxylate (75a') was synthesized according to the literature.[59] Following **GP*1**, but irradiating the reaction for 1 h, **75a'** (16 mg, 0.1 mmol, 1.0 equiv.) gave **75b** (12%) as a permutation product, determined by NMR with using 1,3-dinitrobenzene as an internal standard.

Following **GP*1**, but without light irradiation and reacting for 1 h, **75a’** (16 mg, 0.1 mmol, 1.0 equiv.) didn’t give **75b** and 28% of **75a’** was recovered, determined by NMR with using 1,3-dinitrobenzene as an internal standard.

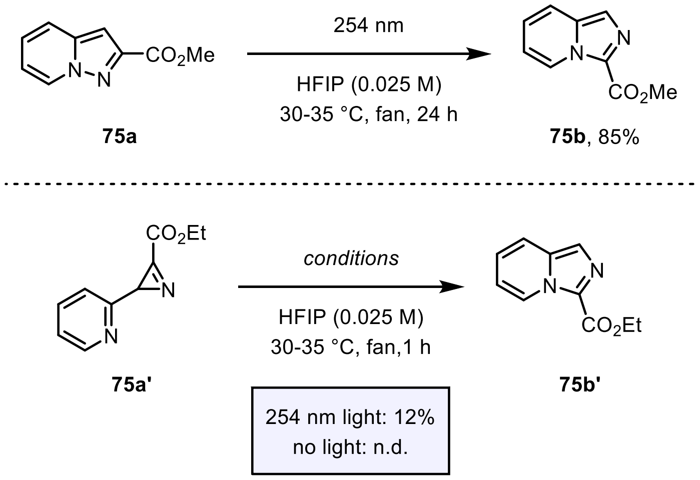

Scheme S1. Permutation of **75a** and the rearrangement of **75a’**

Following **GP*1**, pyrazolo[1,5-*a*]pyridine **75a** rearranged to the corresponding imidazo[1,5-*a*]pyridine **75b** in high yield. Similarly, under 254 nm irradiation, 2*H*-azirine **75a’** also transformed into the corresponding imidazo[1,5-*a*]pyridine **75b’**, albeit in low yield. Without light, 2*H*-azirine **75a’** did not react at all and only starting material was recovered.

2-phenylpyrazolo[1,5-*a*]pyridine (84a) was synthesized according to the literature.[60]

Following **GP*1**, **84a** (18 mg, 0.1 mmol, 1.0 equiv.) gave **3-phenylimidazo[1,5-*a*]pyridine (84b)** (17%).

Rearrangement of **2-(3-phenyl-2*H*-azirin-2-yl)pyridine (84a’)** to **84b** was reported in the literature.[61]

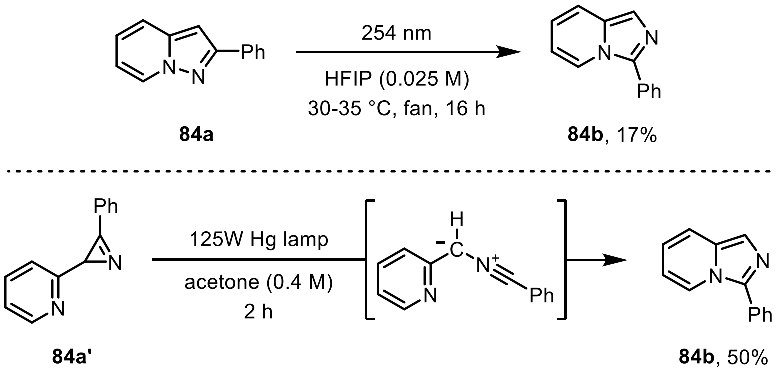

Scheme S2. Permutation of **84a** and literature precedent for the rearrangement of **84a’**

These two results support the involvement of 2*H*-azirine in the permutation of pyrazolo[1,5-*a*]pyridines as it has been proposed and supported by experimental results in the case of pyrazoles.

9.3 Kinetic Studies

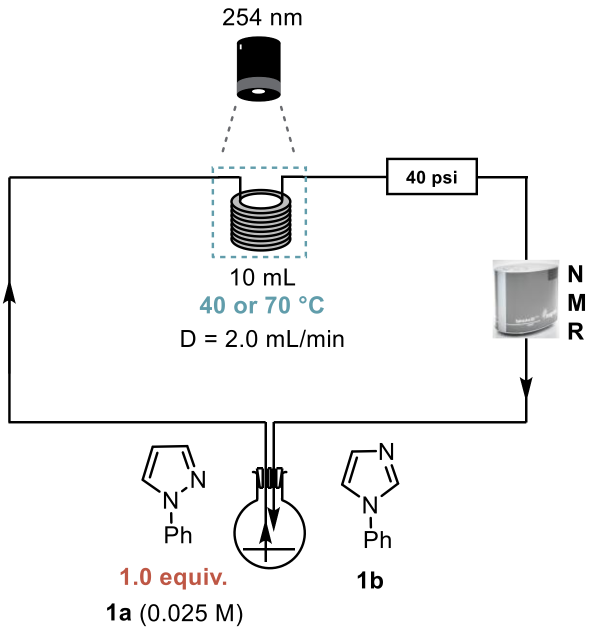

For the kinetic studies, a Magritek Spinsolve 80 Ultra benchtop NMR (80 MHz), a reaction monitoring kit supplied by Magritek® (glass flow tube with an expanded 4.2 mm internal diameter and a 0.5 mL internal volume) and a 40 psi BPR (Back Pressure Regulator) were used. In flow, ^{1}H NMR monitoring was conducted using the following settings on Spinsolve: 1 shim every 5 spectra, acquisition = 8 scans, acquisition time = 3.2 s, repetition time = 4 s, pulse angle 90 °, loops time 36 s — 155 s (without shim — with shim).

Experimental Procedure: A solution of **1a** (180 mg, 1.25 mmol) in HFIP (50 mL) or in a 1:1 mixture of HFIP and CH_2Cl_2 (50 mL) was prepared. The flow system was conditioned with the reaction mixture in the absence of irradiation. The solution was then circulated through the flow setup at a flow rate of 2.0 mL.min^{-1} and collected in the same flask. Irradiation (λ = 254 nm, 99% intensity) and ^{1}H NMR monitoring were initiated once only the starting material was observed on the ^{1}H NMR spectrum. The experiment was run for a total time of 20 min.

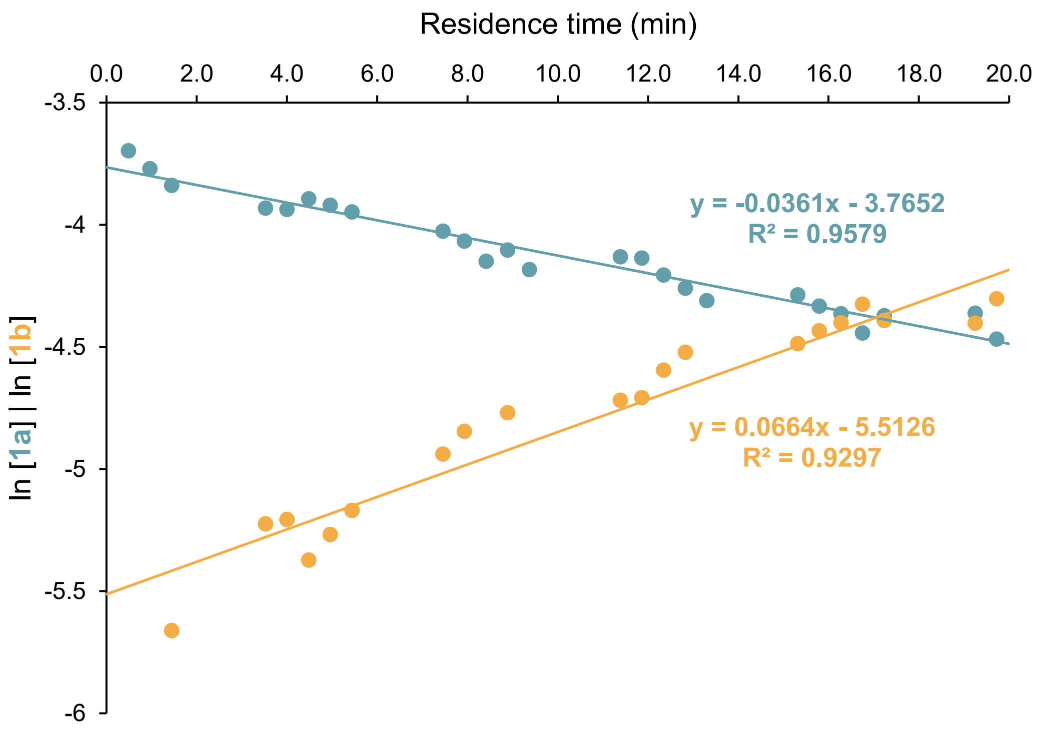

Figure S8. Kinetic study conducted at 40 °C in HFIP solution.

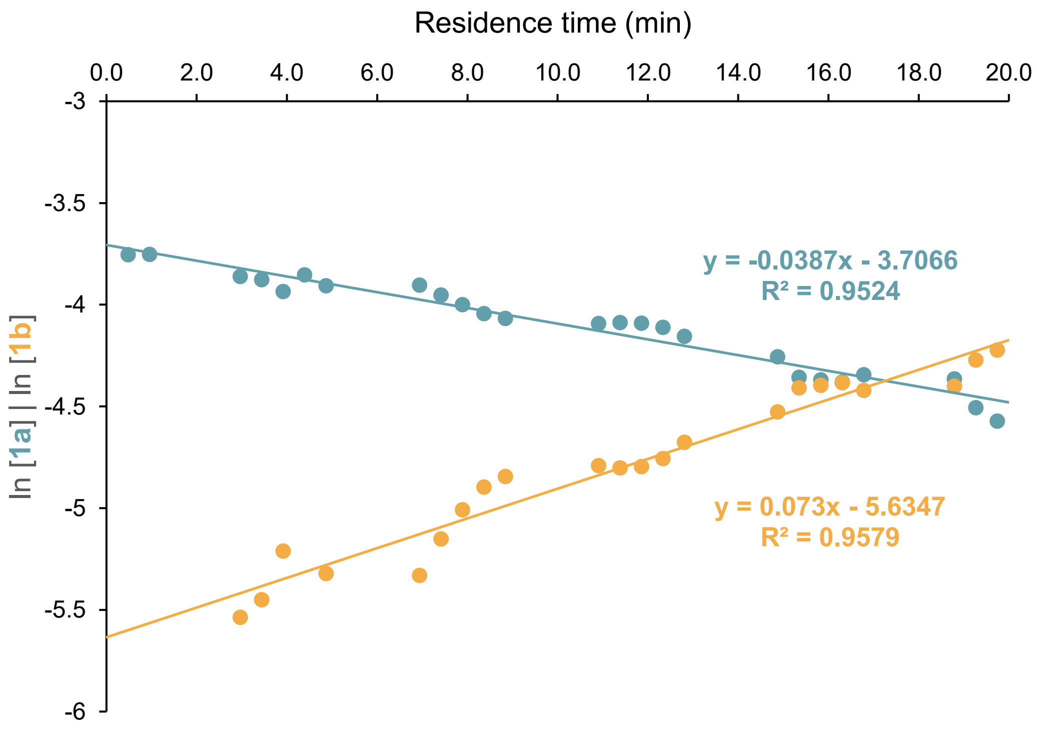

Figure S9. Kinetic study conducted at 40 °C in HFIP/CH_2Cl_2 (1/1) solution.

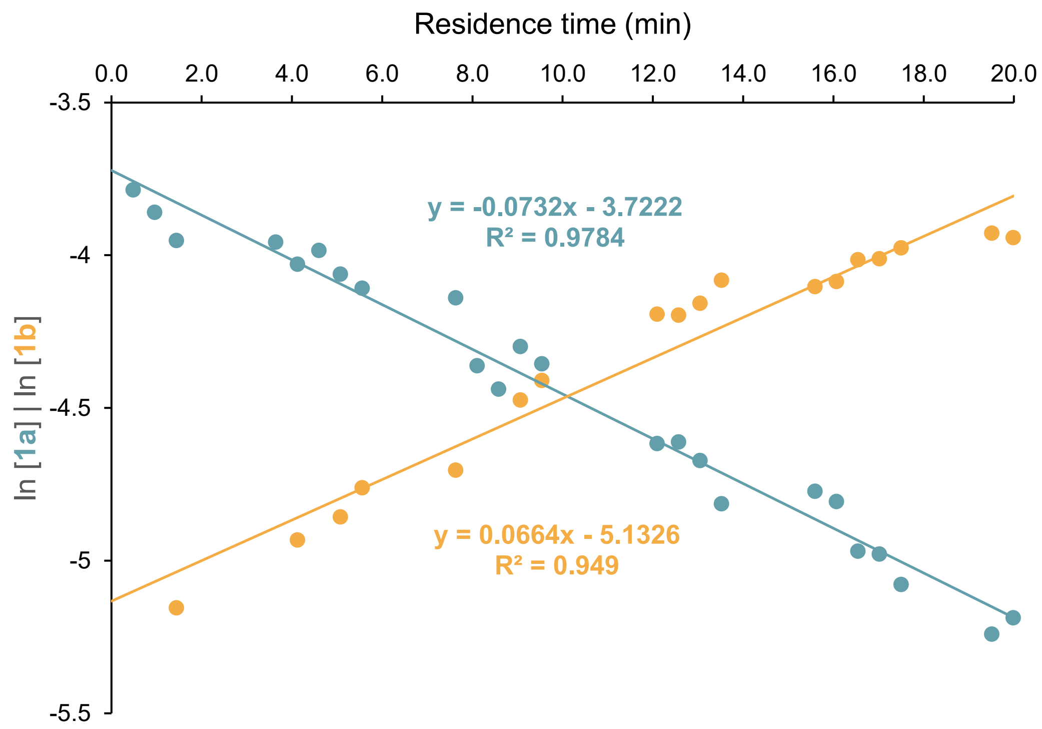

Figure S10. Kinetic study conducted at 70 °C in HFIP solution.

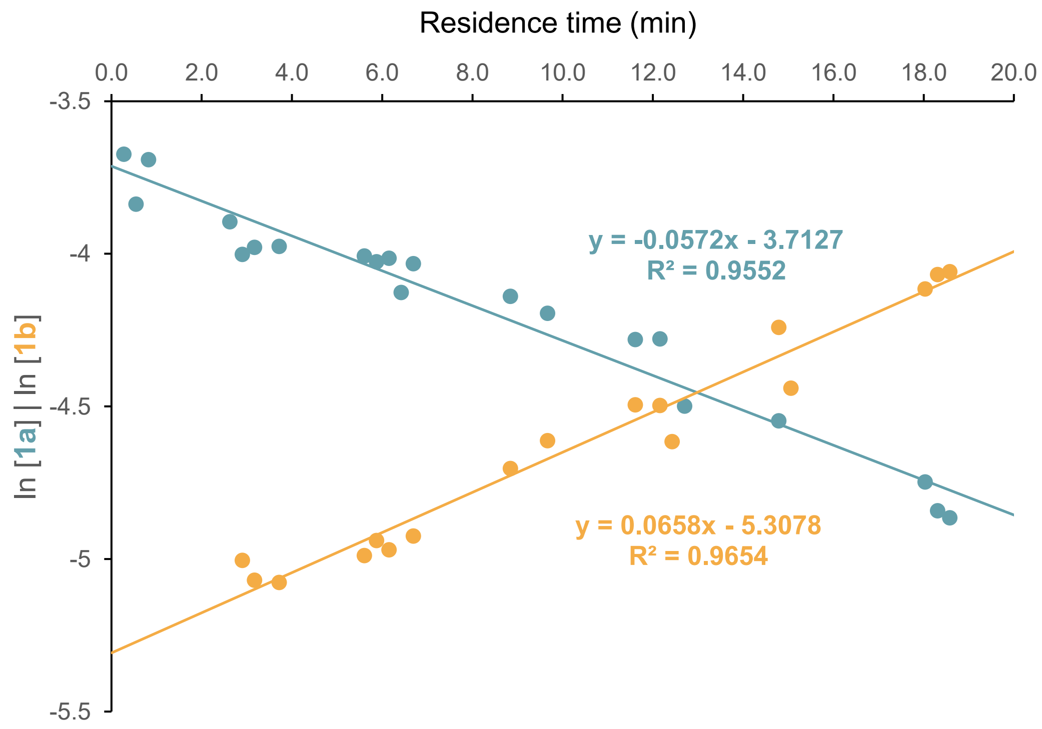

Figure S11. Kinetic study conducted at 70 °C in HFIP/CH_2Cl_2 (1/1) solution.

Since the logarithm of the concentration of **1a** as a function of time forms a straight line with a slope of -k, this indicates a **first-order reaction**.

9.4 Quantum yield measurement

The quantum yield was measured for the formation of **1a**. The reaction was performed using a Vapourtec® R-Series equipped with a UV-150 photoreactor, using a low-pressure mercury UVC lamp (λ_{max} = 254 nm, 99% intensity).

9.4.1 Determination of the Photon Flux – (*E*)-Azobenzene

All connecting tubing between the photoreactor and the vial of collect were covered with aluminium foil to prevent the actinometer solution from being exposed to ambient light. A concentrated solution of (*E*)-azobenzene (0.05 M in methanol) was pumped through the photoreactor and irradiated at 254 nm at various flow rate (between 0.29 and 9.09 $mL.min^{-1}$). Flow rates were selected based on the desired irradiation times, which ranged from 1.1 to 35 min. The solvent of the irradiated solution was evaporated in the dark at 20 °C and the resulting residue was dissolved in 500 µL of $CDCl_3$ for analysis by ^1H NMR spectroscopy. The NMR tube was kept in the dark as long as possible before its introduction into the spectrometer. The (*E*) to (*Z*) conversion was calculated by integrating the peaks that correspond to the (*E*)-azobenzene and to the (*Z*)-azobenzene isomers on the 1H NMR spectrum. The resulting data were used to determine the photon flux inside the reactor.

(1) Determination of the molar extinction coefficient for the (*E*)-azobenzene at 254 nm

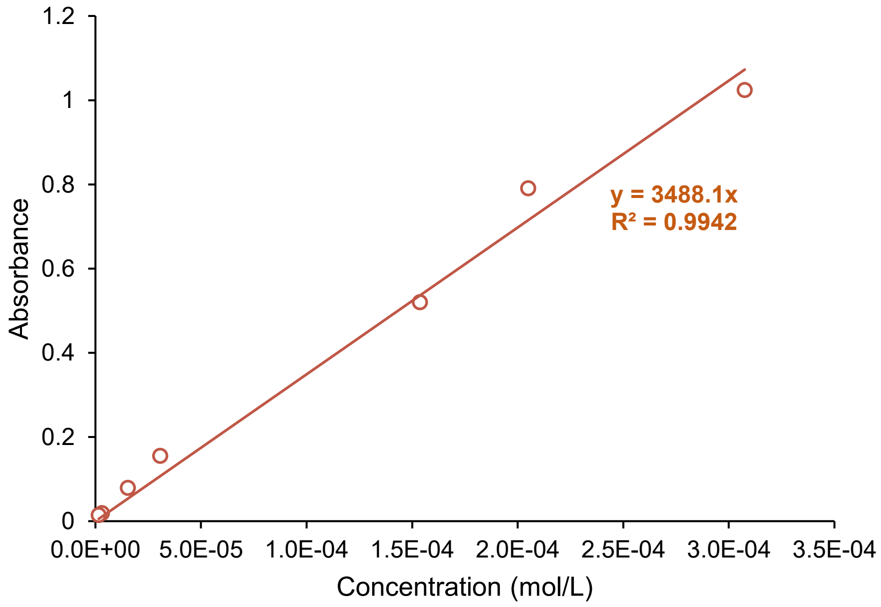

Figure S12. Plots of absorbance of (*E*)-azobenzene vs. its concentration (λ = 254 nm, in methanol).

(2) Determination of the Photostationary State at 254 nm

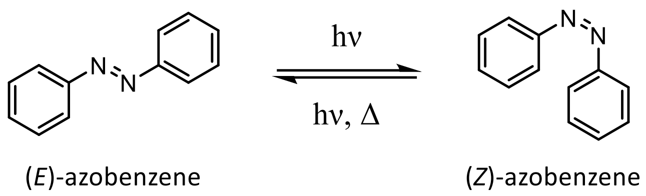

Scheme S3. Photoisomerization of azobenzene.

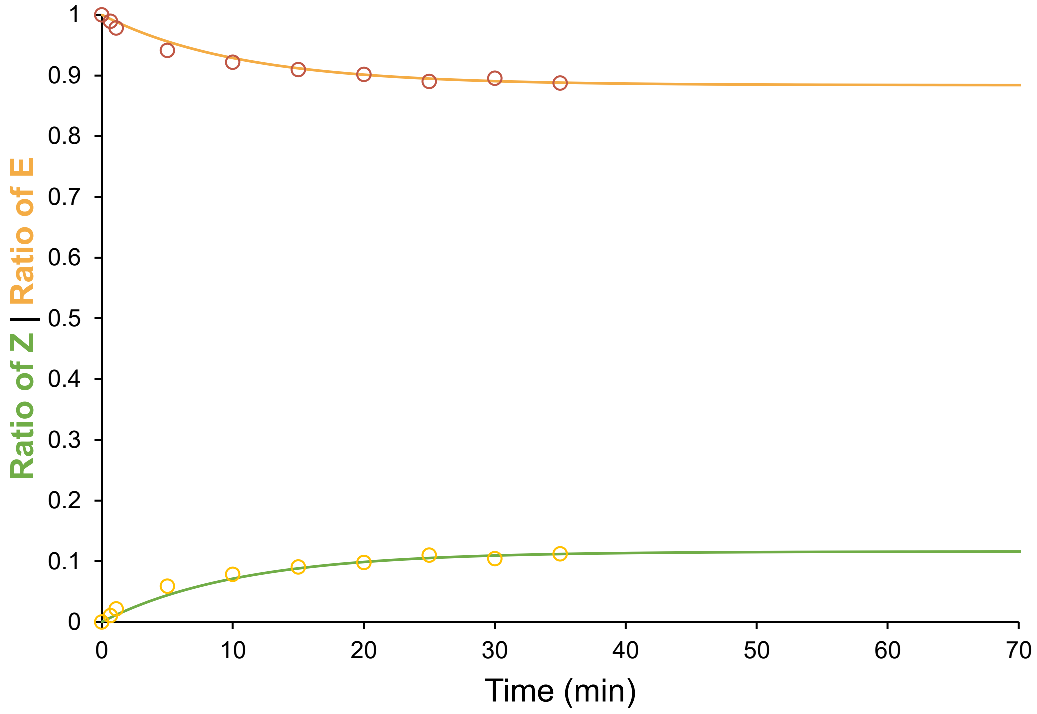

Figure S13. Evolution of (E)-azobenzene and (Z)-azobenzene at 254 nm.

(3) Determination of *k* (slope value)

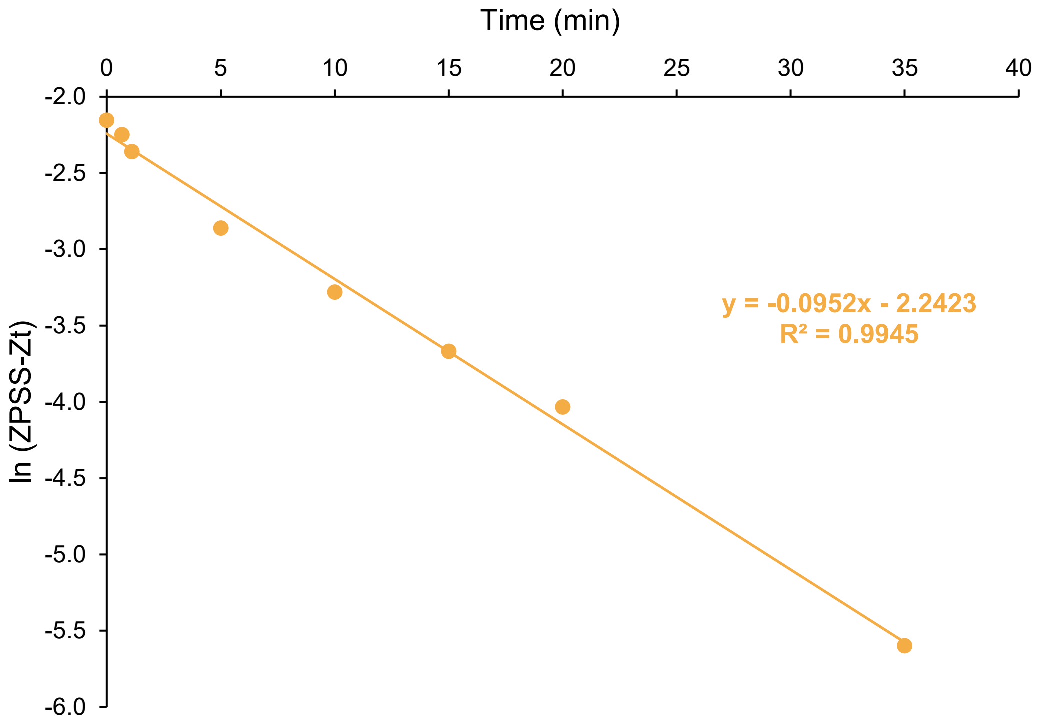

Figure S14. Photokinetic study of (*E*) ↔ (*Z*) azobenzene isomerization at 254 nm.

(4) Determination of the photon flux

The photon flux (I_{photon}) was calculated using equation **(1)**:[62]

$$I_{photon} = \frac{k.Z_{PSS}}{\ln 10.\Phi_E.\varepsilon_E} \qquad (1)$$

Where k is the slope of the photokinetic study of (*E*) ↔ (*Z*) azobenzene isomerization at 254 nm (k = 0.0952 min^{-1}), Z_{PSS} is the photostationary state composition (Z_{PSS} = 0.116), φ_E is the photoreaction quantum yield for the (*E*)-azobenzene actinometer (0.26 at λ = 254 nm),[63] ε_E is the molar extinction coefficient for the (*E*)-azobenzene at 254 nm (ε_E = 3488 L.mol^{-1}.cm^{-1} = 348.8 m^2.mol^{-1}).

$$I_{photon} = \frac{0.0952 \times 0.116}{\ln 10 \times 0.26 \times 348.8} = 5.2885.10^{-5}\ eins.min^{-1}.m^{-2}$$

$$I_{photon} = 8.8141.10^{-7}\ eins.s^{-1}.m^{-2}$$

9.4.2 Quantum Yield Determinations

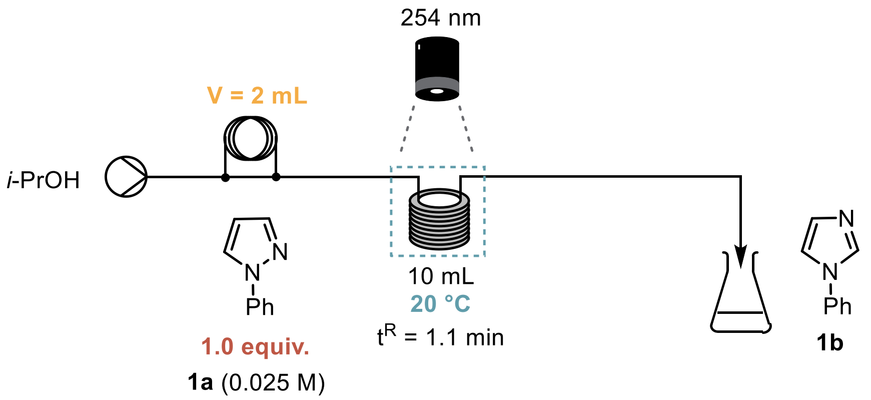

Following the **GP7**, a solution of **1a** (11 mg, 0.075 mmol, 0.025 M) in HFIP or in a 1:1 mixture of HFIP and CH_2Cl_2 (3 mL) was introduced using a 2 mL injection loop. The mixture was irradiated at 254 nm for 1.1 min at 20 °C. After irradiation, the solution was collected, concentrated, and the yield was determined by ^1H NMR spectroscopy using 1,3,5-trimethoxybenzene as an internal standard.

(1) Solvent: HFIP

The yield was determined to be 13.7% (average from 3 parallel experiments giving 11.6%, 13.9% and 15.5%) meaning $3.74.10^{-6}$ mol.

The quantum yield (φ) of the reaction was then calculated using equation **(2)**:

$$\Phi = \frac{n\ (product)}{I_{photon} \times t \times f_R} \qquad \textbf{(2)}$$

Where n (product) is the number of moles of (*Z*)-azobenzene formed during 1.1 min of reaction, I_{photon} the photon flux ($I_{photon} = 8.8141.10^{-7}$ eins.s^{-1}.m^{-2}), determined by actinometry as described above, t is the reaction time (66 s), and f is the fraction of incident light absorbed by the reaction mixture, where $f_R = 1 - 10^{-A}$. The absorbance (A) of the reaction mixture gave a value of 3.873 at 254 nm, so $f_R > 0.999$. The quantum yield of the reaction (φ) was determined to be φ = 0.064.

(2) Solvent: HFIP/CH_2Cl_2 (1/1)

The yield was determined to be 12.4% (average from 3 parallel experiments giving 12.8%, 12.1% and 12.3%) meaning $3.47.10^{-6}$ mol.

The quantum yield (φ) of the reaction was then calculated using equation **(2)**. The absorbance (A) of the reaction mixture gave a value of 1.246 at 254 nm, so $f_R = 0.943$.

The quantum yield of the reaction (φ) was determined to be φ = 0.063.

10 Computational Studies

10.1 Computational Details

To accurately characterize the excited-state properties, time-dependent density functional theory (TD-DFT)[65] calculations were employed. All computations were carried out with the CAM-B3LYP[65] functional in combination with the cc-pVDZ[66–70] basis set and the integration grid3 option, as implemented in ORCA 5.0.4[71–73]. Initially, ground state geometries were optimized at DFT level, and frequency calculations were performed at the same level of theory to confirm that all optimized structures corresponded to true minima on the potential energy surface. The resulting structures were subjected to TD-DFT calculations in order to evaluate vertical excitation energies and ensure a reliable description of the low-lying excited states. Particular attention was devoted to the characterization of the S_0/S_1 conical intersection, which was located and optimized at the same TD-DFT level of theory.

The reaction pathway was investigated using Density Functional Theory (DFT)[74–77] with the Gaussian16 C.01 package[78]. Geometry optimizations were carried out with the long-range corrected functional ωB97XD[79] in combination with the def2-TZVP[80] basis set, employing the unrestricted formalism. Frequency analyses at the same level of theory were used to confirm the nature of stationary points, ensuring the absence of imaginary frequencies for minima and the presence of a single imaginary frequency for transition states. No symmetry restrictions were applied.

Single-point energy calculations were performed with the cc-pVQZ[66–70] basis set. Solvent effects were included through the SMD solvation model[81]. Two solvents, HFIP and 1,4-dioxane, were considered, since byproduct was observed experimentally depending on the solvent. As Gaussian16 does not provide explicit parameters of HFIP, those of 2-propanol were used, with the parameters adjusted to match that of HFIP (dielectric constant, $\varepsilon = 16.7$; square of refractive index, $n^2 = 1.625625$; hydrogen bond acidity, $\alpha = 0.77$; hydrogen bond basicity, $\beta = 0.10$; surface tension at liquid-air interface, $\gamma = 23.23$ cal mol^{-1} Å^{-2}; carbon aromaticity, $\phi = 0.00$; electronegative halogenicity, $\psi = 0.60$).[82]

Thermodynamic properties were obtained by applying quasi-harmonic corrections to entropy within the rigid-rotor/harmonic oscillator (RRHO) approximation[83], where vibrational frequencies below 100 cm^{-1} were treated as hindered rotors with a damping interpolation. These corrections were carried out using *Goodvibes.py*[84], assuming standard solution-phase conditions (298.15 K, c = 1 mol L^{-1}). All energies are reported in kcal mol^{-1}. Cartesian coordinates and thermodynamic data are provided in the electronic supplementary materials.

10.2 Excited State Calculations of Pyrazoles

To gain further insight into the role of excited states in the photochemical pathway, conical intersection points were systematically optimized at TDCAMB3LYP/cc-pVDZ level of theory.

For **1a**, the conical intersection **CX1** was identified. Upon excitation, **1a** populates the singlet excited state, reaching the Frank-Condon (FC) region at 122.4 kcal mol^{-1}. The intersection **CX1** was located at 88.6 kcal mol^{-1}, considerably lower than the *FC* region, and therefore energetically accessible; its optimized geometry (**Table S15**) shows structural features consistent with the formation of the biracial intermediate **A1**.

For **A2**, the ground-state energy was calculated to be 51.6 kcal mol^{-1} relative to **1a**. Upon vertical excitation, the system is promoted to the FC region at 156.5 kcal mol^{-1}. The conical intersection **CX2** was identified at 120.2 kcal mol^{-1}, also relative to **1a**, and therefore lies substantially below the FC region. The optimized geometry of **CX2** (**Table S15**) shows elongation of the C^3–C^4 bond, consistent with bond cleavage, and is predisposed to evolve into the biradical intermediate **A3**.

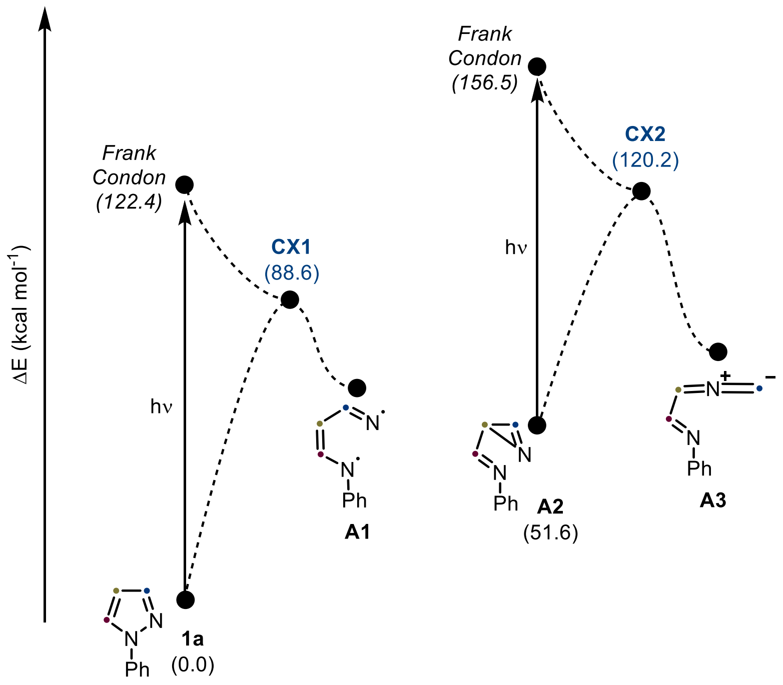

Figure S15. Schematic indicating the energies of **1a**, **CX1**, **A1** and **CX2** at TDCAM-B3LYP/cc-pVDZ level of theory

Table S15. Selected geometric parameters of **1@S$_0$, 1@CX1, A1@CX1**, and **A1@CX2** at TDCAM-B3LYP/cc-pVDZ level of theory

	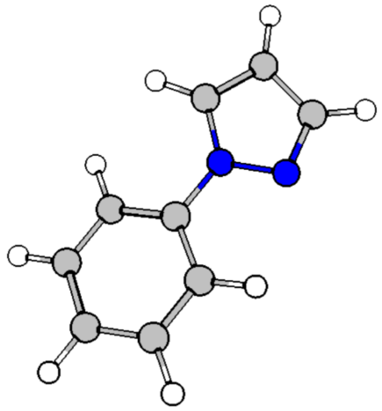 **1@S$_0$**	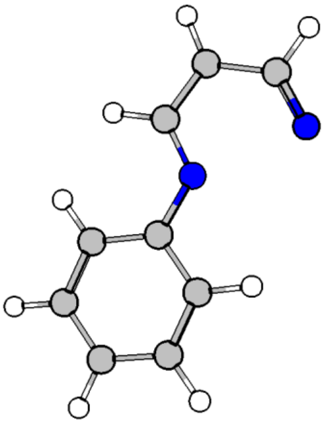 **1@CX1**
**R (N^1–N^2)**	1.345 Å	2.479 Å
	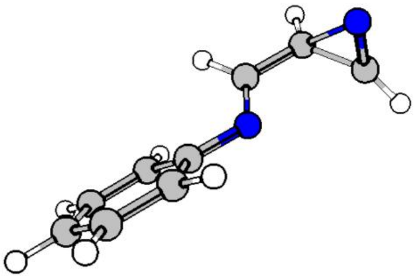 **A1@S$_0$**	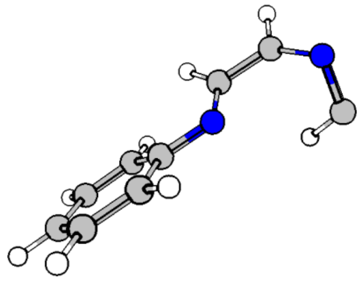 **A1@CX2**
**R (C^3–C^4)**	1.456 Å	1.456 Å
**α(C^3–N^2–C^4)**	61.7°	115.7°

10.3 Electronic Structure of Biradical Intermediates (A1 and A1')

The ground-state electronic structures of the intermediates **A1** and **A1'** (as shown in **Scheme S4**) were analyzed. For both species, calculations performed in the two solvent environments yielded spin contamination values of approximately $\langle S^2 \rangle \sim 1.1$. These values indicate a pronounced open-shell character in the ground states of both intermediates.

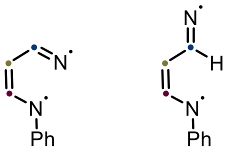

A1 **A1'**

Scheme S4. A1 and **A1'** intermediates

Mulliken spin density analysis indicates that the largest portion of spin density is localized on the terminal nitrogen atom, while a notable contribution is also present on the pyrrolic nitrogen (**Figure S18**). Additional but smaller spin densities are distributed across the adjacent carbon atoms of the heteroaromatic ring, suggesting partial delocalization of the radical character within the five-member core. In contrast, the phenyl substituent carries only negligible spin density, indicating that it does not significantly contribute to the stabilization of the biradical species. Overall, the results confirm that the open-shell character of both intermediates is mainly concentrated on the heteroaromatic framework, with only minor extension onto the substituent.

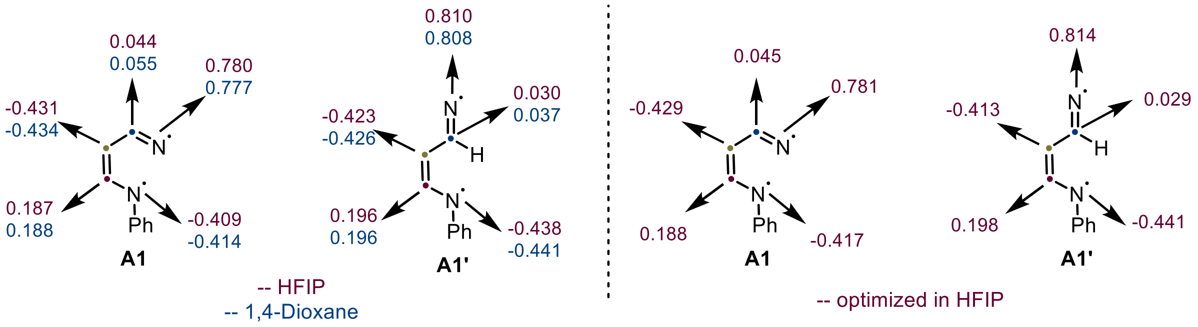

Figure S16. Mulliken Spin Densities of **A1** and **A1'** at UωB97XD/cc-pVQZ (SMD,Solvent)//def2-TZVP level of theory

The spin density plots of intermediates **A1** and **A1'** in both HFIP and 1,4-dioxane show that the unpaired electrons are primarily localized on the terminal nitrogen atom and, to a lesser extent, on the adjacent pyrrolic nitrogen. This distribution confirms the open-shell biradical character of the intermediates. Minor spin delocalization into the heteroaromatic carbon framework is observed, while the phenyl substituent contributes negligibly to the overall spin density. Importantly, the comparison between HFIP and 1,4-dioxane indicates that the solvent environment does not significantly alter the qualitative distribution of spin density.

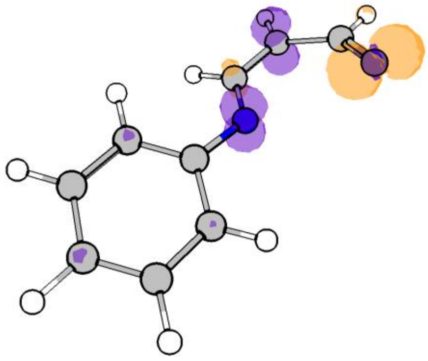

Total Spin Density Plot of **A1**

HFIP

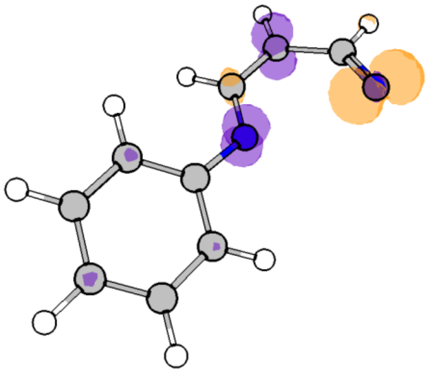

Total Spin Density Plot of **A1**

1,4-Dioxane

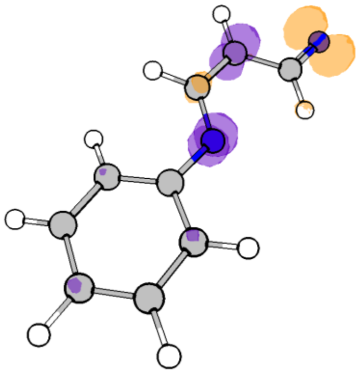

Total Spin Density Plot of **A1’**

HFIP

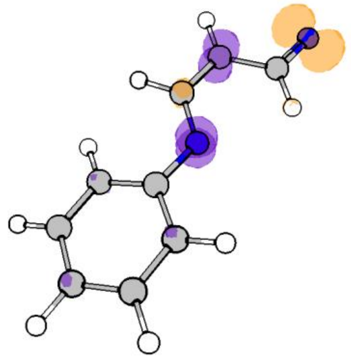

Total Spin Density Plot of **A1’**

1,4-Dioxane

Figure S17. Total Spin Density Plots of **A1** and **A1’** at UωB97XD/cc-pVQZ (SMD,Solvent)//def2-TZVP level of theory

10.4 Rotational Interconversion of A1 and A1’

The rotation about the C^3–C^4 bond was explored by a relaxed scan of the C^5–C^4–C^3–N^2 dihedral. A smooth single-barrier profile was obtained, connecting the two biradical conformers **A1** and **A1’**. The barrier height was found to be modest, indicating that interconversion by internal rotation is readily accessible. Thus, C^3–C^4 rotation provides a feasible pathway between the two intermediates, with **A1’** favored thermodynamically.

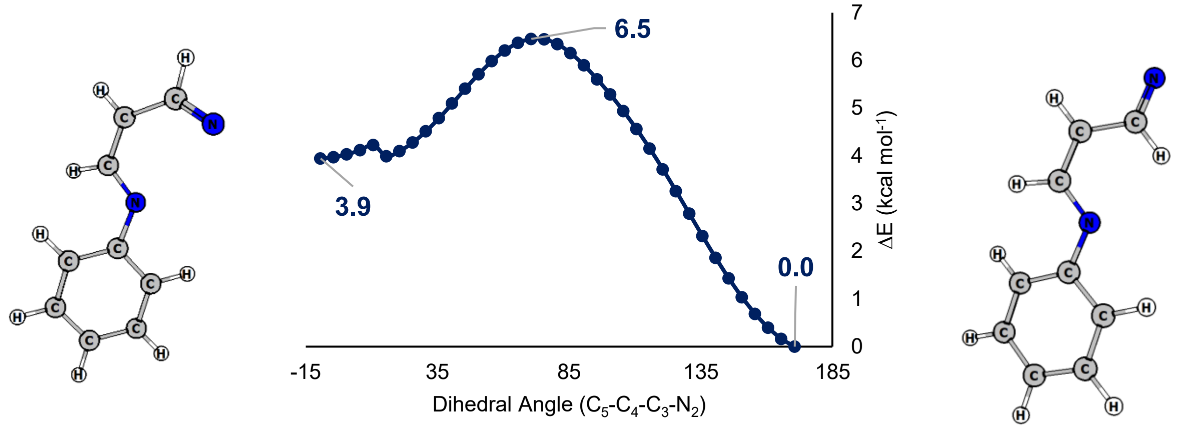

A1 UωB97XD/def2-TZVP **A1’**

Figure S18. Relaxed potential energy scan of the C^5–C^4–C^3–N^2 dihedral angle performed at UωB97XD/def2-TZVP level of theory, illustrating of biradical intermediates **A1** and **A1’** through rotation about the C^3–C^4 bond. Relative energies are reported in kcal mol^{-1}.

To further validate the relative stability of the two biradical intermediates, the energy difference between **A1** and **A1’** was also evaluated at a higher level of theory. The calculations consistently confirmed that **A1**’ is thermodynamically more stable than **A1**, in agreement with the results obtained about the dihedral scan. This outcome supports the conclusion that rotation about the C^3–C^4 bond provides a feasible pathway for the interconversion of the two intermediates, with **A1’** being favored energetically.

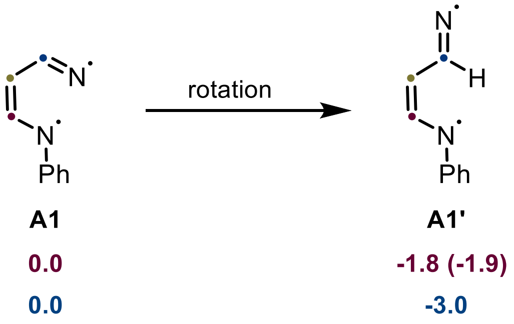

Figure S19. Relative energies of biradical intermediates **A1** and **A1’** calculated at the UωB97XD/cc-pVQZ (SMD,Solvent)//def2-TZVP level of theory; UωB97XD/cc-pVQZ (SMD,HFIP)//def2-TZVP (SMD, HFIP) in paranthesis. Results are shown for two solvent environments: HFIP (red) and 1,4-Dioxane (blue). (kcal mol^{-1})

10.5 Reaction Profile

To gain insight into the productive photochemical transformation, the reaction profile in HFIP was calculated (**Figure S20**). Photoexcitation of **1** generates the biradical intermediate **A1** at 60.2 kcal mol^{-1}

relative to starting material. From this species, progression through **TS3** (63.0 kcal mol^{-1}) enables the formation of the azirine intermediate **A2**, which is thermodynamically stabilized (40.2 kcal mol^{-1}). Further reactivity of **A2** requires cleavage of the C^3–C^4 bond. At the ground state, this process is prohibitively high barrier of 46.4 kcal mol^{-1}, rendering a thermal pathway inaccessible. Under photochemical conditions, however, population of the excited state provides an alternative route: at the conical intersection, the C^3–C^4 bond can be cleaved efficiently to generate **A3** (51.1 kcal mol^{-1}). Subsequent rearrangement of **A3** proceeds via **TS6** (4.9 kcal mol^{-1}), followed by intramolecular ring closure, ultimately producing the final product **2**, which is stabilized at -1.8 kcal mol^{-1}).

Overall, this analysis demonstrates that the productive pathway involves initial formation of the azirine intermediate **A2**, followed by photochemically assisted C–C bond cleavage yield **A3**, and a subsequent ring-closing step leading to the thermodynamically favoured product **1b**.

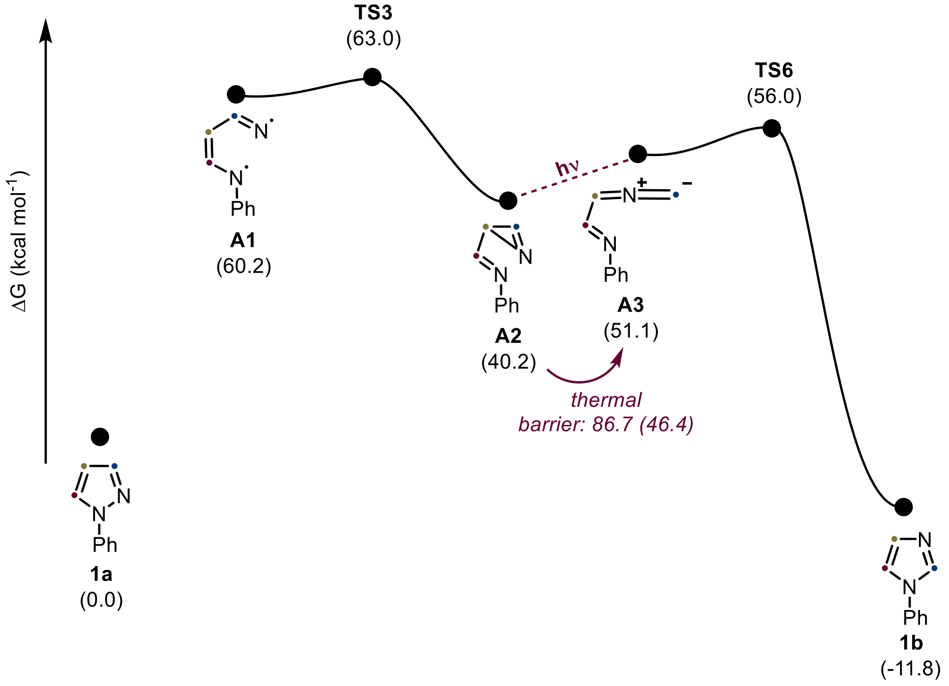

Figure S20. Calculated reaction energy profile for **1a**. The numbers are the relative Gibbs free energies at UωB97XD/cc-pVQZ (SMD, HFIP)//def2-TZVP level of theory (kcal mol^{-1})

In 1,4-dioxane, the photogenerated biradical **A1** can evolve along two competing pathways. In the first pathway, internal rotation about C^3–C^4 bond converts **A1** into the alternative biradical **A1'** via **TS7** (2.3 kcal mol^{-1}). This conformer subsequently promotes hydrogen atom transfer, leading to the byproduct **1c**

(-1.8 kcal mol^{-1}). The relatively low barrier associated with this pathway makes byproduct formation kinetically feasible, in line with experimental observations.

In the second pathway, **A1** rearranges to the azirine intermediate **A2** (44.9 kcal mol^{-1}) via **TS3** (2.6 kcal mol^{-1}). Similar to the situation in HFIP, thermal C^3–C^4 bond cleavage is associated with a prohibitively high barrier (43.5 kcal mol^{-1}), but photoexcitation enables access to **A3** (51.0 kcal mol^{-1}) through a conical intersection. From there, rearrangement via **TS6** (4.3 kcal mol^{-1}) and subsequent ring closure furnish the stable product **1b** (-12.3 kcal mol^{-1}).

Thus, in 1,4-dioxane, both the productive pathway leading to **1b** and the competing side reaction forming **1c** are accessible. The former proceeds through azirine formation and photochemically assisted band cleavage, ultimately yielding the thermodynamically favoured product **1b**, whereas the latter arises from conformational reorganization and hydrogen atom transfer, producing the byproduct **1c**.

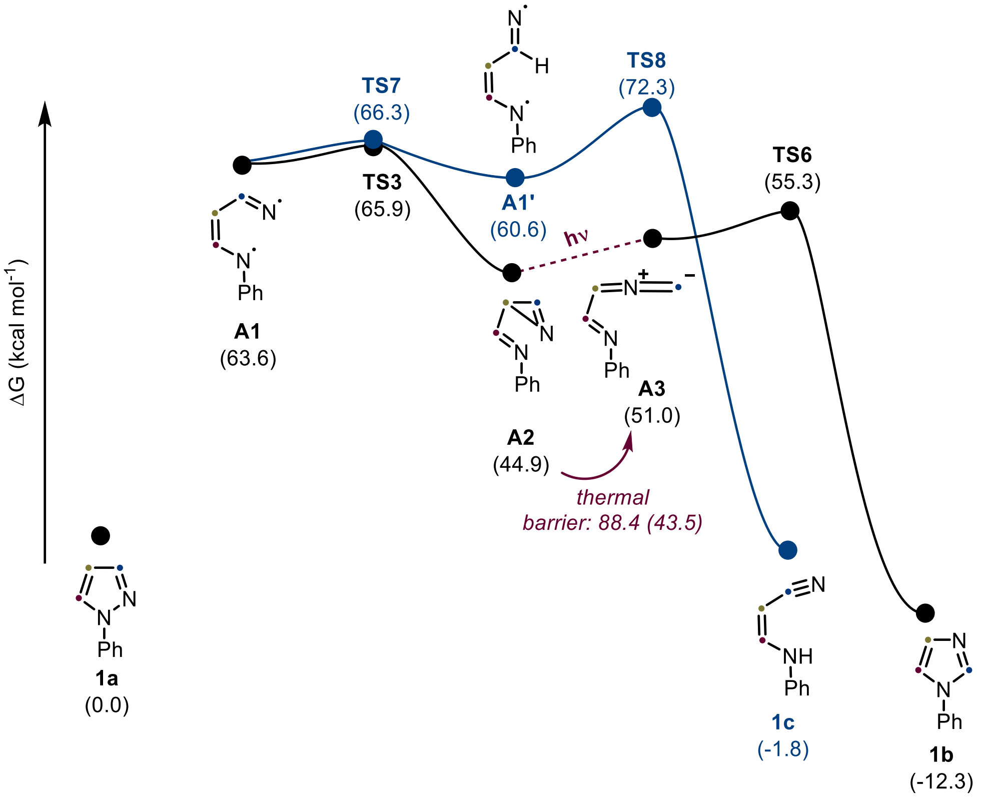

Figure S21. Calculated reaction energy profile for 1-phenyl-1*H*-pyrazole. The numbers are the relative Gibbs free energies at UωB97XD/cc-pVQZ (SMD, 1,4-Dioxane)//def2-TZVP level of theory (kcal mol^{-1})

10.6 Tunneling Calculations

Classical Transition State Theory (TST) determines the reaction rate constant based on the statistical

partition functions of reactants and the transition state (TS), assuming that all reactive trajectories must surmount the potential energy barrier. This formalism inherently neglects quantum mechanical tunneling, the process by which particles penetrate the barrier. While adequate for reactions involving heavy atoms at moderate or high temperatures, this approximation fails for light-atom transfer reactions, such as hydrogen atom transfer (HAT) and proton-coupled electron transfer (PCET), or at low temperatures, where tunneling significantly enhances the reaction rate.

To account for this deficiency, a tunneling transmission coefficient (κ) is introduced as a multiplicative correction to the conventional TST rate constant ($k_{TST}(T)$):

$$k(T) = \kappa(T) \cdot k_{TST}(T) \qquad \textbf{(3)}$$

Here, k is the corrected rate constant, and κ quantifies the increase in transmission probability due to quantum tunneling.

Common semi-classical approximation to estimate κ include Wigner,[85] Skodje-Truhlar,[86] and Eckart[87,88] models. The Wigner correction assumes a simple symmetric parabolic barrier and is valid primarily for small tunneling corrections. To address the limitations of parabolic approximation at lower temperatures, the Skodje-Truhlar method employs truncated potential. A more rigorous treatment is provided Eckart model, which explicitly incorporates barrier asymmetry, thereby yielding a more realistic description of the potential energy surface.

(1) Wigner Correction

The Wigner method applies a second-order perturbative correction assuming a symmetric, inverted parabolic potential at the transition state. It is defined as:

$$\kappa_{Wigner} = 1 + \frac{1}{24}\left(\frac{h\upsilon^{\ddagger}}{k_B T}\right)^2 \qquad \textbf{(4)}$$

where h is Planck's constant, k_B is the Boltzmann constant, and $\upsilon^{\ddagger}$ is the magnitude of the imaginary vibrational frequency at the saddle point.

(2) Skodje-Truhlar Corrections

The Skodje-Truhlar model extends the parabolic approximation to lower temperatures using a truncated potential. Within this framework, the tunneling transmission coefficient is expressed through two dimensionless parameters,

$$\alpha = \frac{2\pi}{h\upsilon^{\ddagger}} \qquad\qquad \beta = \frac{1}{k_B T} \quad \textbf{(5)}$$

Which relate κ_S the quantum-mechanical time scale associated with the transition-state frequency to the thermal energy at temperature T. The transmission coefficient is defined by a piecewise analytic function, given by:

$$\kappa_S = \begin{cases} \frac{\pi\beta}{\alpha \sin\left(\frac{\pi\beta}{\alpha}\right)} + \frac{\beta}{\beta-\alpha} e^{(\beta-\alpha)V_B} & (\alpha > \beta) \\ \beta V_\beta & (\alpha = \beta) \\ \frac{\beta\left(e^{(\beta-\alpha)V_B - 1}\right)}{\beta-\alpha} & (\alpha < \beta) \end{cases} \quad \textbf{(6)}$$

where V_B denotes the potential barrier height corrected for zero-point energy effects.

(3) Eckart Correction

The Eckart model accounts for barrier asymmetry, which is critical for accurate kinetics in exo- or endothermic reactions. The transmission coefficient is obtained by integrating the transmission probability P(E) over the Boltzmann distribution:

$$\kappa_E = \frac{\int_0^\infty e^{-E/(k_B T)} P_{Eckart}(E) dE}{\int_0^\infty e^{-E/(k_B T)} dE} \quad \textbf{(7)}$$

The probability P(E) is derived analytically from the Eckart potential, parametrized by the forward barrier (ΔV_f), reverse barrier (ΔV_r), and the imaginary frequency ($\upsilon^{\ddagger}$).

(4) Application to **TS8**

The transition state **TS8** facilitates the hydrogen atom transfer converting **A1'** to **1c** as shown in **Scheme S5**. This structure is characterized by a high imaginary frequency of 1461.9 cm^{-1}, which implies a marrow potential barrier and significant quantum tunneling contributions.

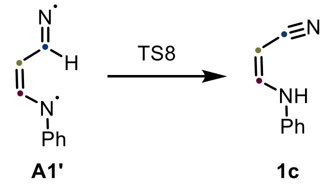

Scheme S5. Hydrogen atom transfer step converting **A1'** to **1c**

To strictly account of these effects, tunneling coefficients were computed using Wigner, Skodje-Truhlar, and Eckart models via the *PyTun.py* [89]script. The calculated correction factors are listed in **Table S16**.

Table S16. Calculated tunneling coefficients for TS8 at UωB97XD/cc-pVQZ (SMD, 1,4-Dioxane)//def2-TZVP level of theory

	$\boldsymbol{\kappa_W}$	$\boldsymbol{\kappa_S}$	$\boldsymbol{\kappa_E}$
**TS8**	3.06	60.36	14.55

11 Comparison Between Permutation Reactivity and Current Synthetic Approaches for Imidazole Synthesis

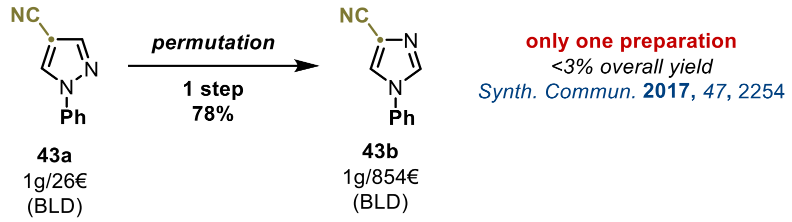

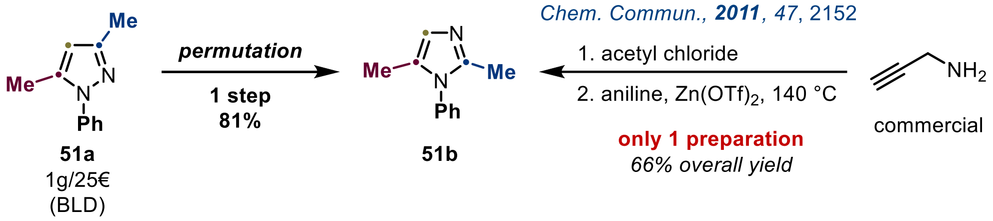

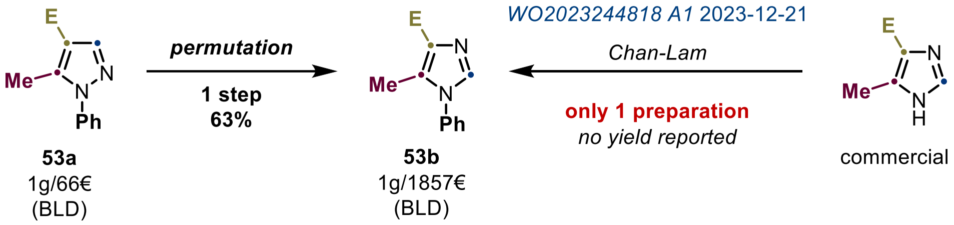

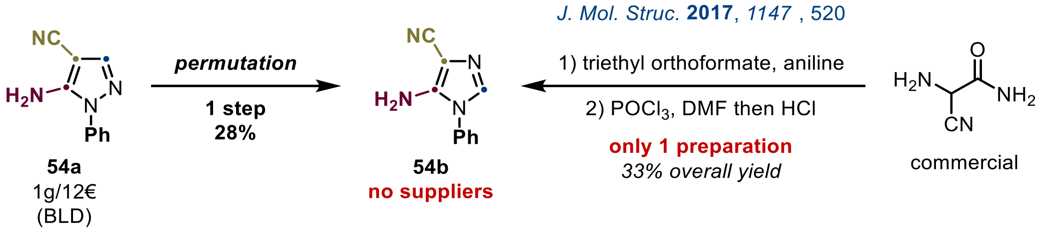

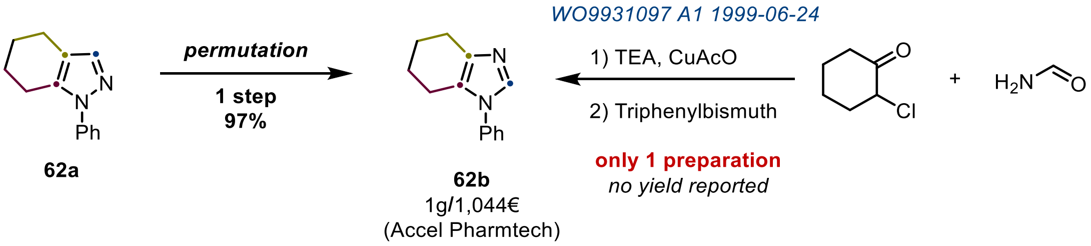

Chem Heterocycl Comp **2021**, *57*, 554–559

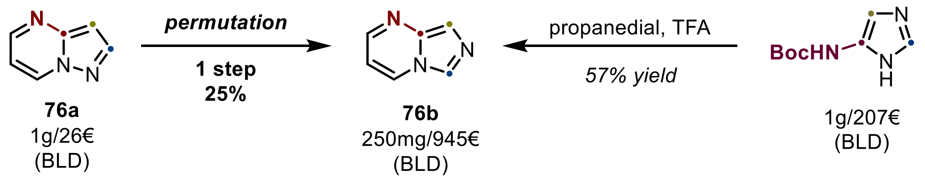

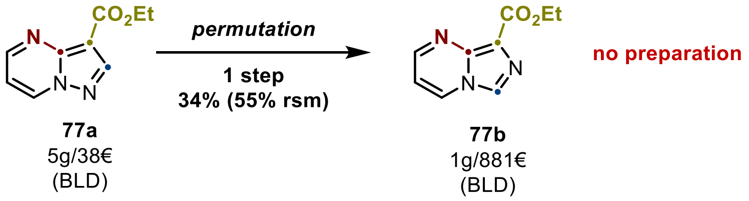

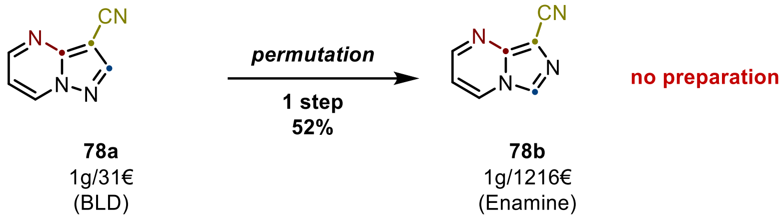

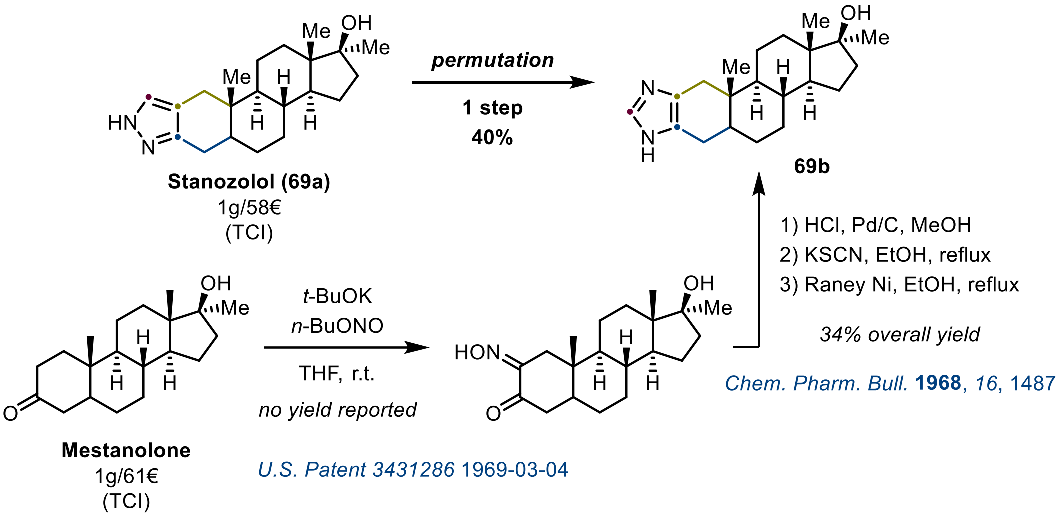

WO2006084835 A1 2006-08-16

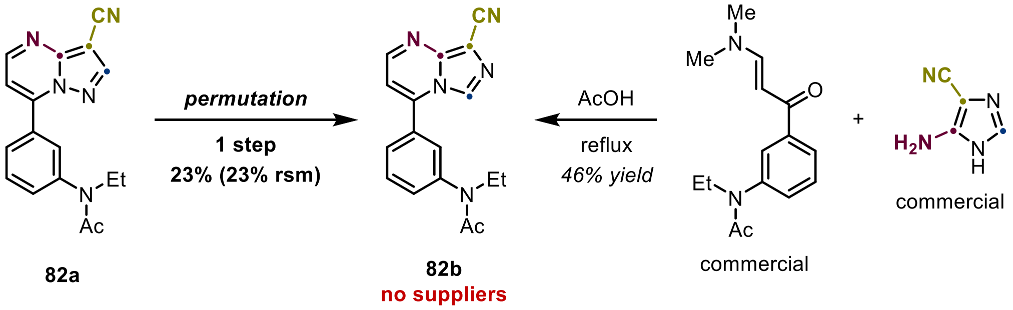

12 NMR Spectra

4-Methoxy-1-phenyl-1*H*-pyrazole (20a)

^{1}H NMR (600 MHz, $CDCl_3$)

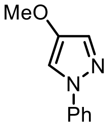

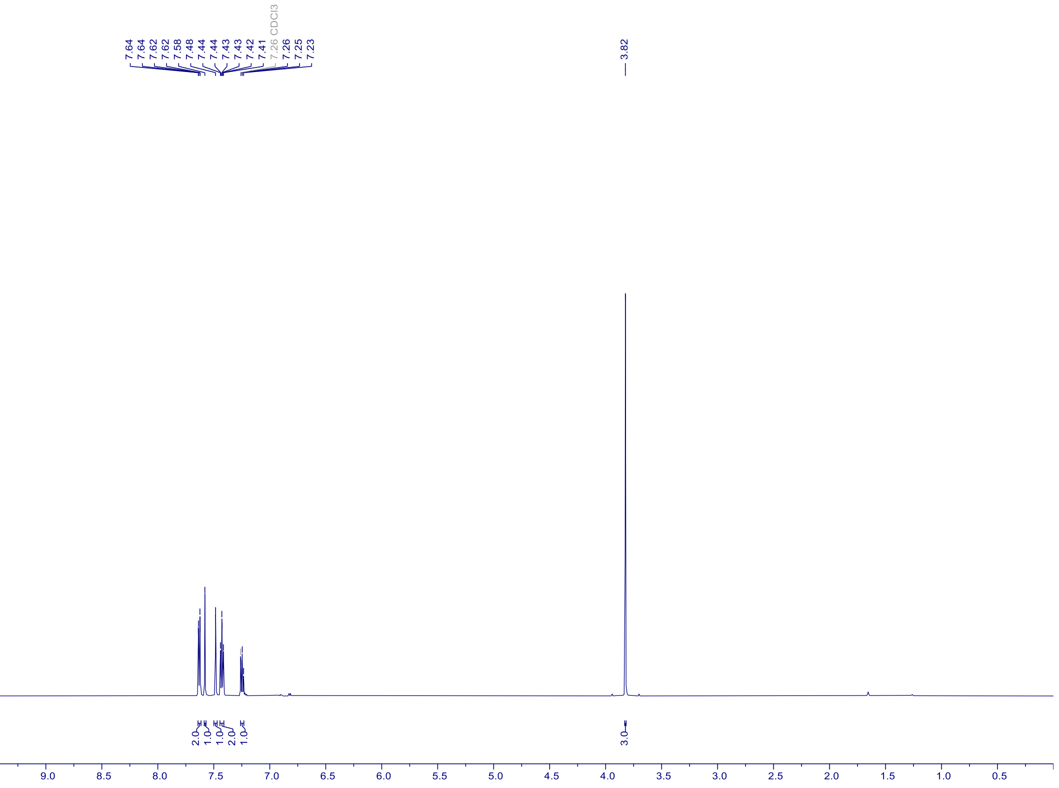

^{13}C NMR (151 MHz, $CDCl_3$)

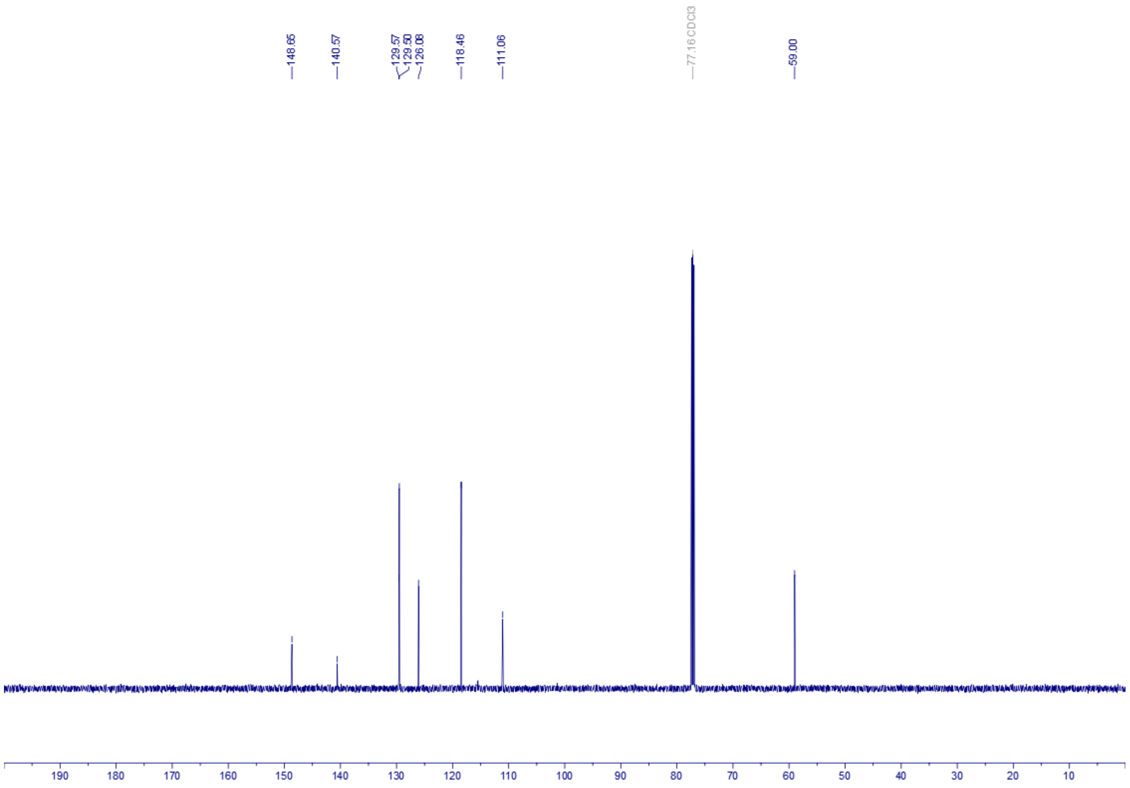

4-Phenyl-1-(4-(trifluoromethyl)phenyl)-1*H*-pyrazole (32a)

^{1}H NMR (600 MHz, $CDCl_3$)

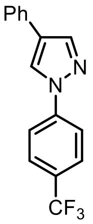

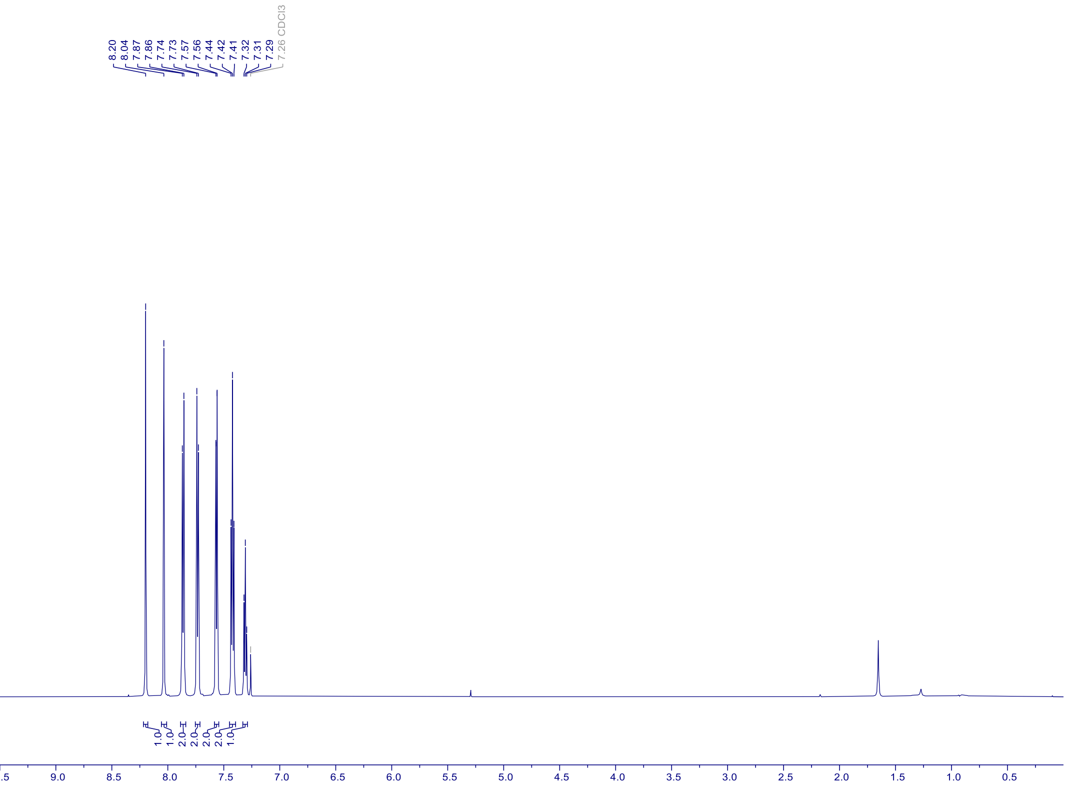

^{13}C NMR (151 MHz, $CDCl_3$)

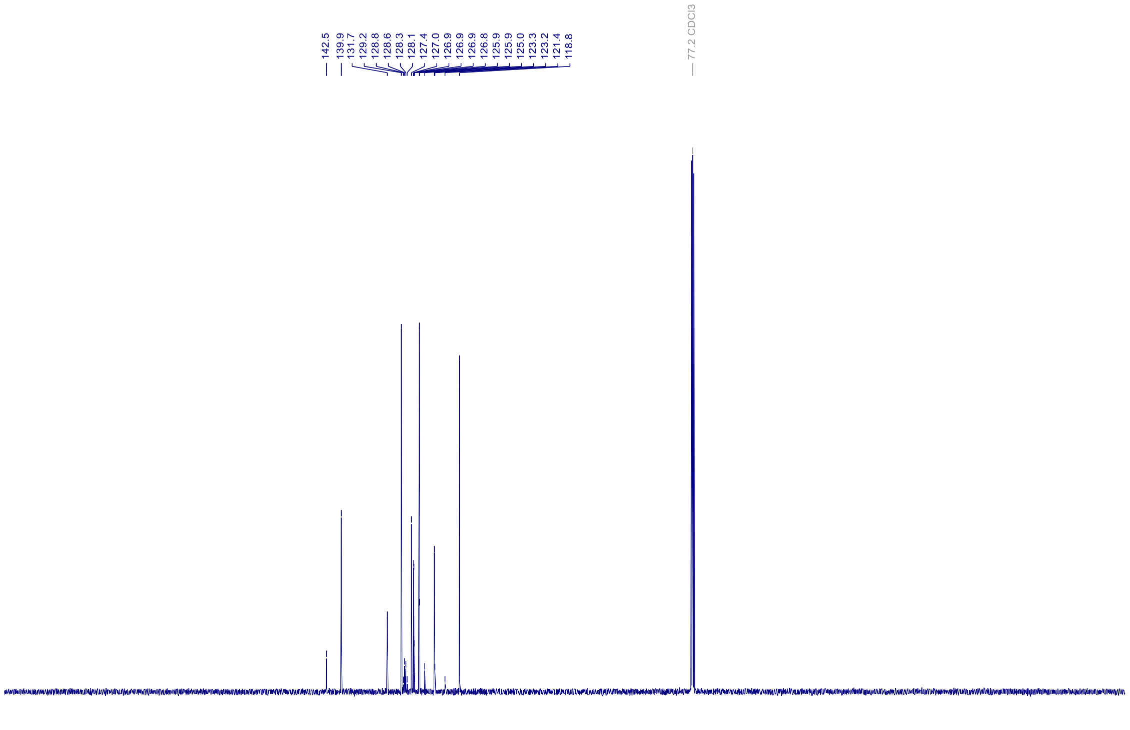

^{19}F NMR (565 MHz, $CDCl_3$)

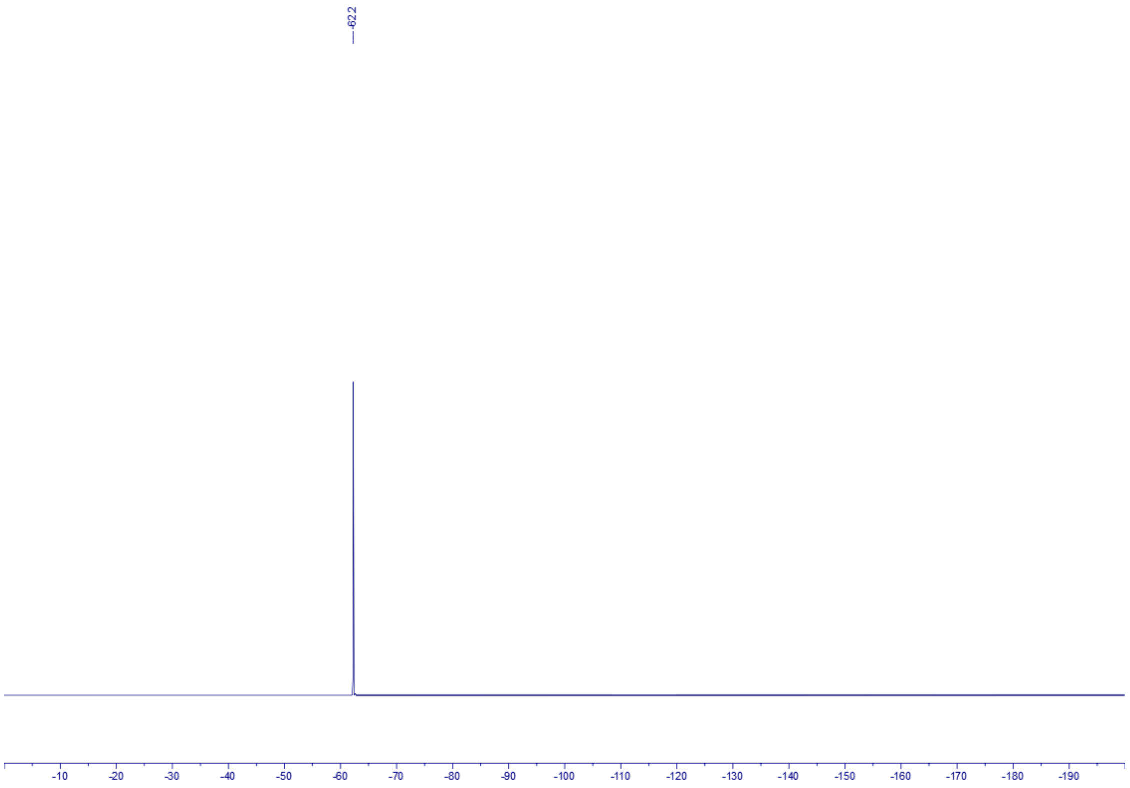

4-Phenyl-1-(*o*-tolyl)-1*H*-pyrazole (33a)

^{1}H NMR (600 MHz, $CDCl_3$)

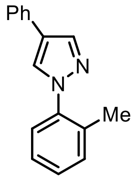

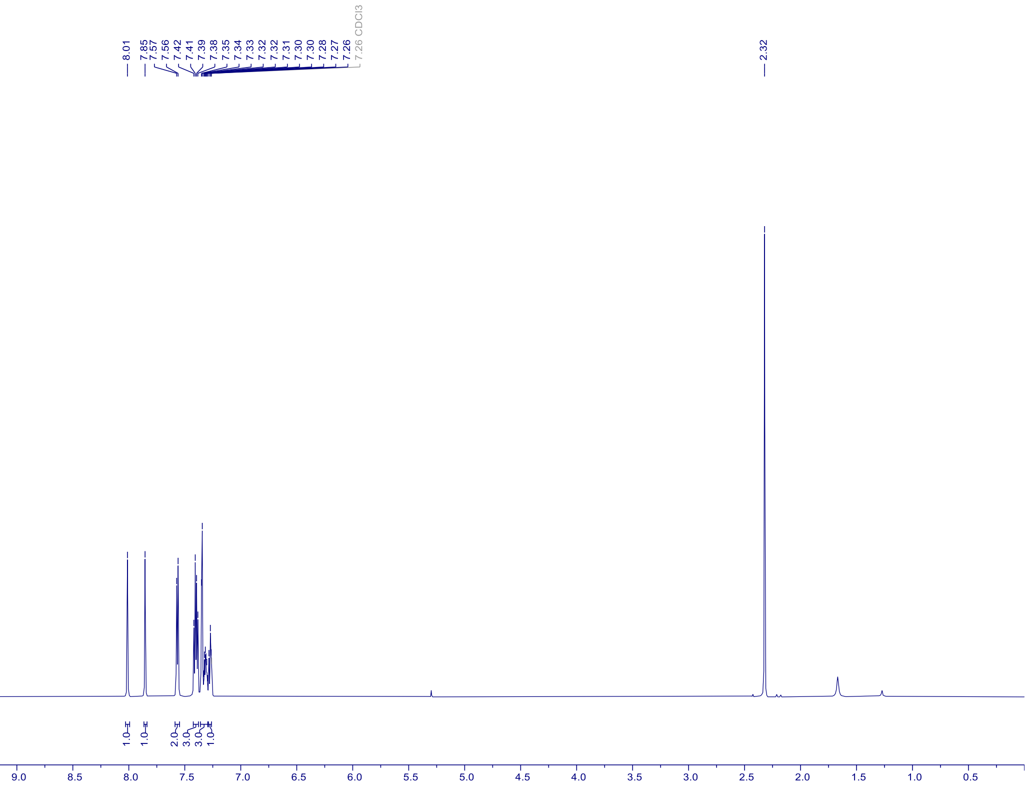

^{13}C NMR (151 MHz, $CDCl_3$)

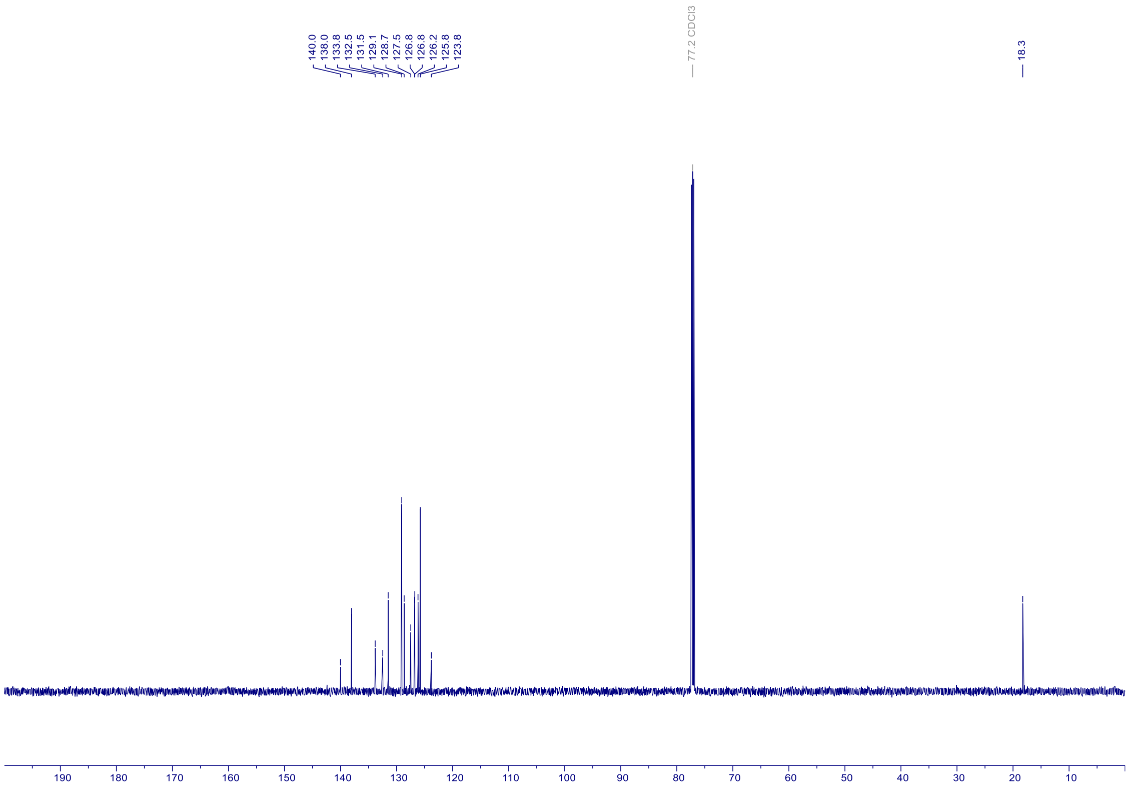

4-Phenyl-1-vinyl-1*H*-pyrazole (34a)

^{1}H NMR (600 MHz, $CDCl_3$)

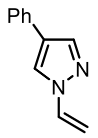

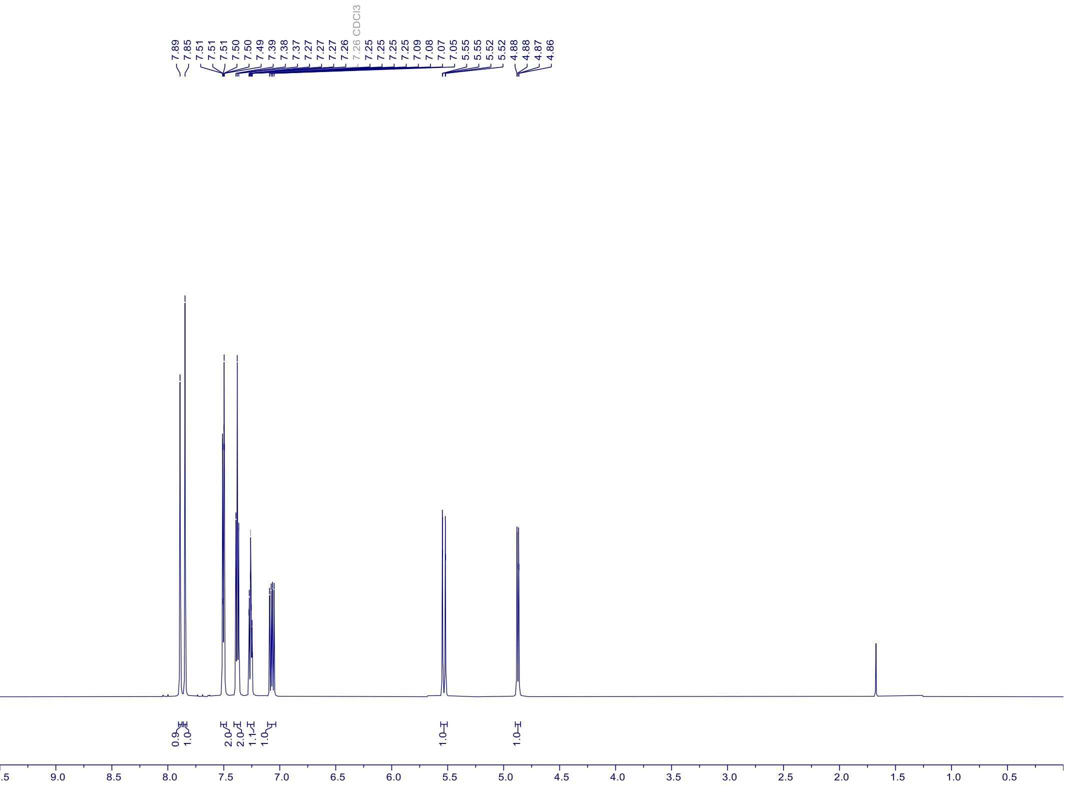

^{13}C NMR (151 MHz, $CDCl_3$)

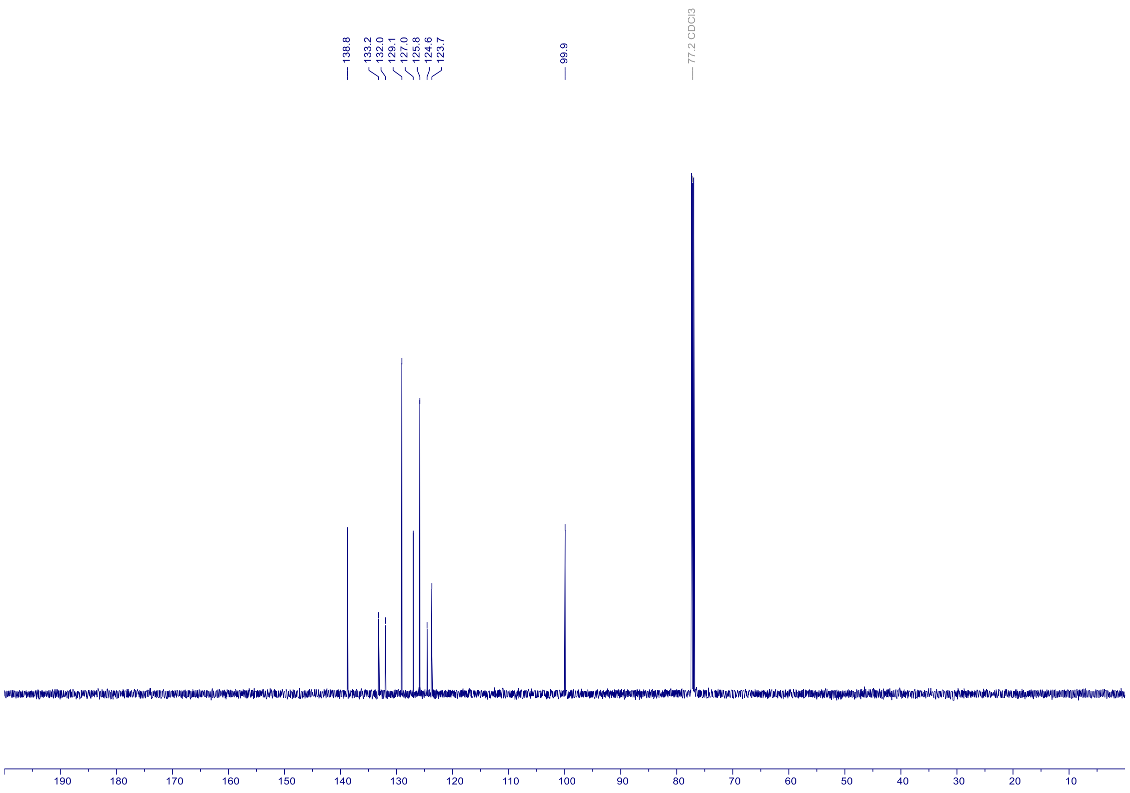

4-Phenyl-1-((triisopropylsilyl)ethynyl)-1*H*-pyrazole (35a)

^{1}H NMR (600 MHz, $CDCl_3$)

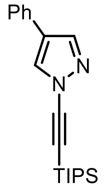

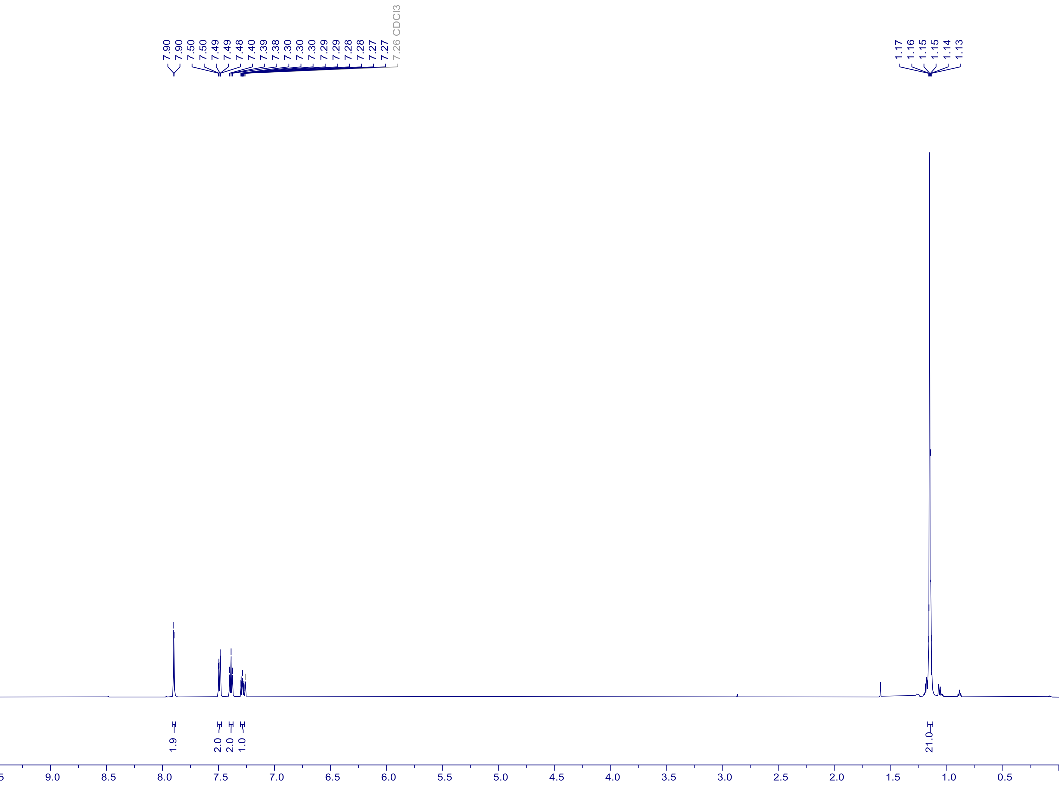

^{13}C NMR (151 MHz, $CDCl_3$)

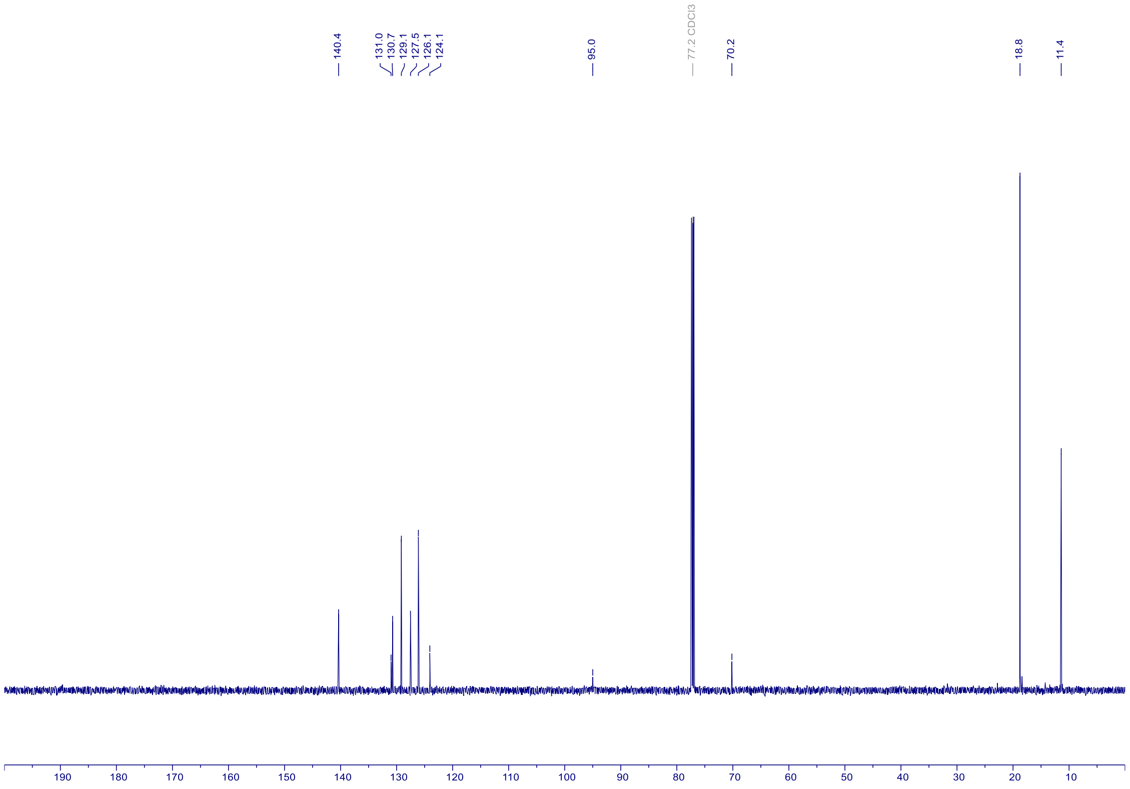

_N,N_-dimethyl-1-phenyl-1_H_-pyrazole-4-carboxamide (42a)

^{1}H NMR (600 MHz, $CDCl_3$)

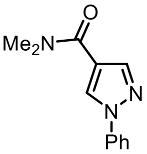

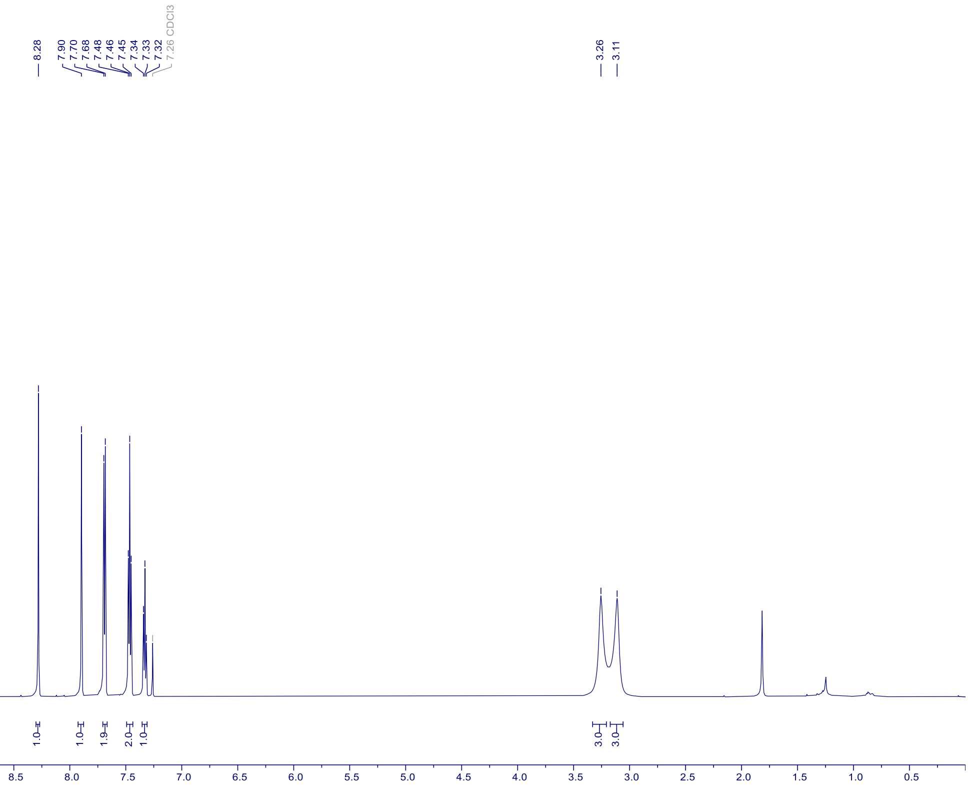

^{13}C NMR (151 MHz, $CDCl_3$)

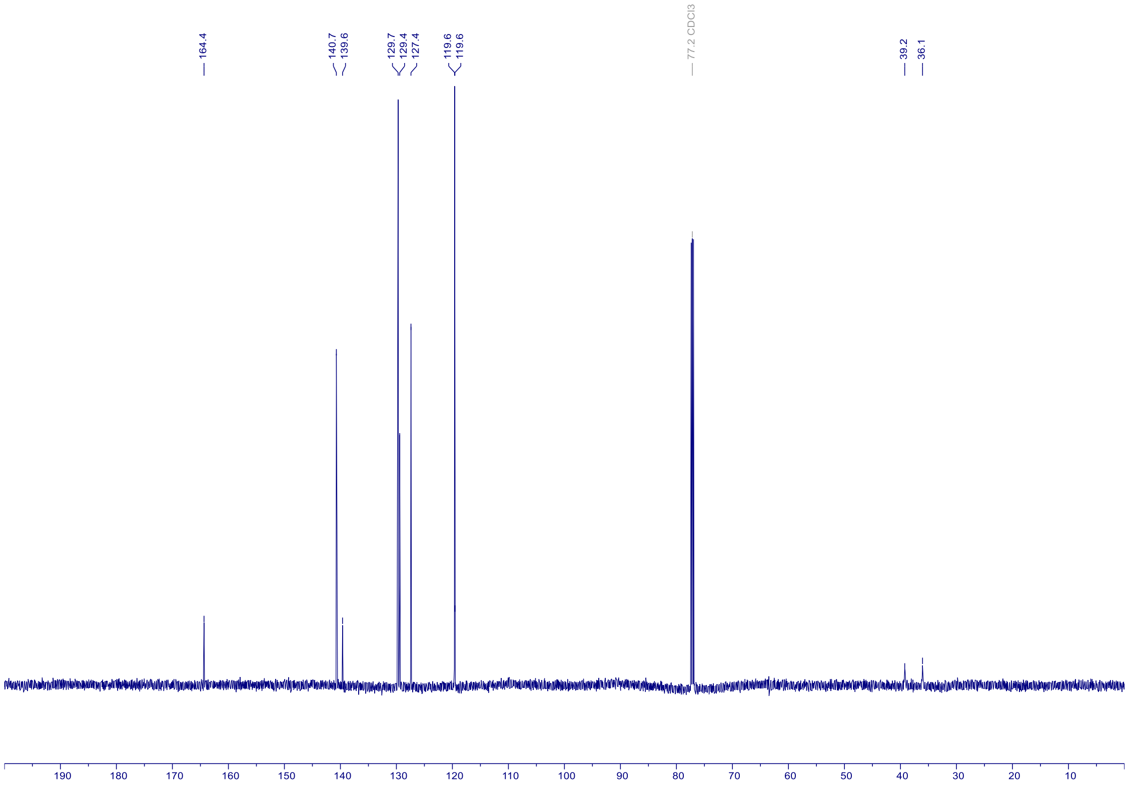

3,5-Dimethyl-1'-phenyl-1'*H*-1,4'-bipyrazole (46a)

^{1}H NMR (600 MHz, $CDCl_3$)

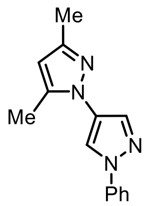

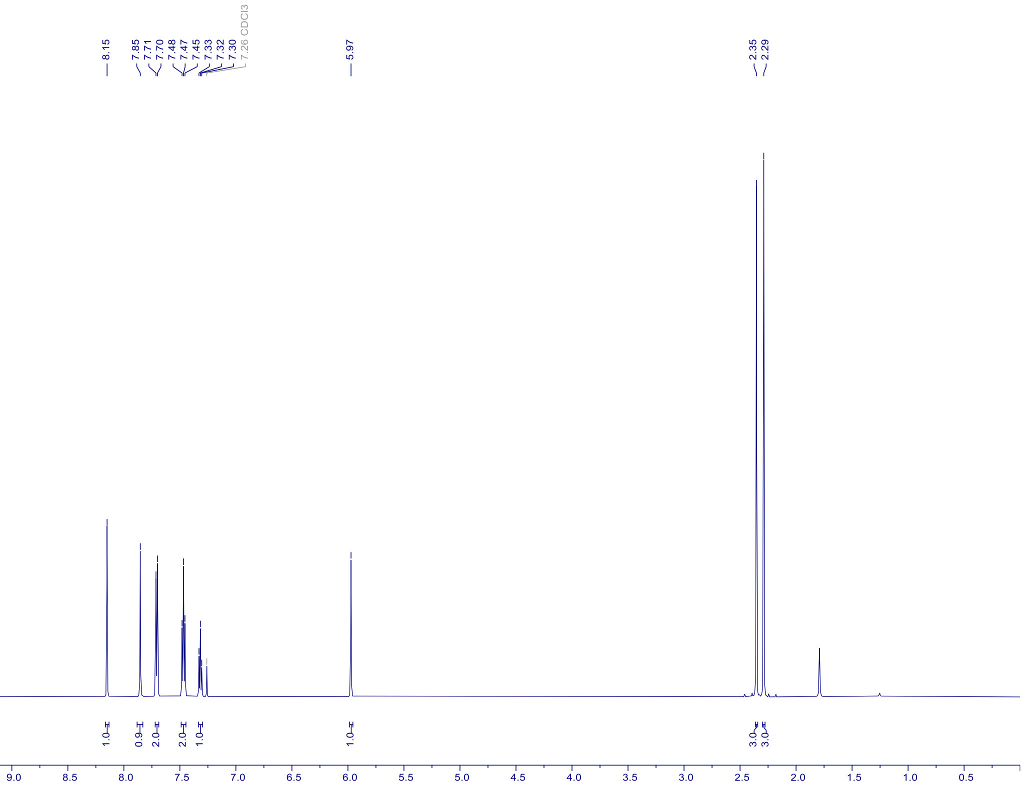

^{13}C NMR (151 MHz, $CDCl_3$)

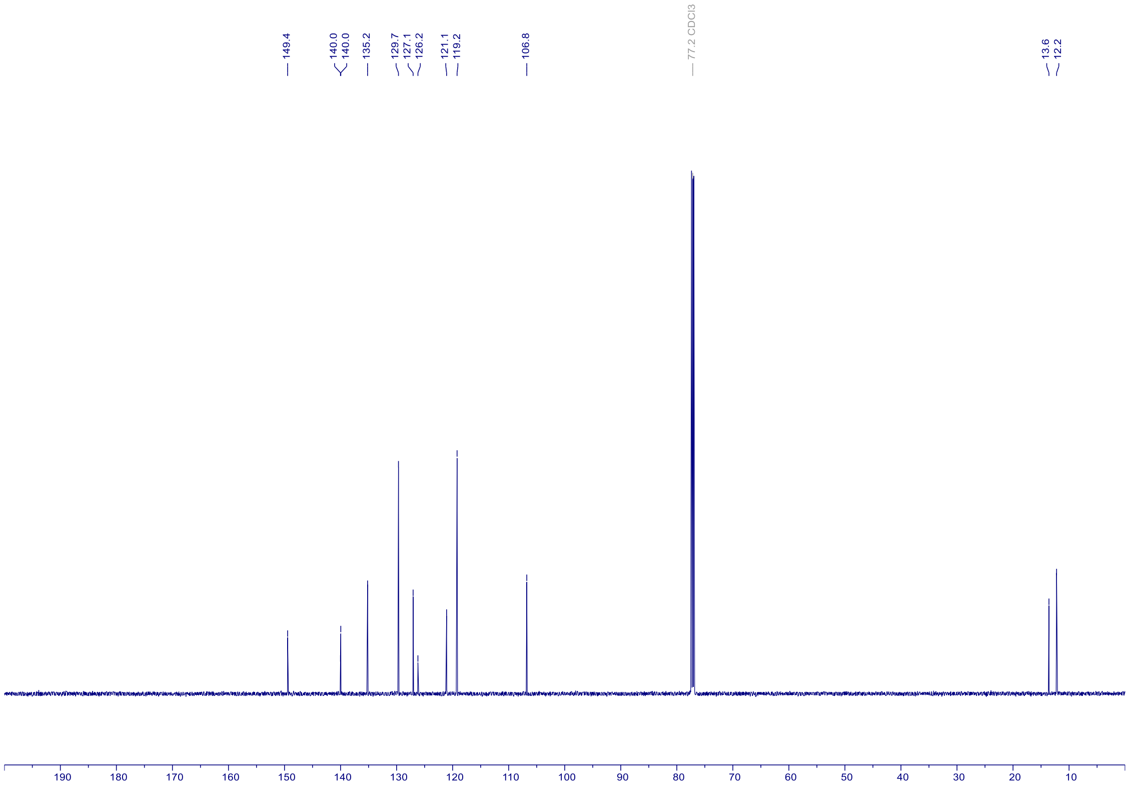

3-Methyl-1,4-diphenyl-1*H*-pyrazole (50a)

^{1}H NMR (600 MHz, $CDCl_3$)

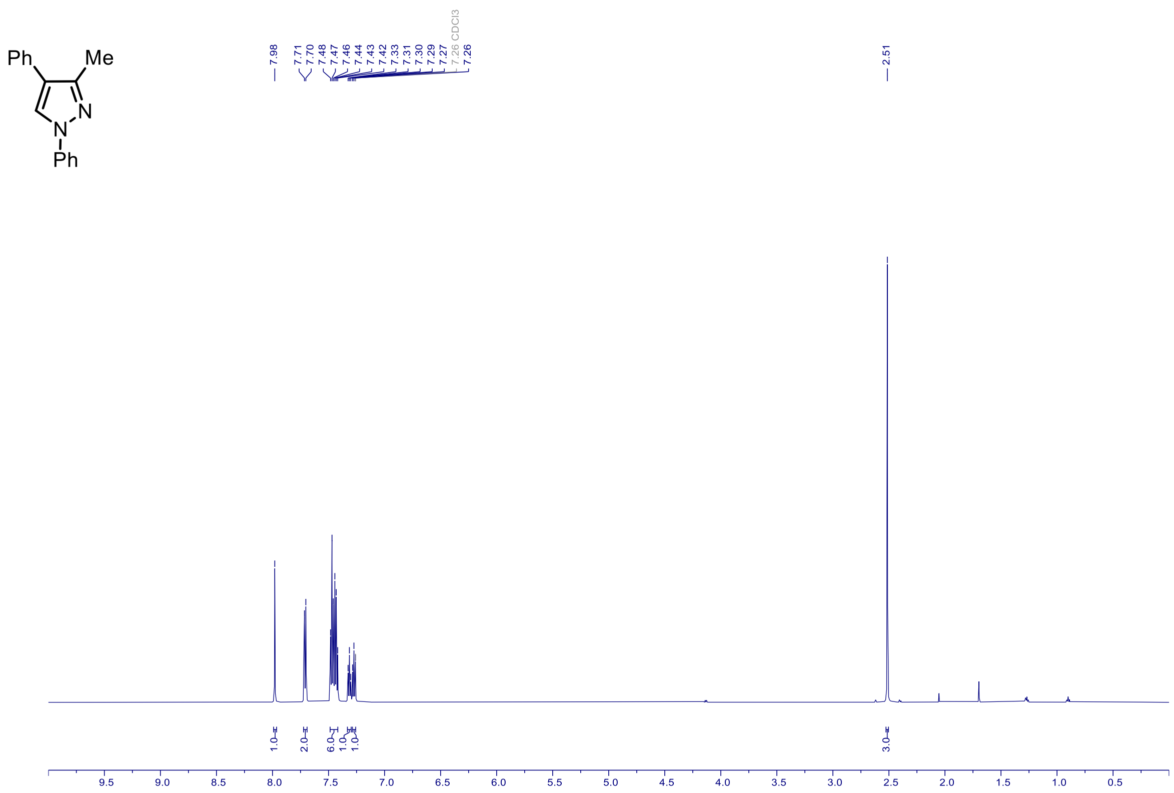

^{13}C NMR (151 MHz, $CDCl_3$)

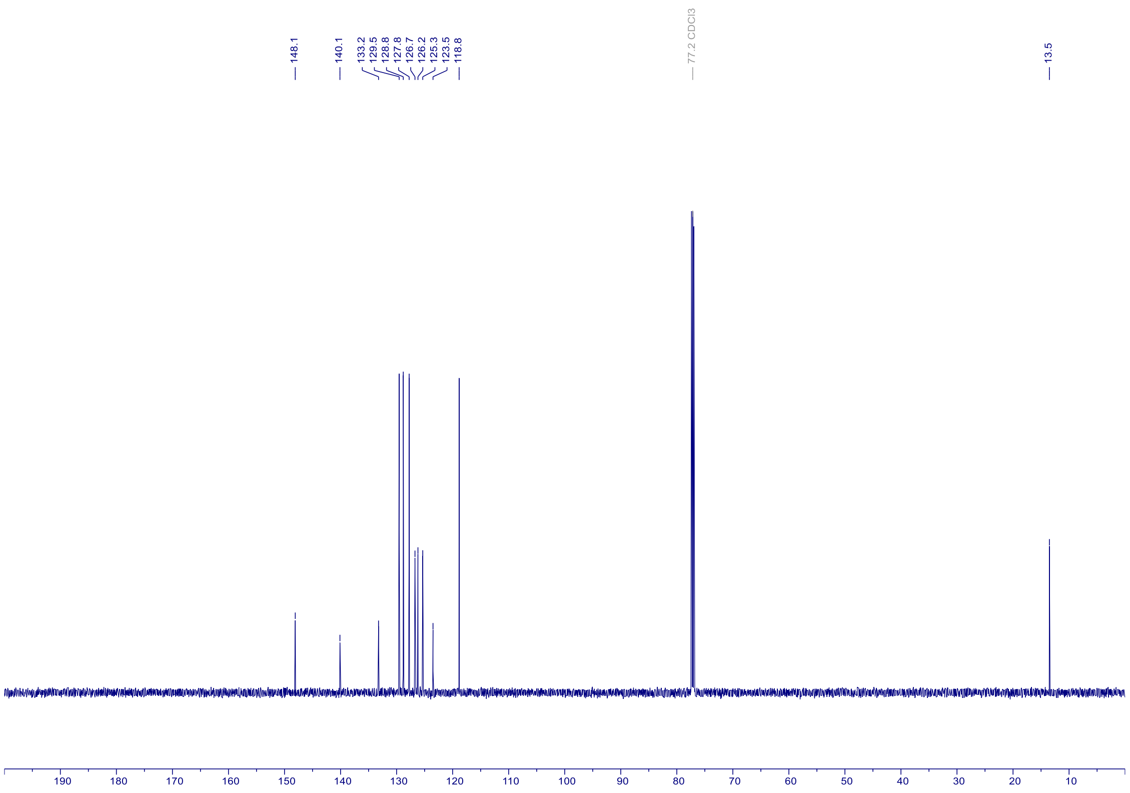

Methyl 5-Methyl-1-phenyl-1H-pyrazole-4-carboxylate (53a)

^{1}H NMR (600 MHz, $CDCl_3$)

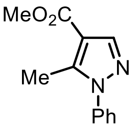

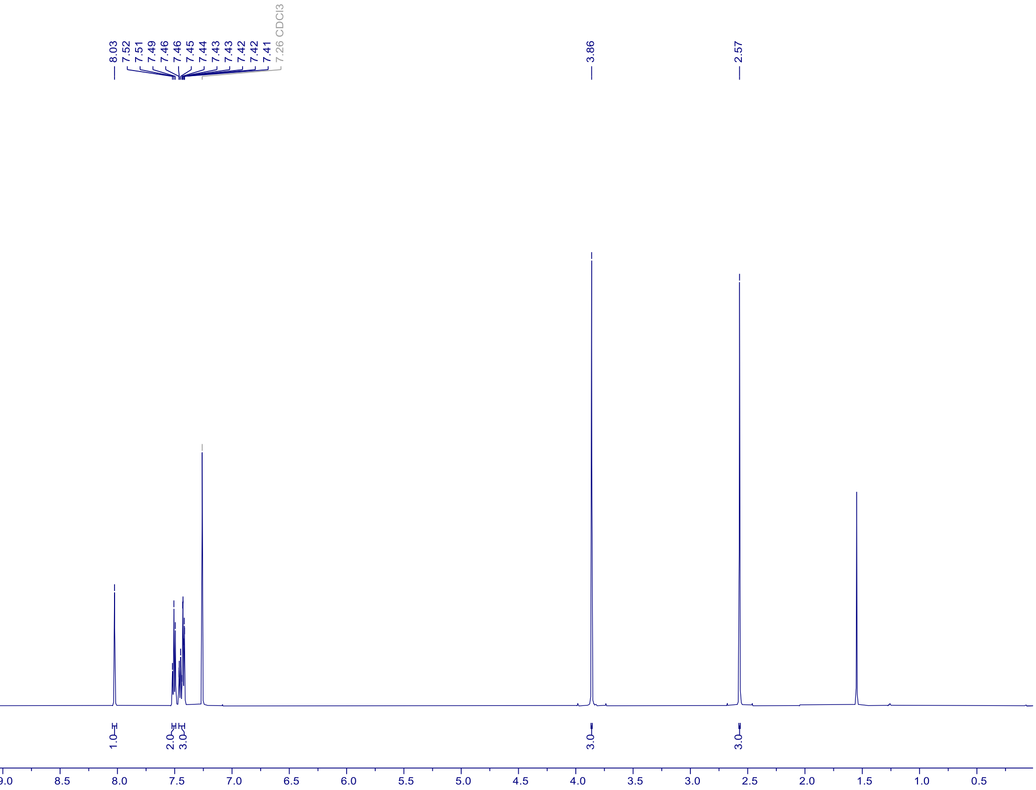

^{13}C NMR (151 MHz, $CDCl_3$)

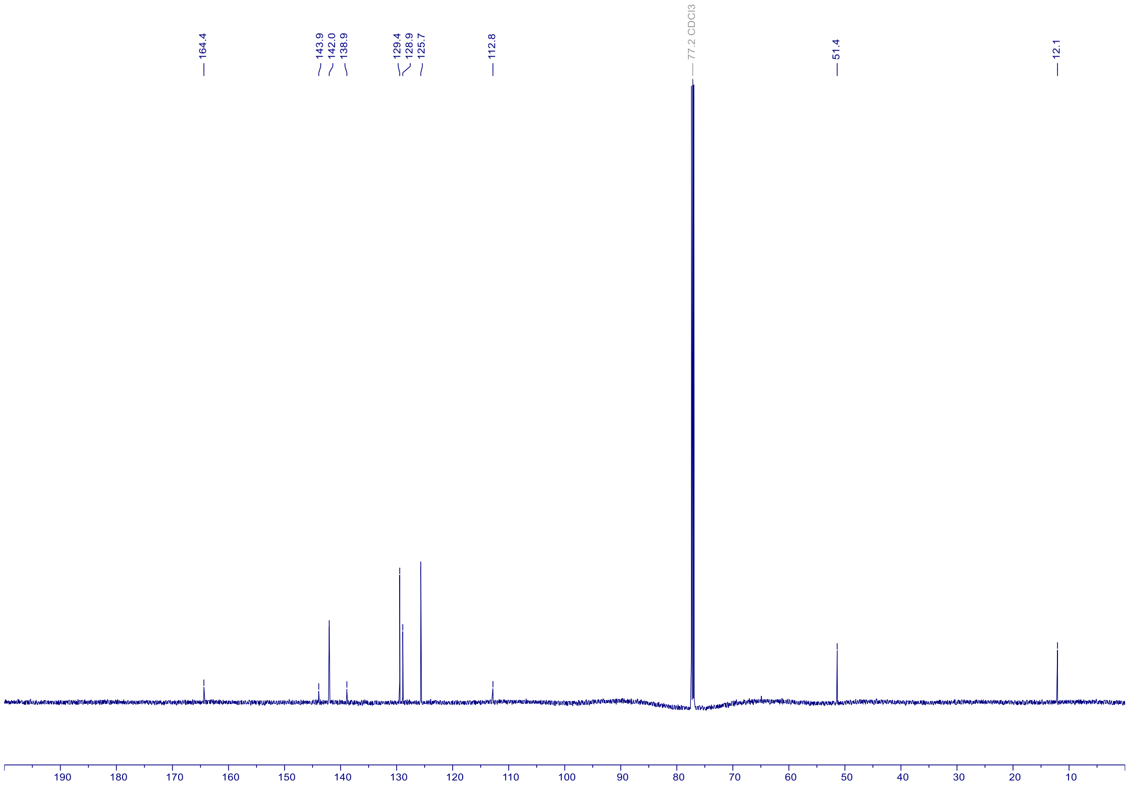

***tert*-Butyl 1-Phenyl-1,4,6,7-tetrahydro-5*H*-pyrazolo[4,3-*c*]pyridine-5-carboxylate (64a)**

^{1}H NMR (600 MHz, $CDCl_3$)

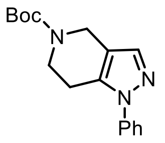

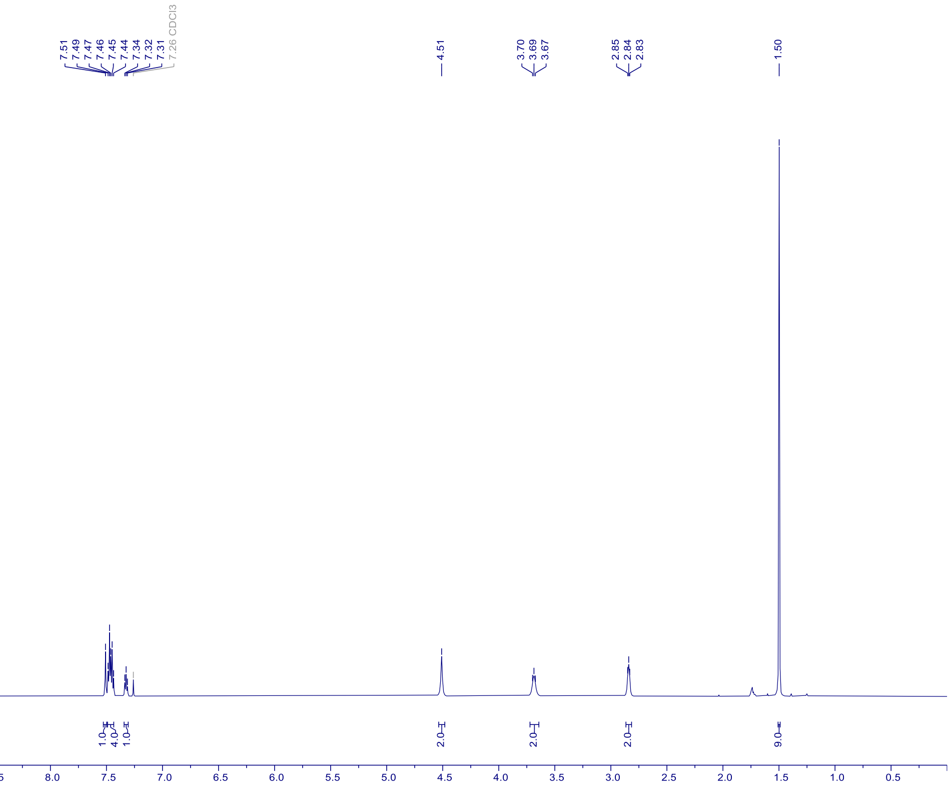

^{13}C NMR (151 MHz, $CDCl_3$)

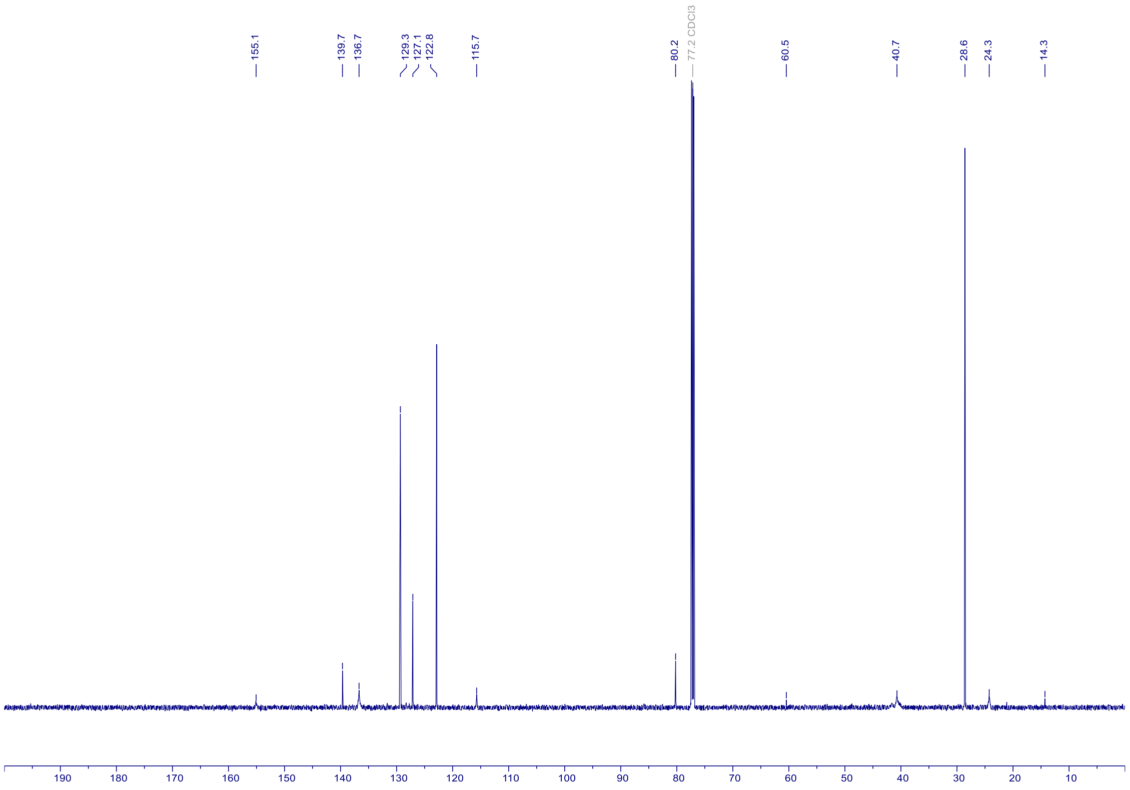

tert-Butyl 1-Phenyl-4,6-dihydropyrrolo[3,4-_c_]pyrazole-5(1_H_)-carboxylate (65a)

^{1}H NMR (600 MHz, $CDCl_3$)

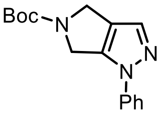

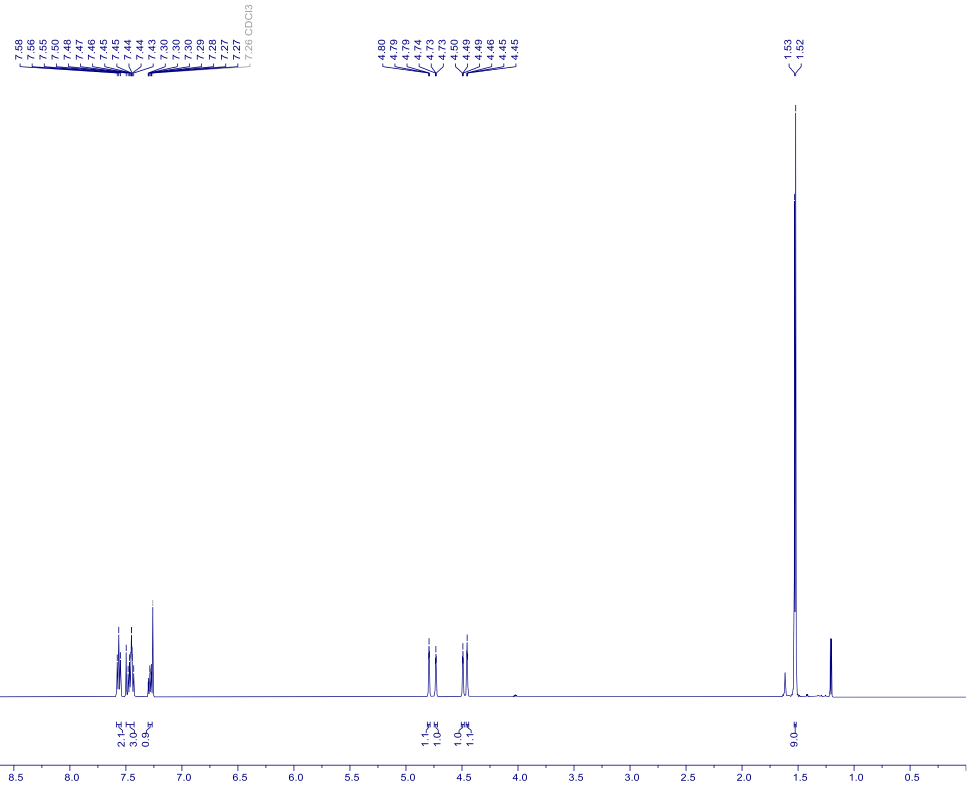

^{13}C NMR (151 MHz, $CDCl_3$)

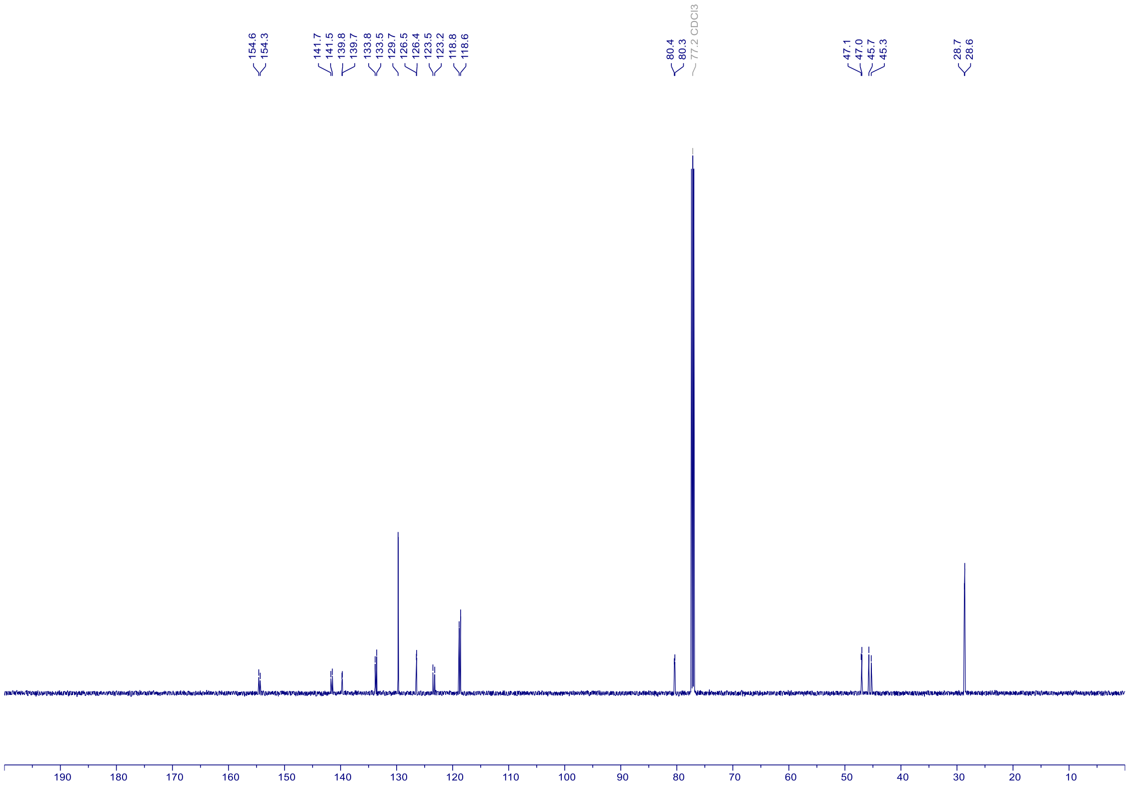

1-Phenyl-1,4,6,7-tetrahydropyrano[4,3-*c*]pyrazole (66a)

^{1}H NMR (600 MHz, $CDCl_3$)

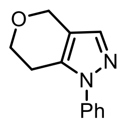

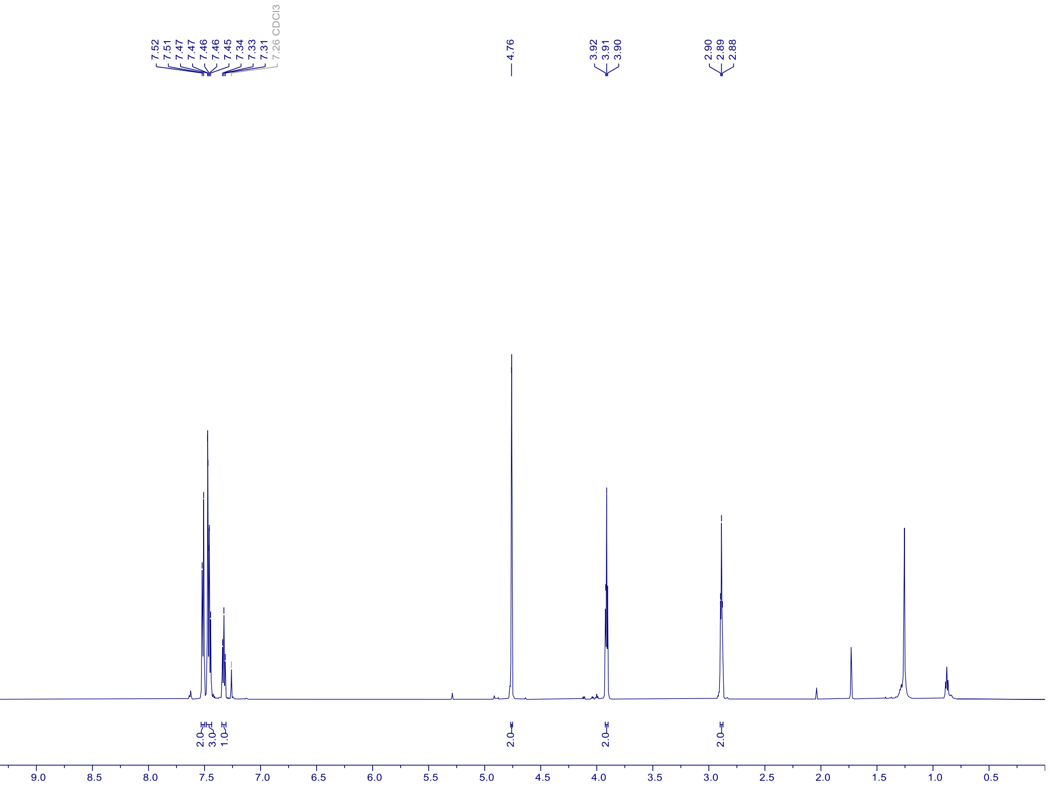

^{13}C NMR (151 MHz, $CDCl_3$)

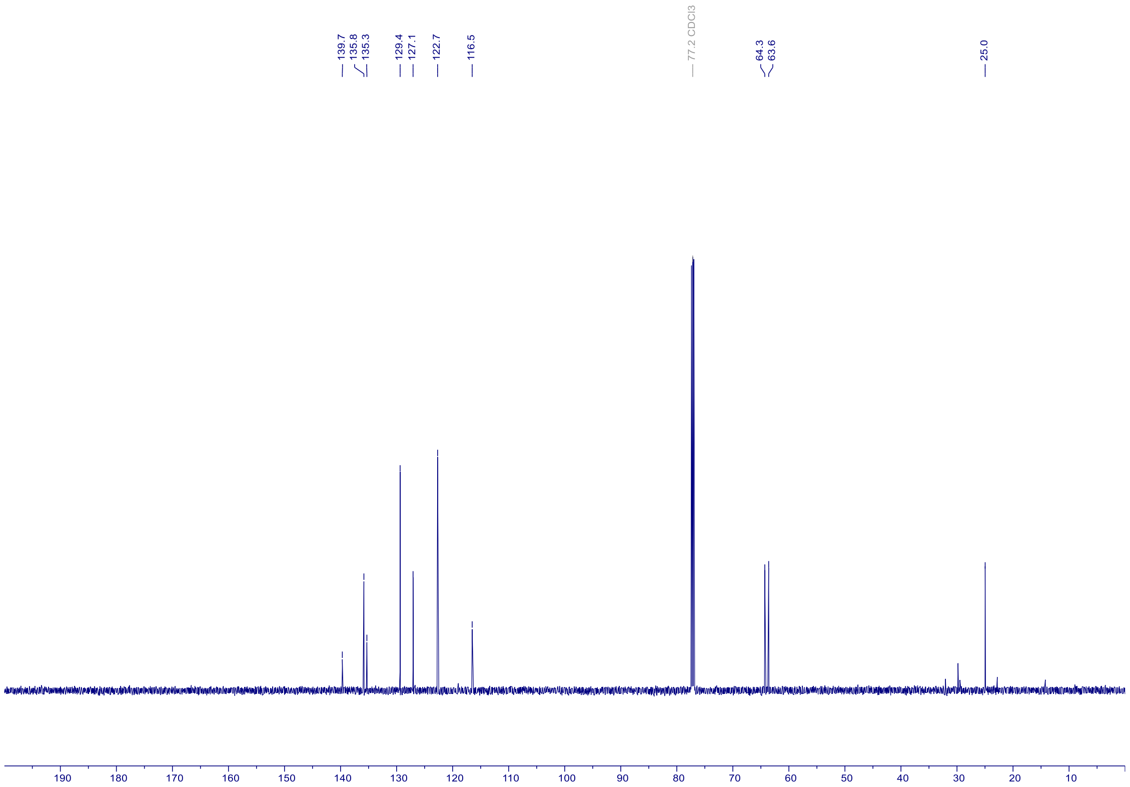

1-Phenyl-4,6-dihydro-1*H*-furo[3,4-*c*]pyrazole (67a)

^{1}H NMR (600 MHz, $CDCl_3$)

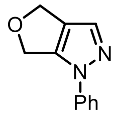

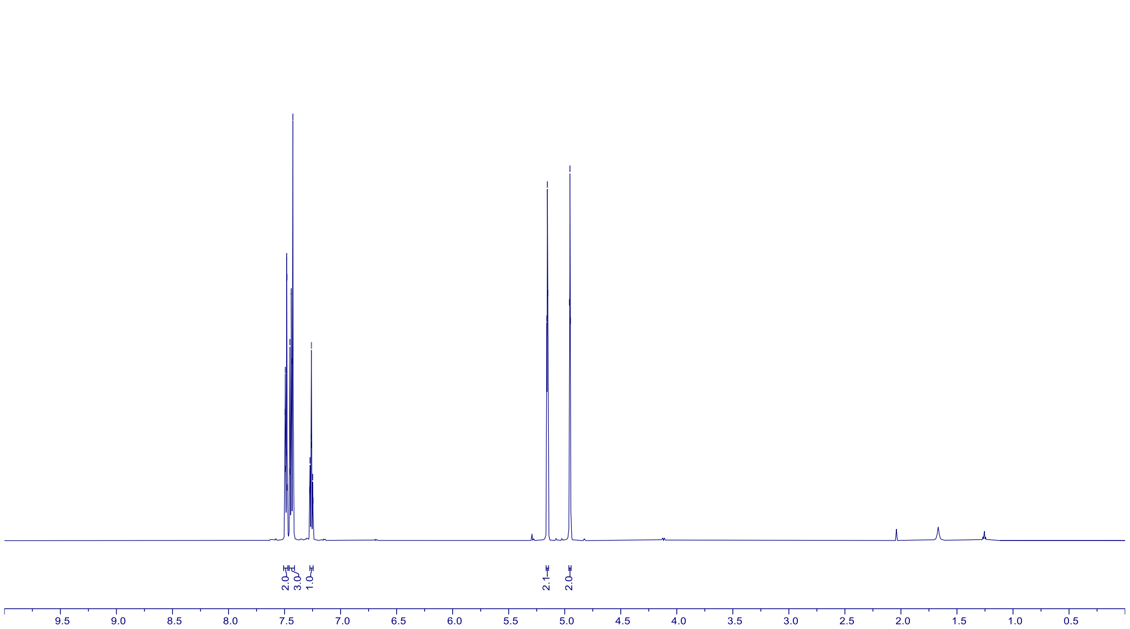

^{13}C NMR (151 MHz, $CDCl_3$)

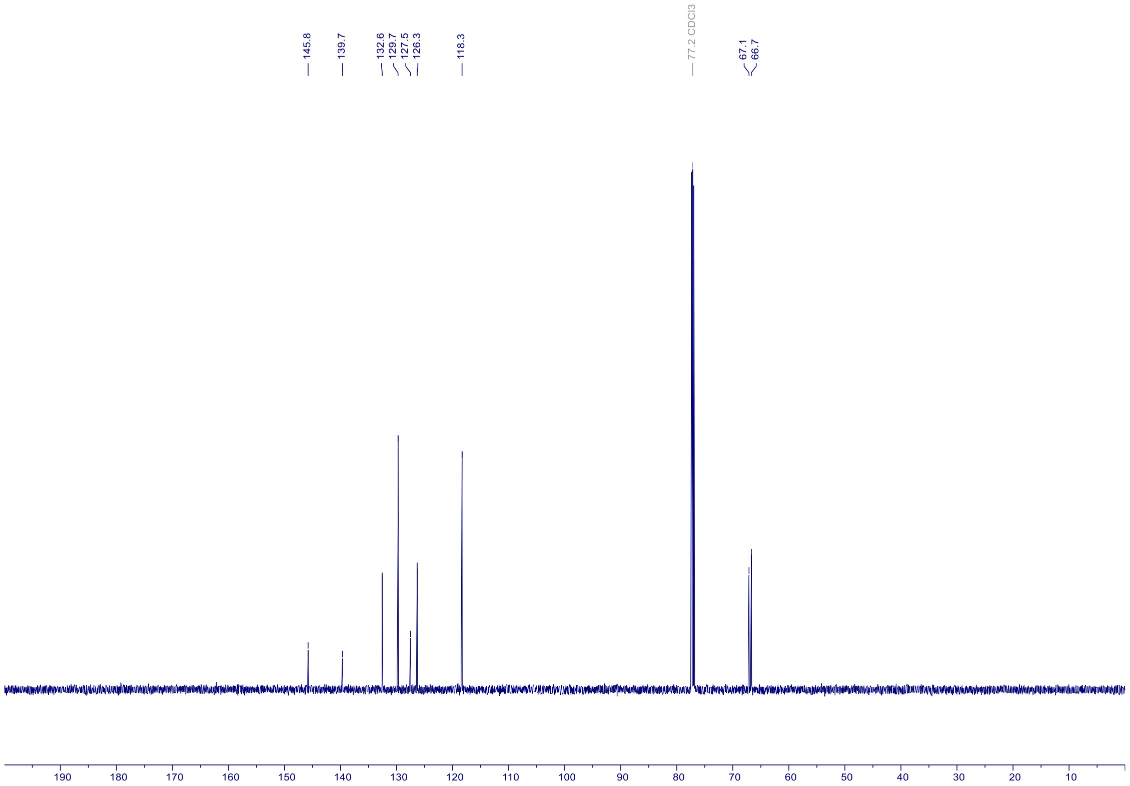

Ethyl 1-Phenyl-1,4,6,7-tetrahydropyrano[4,3-*c*]pyrazole-3-carboxylate (68a)

^{1}H NMR (600 MHz, $CDCl_3$)

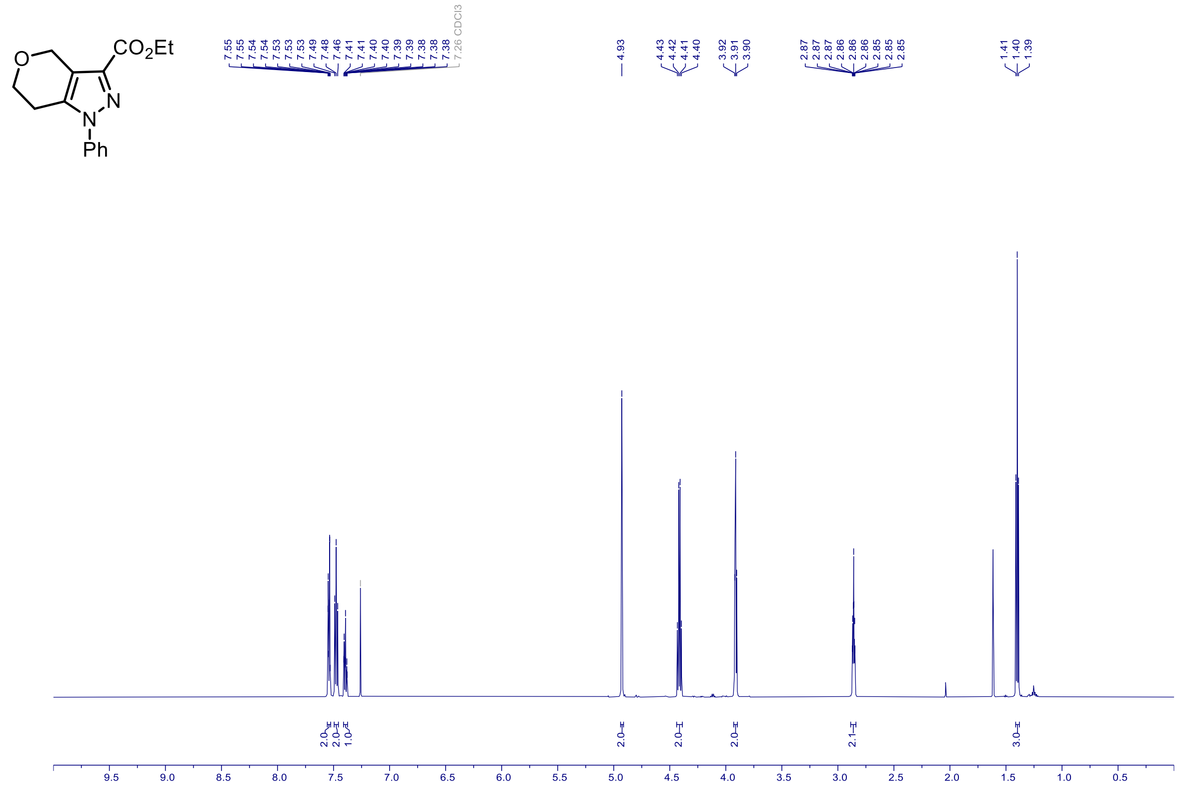

^{13}C NMR (151 MHz, $CDCl_3$)

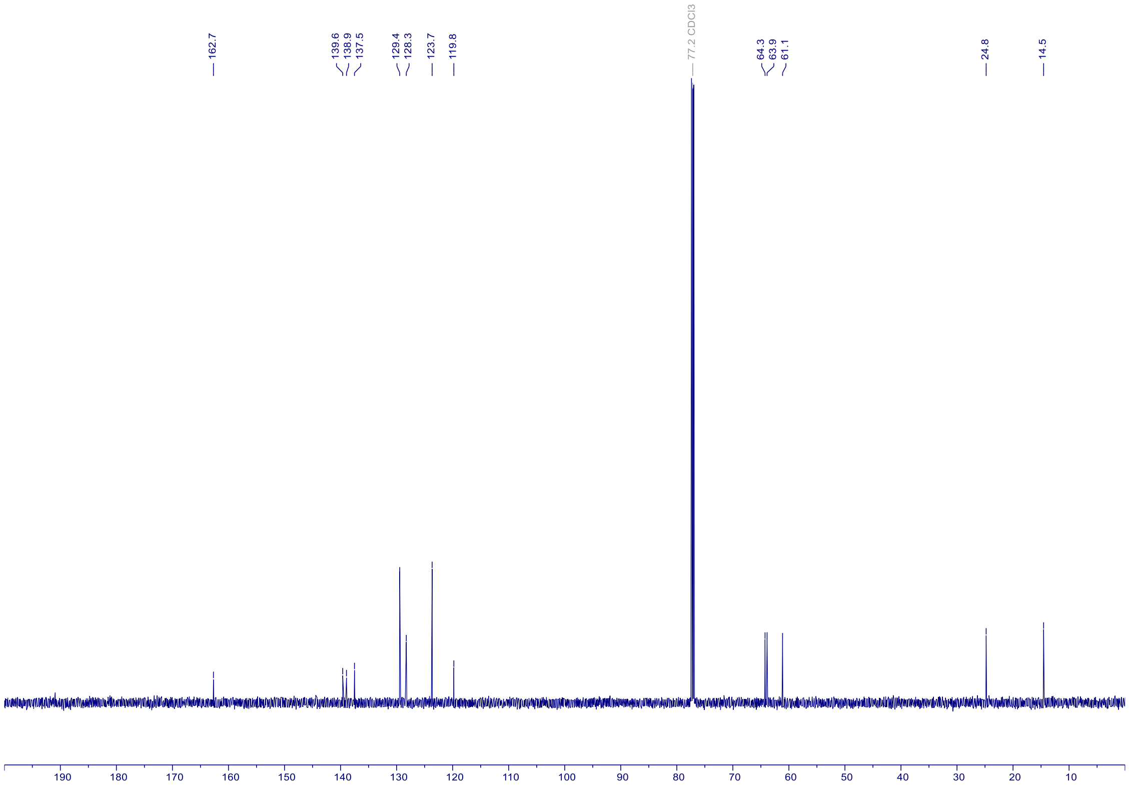

2-Phenyl-5,6-dihydro-4*H*-pyrrolo[1,2-*b*]pyrazole (71a)

^{1}H NMR (600 MHz, $CDCl_3$)

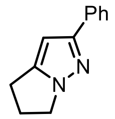

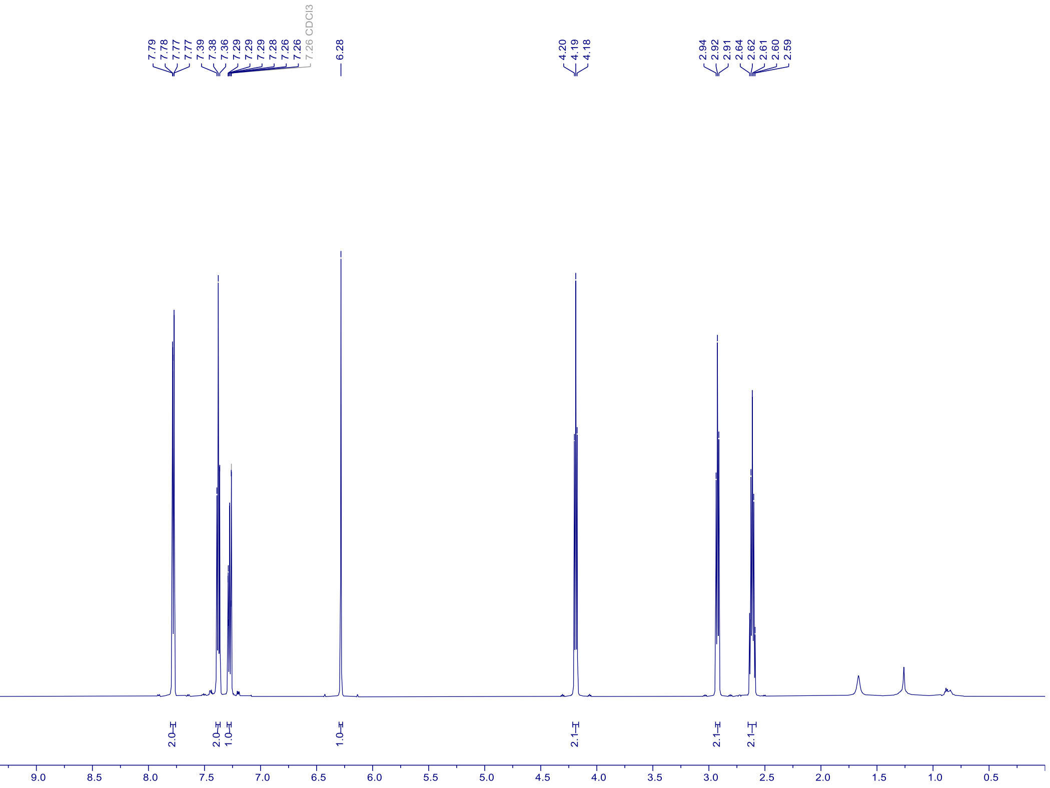

^{13}C NMR (151 MHz, $CDCl_3$)

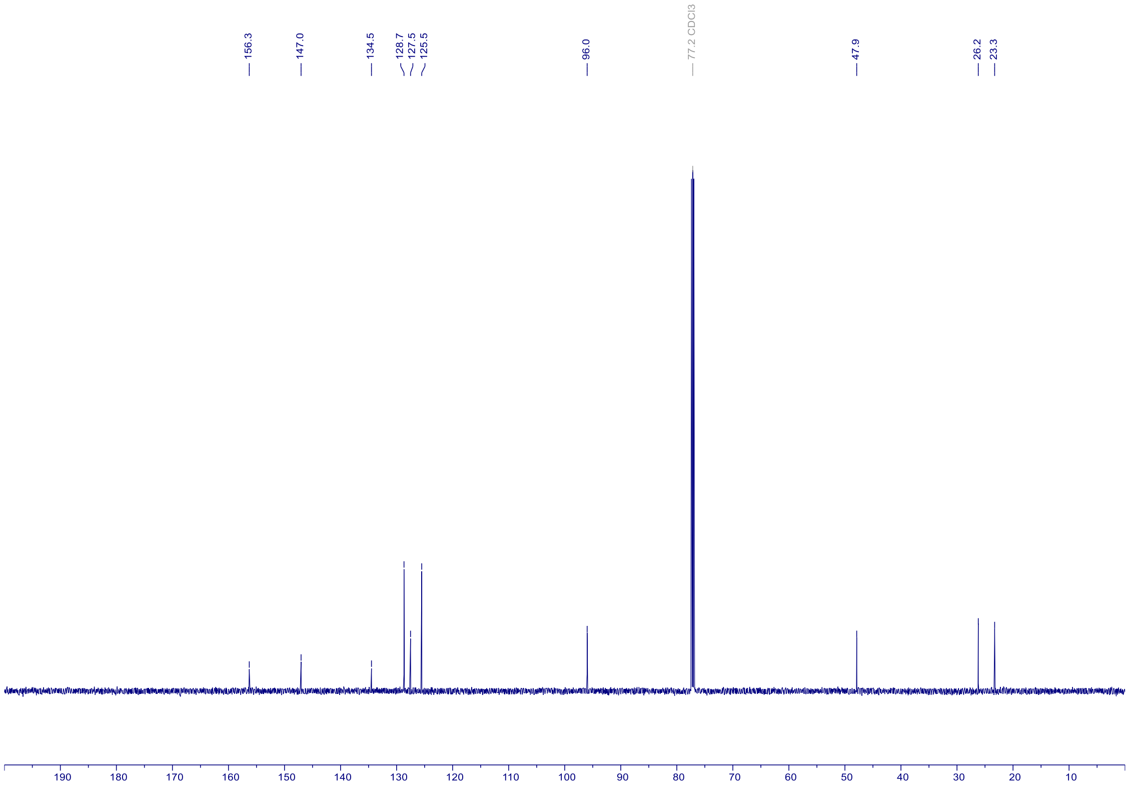

2-Methylpyrazolo[1,5-*a*]pyridine (73a)

^{1}H NMR (400 MHz, $CDCl_3$)

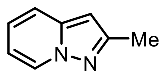

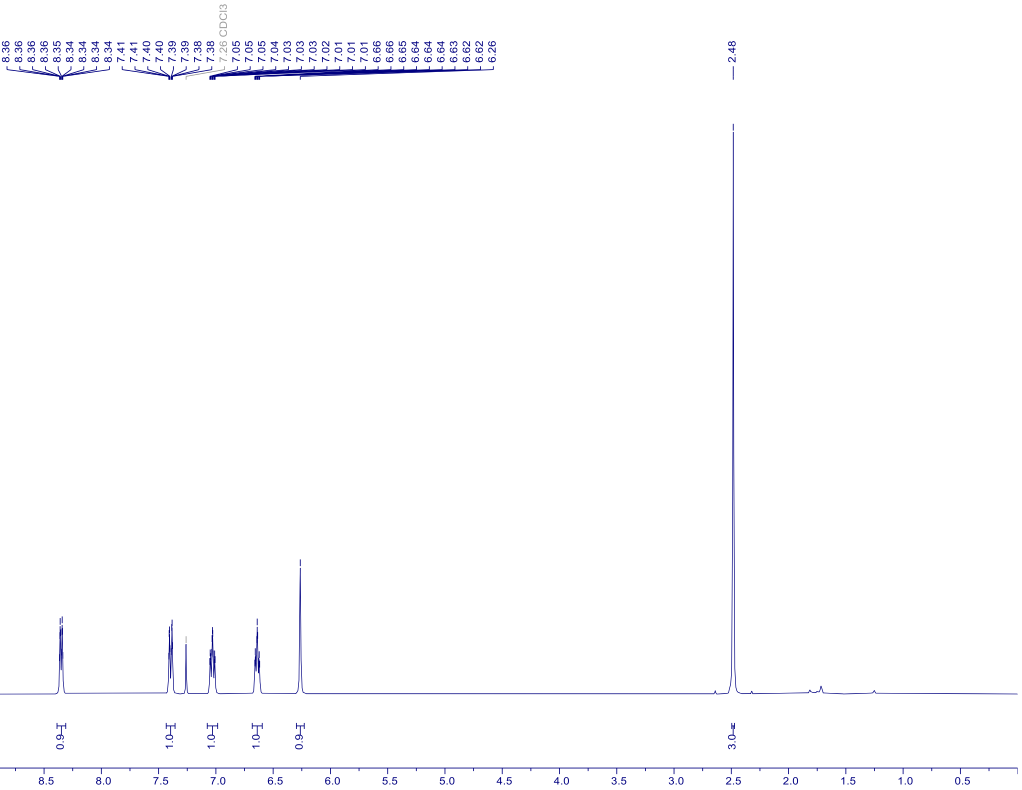

^{13}C NMR (100 MHz, $CDCl_3$)

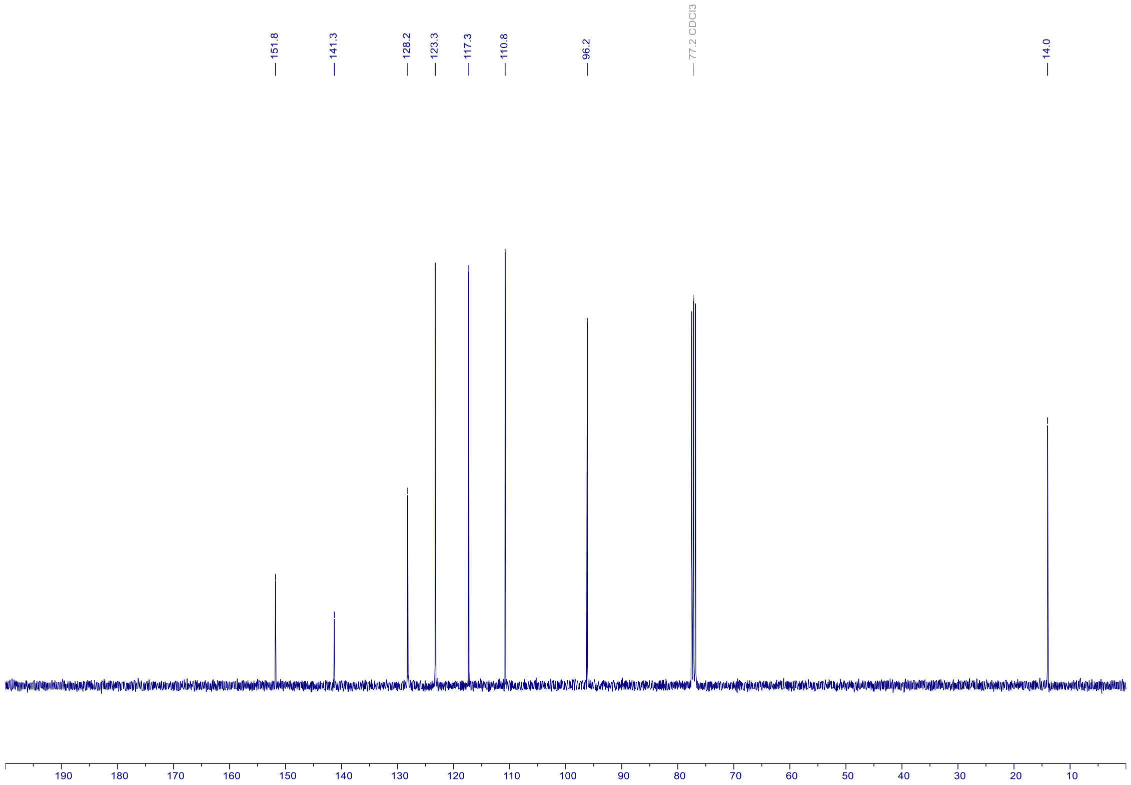

5-Methyl-1-phenyl-1H-imidazole (15b)

^{1}H NMR (600 MHz, $CDCl_3$)

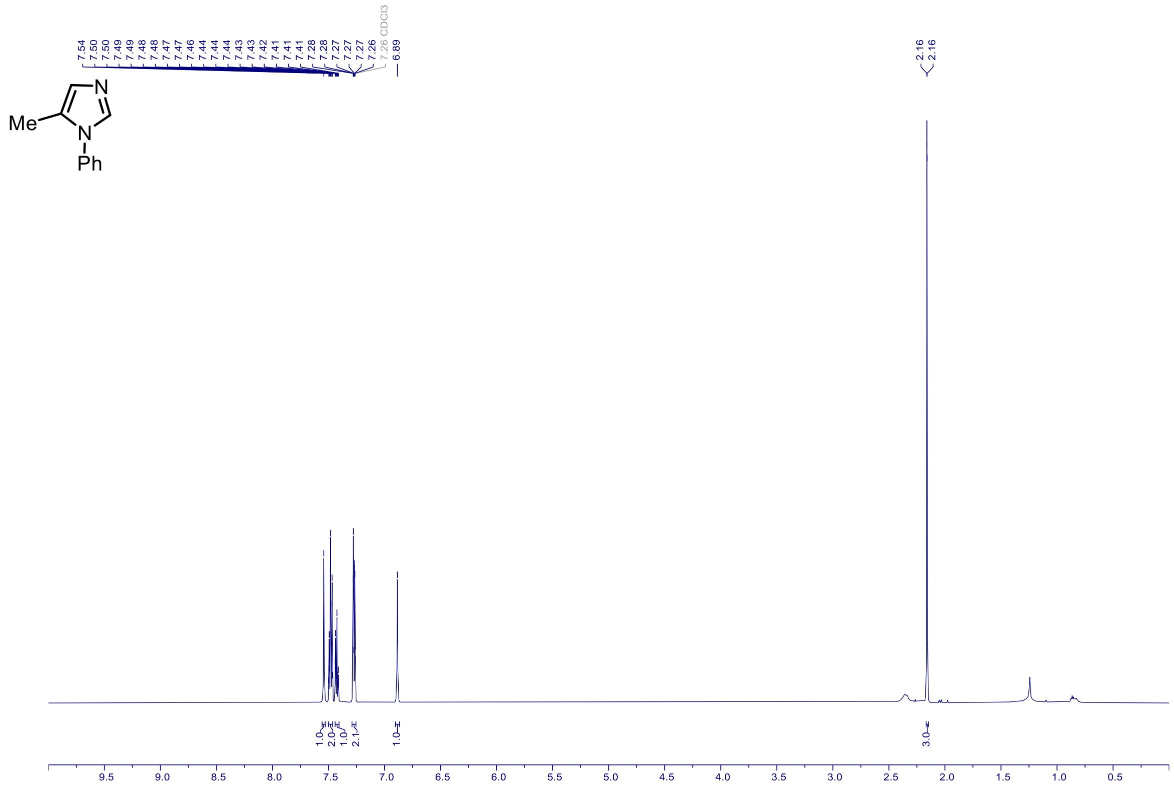

^{13}C NMR (151 MHz, $CDCl_3$)

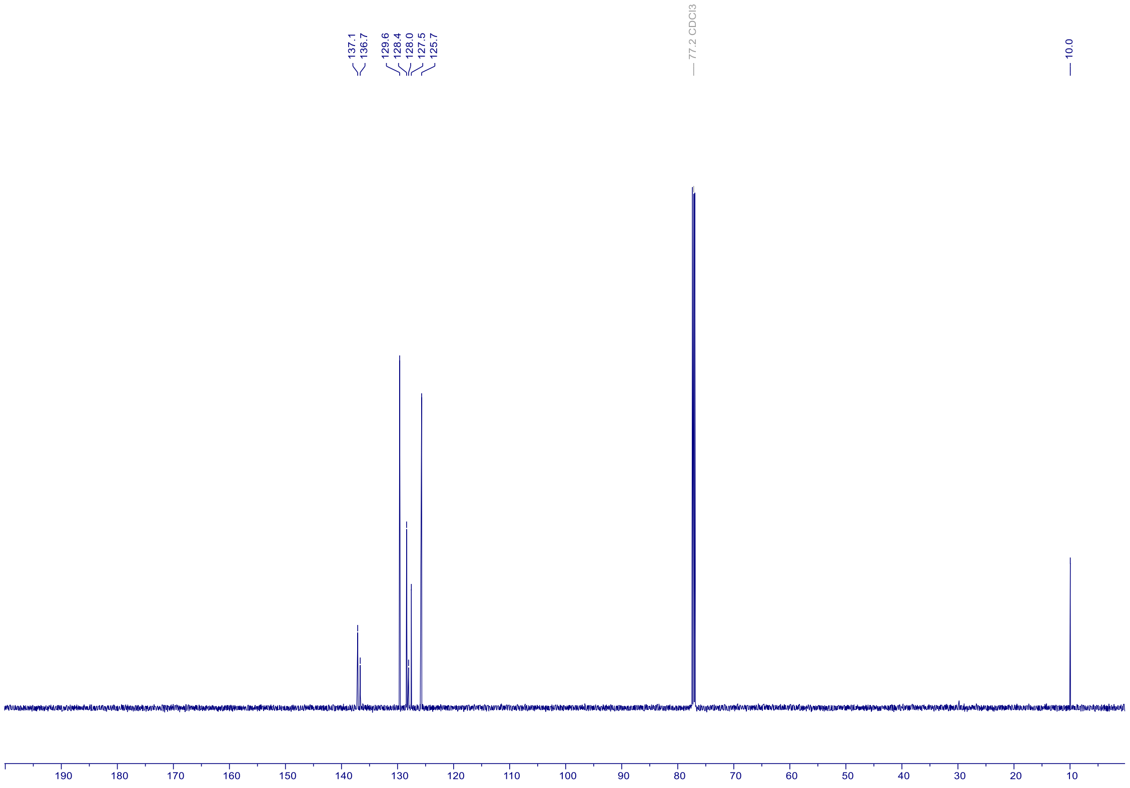

Methyl 1-Phenyl-1*H*-imidazole-2-carboxylate (16b)

^{1}H NMR (600 MHz, $CDCl_3$)

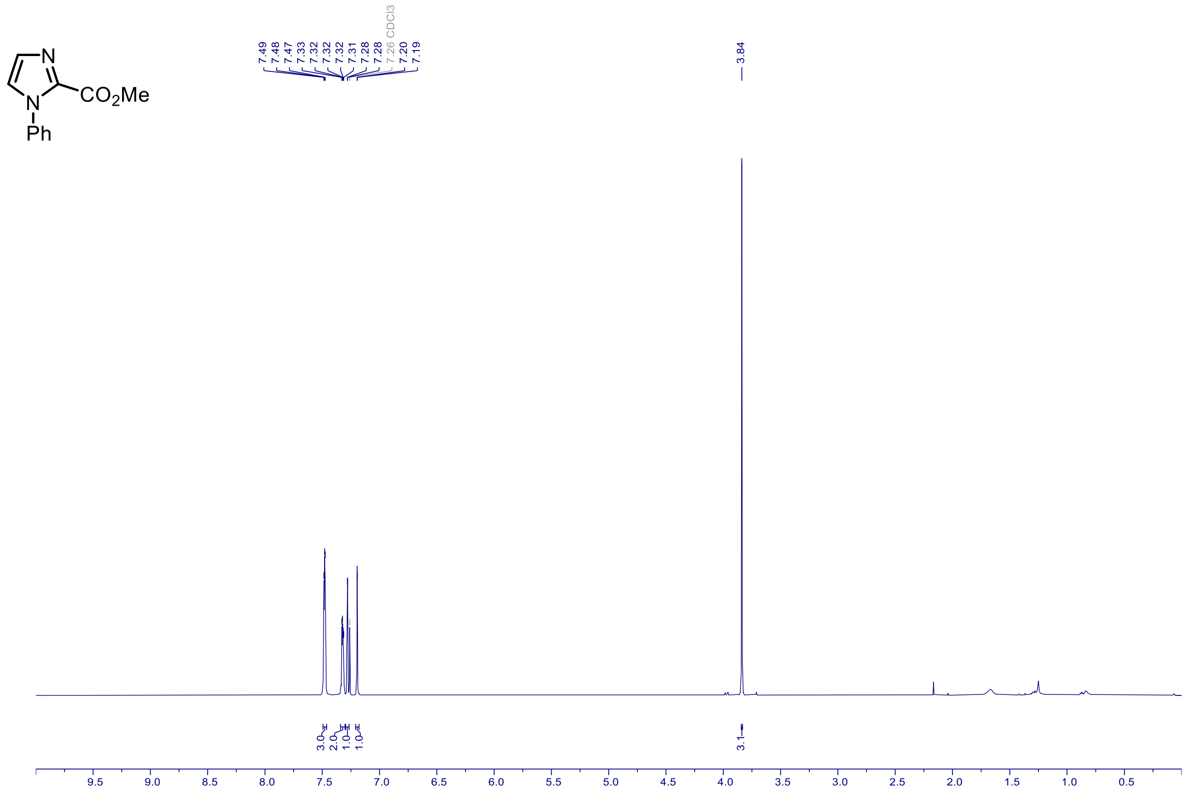

^{13}C NMR (151 MHz, $CDCl_3$)

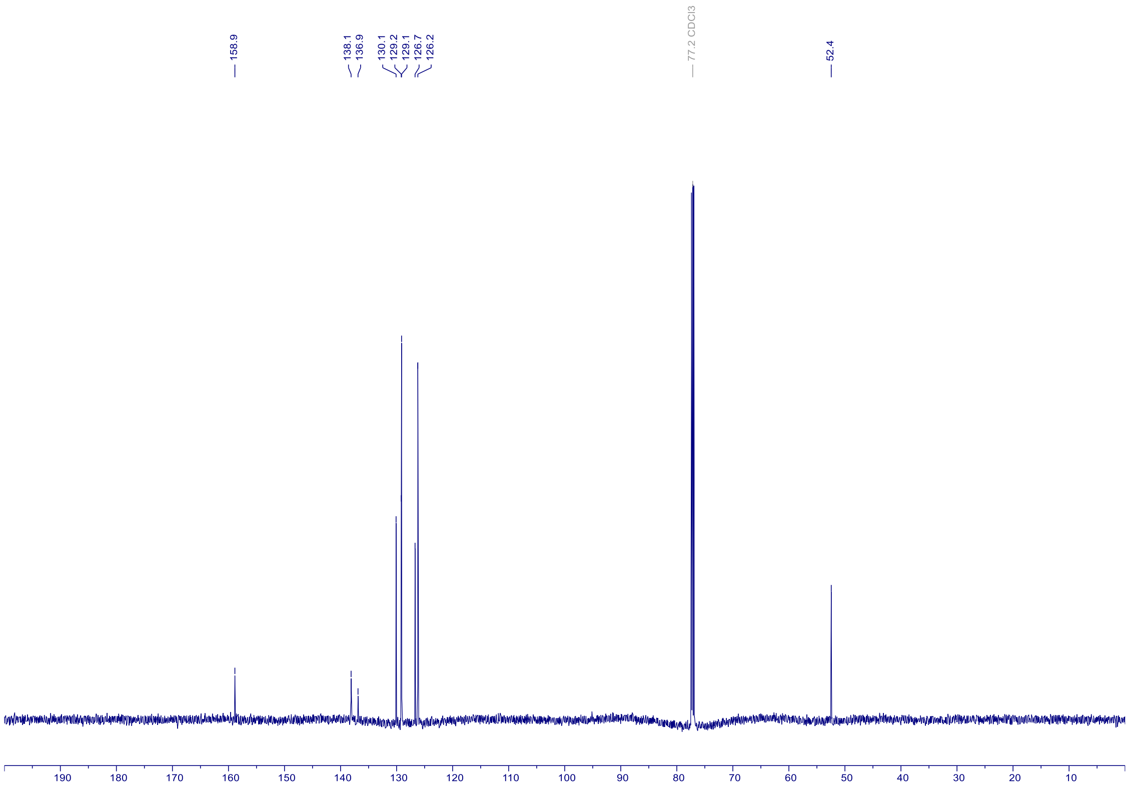

Methyl 1-Phenyl-1*H*-imidazole-4-carboxylate (17b)

^{1}H NMR (400 MHz, $CDCl_3$)

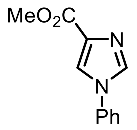

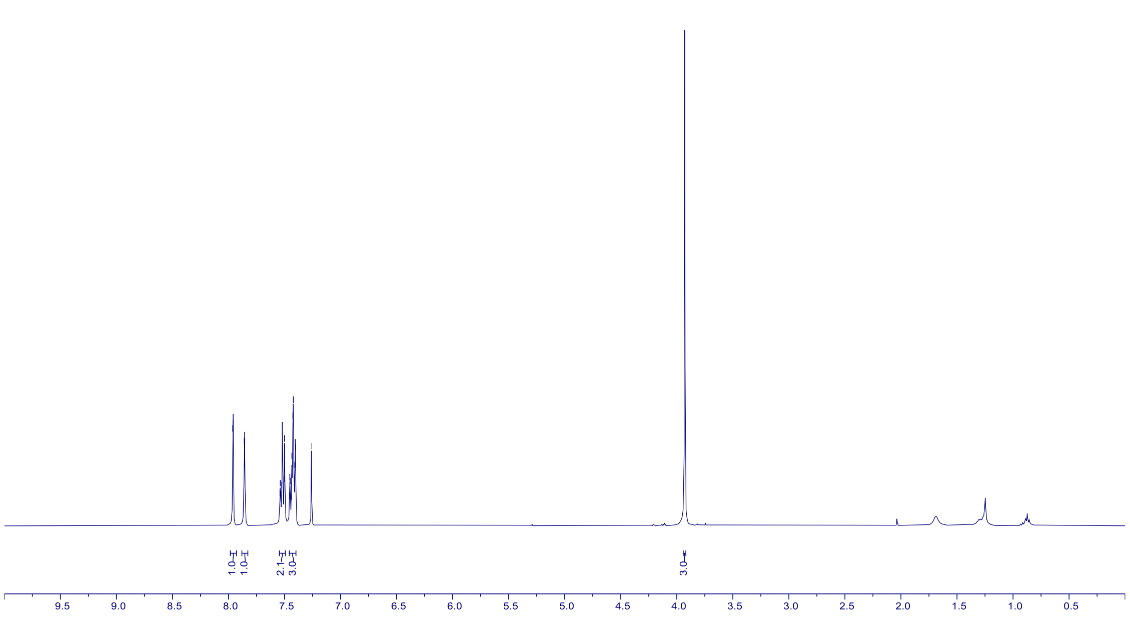

^{13}C NMR (101 MHz, $CDCl_3$)

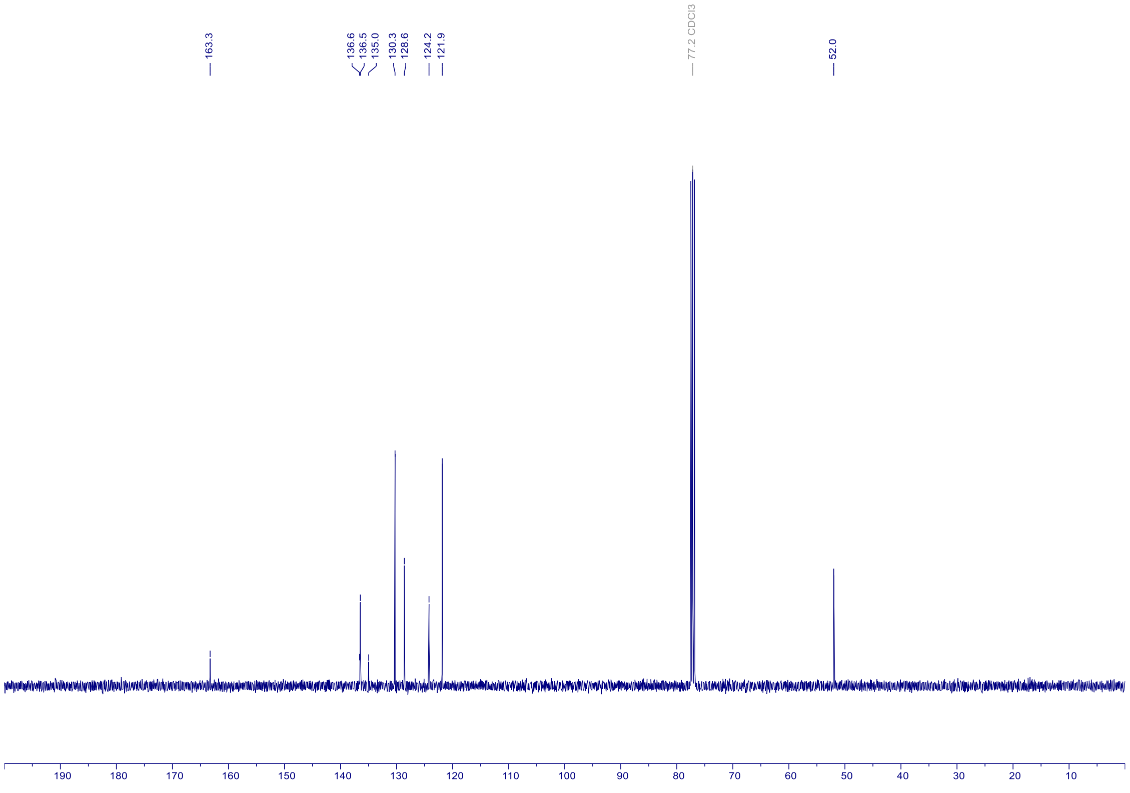

Methyl 1-Phenyl-1*H*-imidazole-5-carboxylate (18b)

^{1}H NMR (600 MHz, $CDCl_3$)

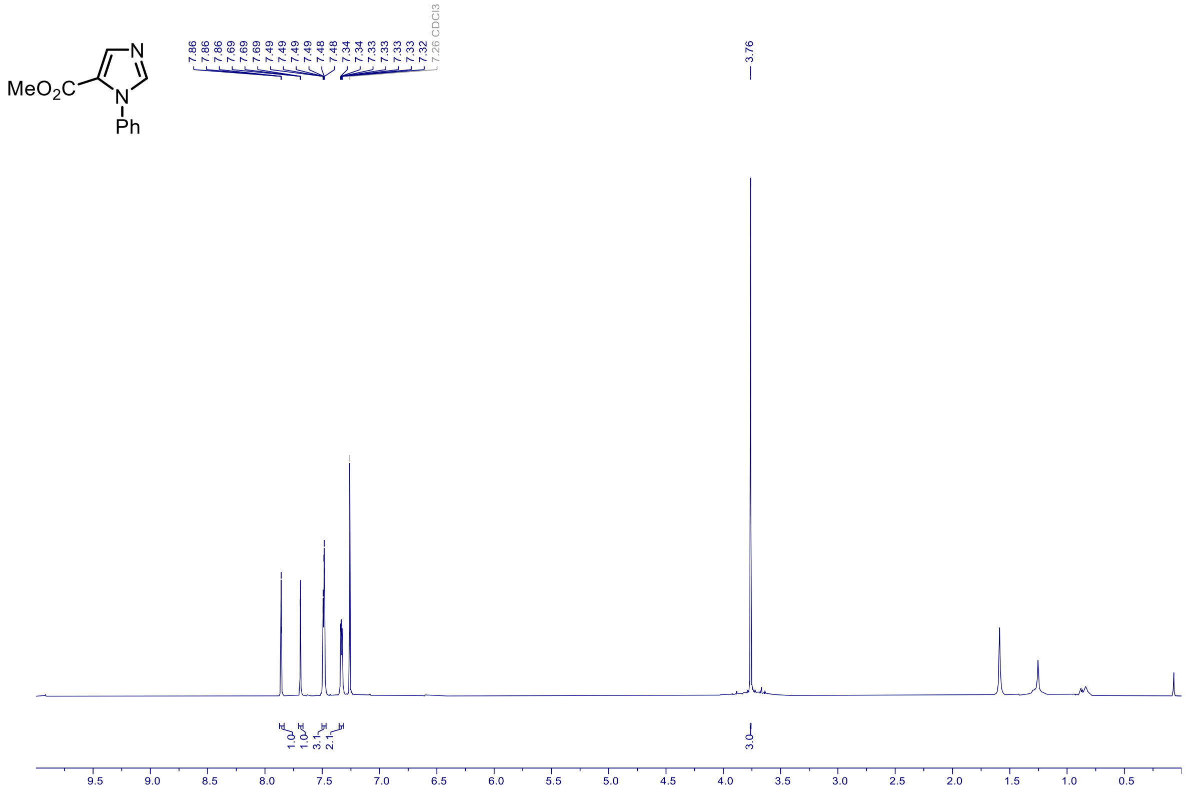

^{13}C NMR (151 MHz, $CDCl_3$)

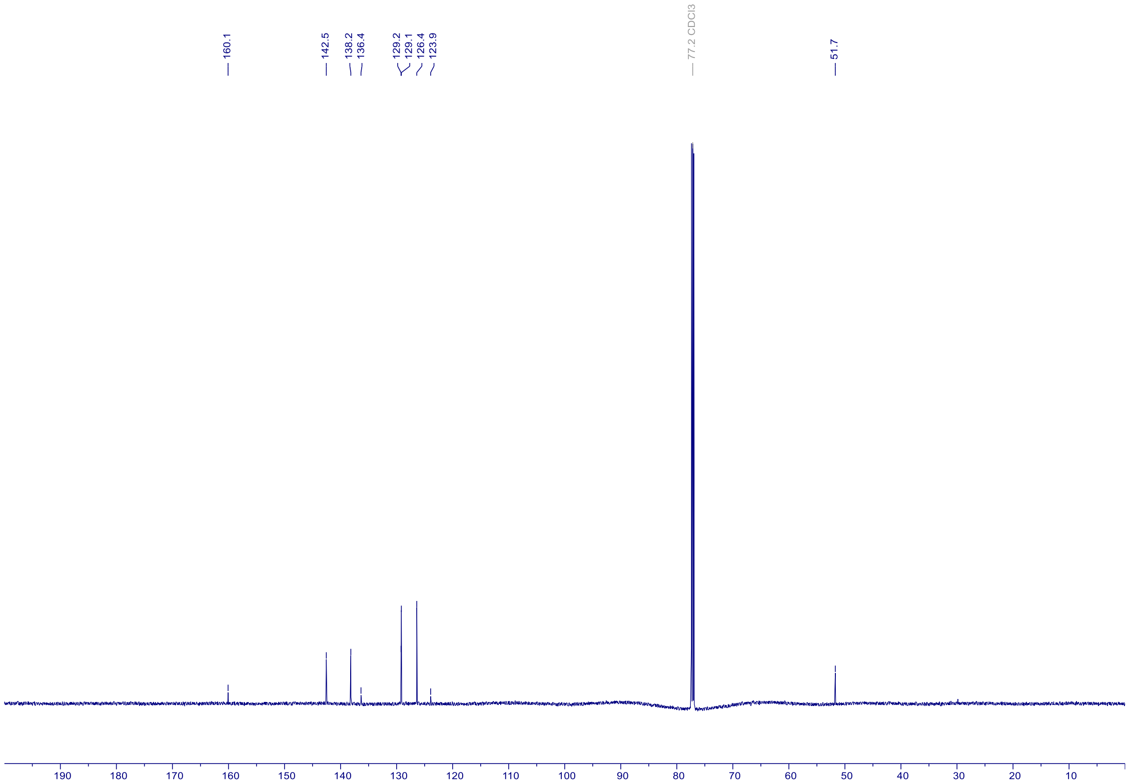

2-Methoxy-1-phenyl-1*H*-imidazole (19b)

^{1}H NMR (600 MHz, $CDCl_3$)

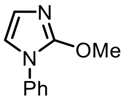

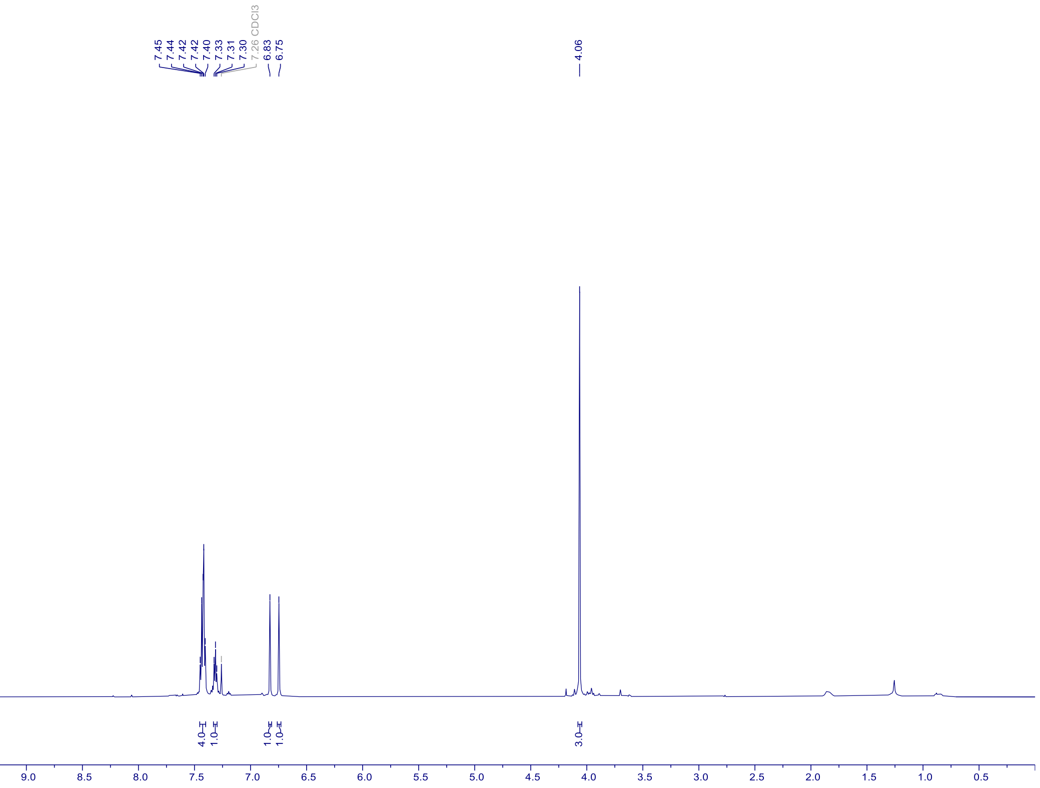

^{13}C NMR (151 MHz, $CDCl_3$)

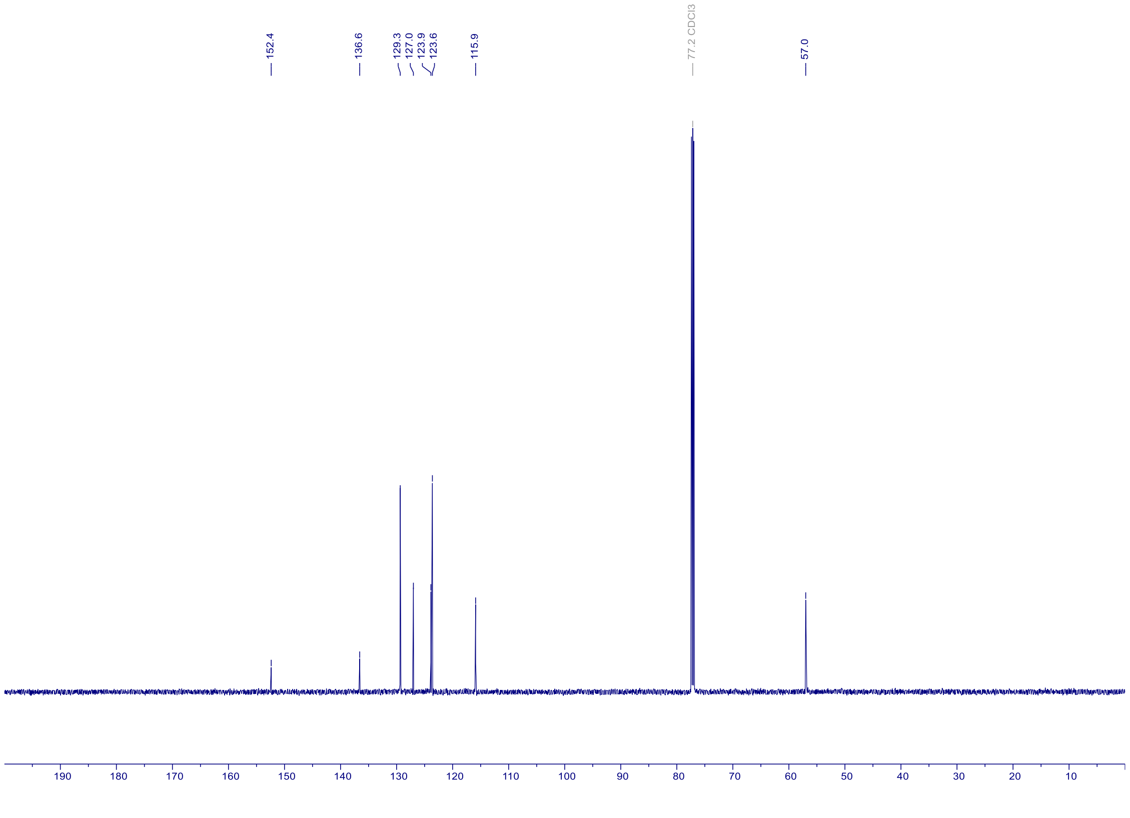

4-Methoxy-1-phenyl-1*H*-imidazole (20b)

^{1}H NMR (600 MHz, $CDCl_3$)

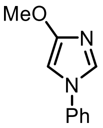

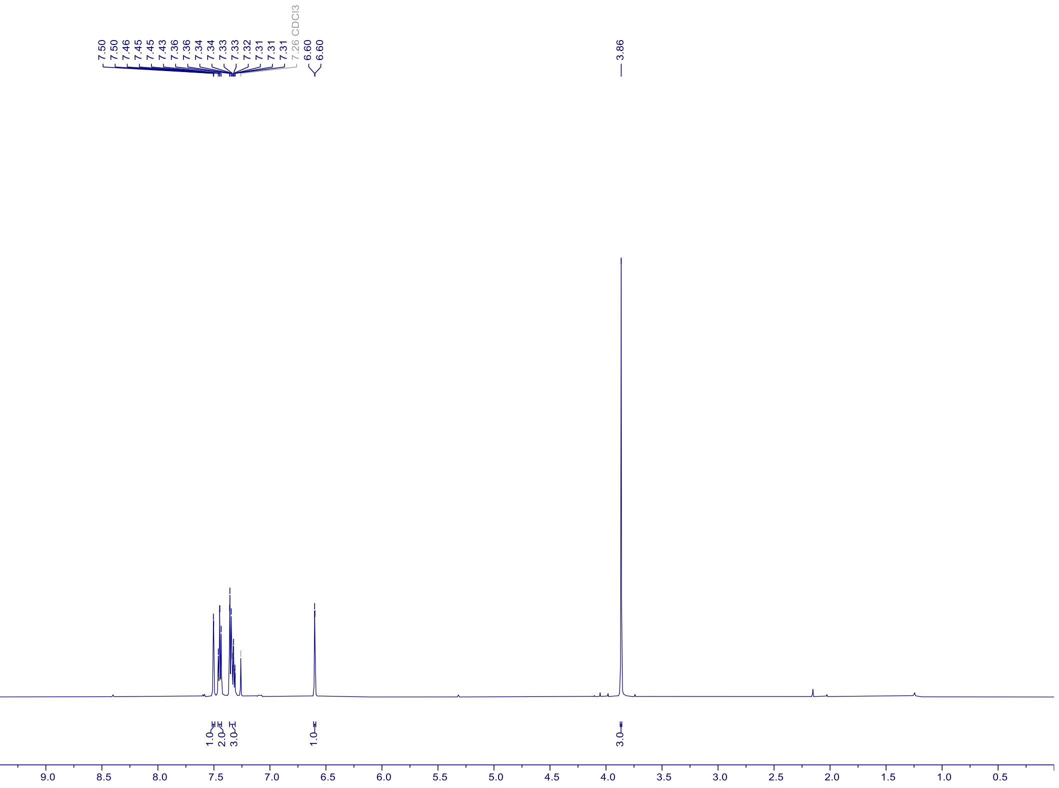

^{13}C NMR (151 MHz, $CDCl_3$)

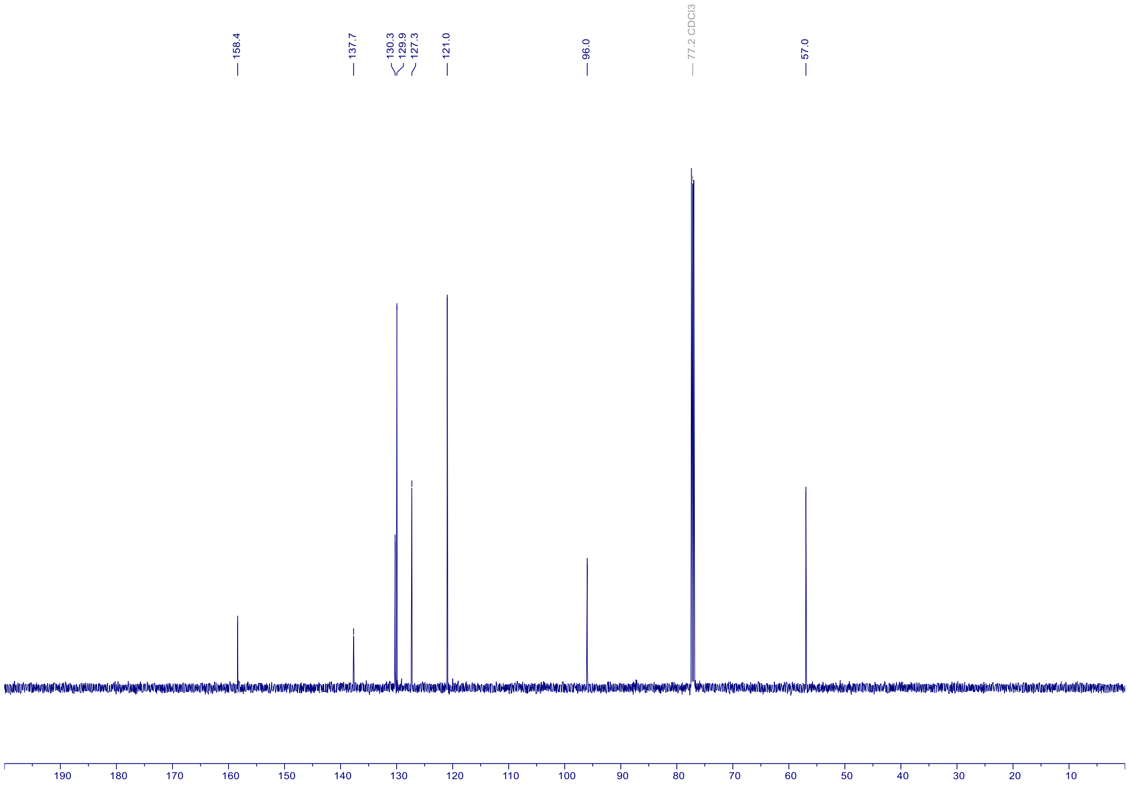

1-Phenyl-4-(trifluoromethyl)-1*H*-imidazole (23b)

^1H NMR (600 MHz, $CDCl_3$)

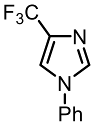

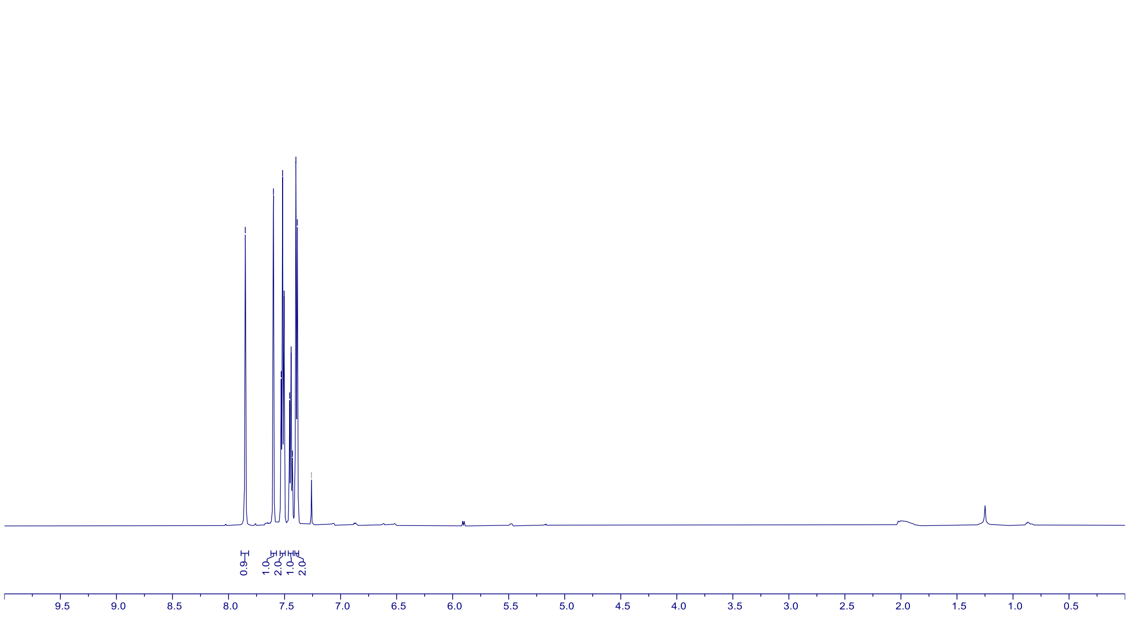

^{13}C NMR (151 MHz, $CDCl_3$)

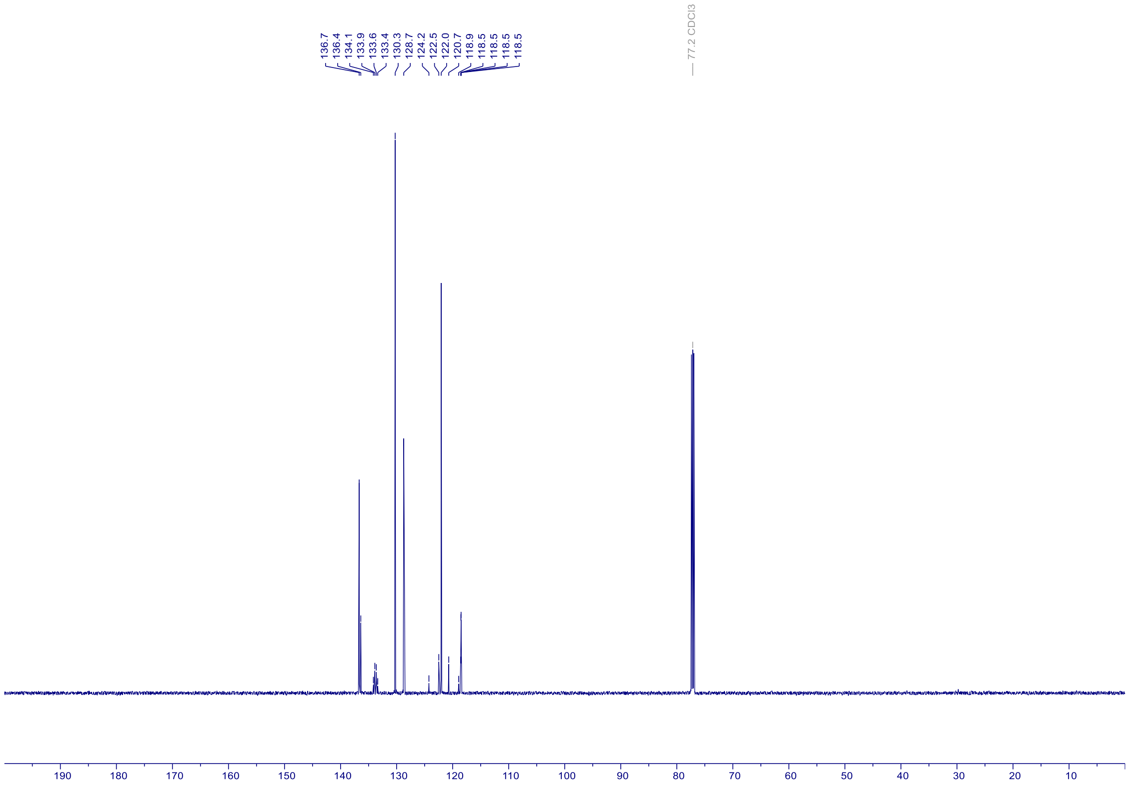

^{19}F NMR (565 MHz, $CDCl_3$)

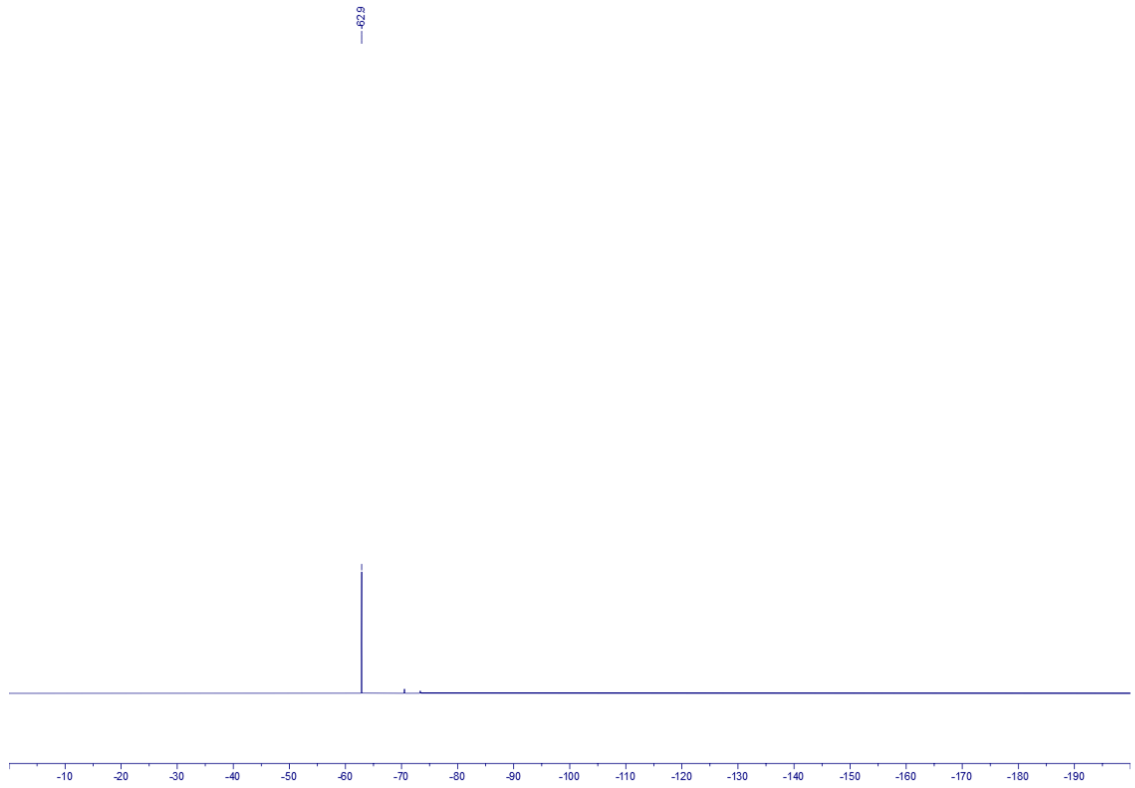

1,4-Diphenyl-1*H*-imidazole (26b)

^{1}H NMR (600 MHz, $CDCl_3$)

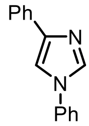

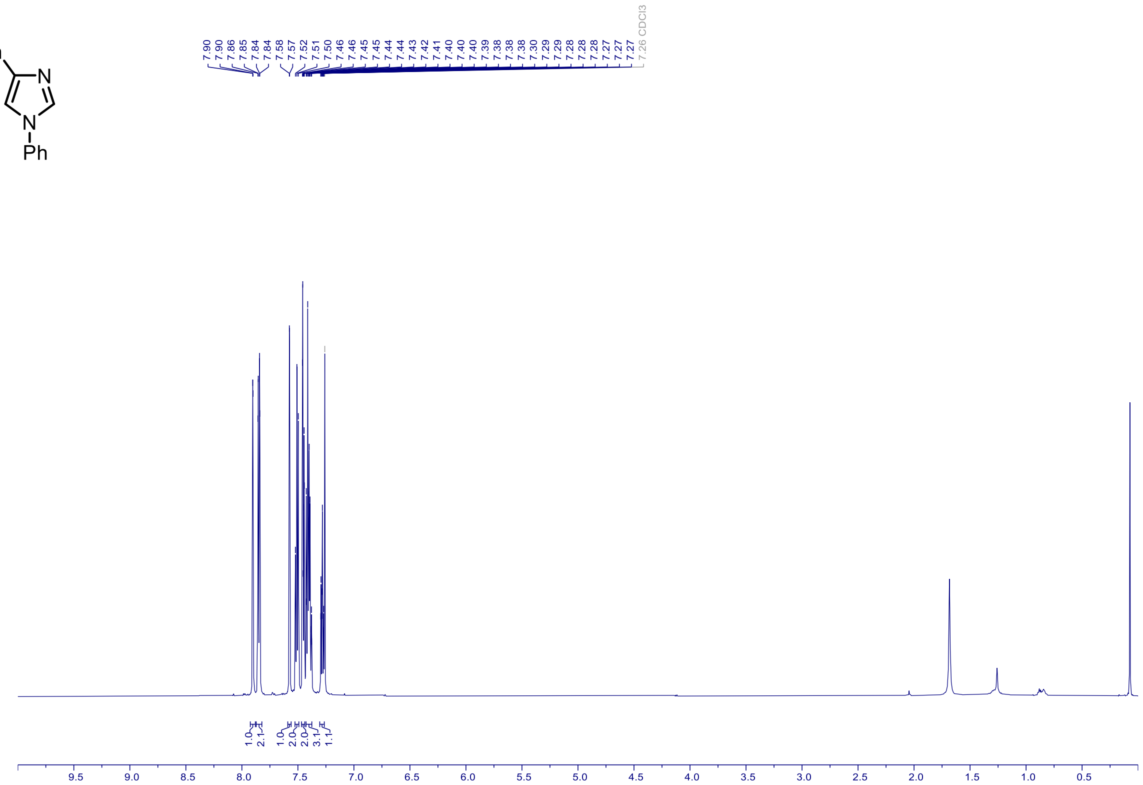

^{13}C NMR (151 MHz, $CDCl_3$)

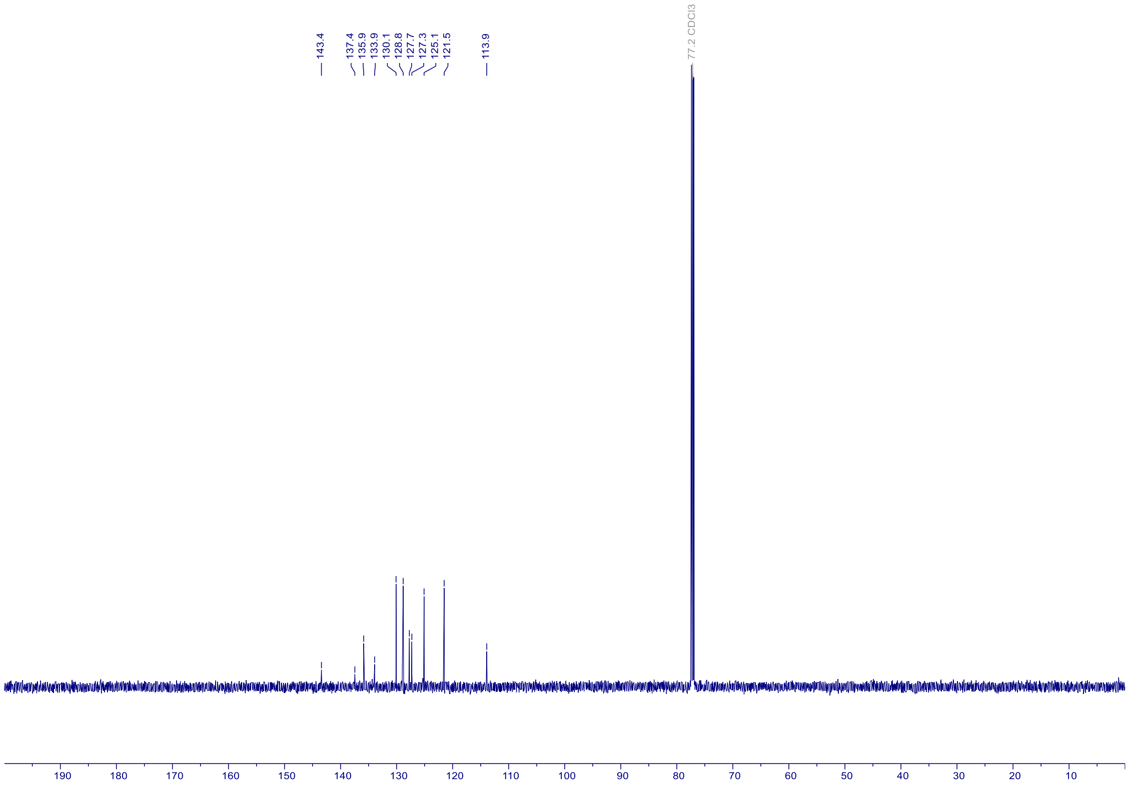

1-(4-Methoxyphenyl)-4-phenyl-1*H*-imidazole (31b)

^{1}H NMR (600 MHz, $CDCl_3$)

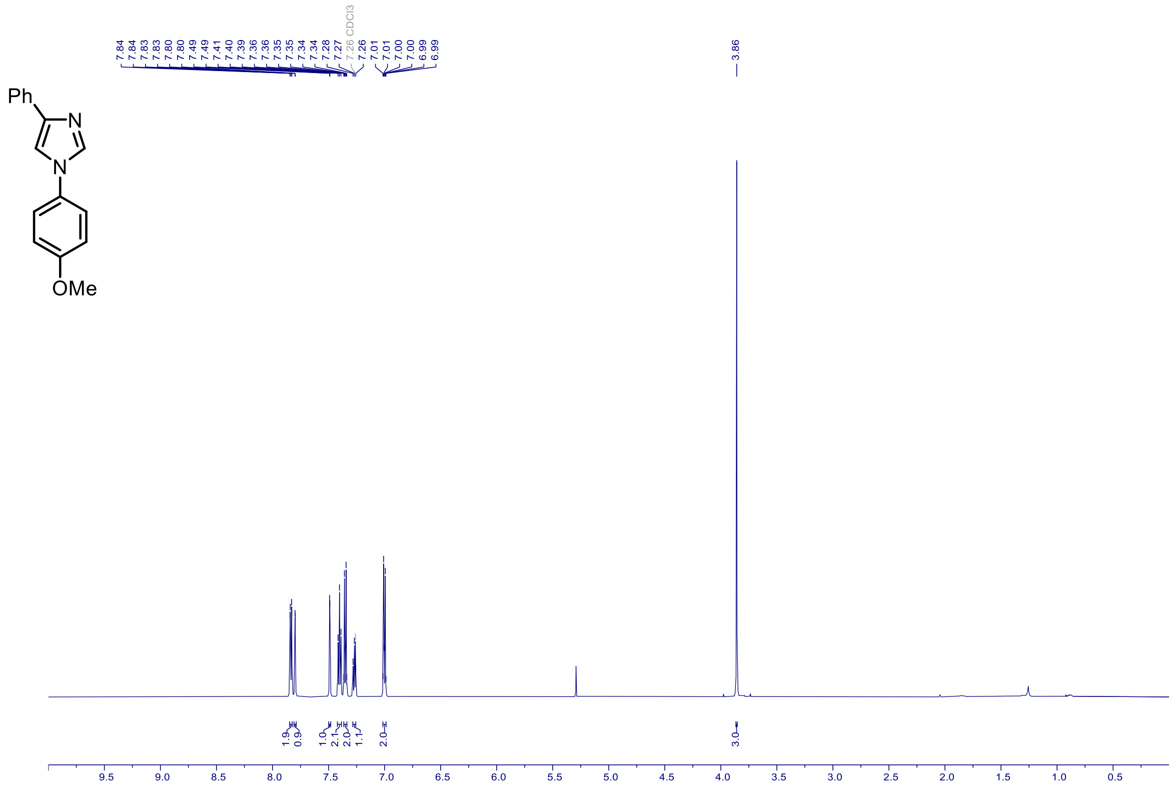

^{13}C NMR (151 MHz, $CDCl_3$)

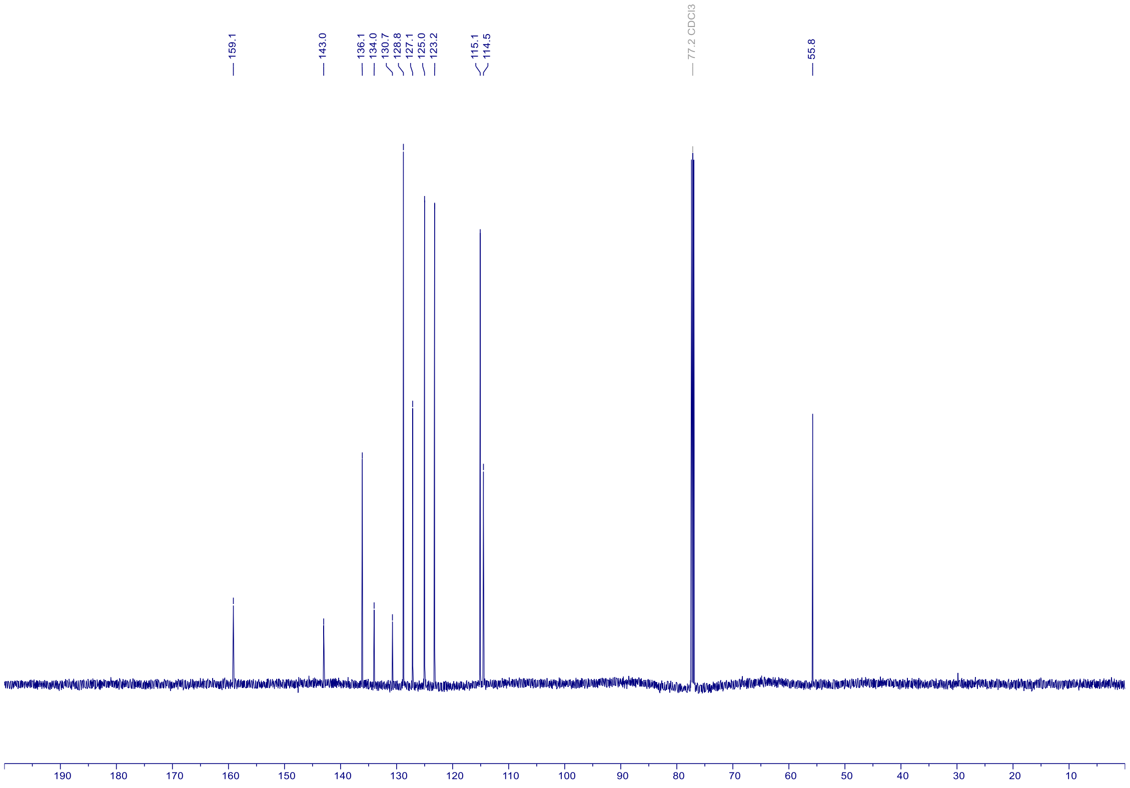

4-Phenyl-1-(4-(trifluoromethyl)phenyl)-1*H*-imidazole (32b)

^{1}H NMR (600 MHz, $CDCl_3$)

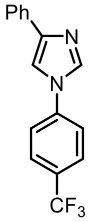

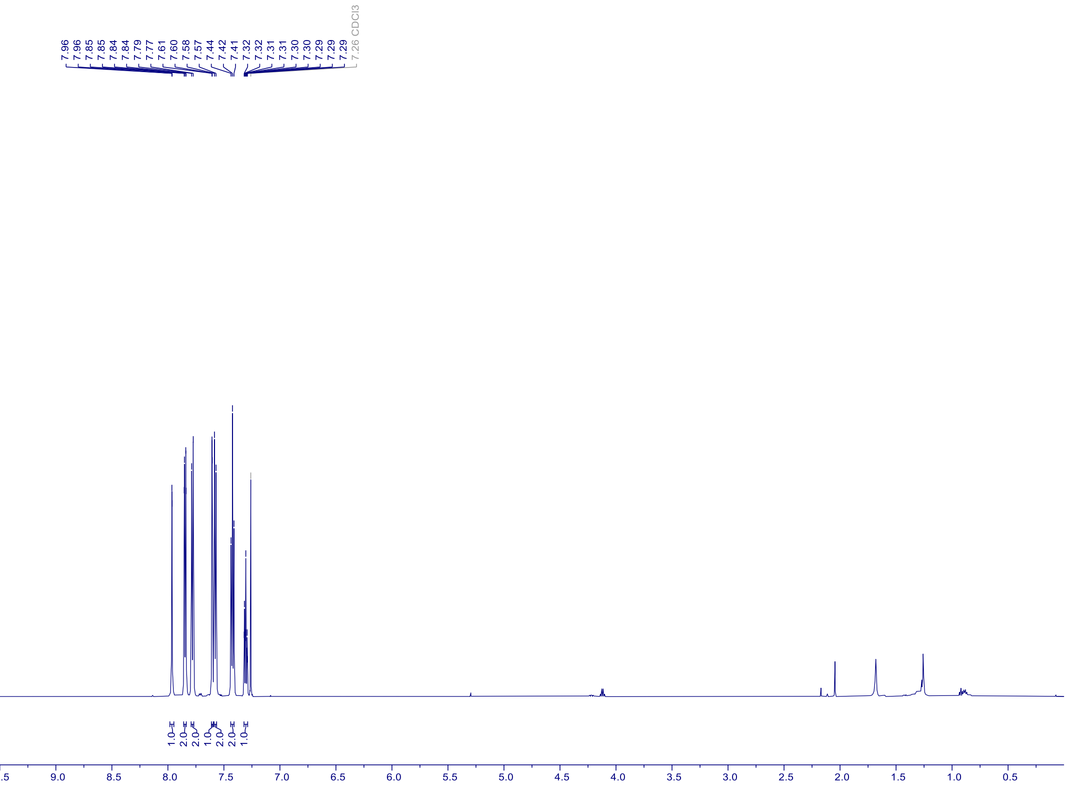

^{13}C NMR (151 MHz, $CDCl_3$)

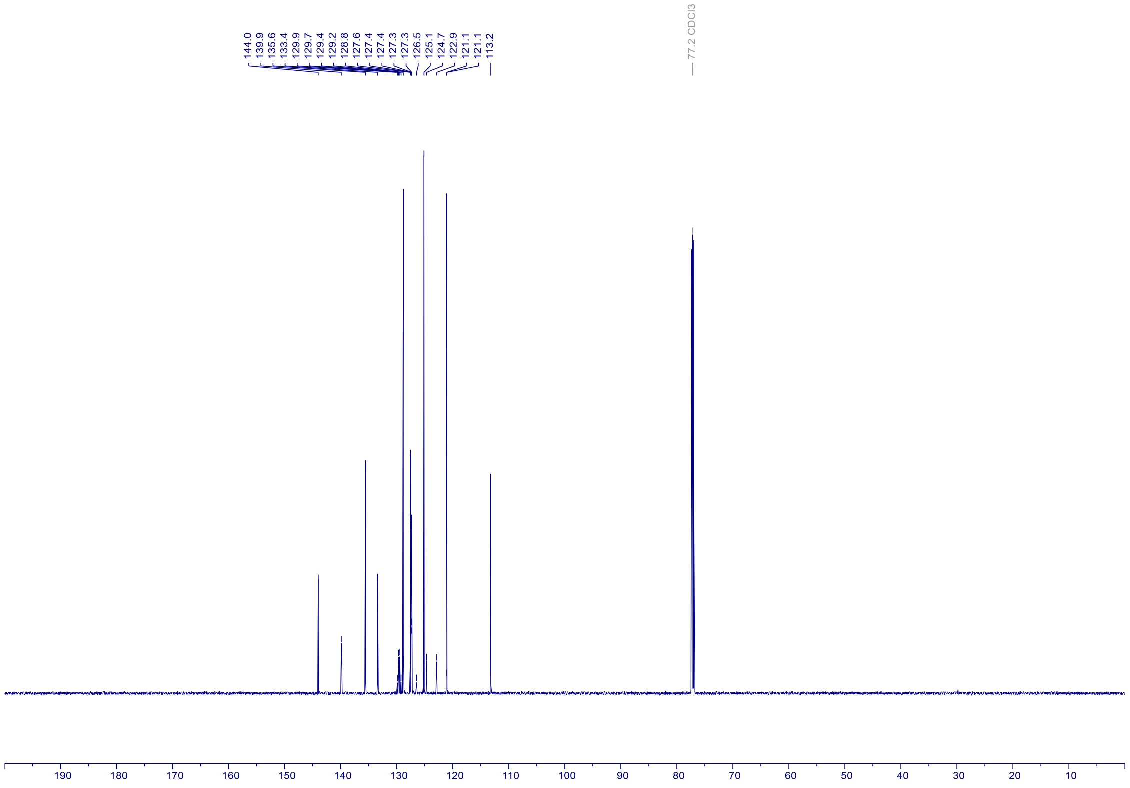

^{19}F NMR (564 MHz, $CDCl_3$)

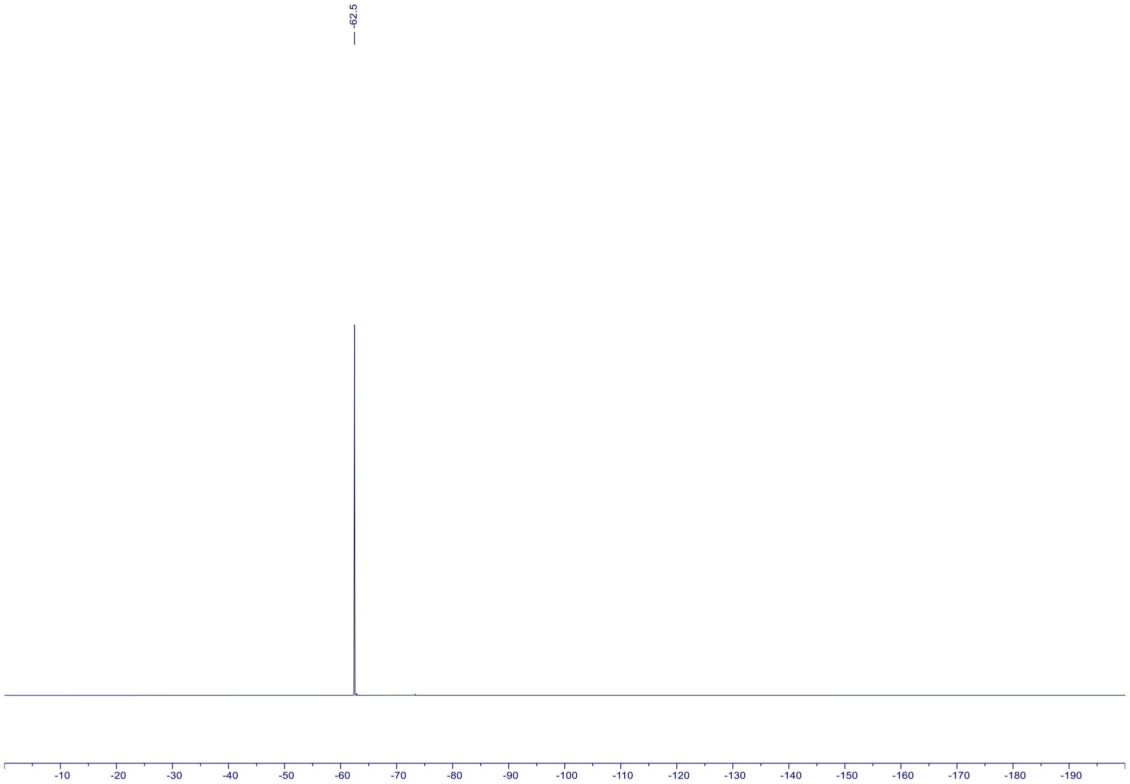

4-Phenyl-1-(*o*-tolyl)-1*H*-imidazole (33b)

^{1}H NMR (600 MHz, $CDCl_3$)

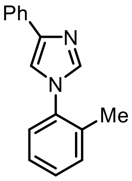

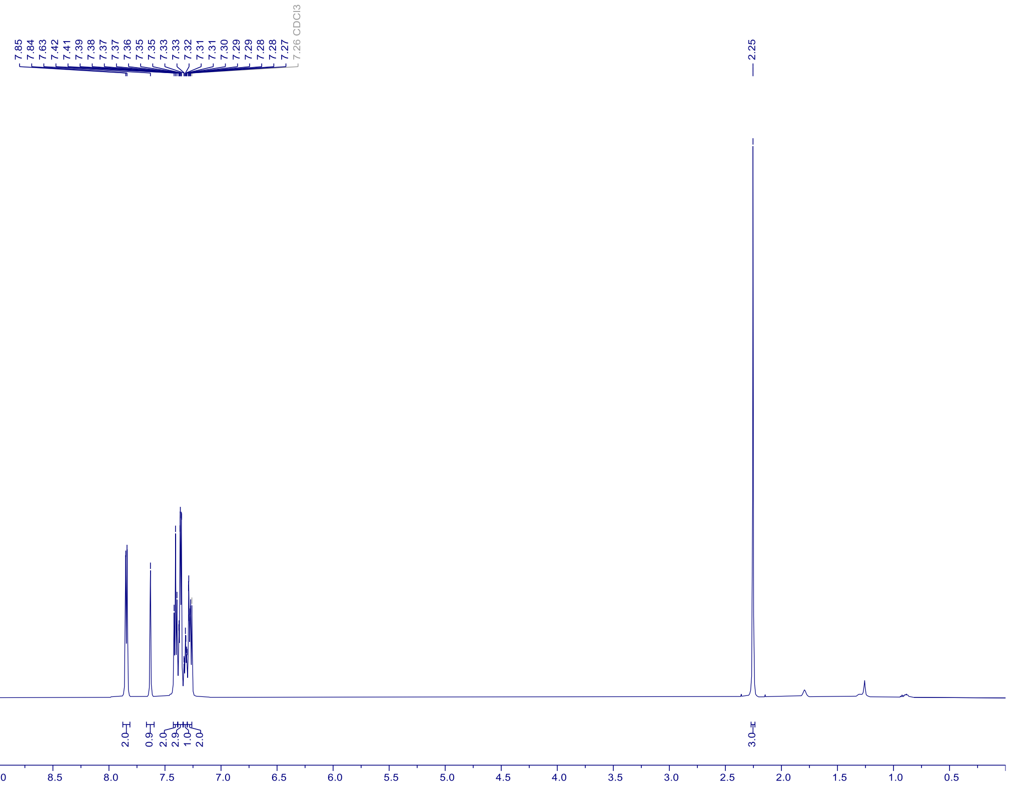

^{13}C NMR (151 MHz, $CDCl_3$)

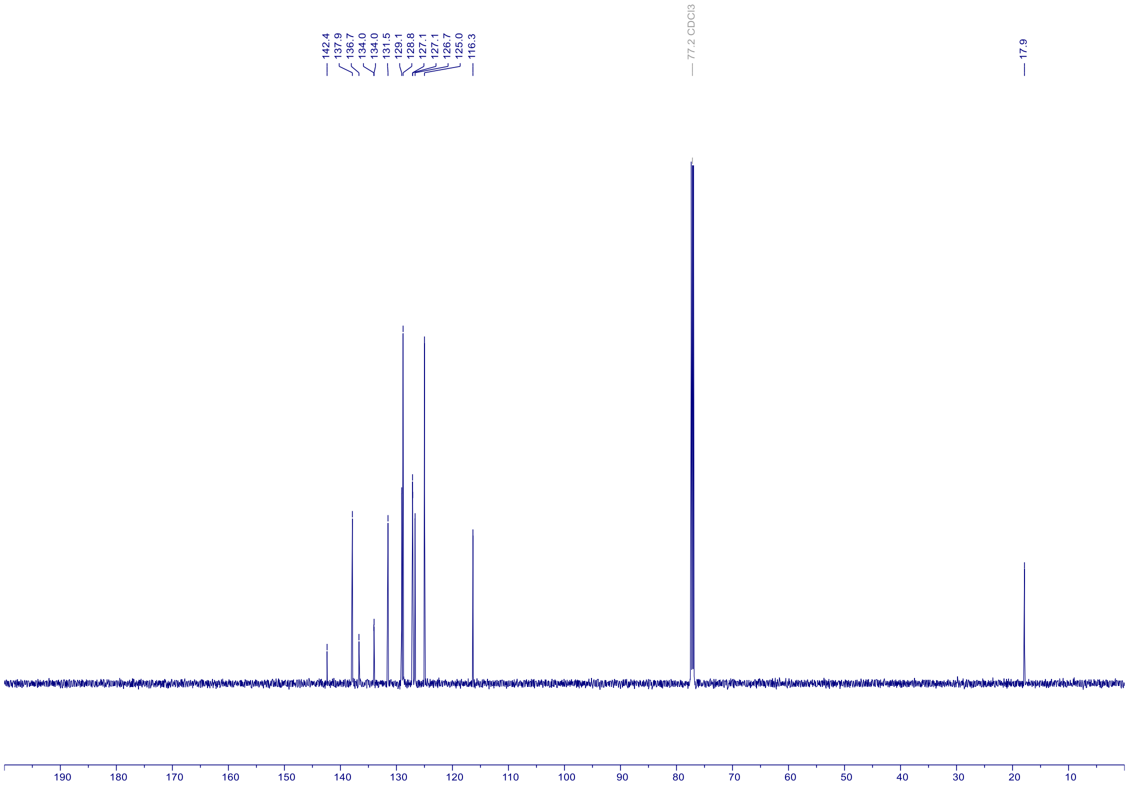

4-Phenyl-1-vinyl-1*H*-imidazole (34b)

^{1}H NMR (600 MHz, $CDCl_3$)

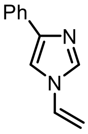

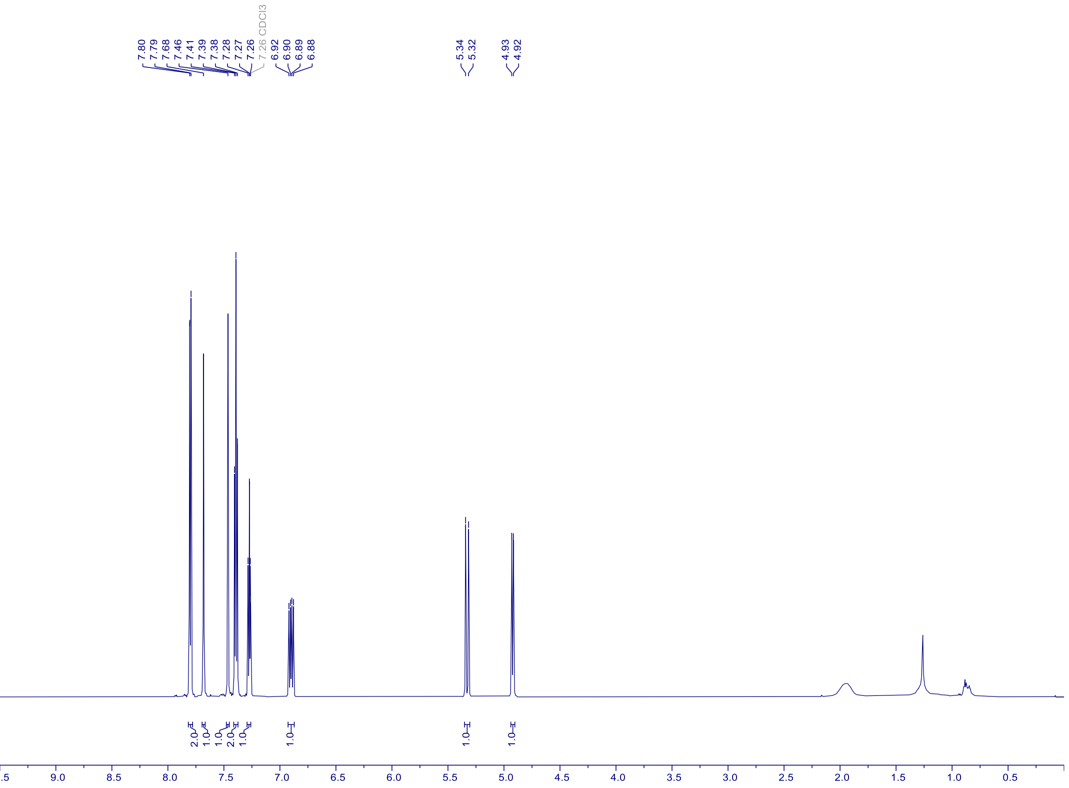

^{13}C NMR (151 MHz, $CDCl_3$)

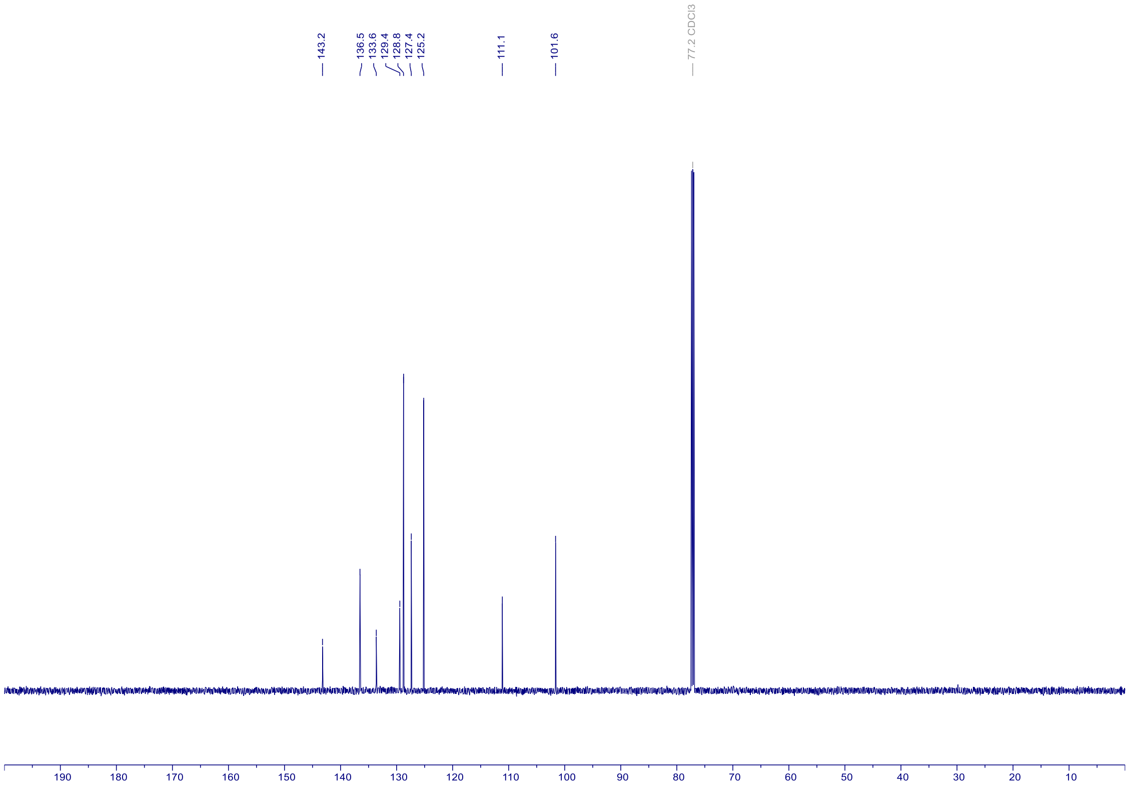

***N*,*N*-dimethyl-1-phenyl-1*H*-imidazole-4-carboxamide (42b)**

^{1}H NMR (600 MHz, $CDCl_3$)

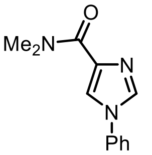

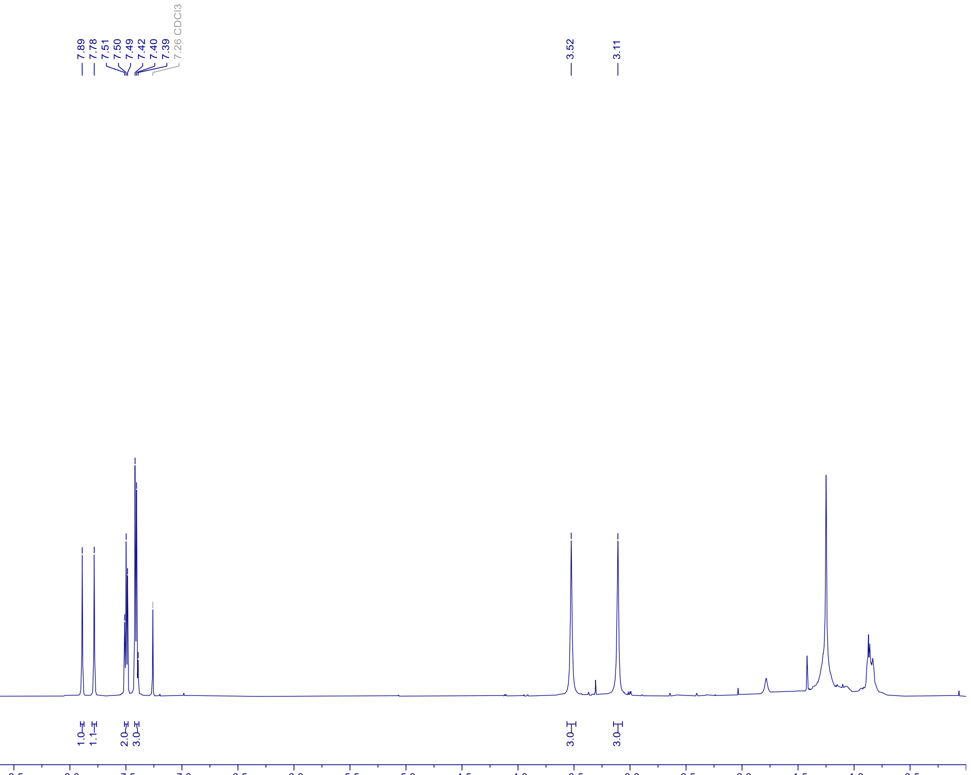

^{13}C NMR (151 MHz, $CDCl_3$)

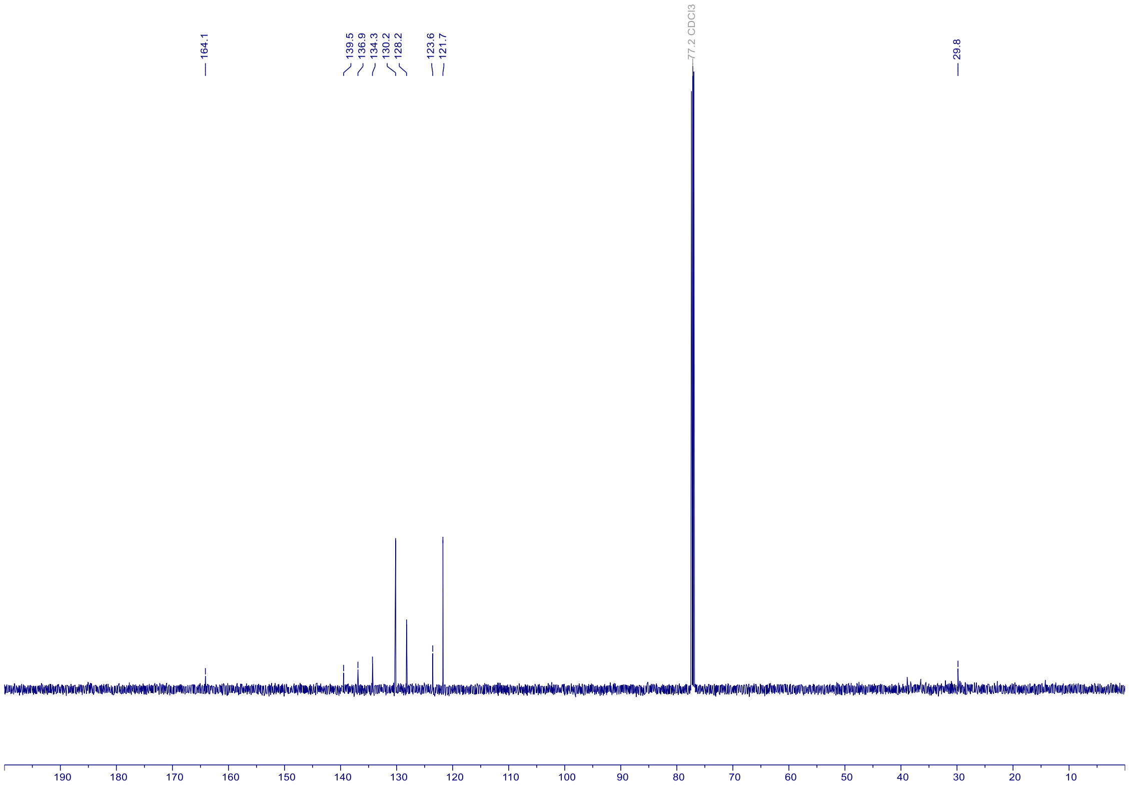

(1-Phenyl-1*H*-imidazol-4-yl)methanol (44b)

^{1}H NMR (600 MHz, $CDCl_3$)

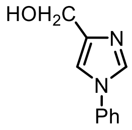

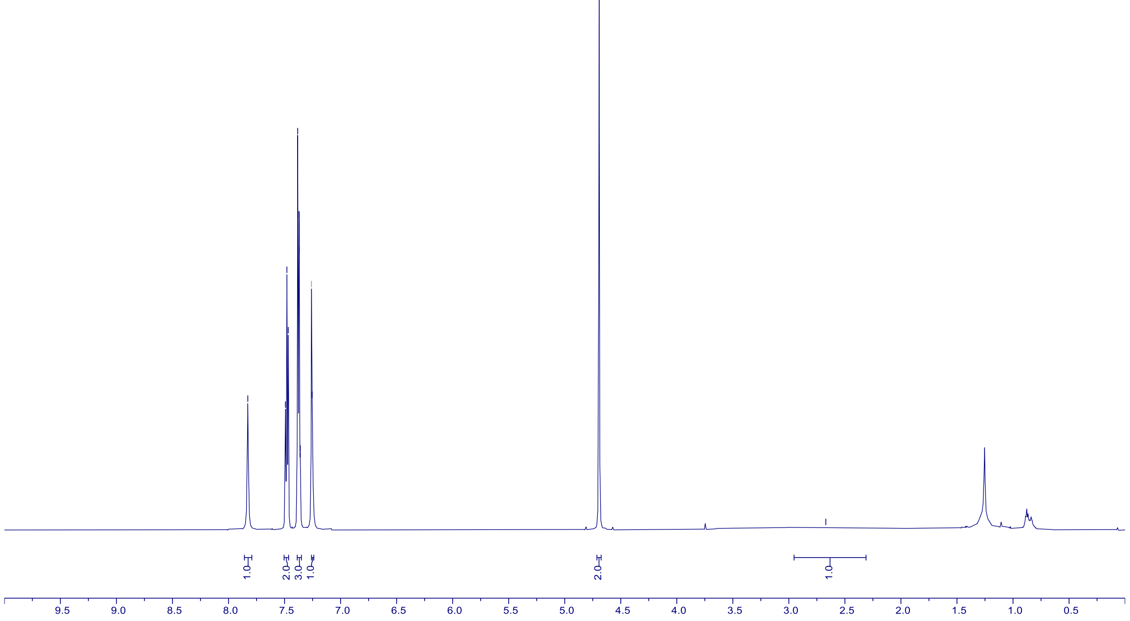

^{13}C NMR (151 MHz, $CDCl_3$)

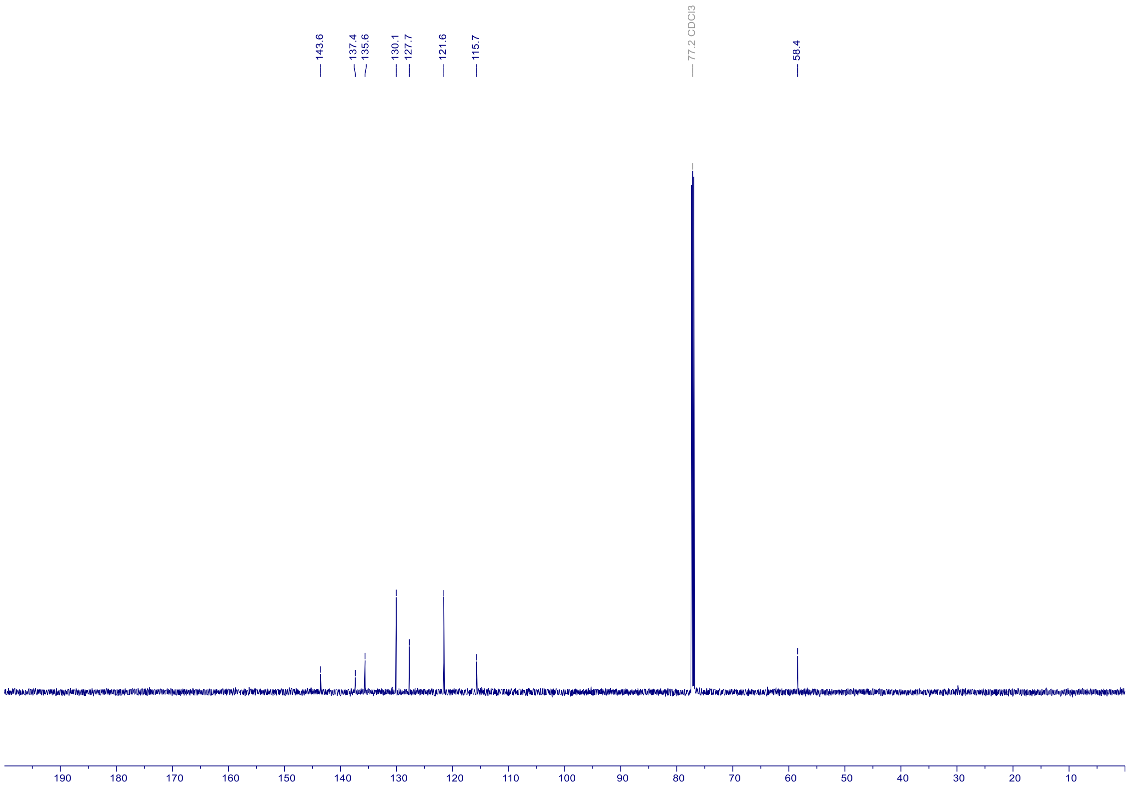

4-Fluoro-1-phenyl-1*H*-imidazole (45b)

^{1}H NMR (600 MHz, $CDCl_3$)

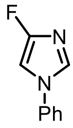

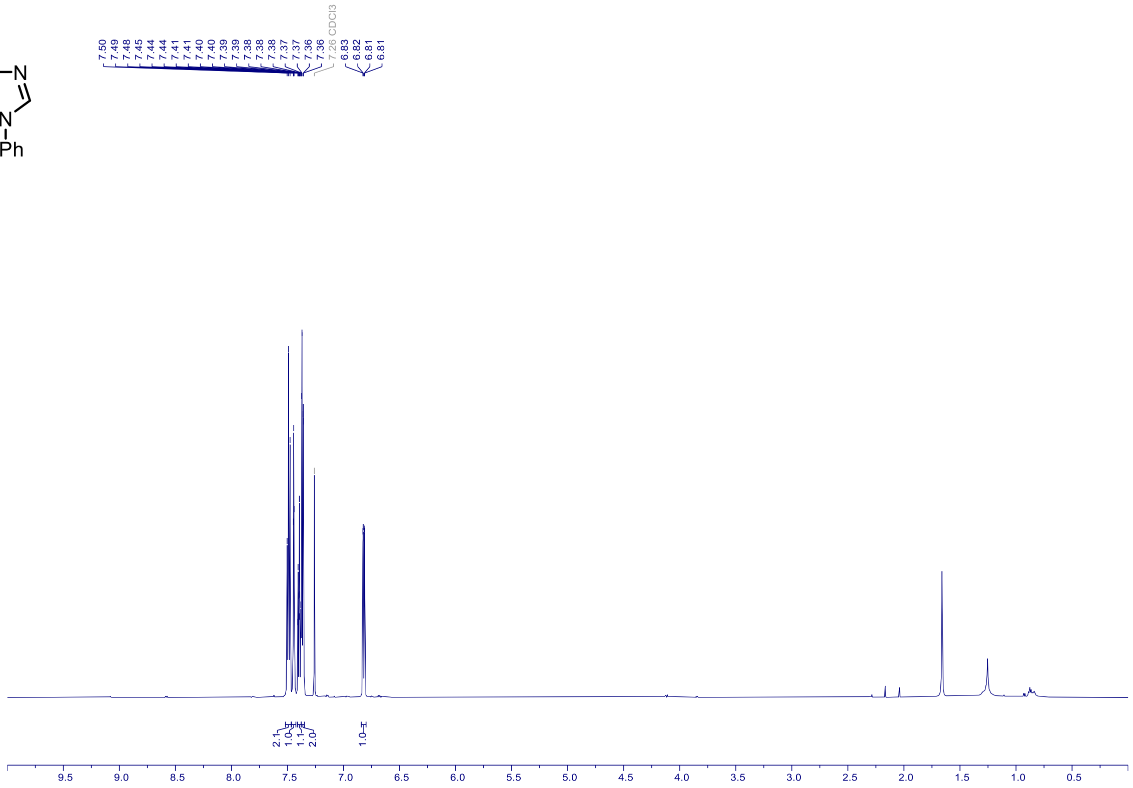

^{13}C NMR (151 MHz, $CDCl_3$)

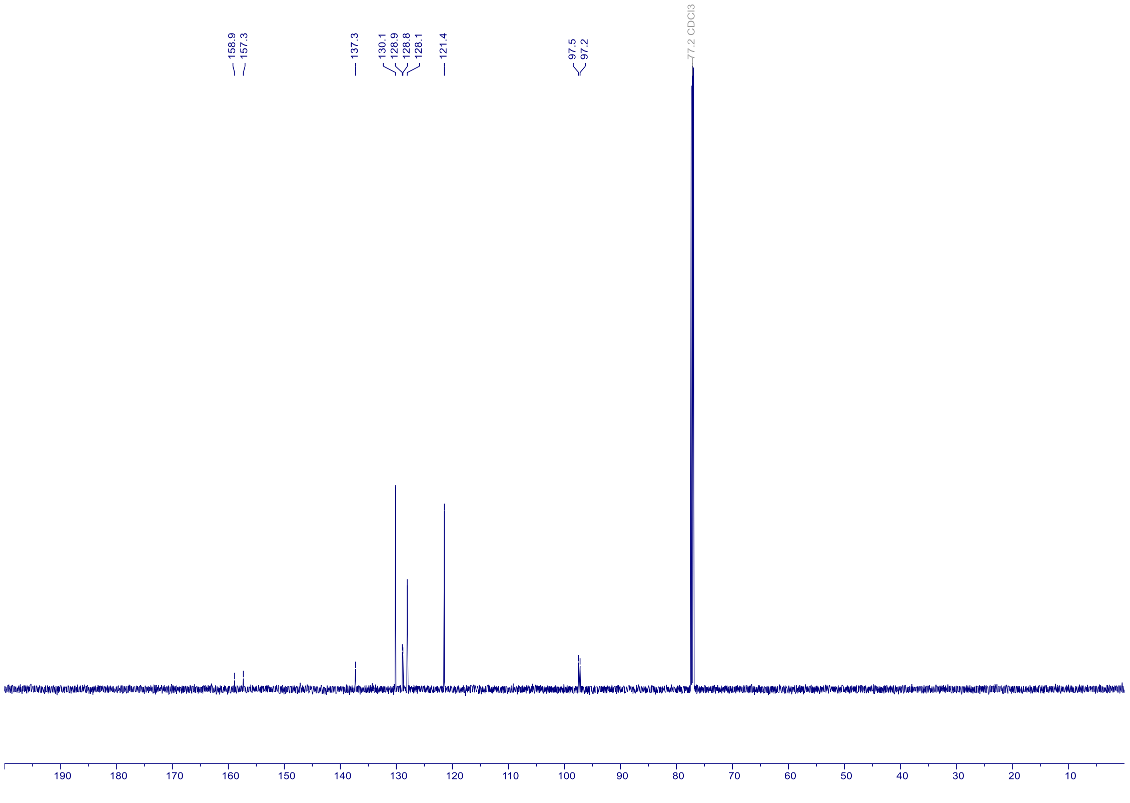

^{19}F NMR (565 MHz, $CDCl_3$)

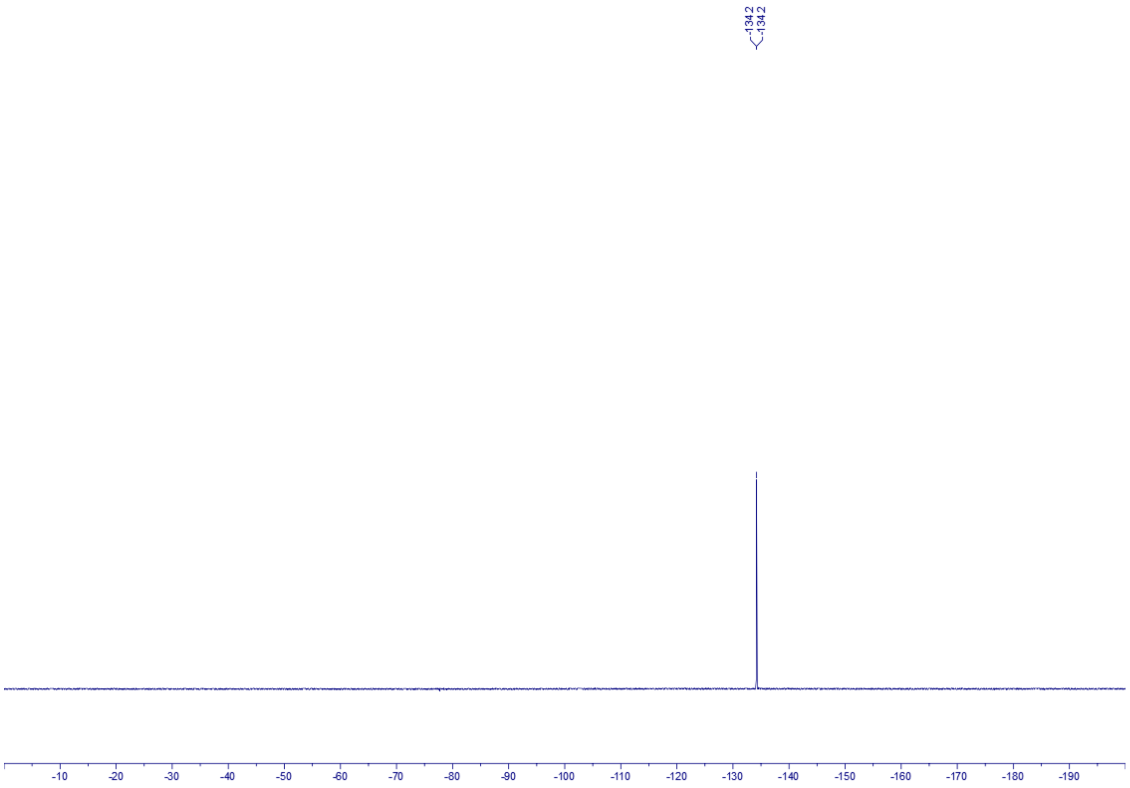

3,5-Dimethyl-1-(1-phenyl-1*H*-imidazol-4-yl)-1*H*-pyrazole (46b)

^{1}H NMR (600 MHz, $CDCl_3$)

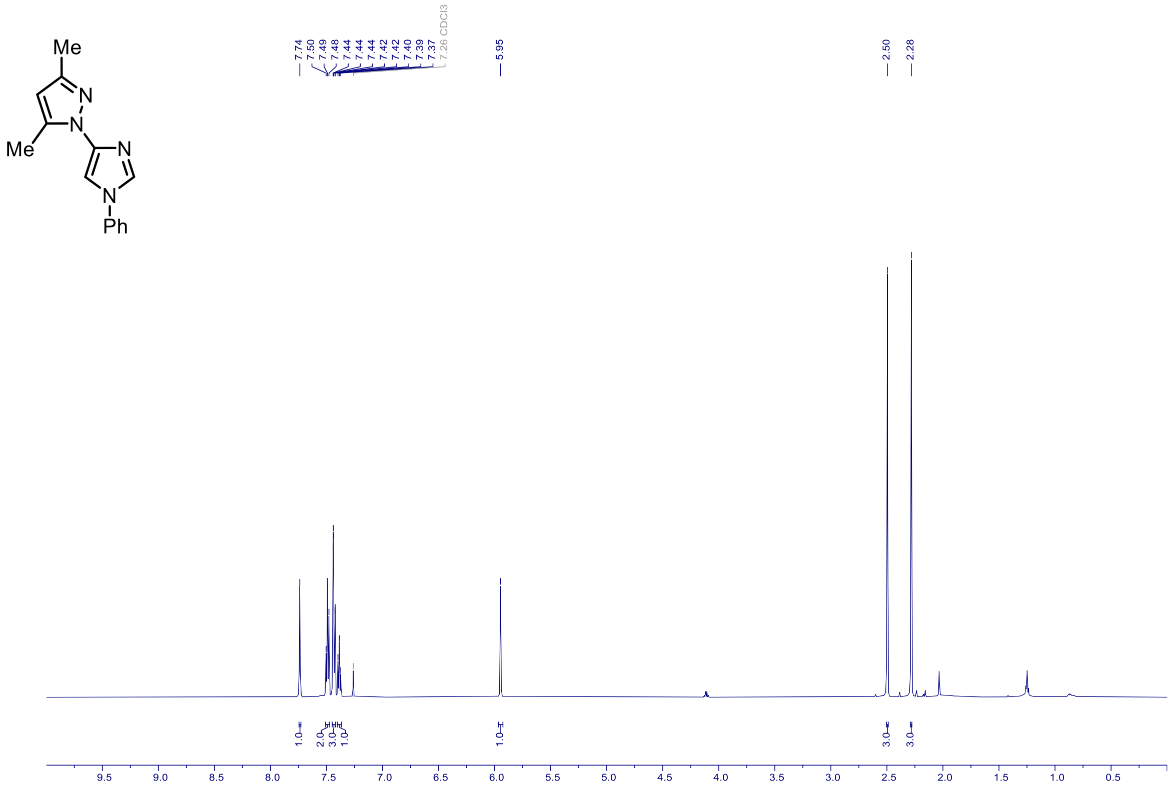

^{13}C NMR (151 MHz, $CDCl_3$)

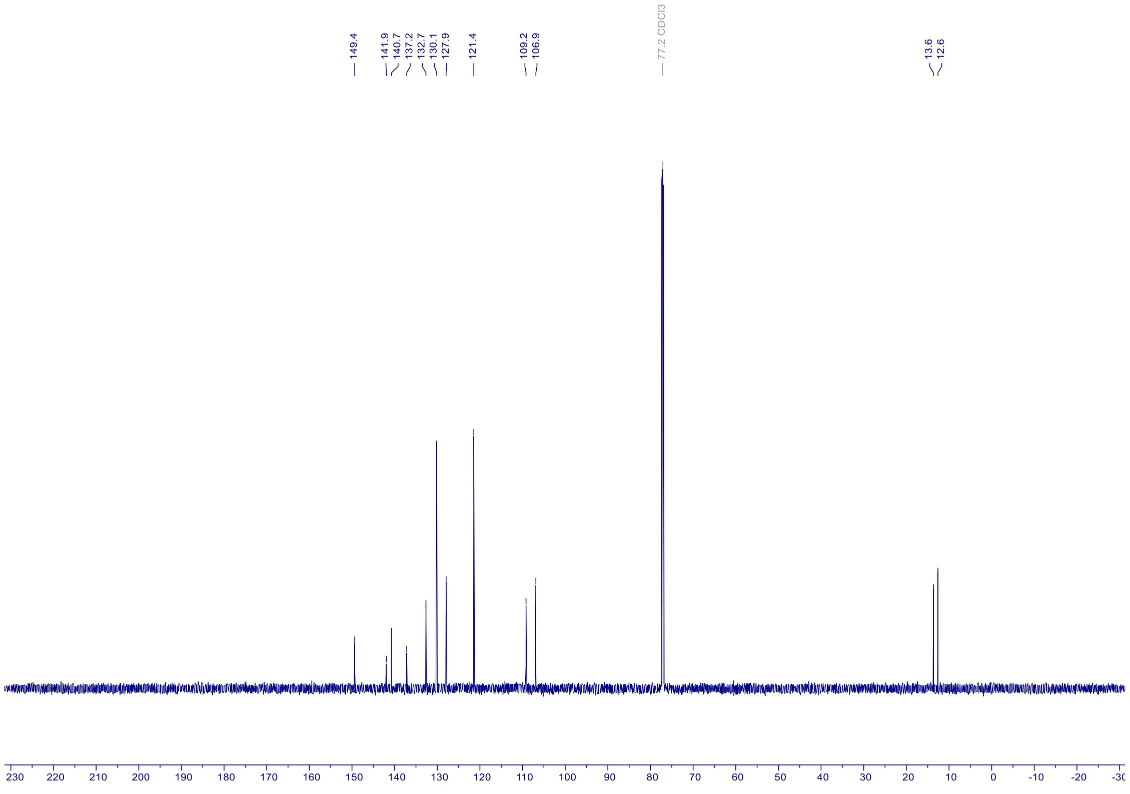

5-Methyl-1,4-diphenyl-1*H*-imidazole (49b)

^{1}H NMR (600 MHz, $CDCl_3$)

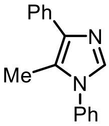

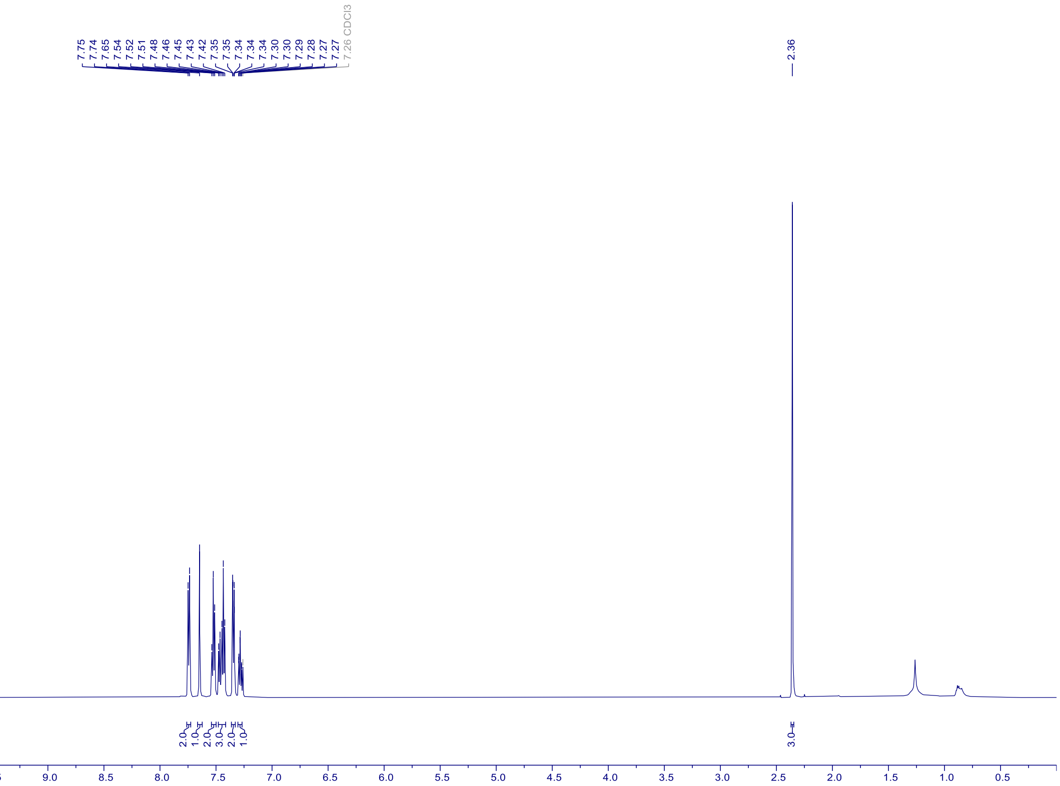

^{13}C NMR (151 MHz, $CDCl_3$)

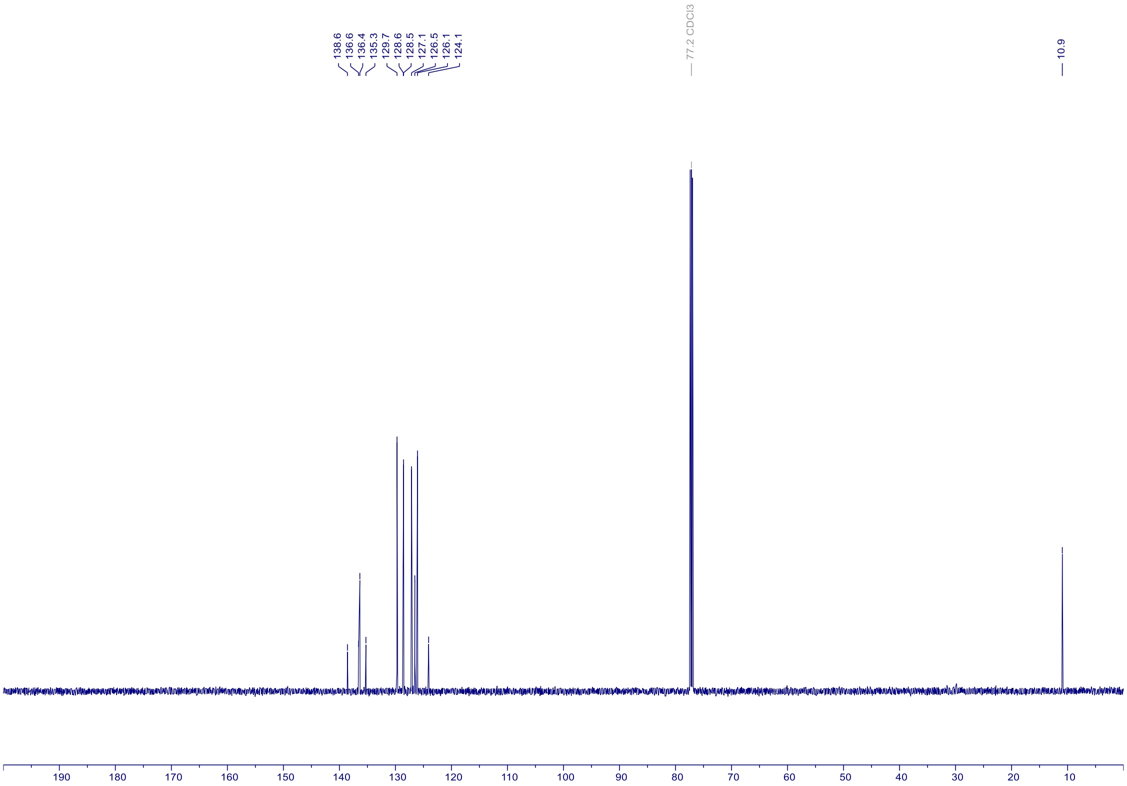

2-Methyl-1,4-diphenyl-1*H*-imidazole (50b)

^{1}H NMR (600 MHz, $CDCl_3$)

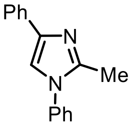

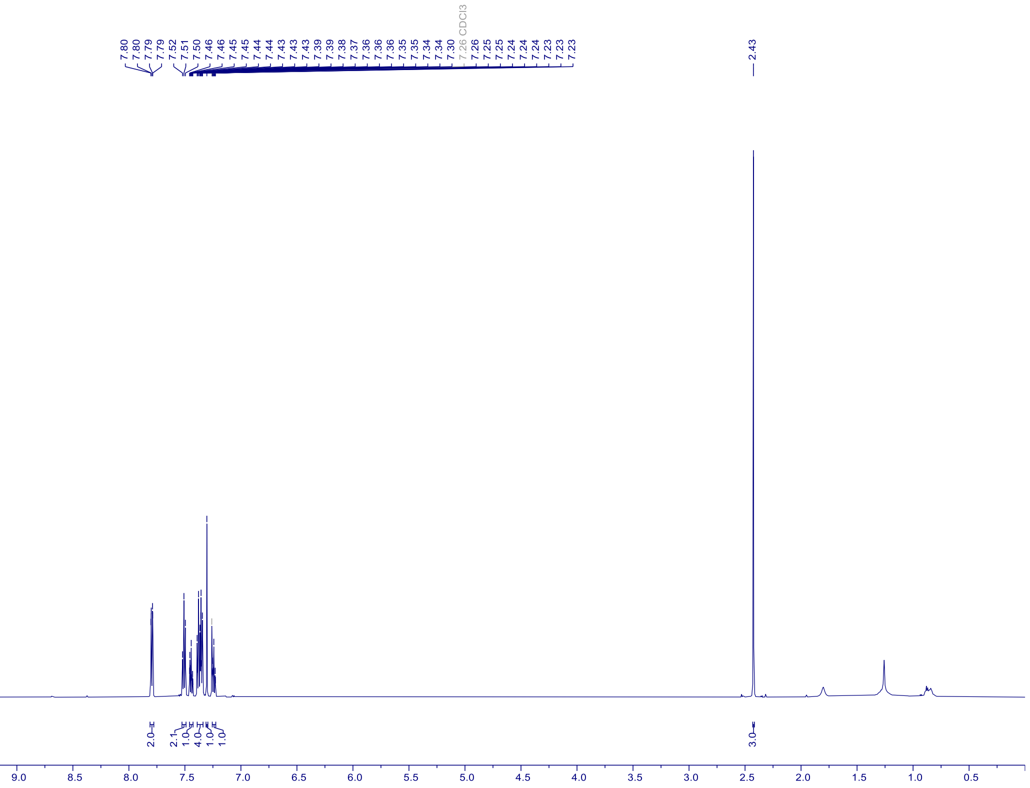

^{13}C NMR (151 MHz, $CDCl_3$)

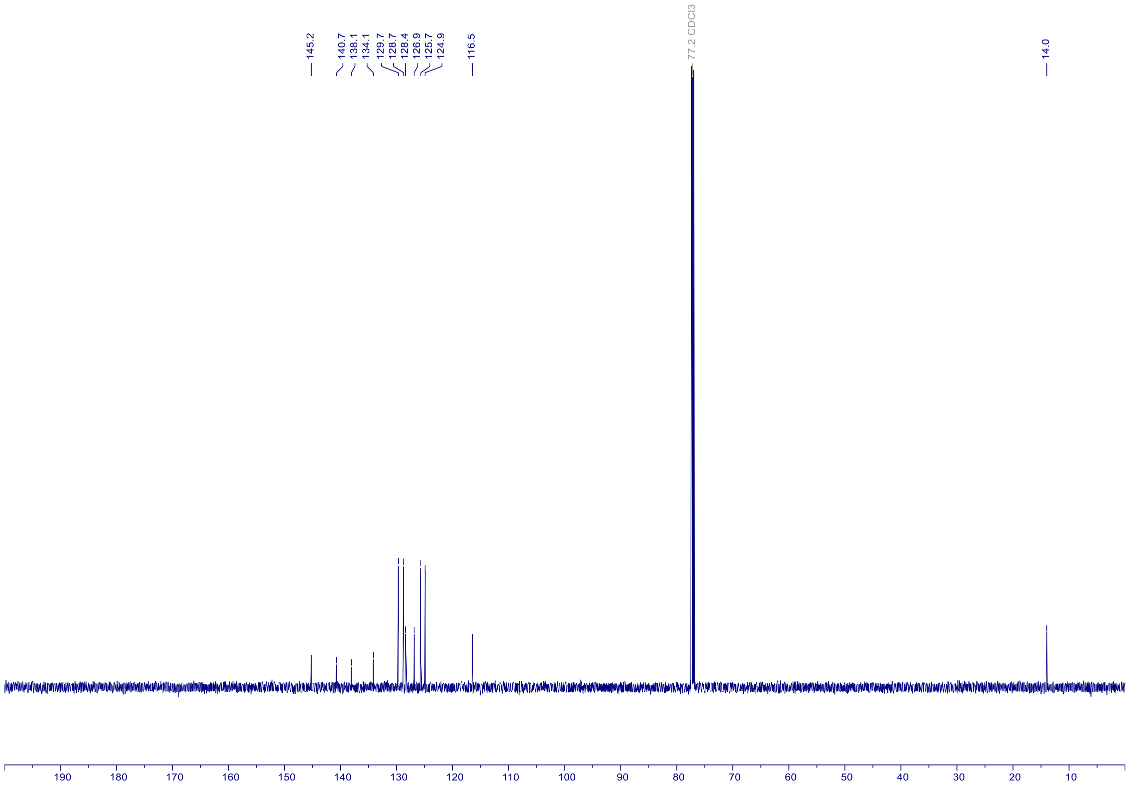

Methyl 5-Methyl-1-phenyl-1*H*-imidazole-2-carboxylate (52b)

^{1}H NMR (600 MHz, $CDCl_3$)

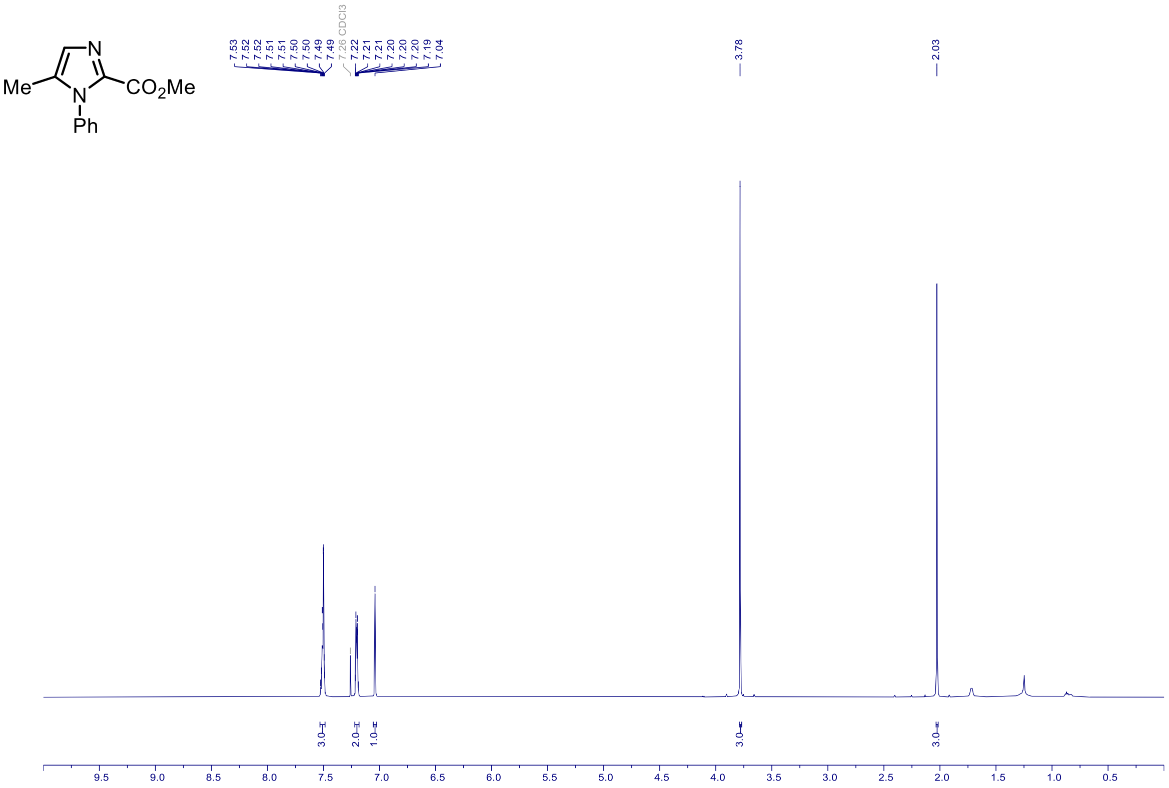

^{13}C NMR (151 MHz, $CDCl_3$)

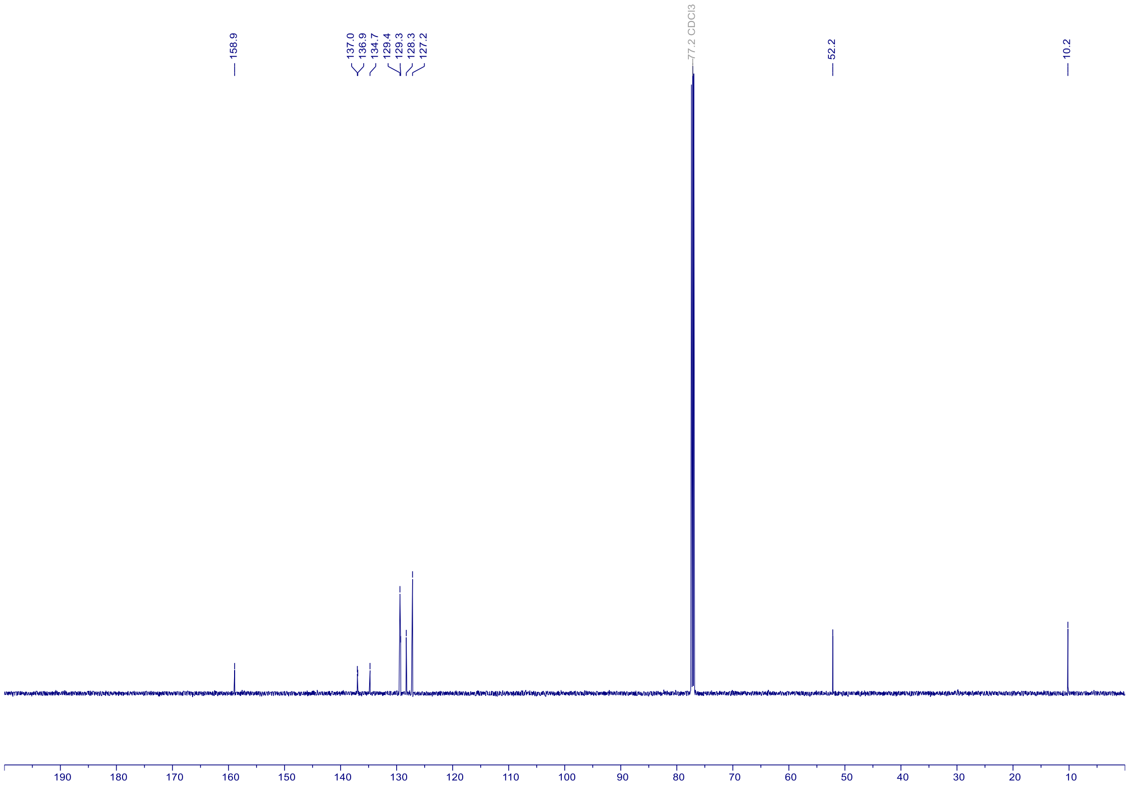

2-Methyl-4-phenyl-1*H*-imidazole (55b) and 5-Methyl-4-phenyl-1*H*-imidazole (55c)

^{1}H NMR (600 MHz, DMSO-d$_6$)

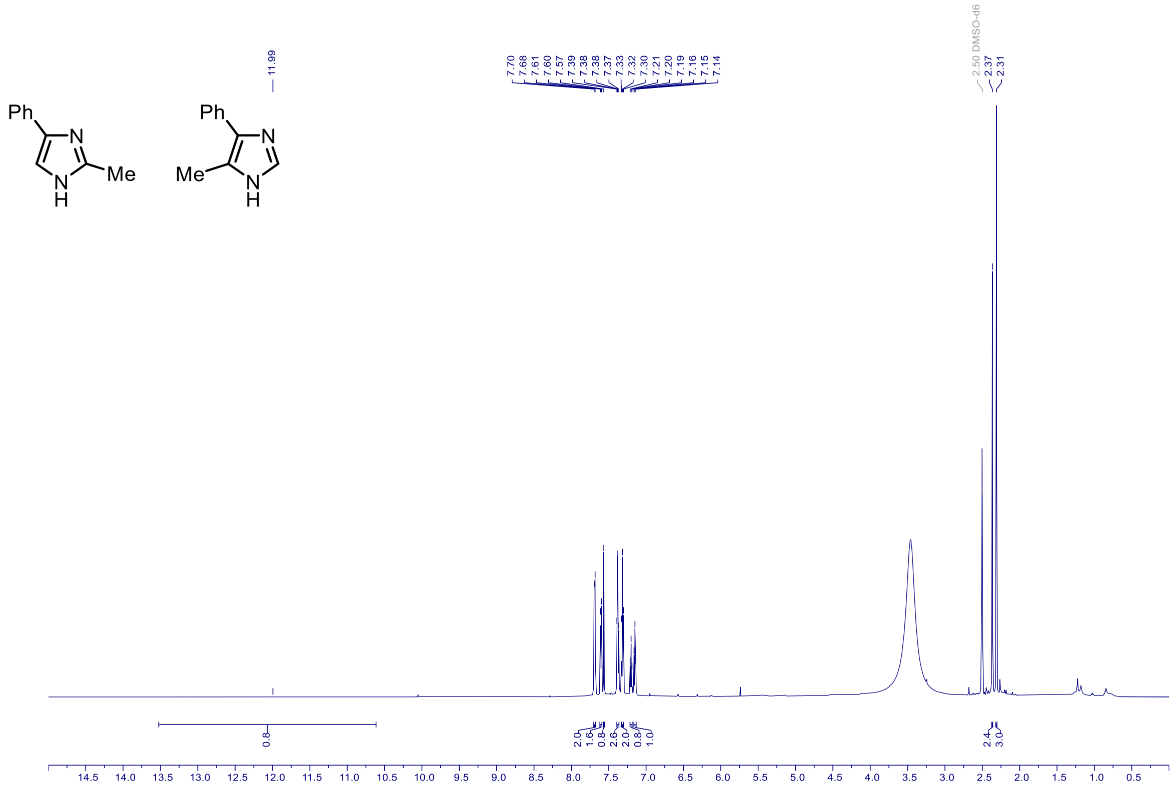

^{13}C NMR (151 MHz, DMSO-d$_6$)

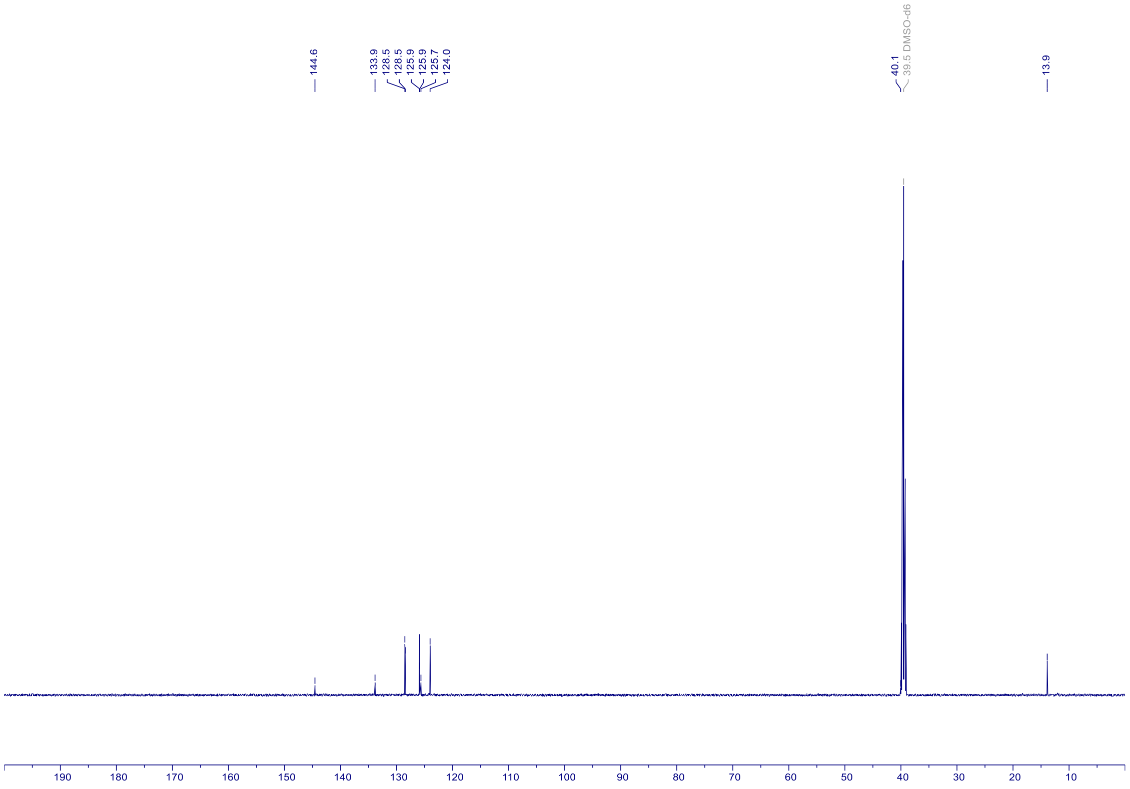

4-Methyl-2-phenyl-1*H*-imidazole (56b & 57b)

^{1}H NMR (600 MHz, $CDCl_3$)

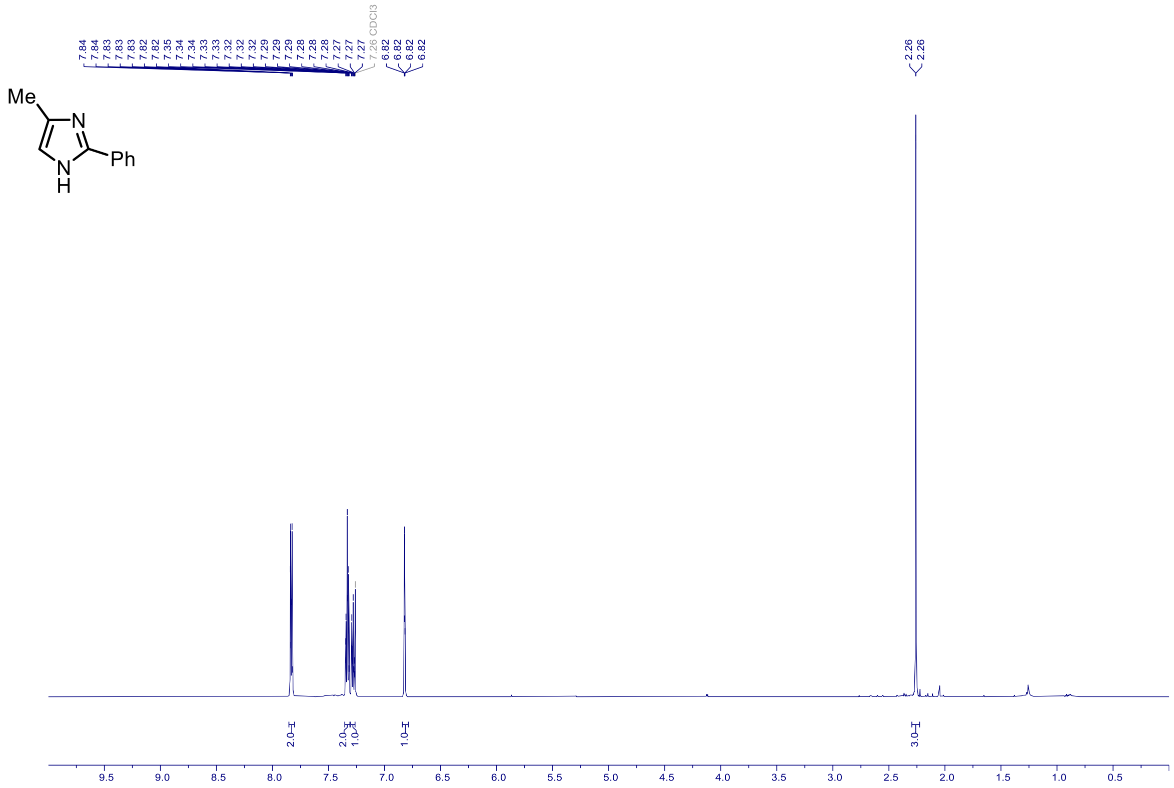

^{13}C NMR (151 MHz, $CDCl_3$)

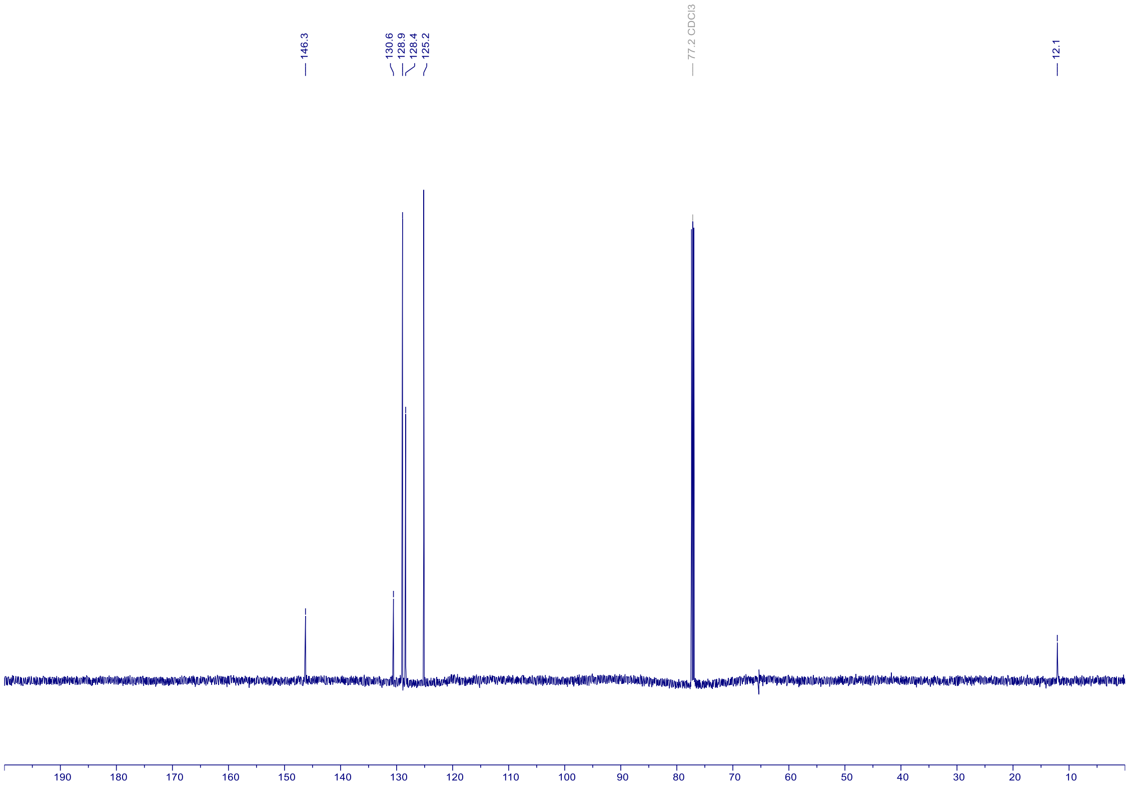

Methyl 4-Methyl-1*H*-imidazole-2-carboxylate (58b)

^{1}H NMR (600 MHz, $CDCl_3$)

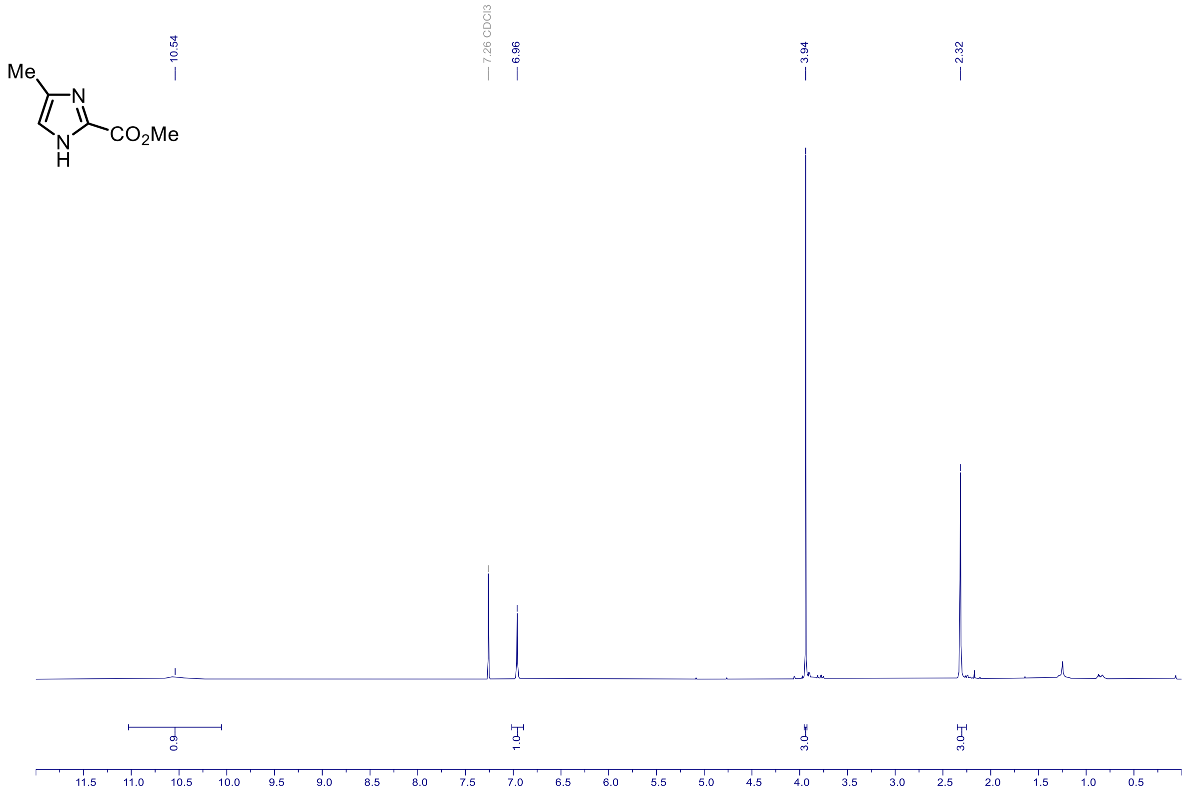

^{13}C NMR (151 MHz, $CDCl_3$)

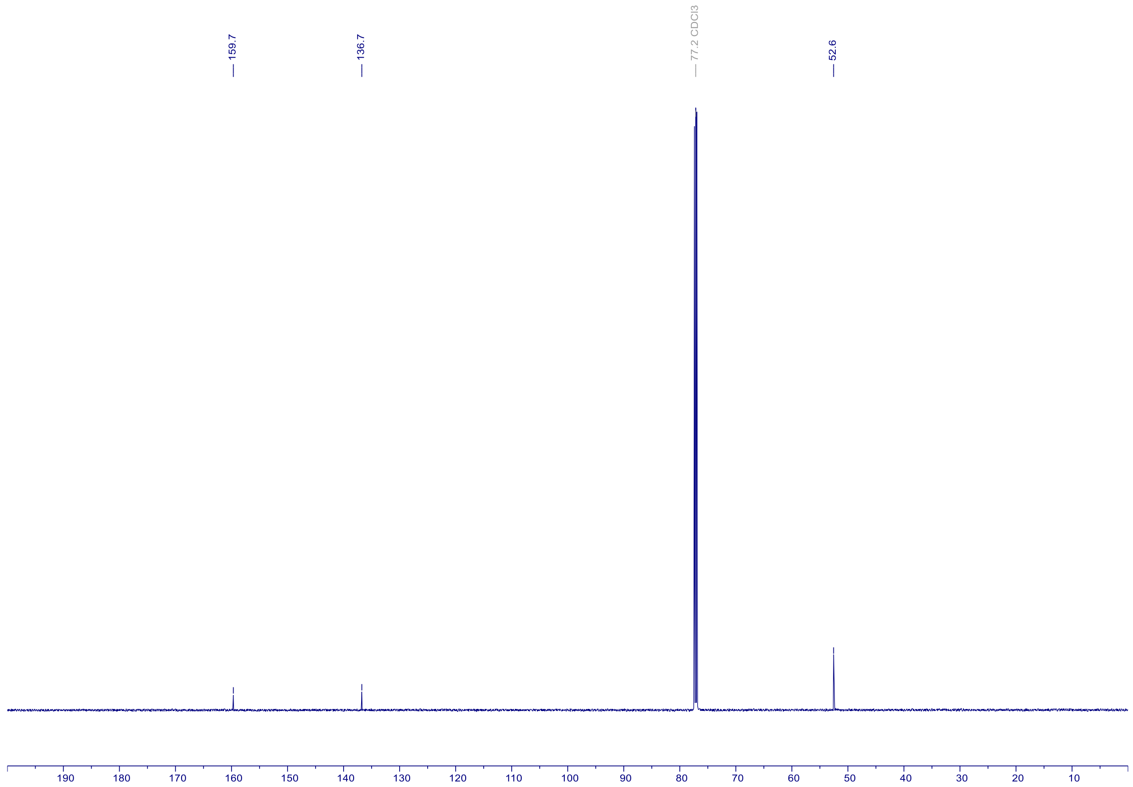

1-Phenyl-4,5,6,7-tetrahydro-1*H*-benzo[*d*]imidazole (62b)

^{1}H NMR (600 MHz, $CDCl_3$)

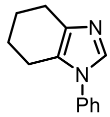

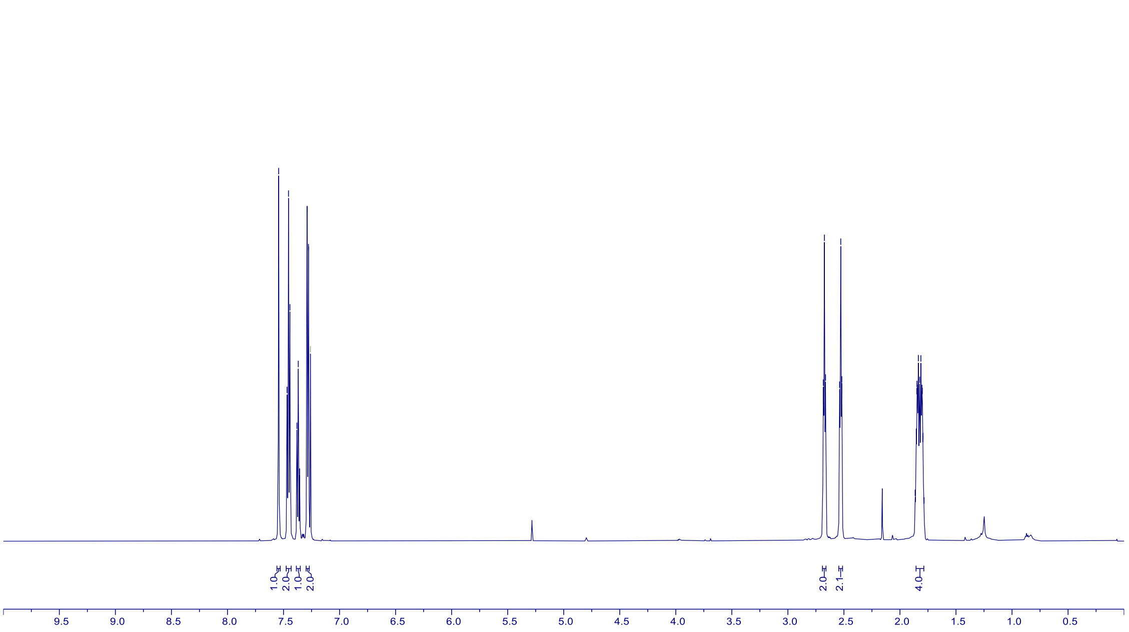

^{13}C NMR (151 MHz, $CDCl_3$)

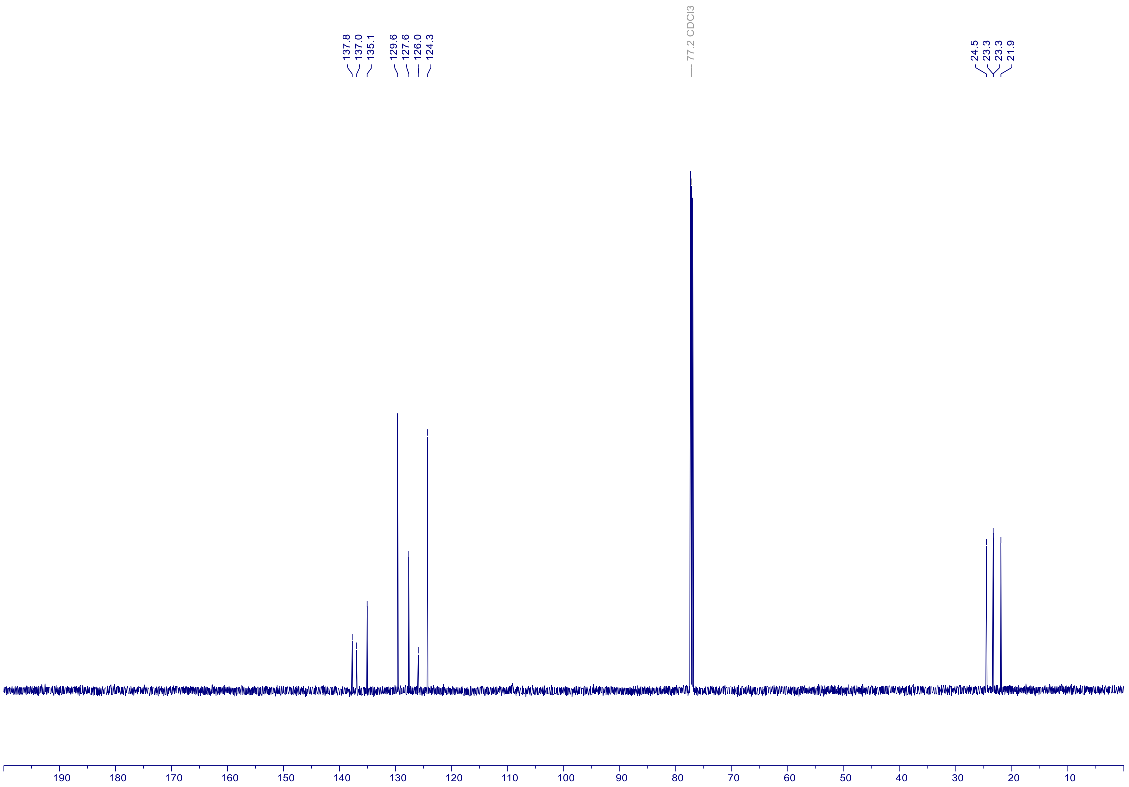

1-Phenyl-1,4,5,6-tetrahydrocyclopenta[*d*]imidazole (63b)

^{1}H NMR (600 MHz, $CDCl_3$)

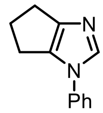

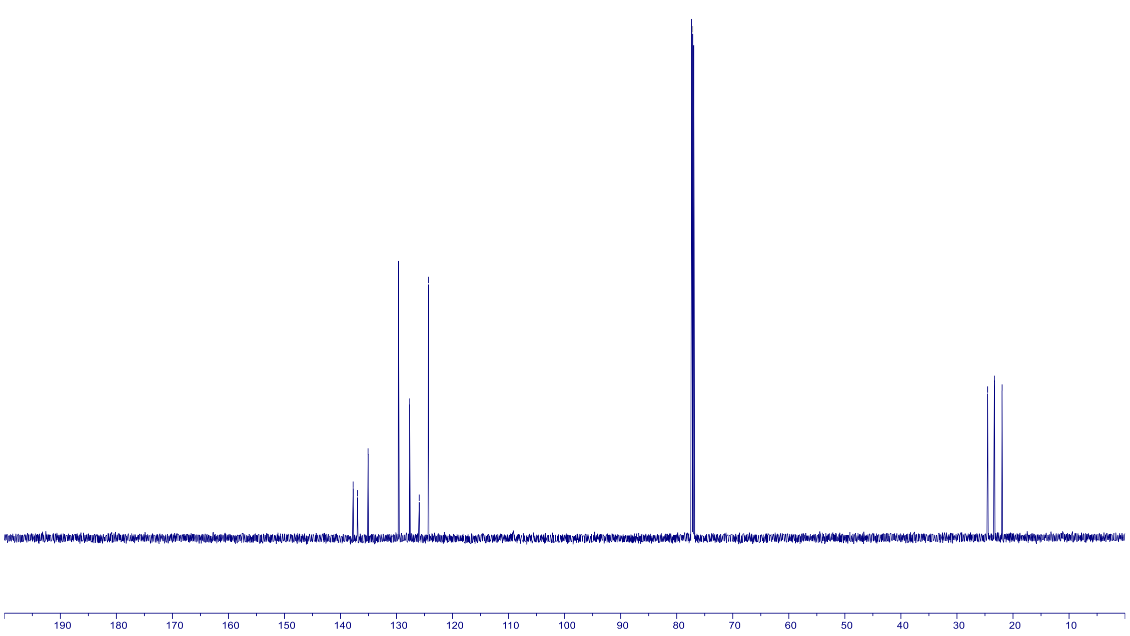

^{13}C NMR (151 MHz, $CDCl_3$)

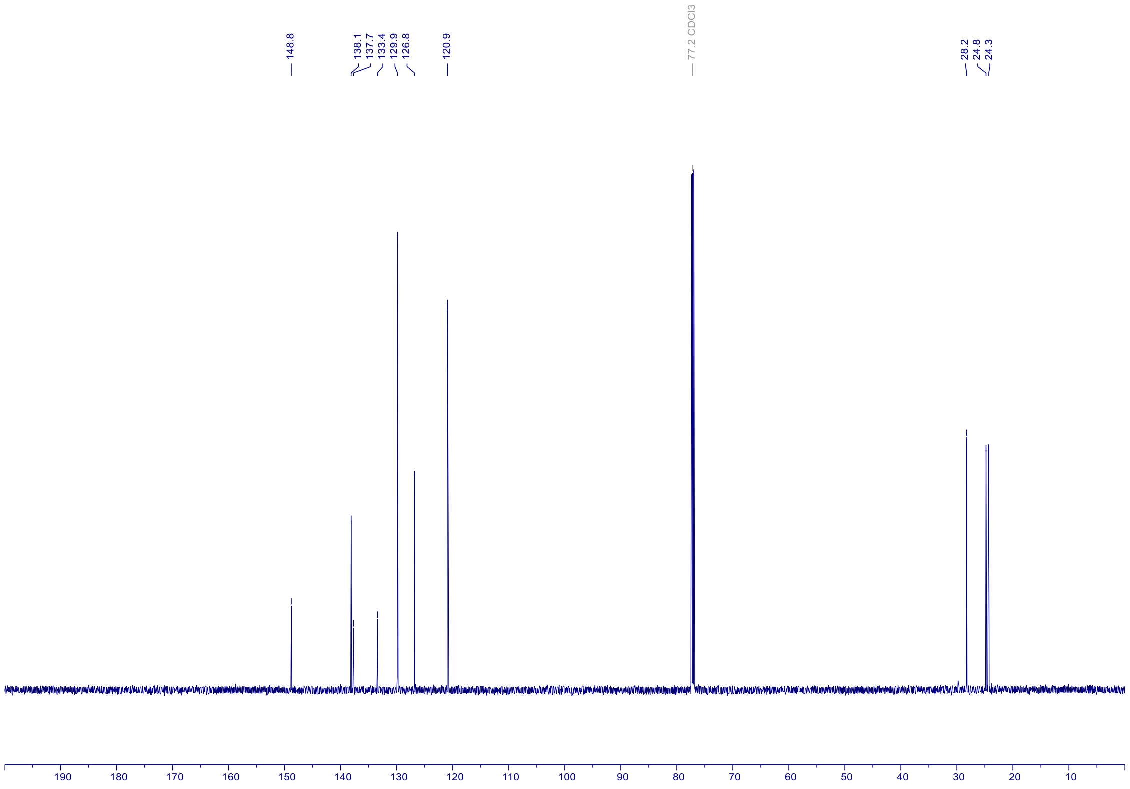

***tert*-Butyl 1-Phenyl-1,4,6,7-tetrahydro-5*H*-imidazo[4,5-*c*]pyridine-5-carboxylate (64b)**

^{1}H NMR (600 MHz, $CDCl_3$)

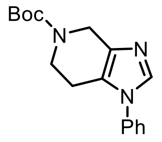

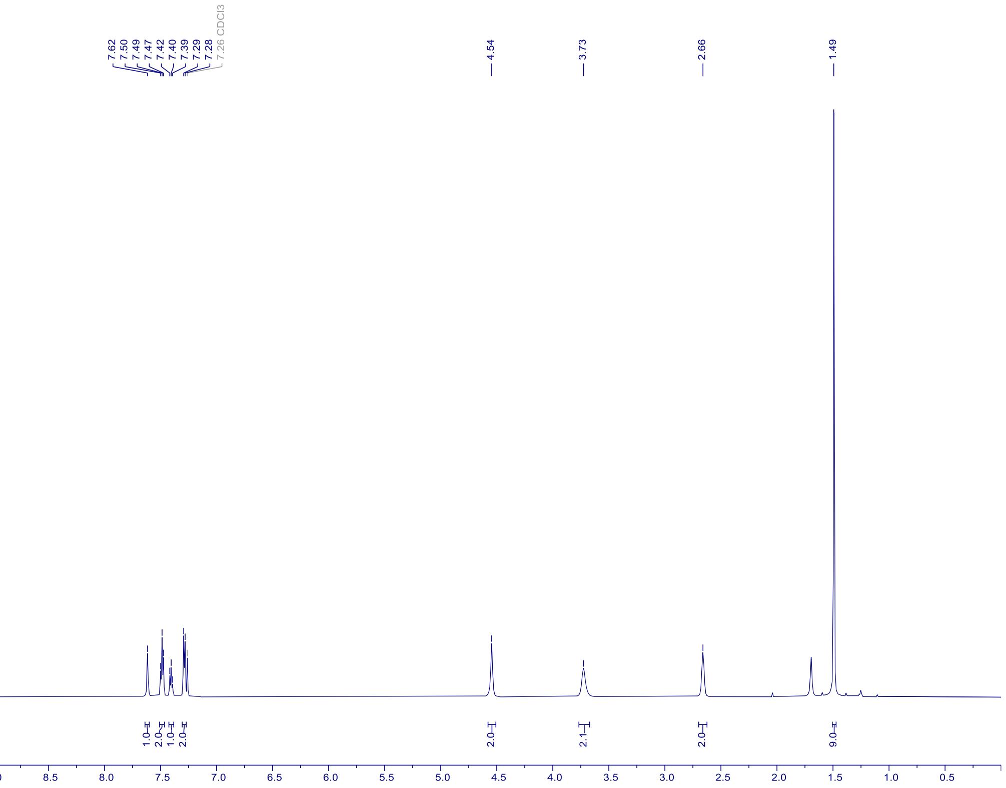

^{13}C NMR (151 MHz, $CDCl_3$)

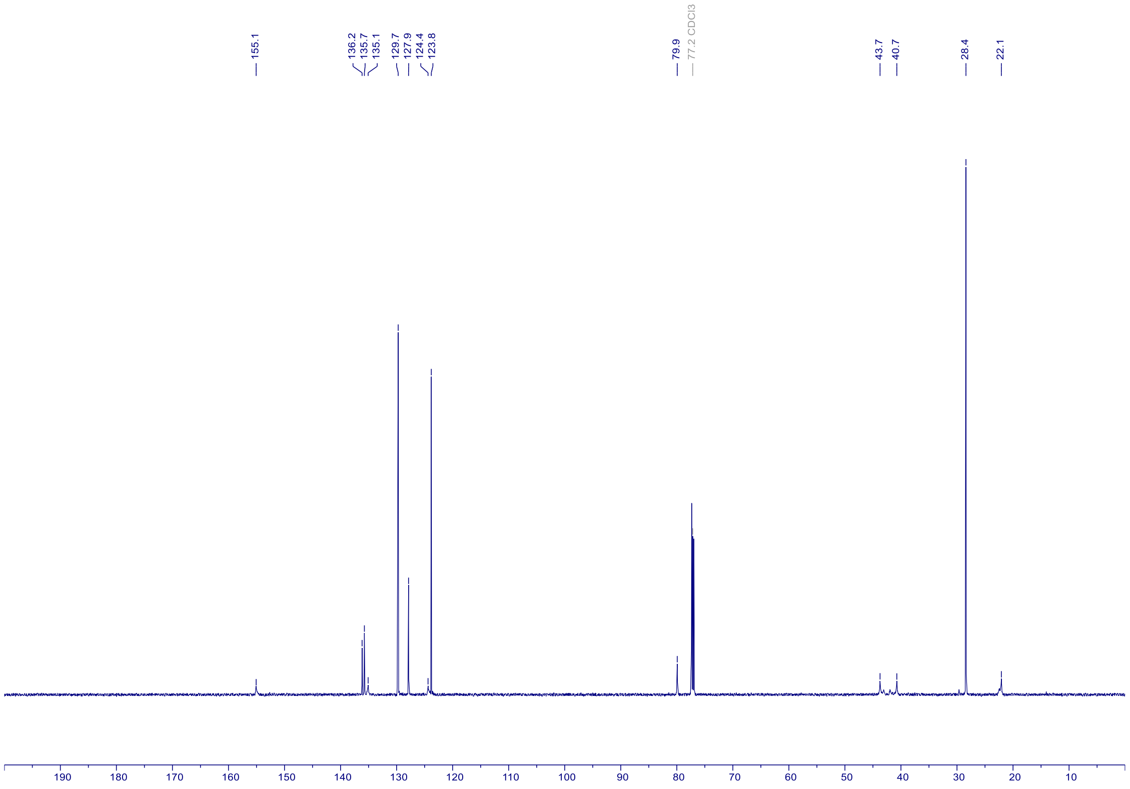

***tert*-Butyl 1-Phenyl-4,6-dihydropyrrolo[3,4-*d*]imidazole-5(1*H*)-carboxylate (65b)**

^{1}H NMR (600 MHz, $CDCl_3$)

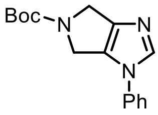

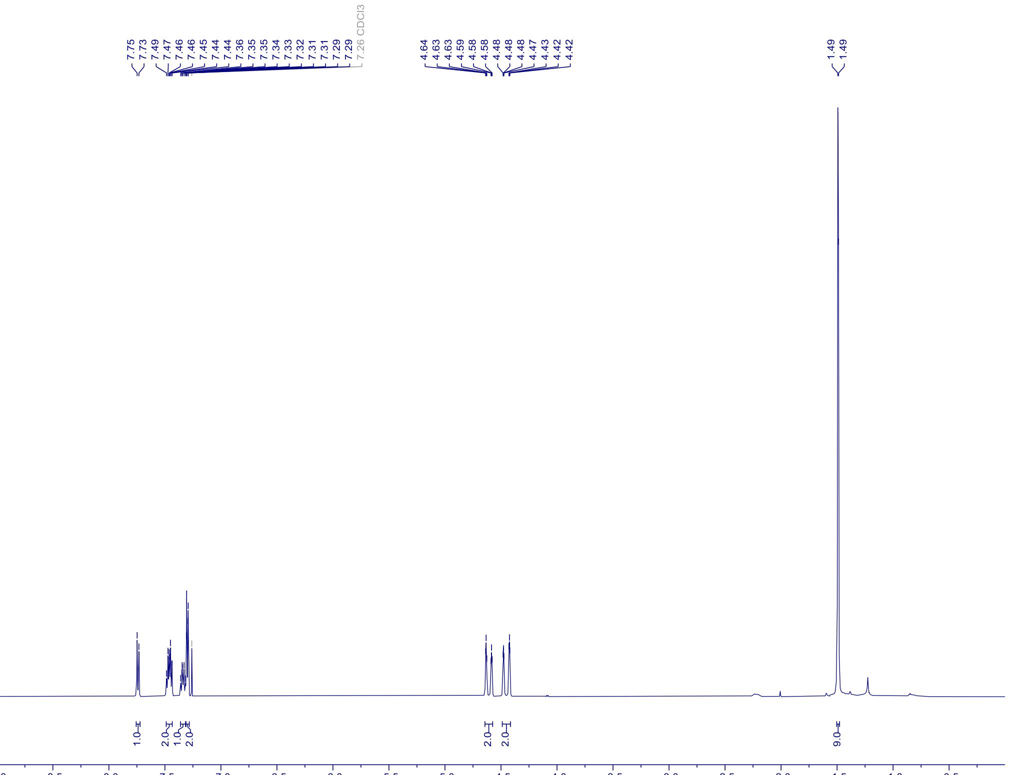

^{13}C NMR (151 MHz, $CDCl_3$)

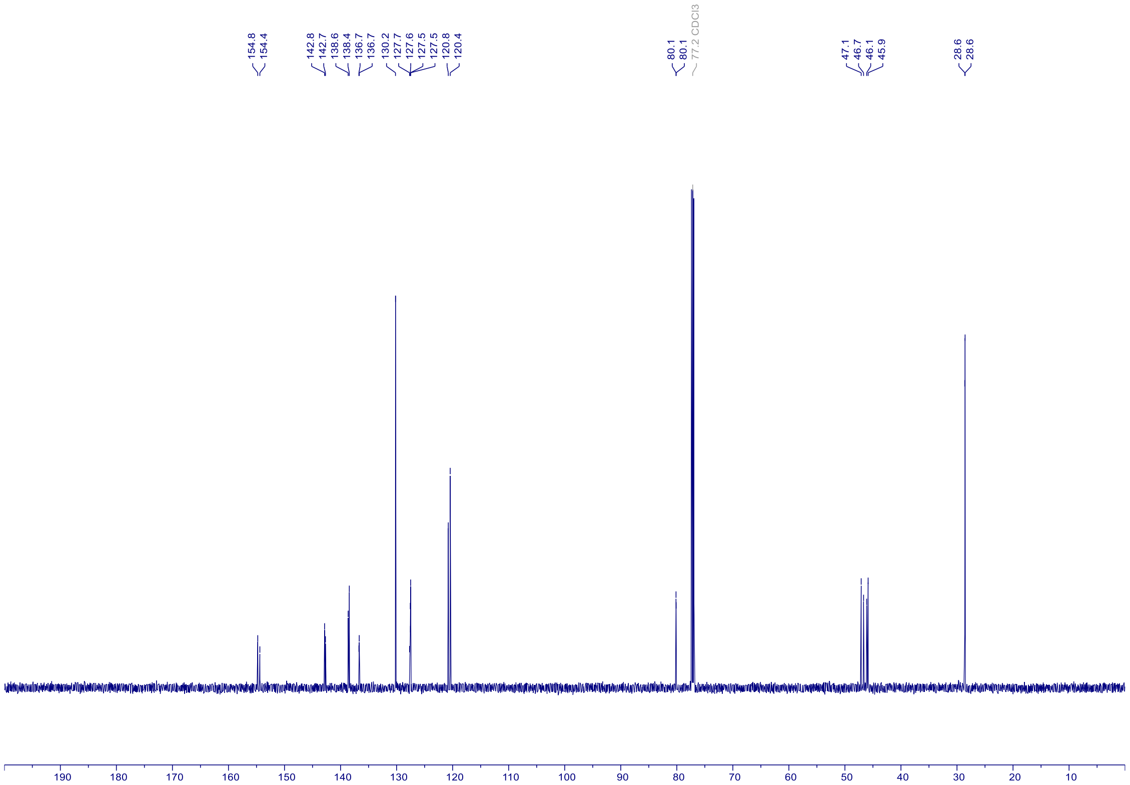

1-Phenyl-1,4,6,7-tetrahydropyrano[3,4-*d*]imidazole (66b)

^{1}H NMR (600 MHz, $CDCl_3$)

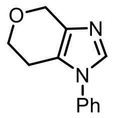

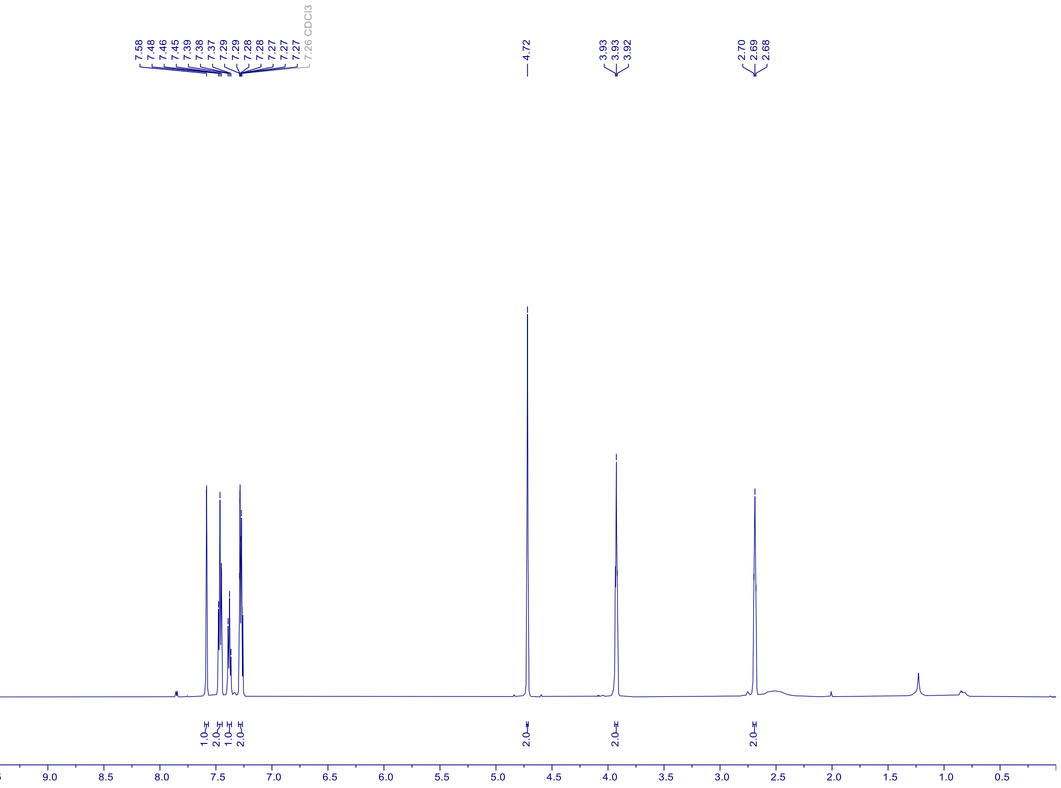

^{13}C NMR (151 MHz, $CDCl_3$)

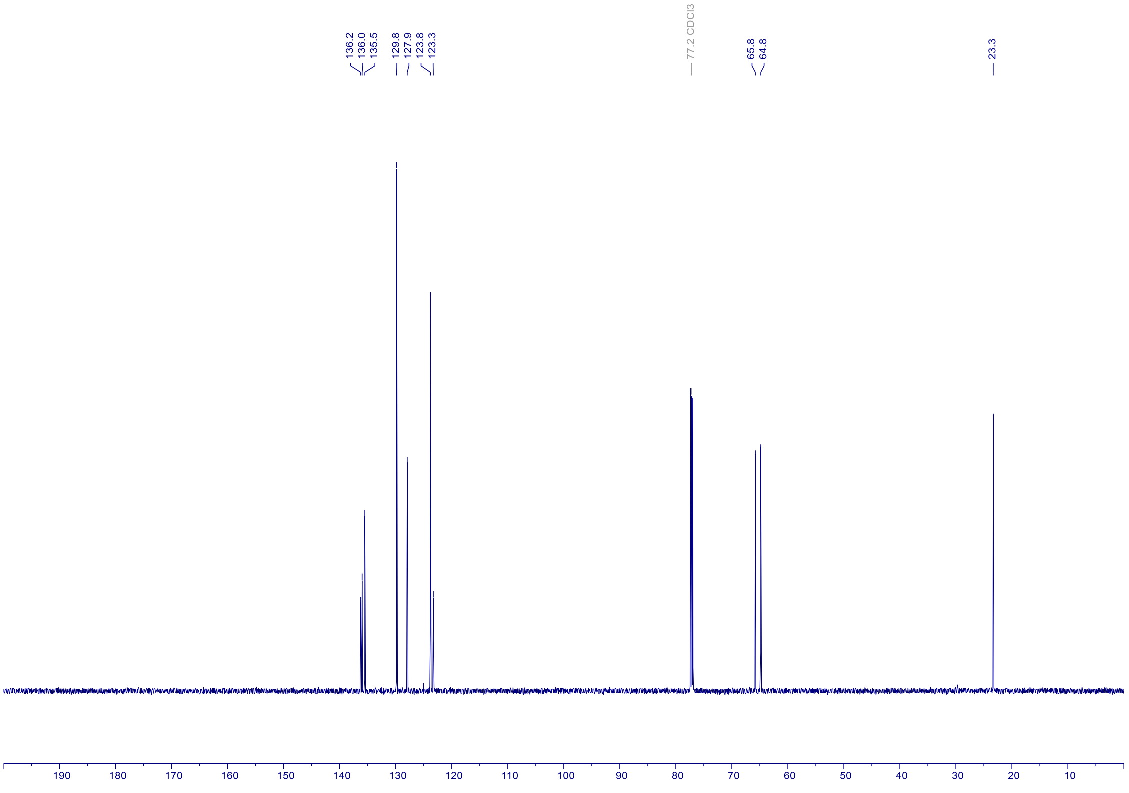

1-Phenyl-4,6-dihydro-1*H*-furo[3,4-*d*]imidazole (67b)

^{1}H NMR (600 MHz, CDCl$_3$)

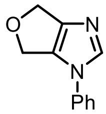

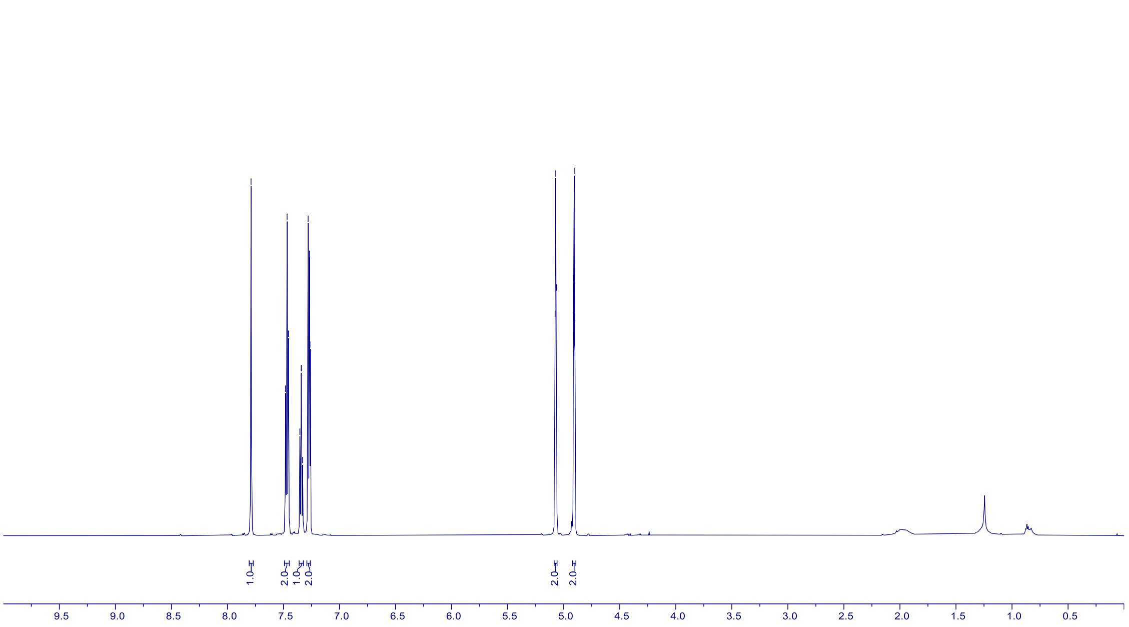

^{13}C NMR (151 MHz, CDCl$_3$)

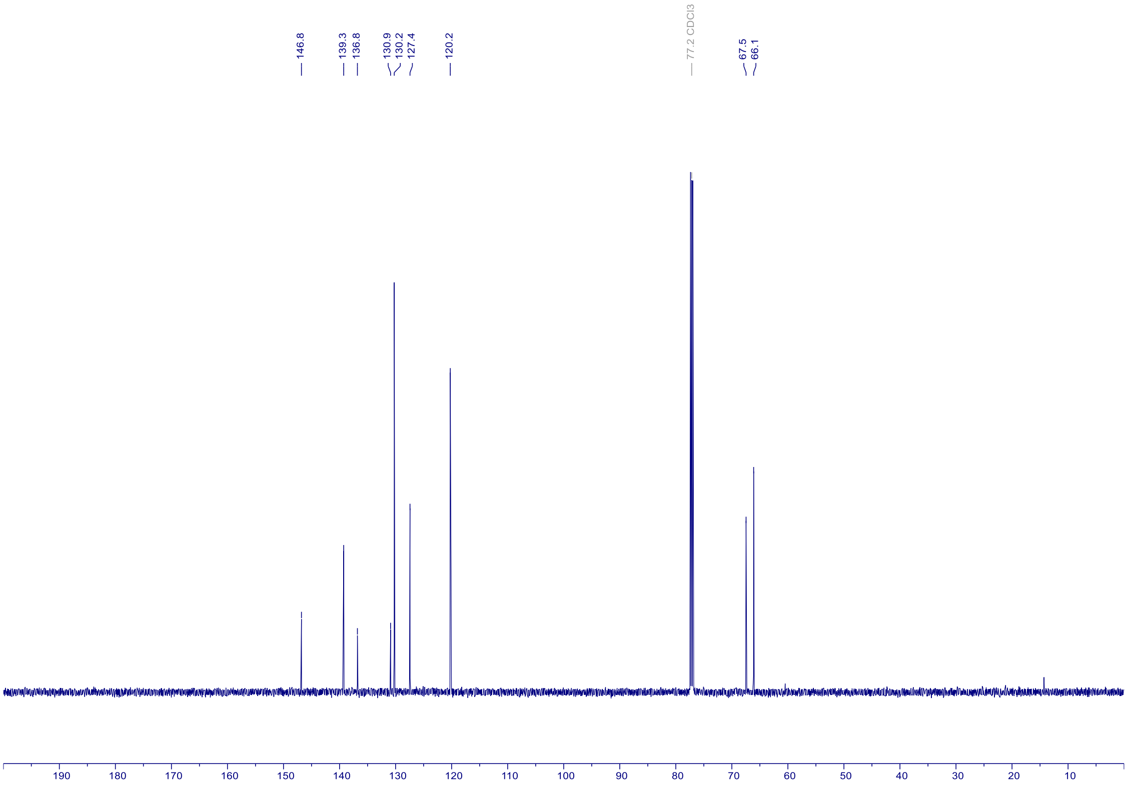

Ethyl 1-Phenyl-1,4,6,7-tetrahydropyrano[3,4-*d*]imidazole-2-carboxylate (68b)

^{1}H NMR (600 MHz, $CDCl_3$)

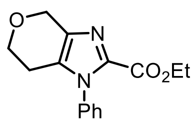

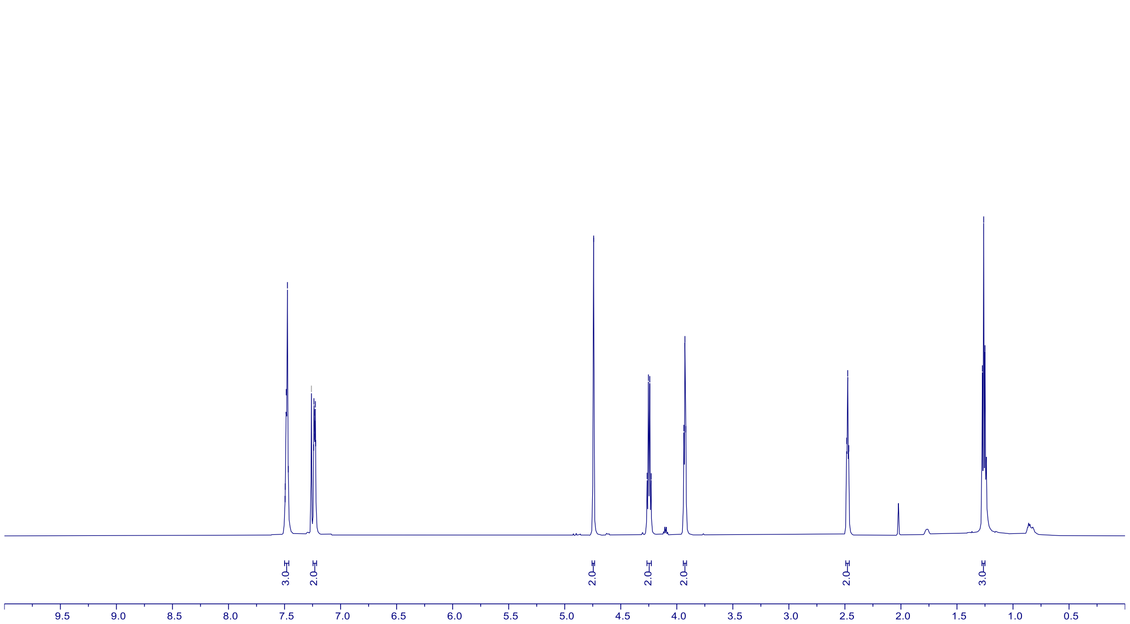

^{13}C NMR (151 MHz, $CDCl_3$)

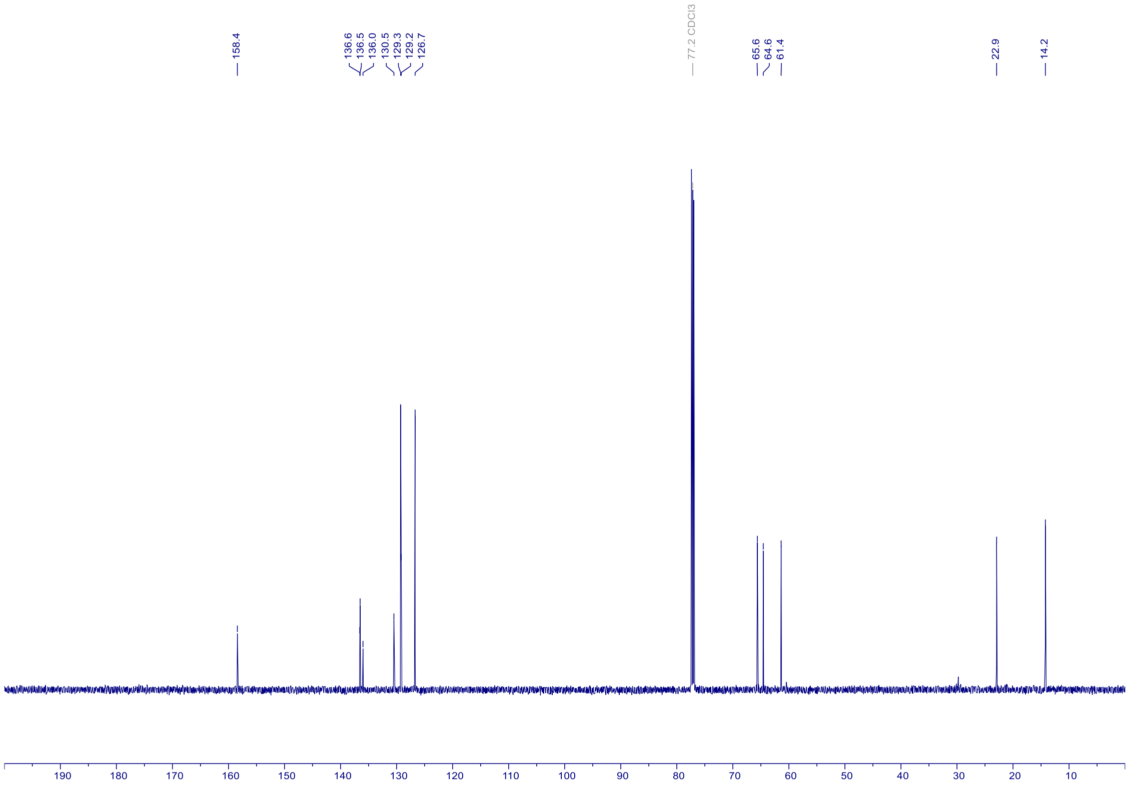

(1*S*,3a*S*,3b*R*,10a*S*,10b*S*,12a*S*)-1,10a,12a-Trimethyl-1,2,3,3a,3b,4,5,5a,6,7,10,10a,10b,11,12,12a-hexadecahydrocyclopenta[7,8]phenanthro[2,3-*d*]imidazol-1-ol (69b)

^{1}H NMR (600 MHz, $CDCl_3$)

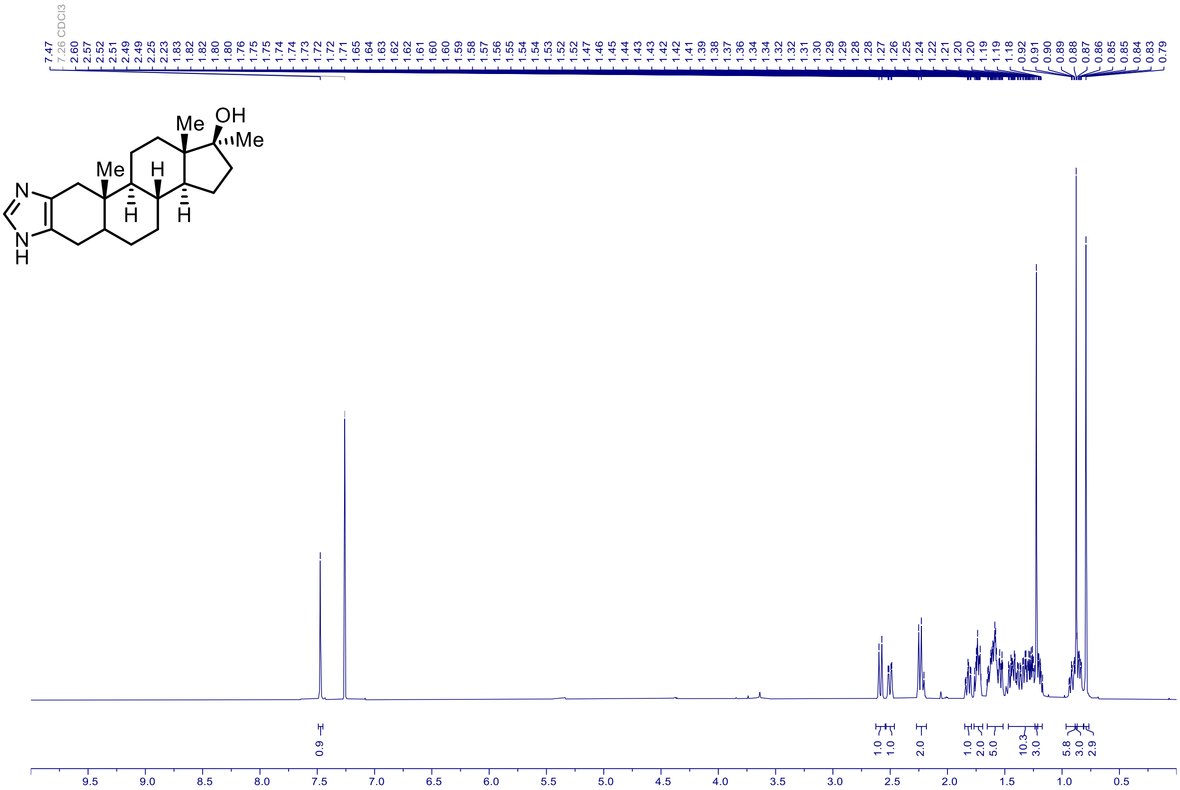

^{13}C NMR (151 MHz, $CDCl_3$)

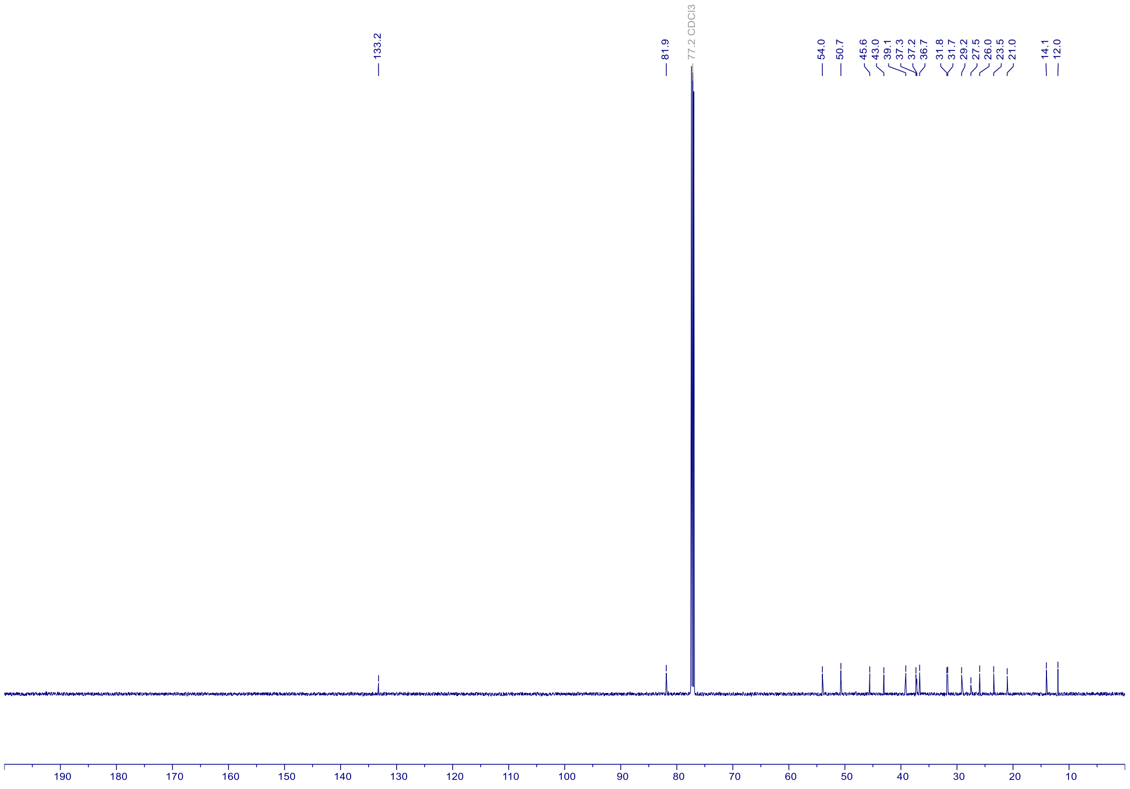

1-Phenyl-6,7-dihydro-5*H*-pyrrolo[1,2-*c*]imidazole (70b)

^{1}H NMR (600 MHz, $CDCl_3$)

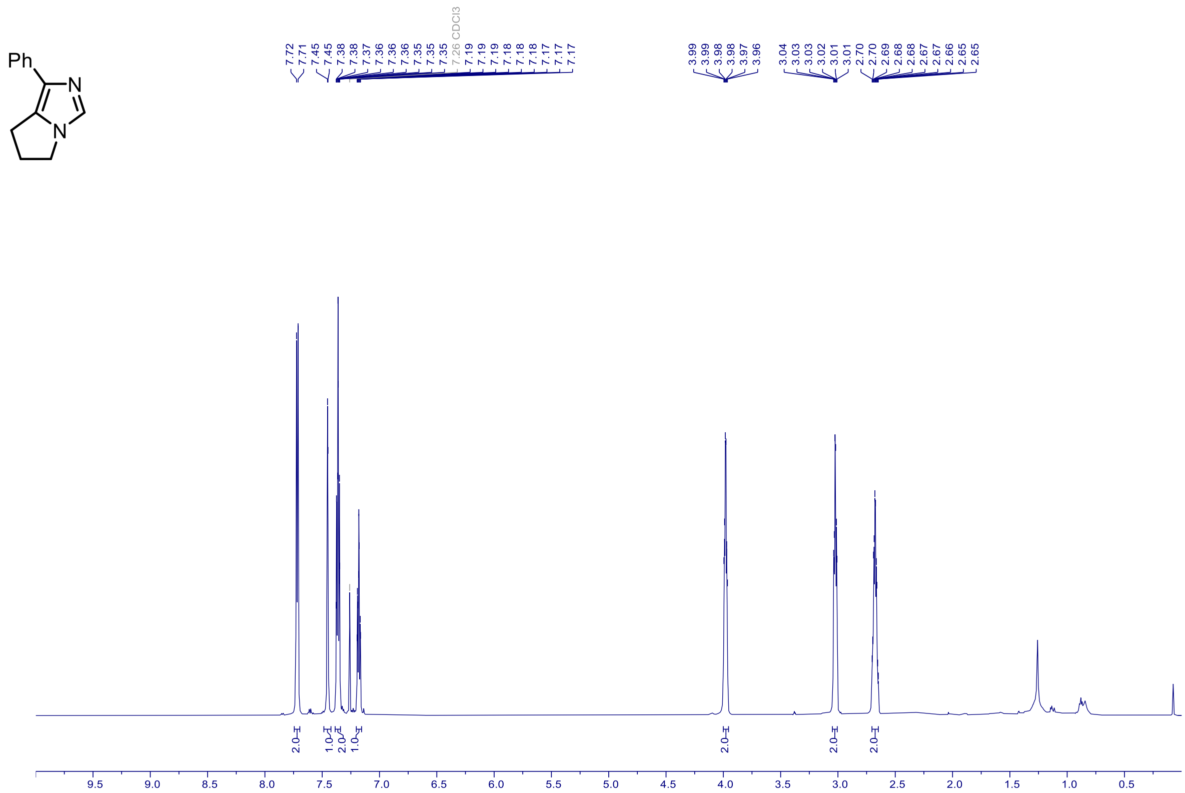

^{13}C NMR (151 MHz, $CDCl_3$)

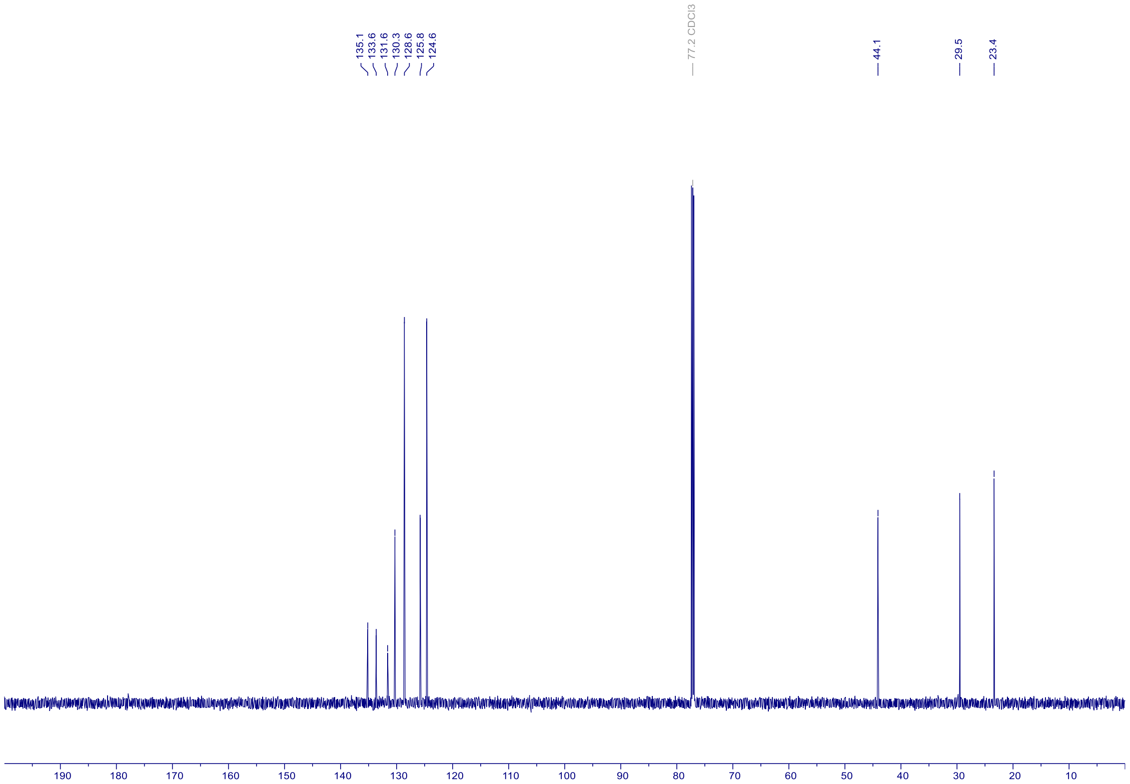

Methyl Imidazo[1,5-*a*]pyridine-1-carboxylate (74b)

^{1}H NMR (600 MHz, $CDCl_3$)

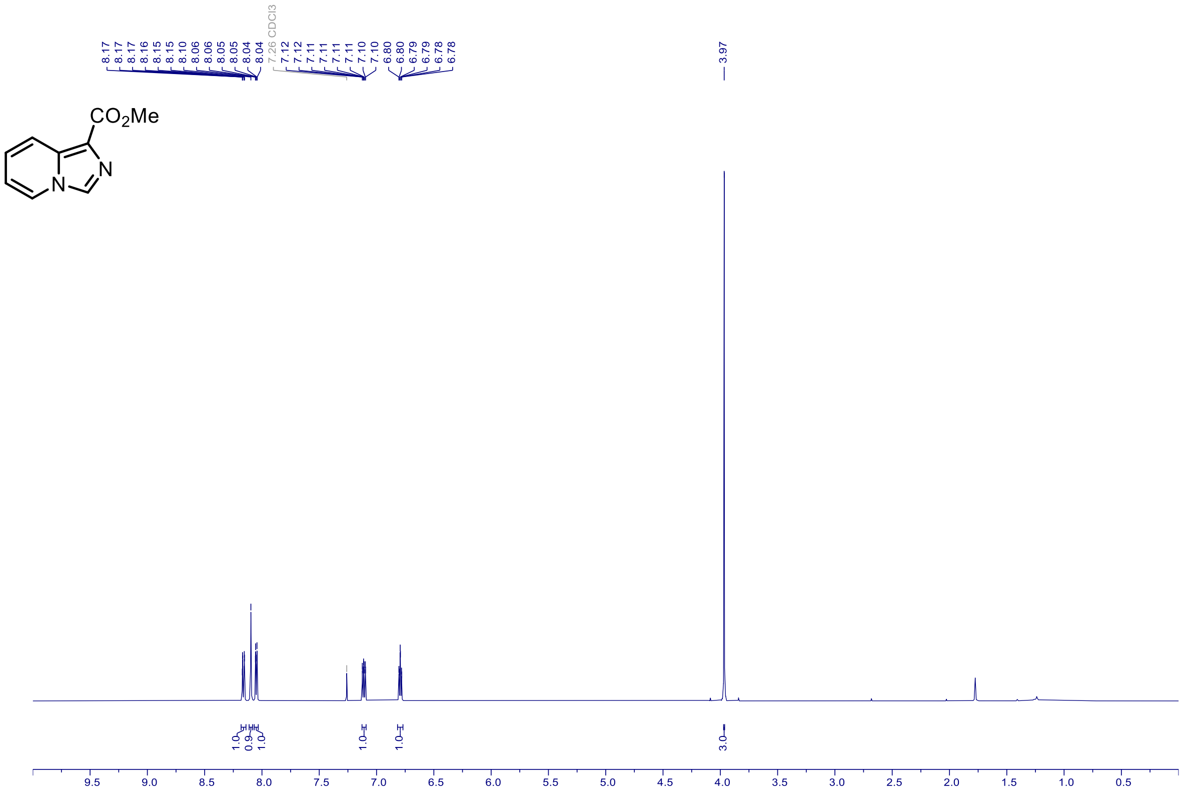

^{13}C NMR (151 MHz, $CDCl_3$)

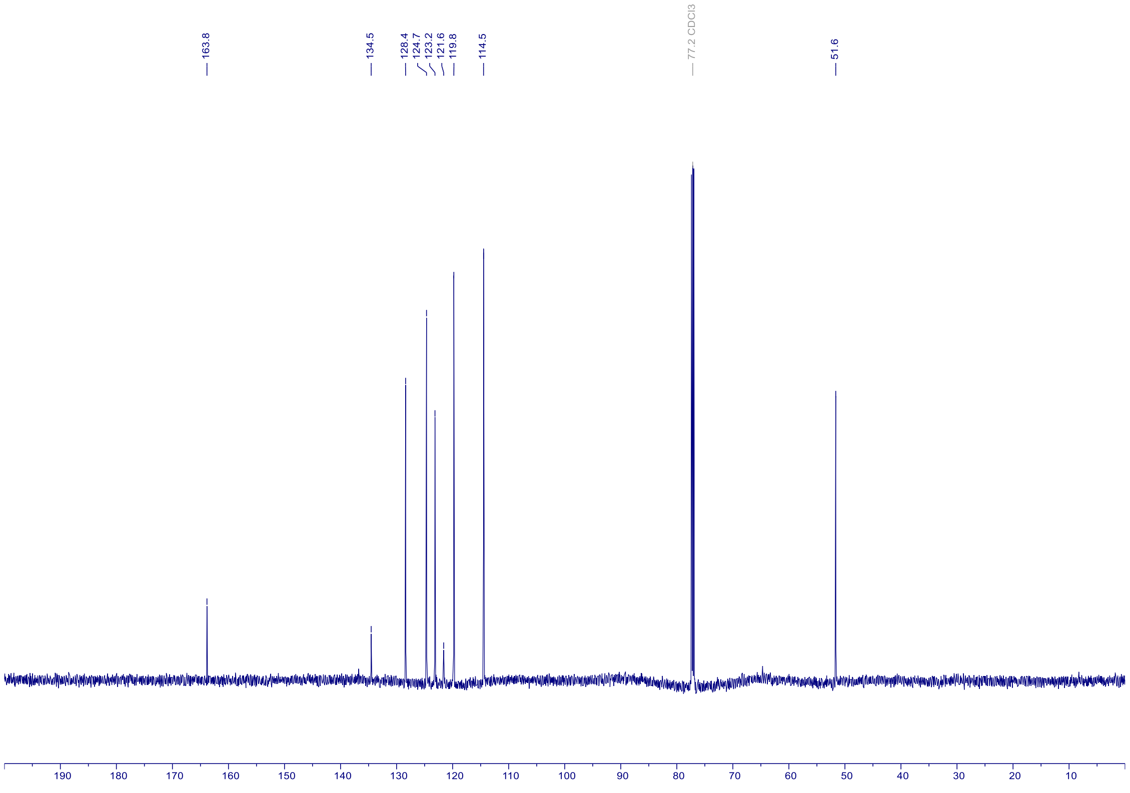

Methyl Imidazo[1,5-*a*]pyridine-3-carboxylate (75b)

^{1}H NMR (600 MHz, $CDCl_3$)

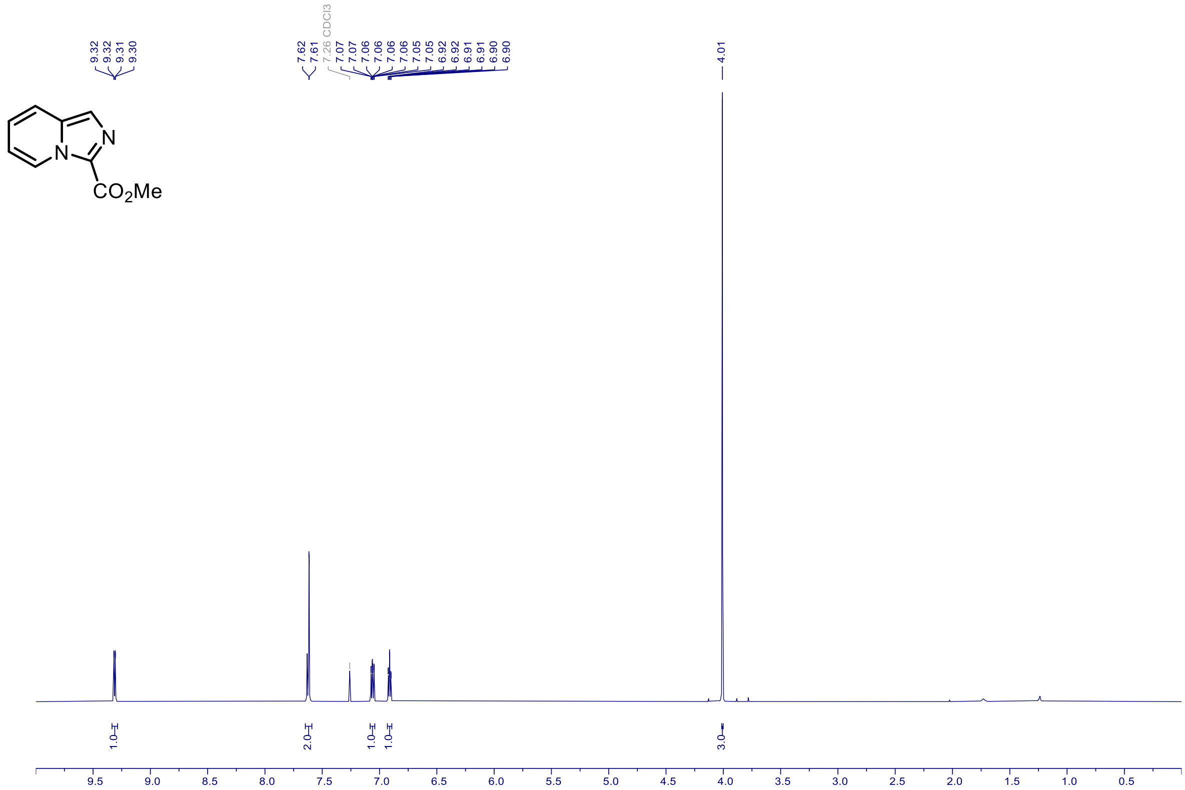

^{13}C NMR (151 MHz, $CDCl_3$)

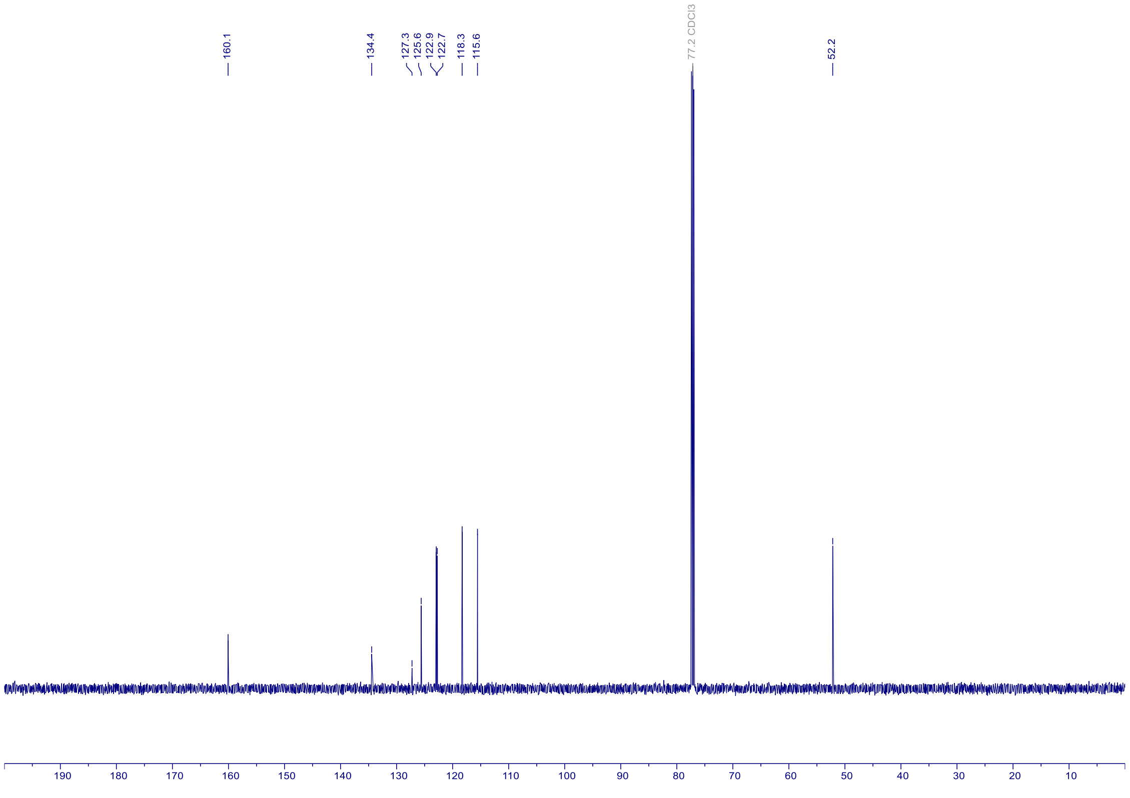

Ethyl Imidazo[1,5-*a*]pyrimidine-8-carboxylate (77b)

^{1}H NMR (600 MHz, $CDCl_3$)

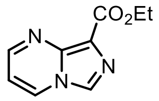

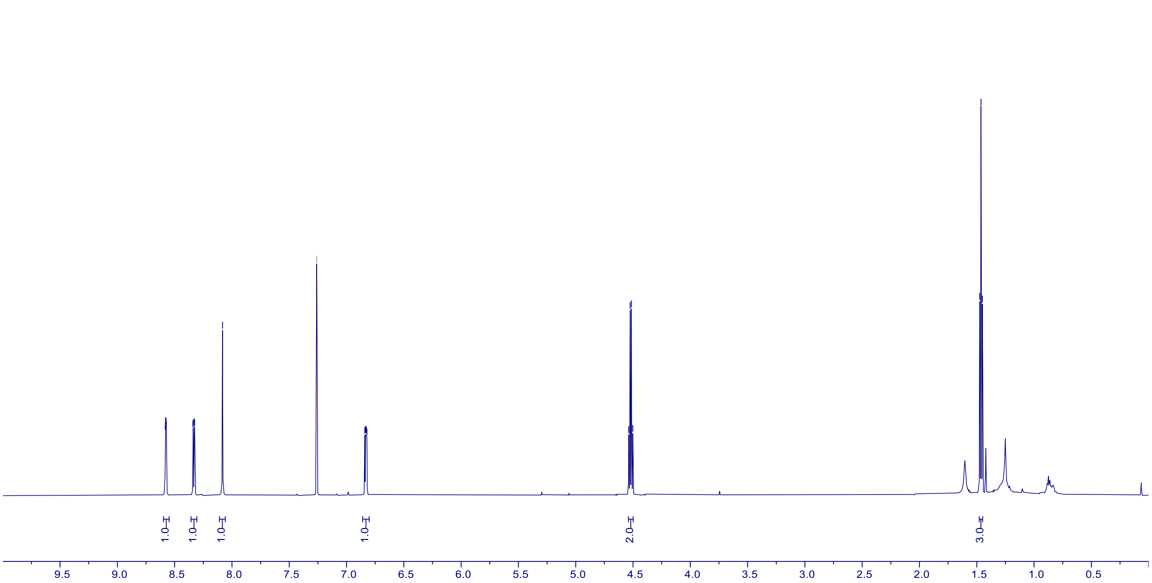

^{13}C NMR (151 MHz, $CDCl_3$)

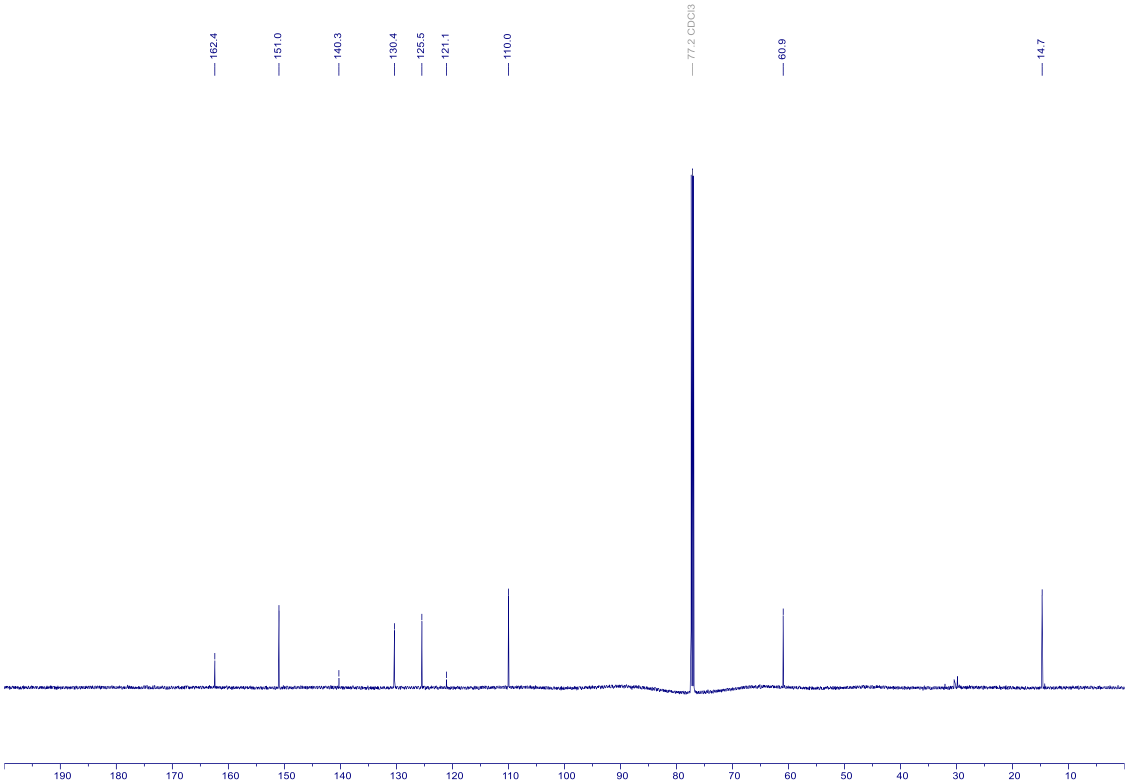

Imidazo[1,5-*a*]pyrimidine-8-carbonitrile (78b)

^{1}H NMR (600 MHz, CD_3OD)

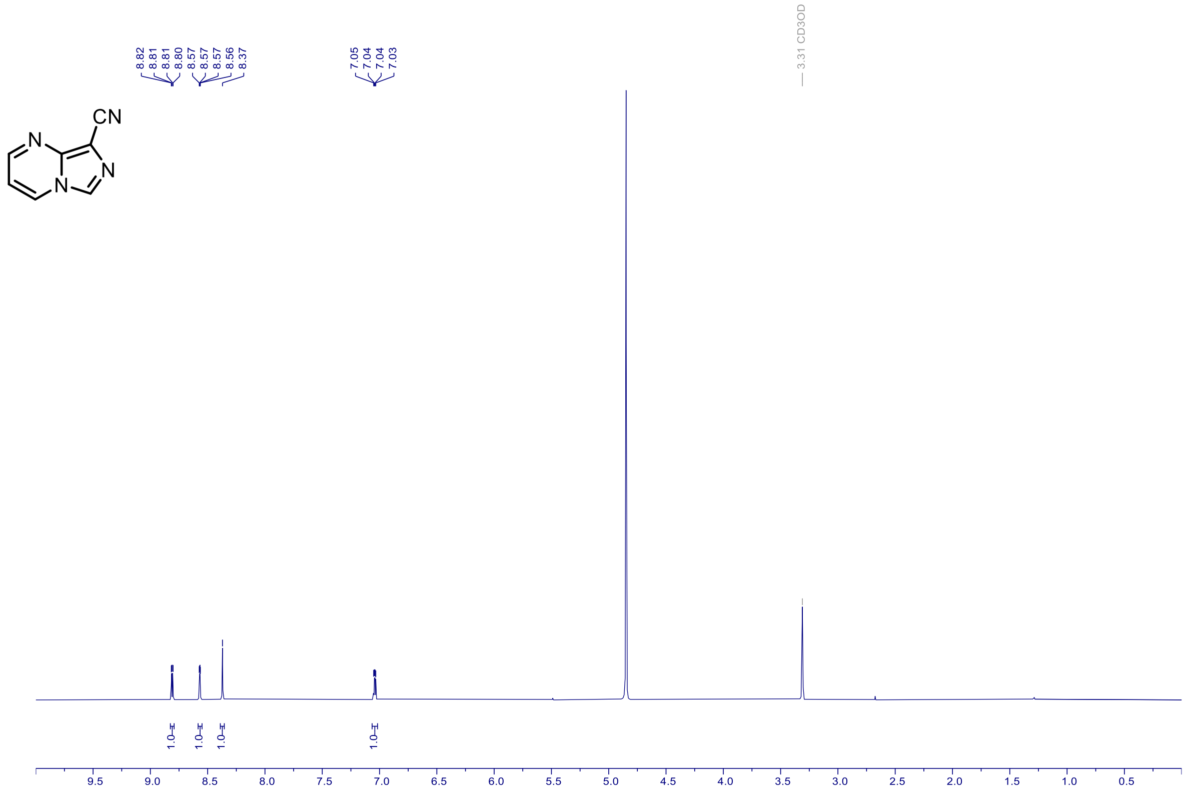

^{13}C NMR (151 MHz, CD_3OD)

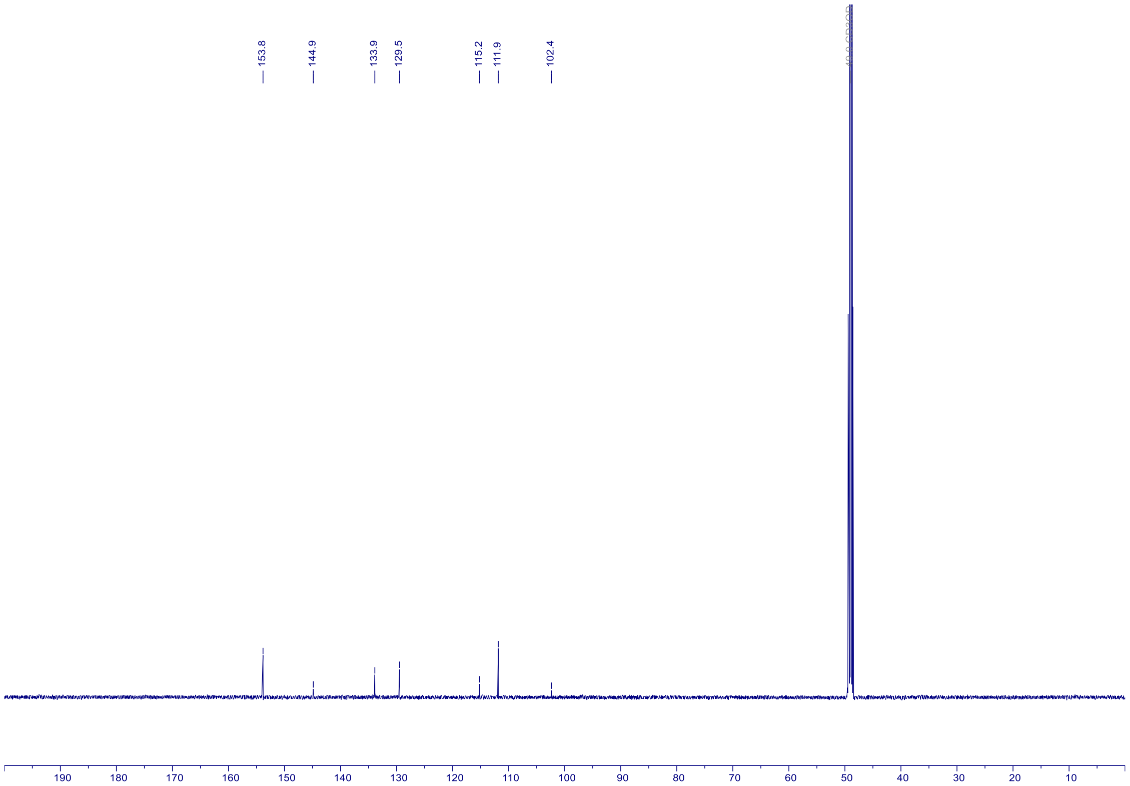

2,4-Dichloro-8-ethylimidazo[1,5-*a*]pyrimidine (79b)

^{1}H NMR (600 MHz, $CDCl_3$)

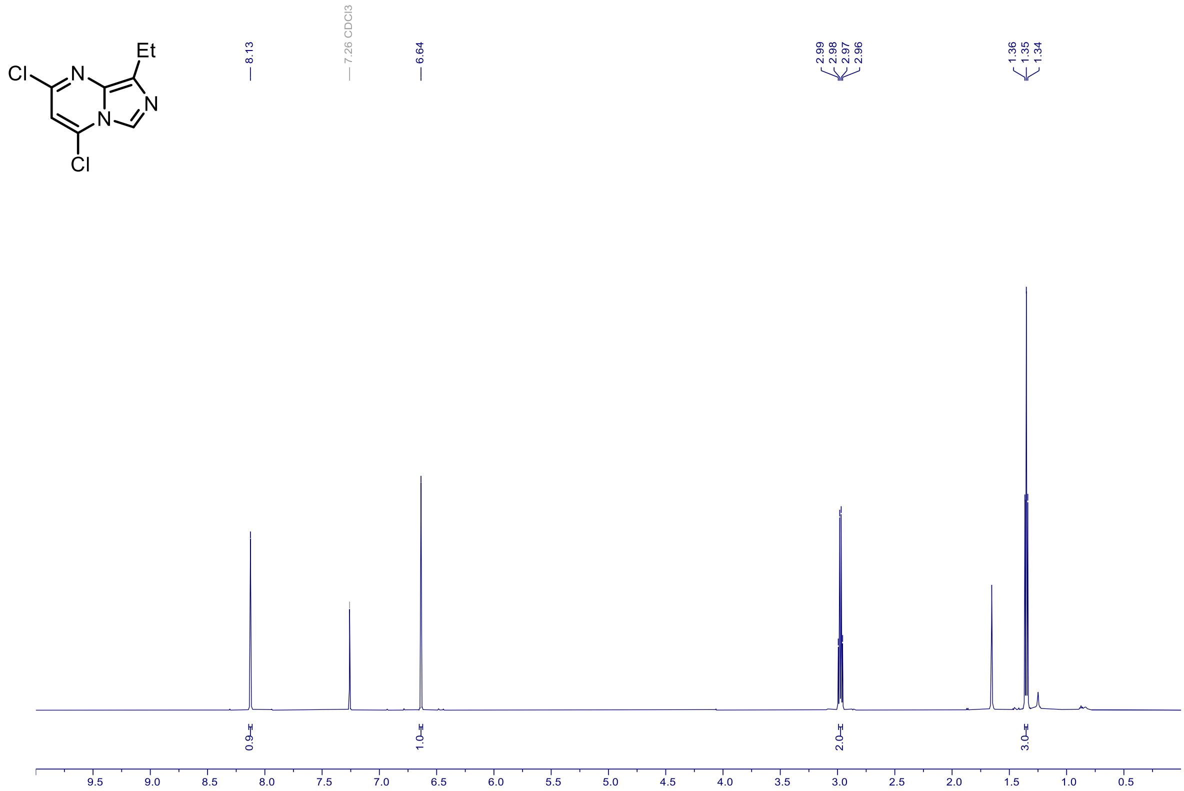

^{13}C NMR (151 MHz, $CDCl_3$)

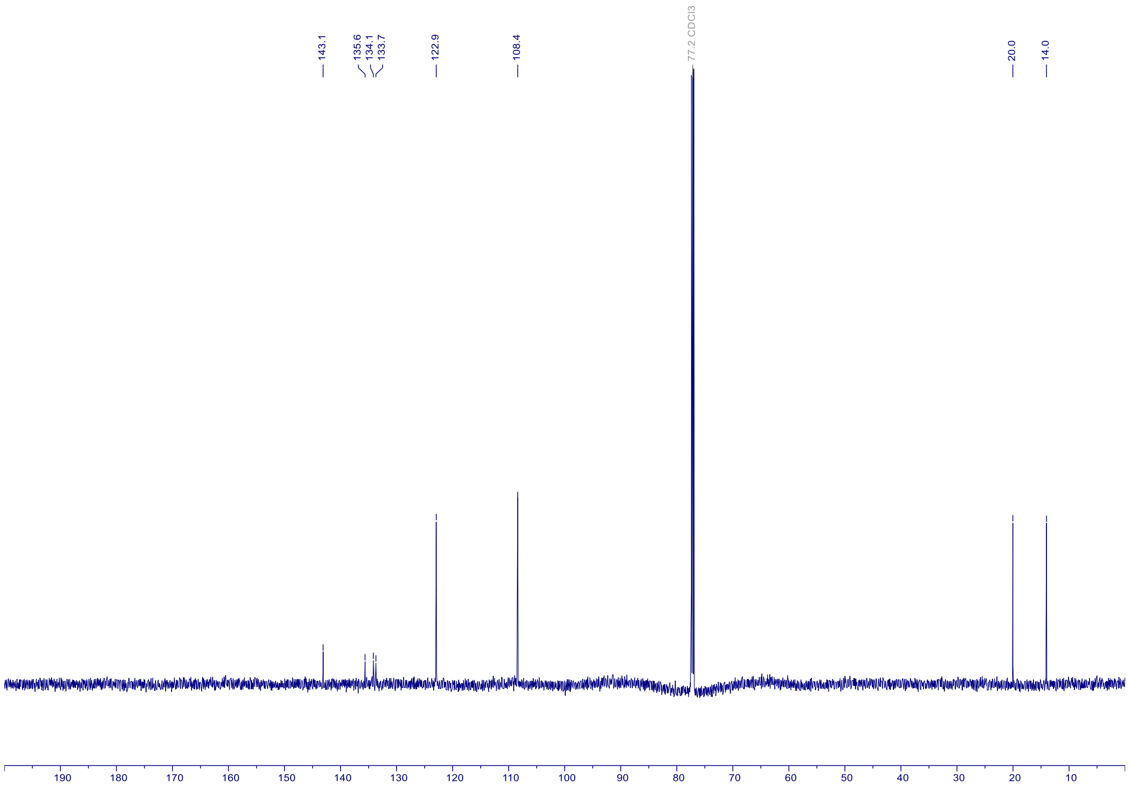

Methyl Imidazo[1,5-*a*]pyrazine-1-carboxylate (80b)

^{1}H NMR (600 MHz, $CDCl_3$)

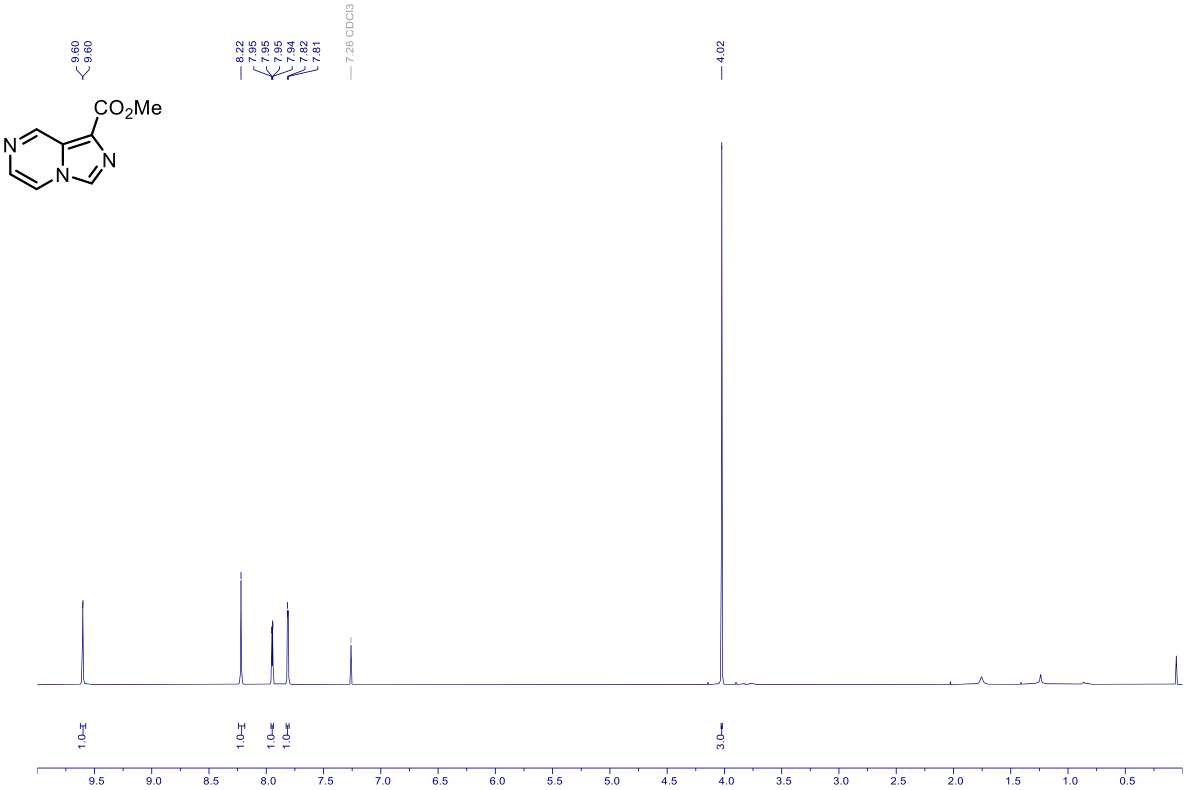

^{13}C NMR (151 MHz, $CDCl_3$)

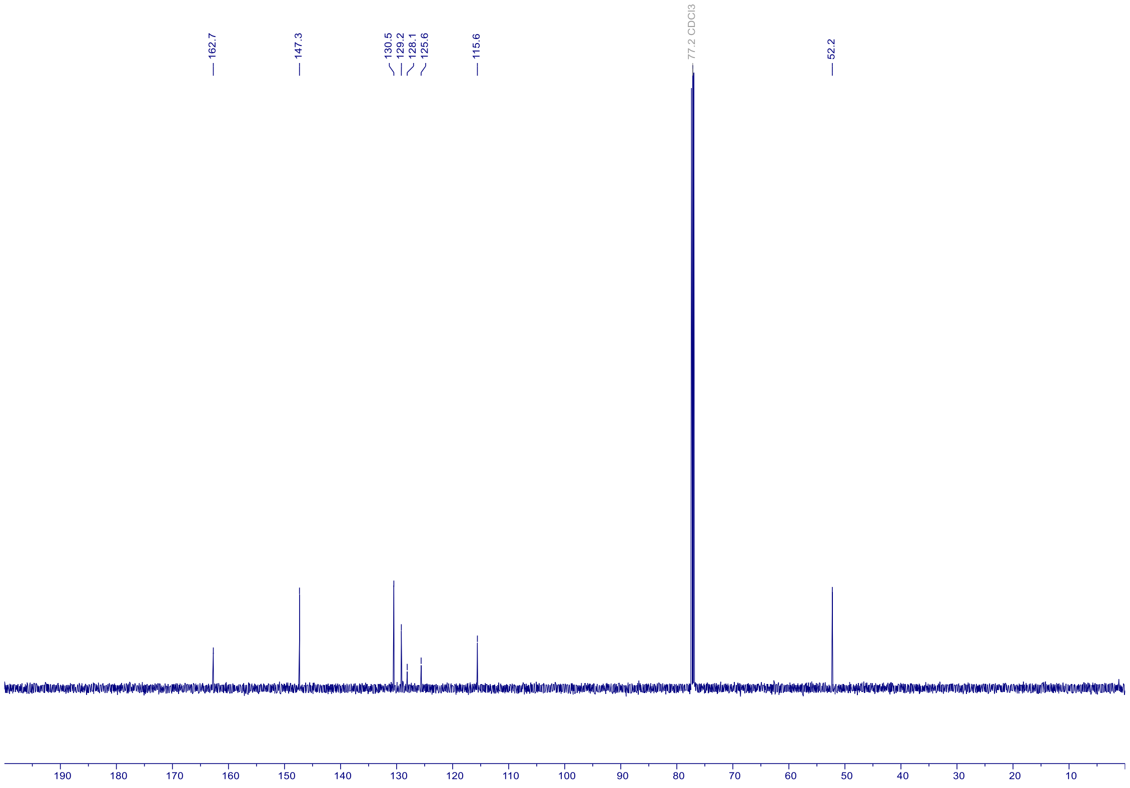

Methyl Imidazo[1,5-*b*]pyridazine-5-carboxylate (81b)

^{1}H NMR (600 MHz, $CDCl_3$)

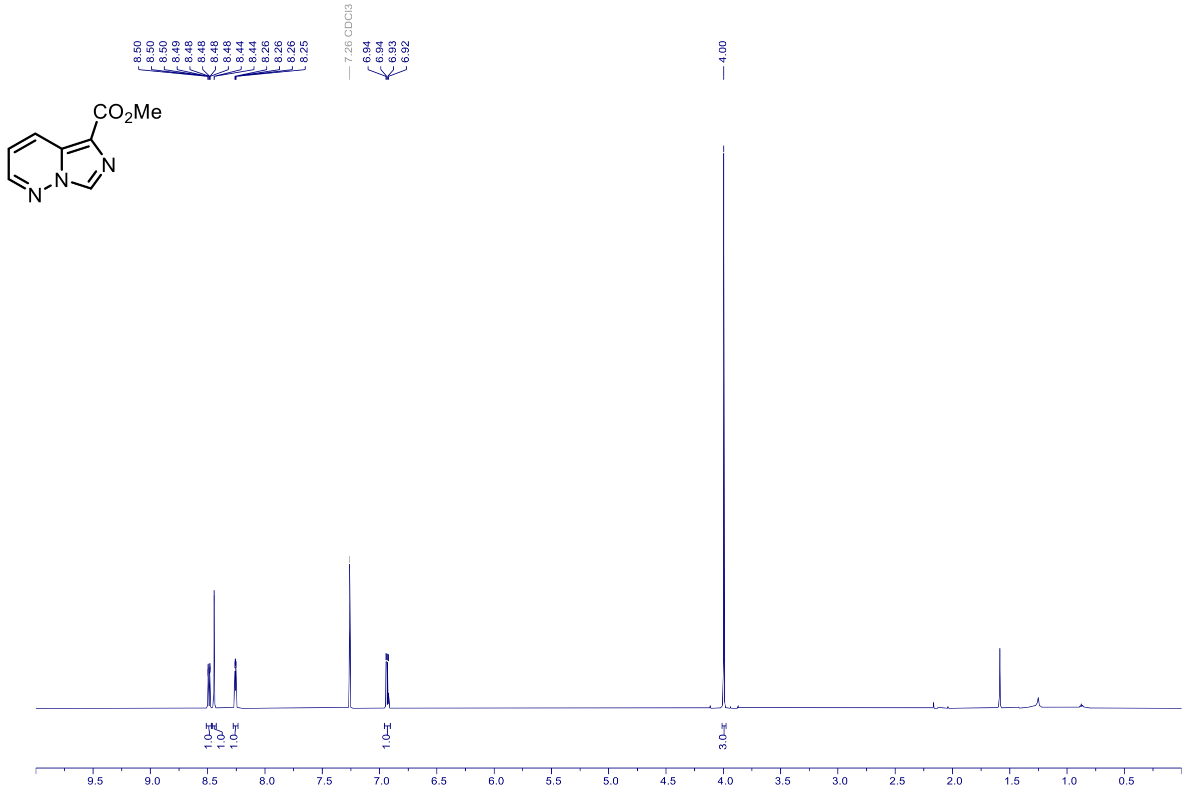

^{13}C NMR (151 MHz, $CDCl_3$)

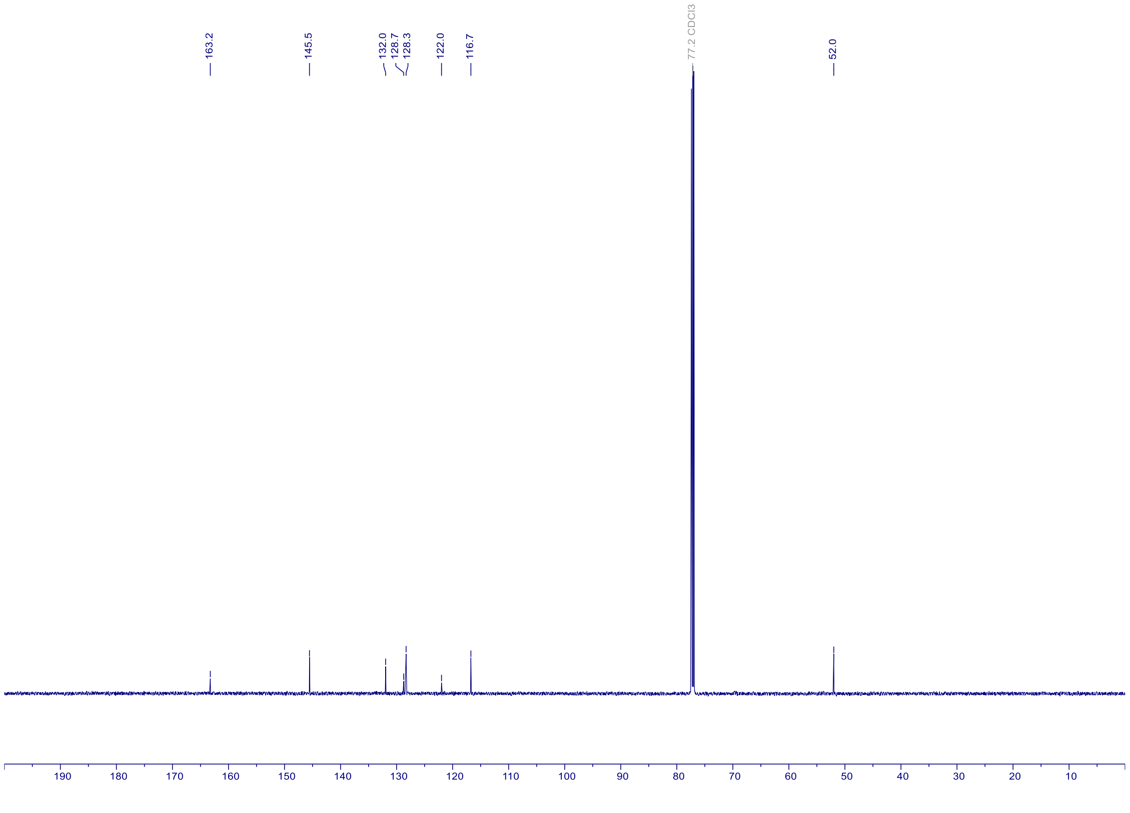

***N*-(3-(8-Cyanoimidazo[1,5-*a*]pyrimidin-4-yl)phenyl)-*N*-ethylacetamide (82b)**

^{1}H NMR (600 MHz, $CDCl_3$)

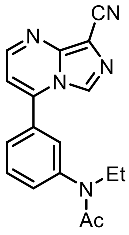

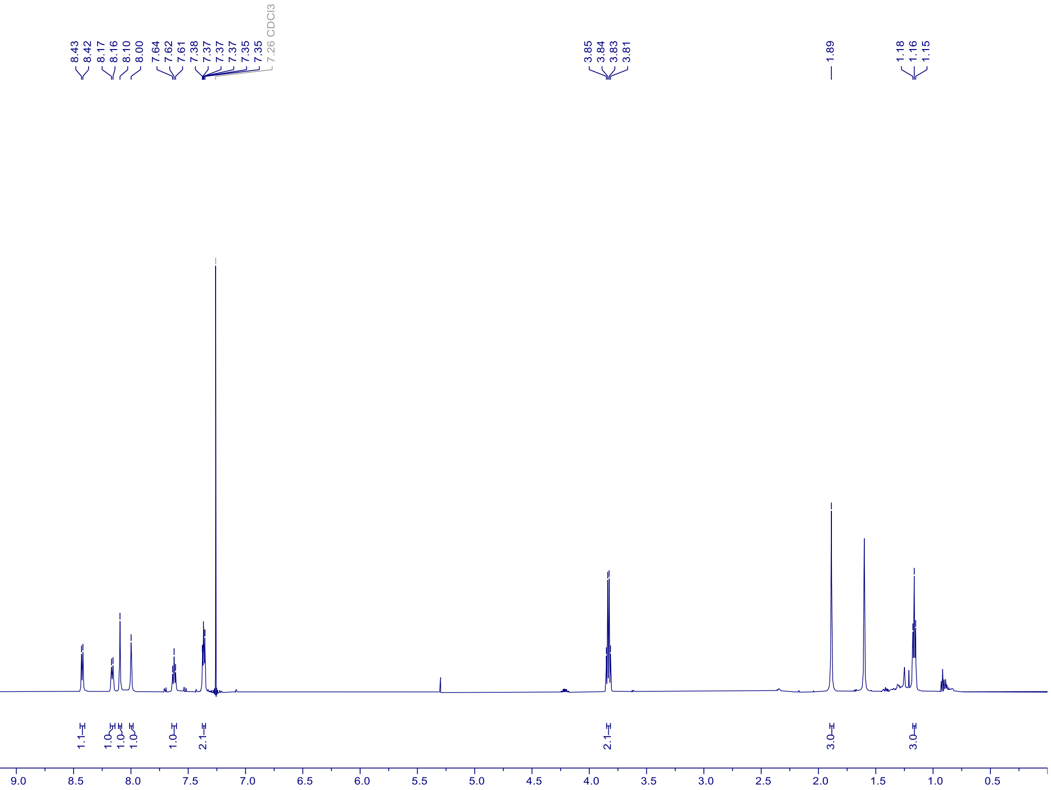

^{13}C NMR (151 MHz, $CDCl_3$)

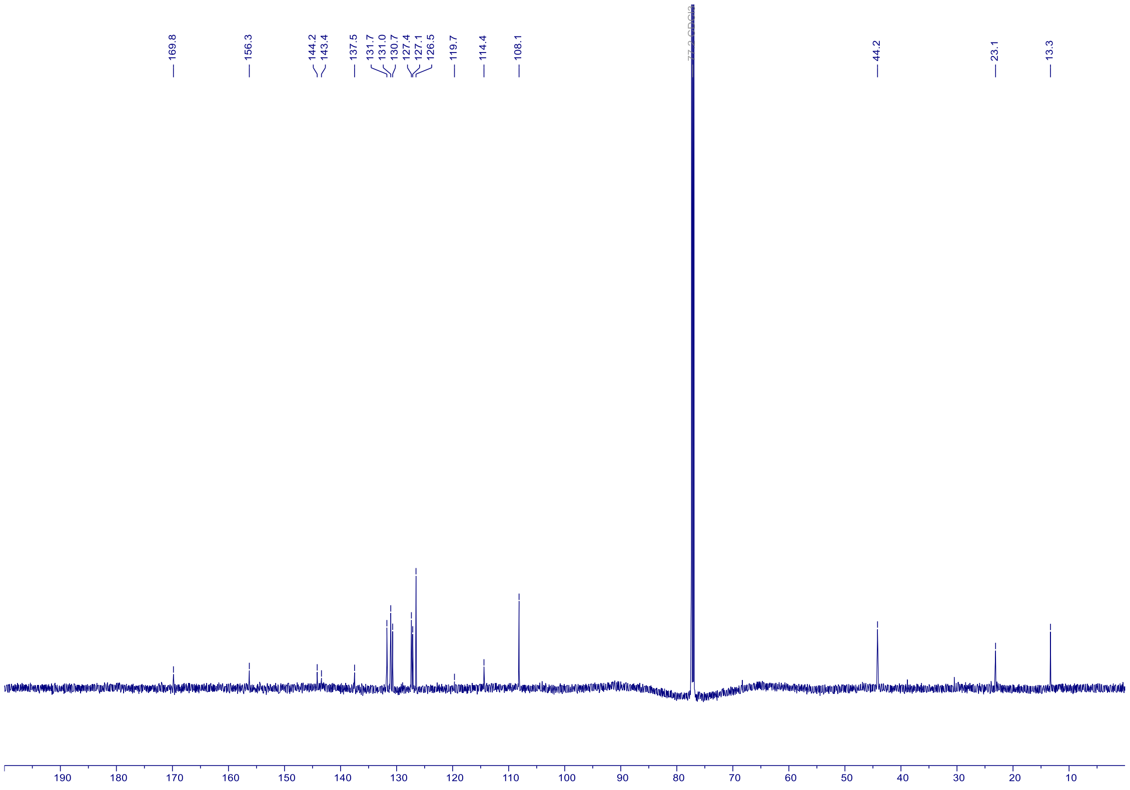

6-(2-Hydroxy-2-methylpropoxy)-8-(6-(6-((6-methoxypyridin-3-yl)methyl)-3,6-diazabicyclo[3.1.1]heptan-3-yl)pyridin-3-yl)imidazo[1,5-*a*]pyridine-1-carbonitrile (83b)

^{1}H NMR (600 MHz, $CDCl_3$)

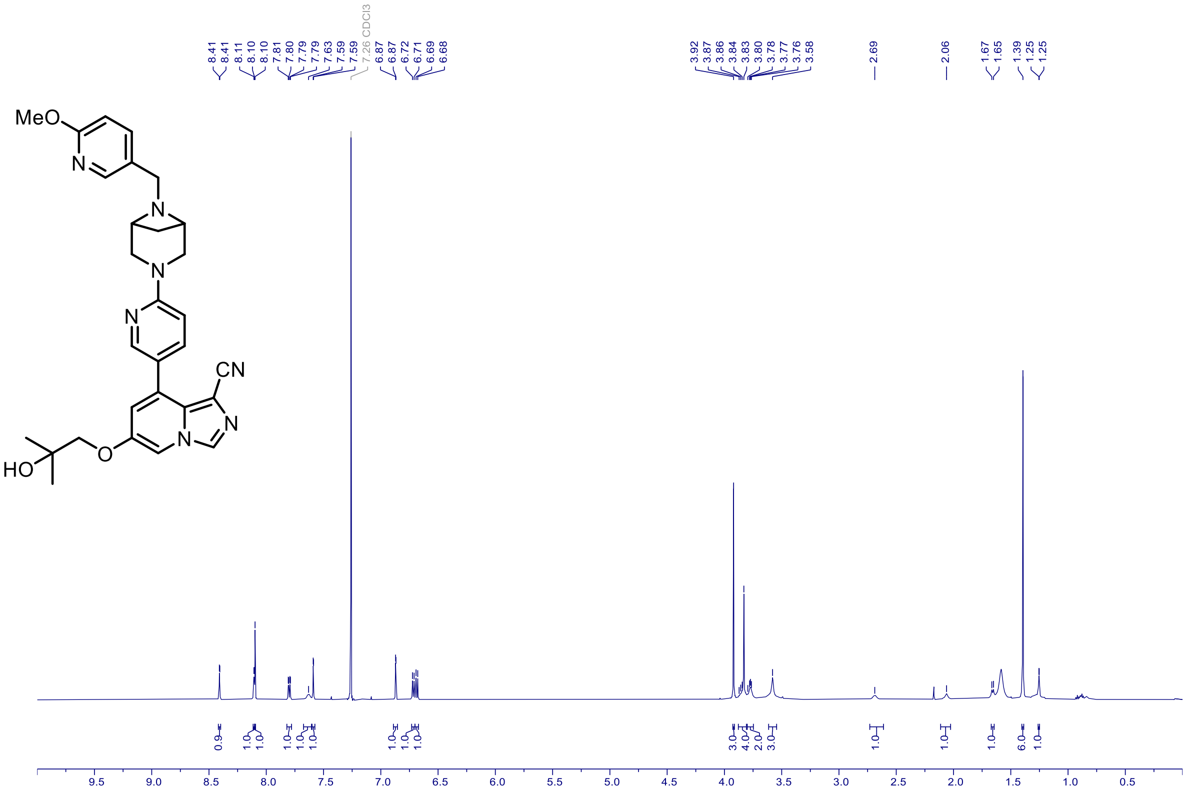

^{13}C NMR (151 MHz, $CDCl_3$)

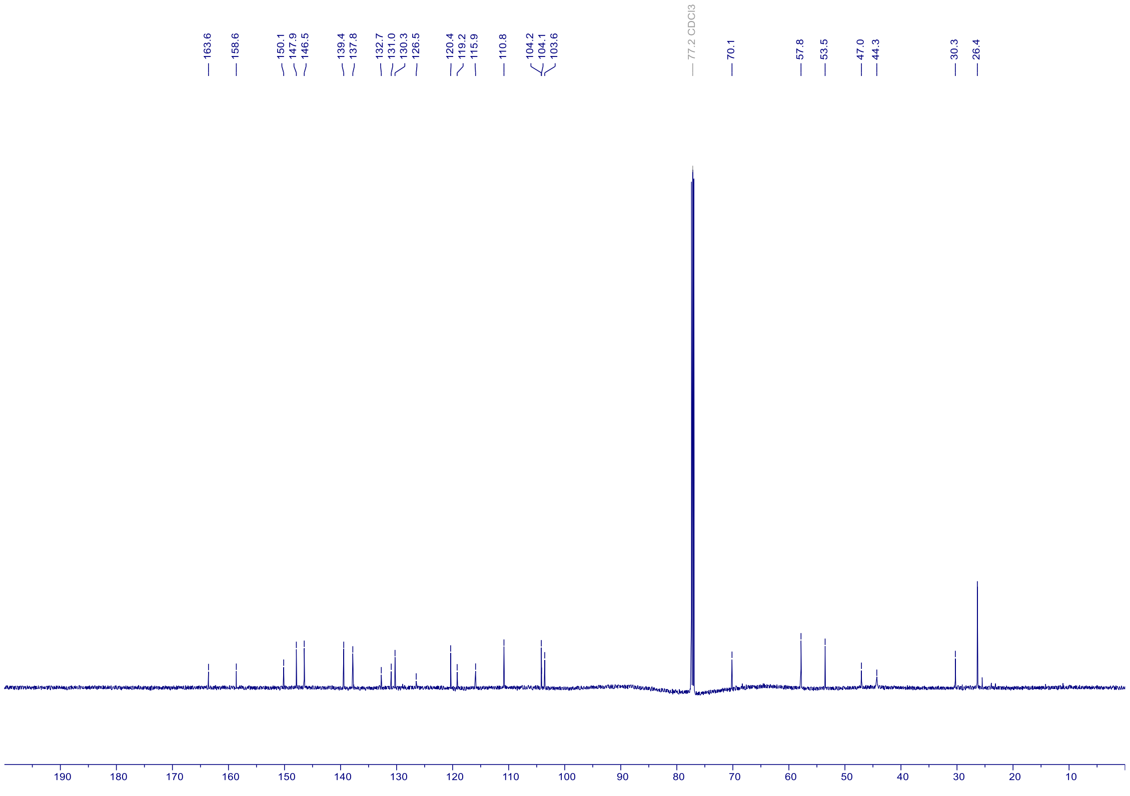

13 References

1. Liu, P.M., and Frost, C.G. (2013). Ruthenium-Catalyzed C–H Functionalization of Arylpyrazoles: Regioselective Acylation with Acid Chlorides. Org. Lett. *15*, 5862–5865. https://doi.org/10.1021/ol402936c.

2. Chevallier, F., Halauko, Y.S., Pecceu, C., Nassar, I.F., Dam, T.U., Roisnel, T., Matulis, V.E., Ivashkevich, O.A., and Mongin, F. (2011). N-aryl pyrazoles: DFT calculations of CH acidity and deprotonative metallation using a combination of lithium and zinc amides. Org. Biomol. Chem. *9*, 4671–4684. https://doi.org/10.1039/C1OB05267E.

3. Yuan, C., Zhao, Y., and Zheng, L. (2019). α-d-Galacturonic Acid as Natural Ligand for Selective Copper-Catalyzed N-Arylation of N-Containing Heterocycles. Synlett *30*, 2173–2180. https://doi.org/10.1055/s-0039-1690226.

4. Liu, Z.-J., Vors, J.-P., Gesing, E.R.F., and Bolm, C. (2011). Microwave-assisted solvent- and ligand-free copper-catalysed cross-coupling between halopyridines and nitrogen nucleophiles. Green Chem. *13*, 42–45. https://doi.org/10.1039/C0GC00296H.

5. Zhang, J., Jia, R.-P., and Wang, D.-H. (2016). Copper-catalyzed C–N cross-coupling of arylboronic acids with *N*-acylpyrazoles. Tetrahedron Lett. *57*, 3604–3607. https://doi.org/10.1016/j.tetlet.2016.06.044.

6. Pavlik, J.W., Connors, R.E., Burns, D.S., and Kurzweil, E.M. (1993). Phototransposition chemistry of 1-phenylpyrazole. Experimental and computational studies. J. Am. Chem. Soc. *115*, 7645–7652. https://doi.org/10.1021/ja00070a008.

7. Molteni, G., Ponti, A., and Orlandi, M. (2002). Uncommon aqueous media for nitrilimine cycloadditions. I. Synthetic and mechanistic aspects in the formation of 1-aryl-5-substituted-4,5-dihydropyrazoles. New J. Chem. *26*, 1340–1345. https://doi.org/10.1039/B205063C.

8. Hou, Z.-W., Yan, H., Song, J., and Xu, H.-C. (2023). Photoelectrocatalytic C–H amination of arenes. Green Chem. *25*, 7959–7962. https://doi.org/10.1039/D3GC02126B.

9. Kazlauskas, K., Kreiza, G., Arbačiauskienė, E., Bieliauskas, A., Getautis, V., Šačkus, A., and Juršėnas, S. (2014). Morphology and Emission Tuning in Fluorescent Nanoparticles Based on Phenylenediacetonitrile. J. Phys. Chem. C *118*, 25261–25271. https://doi.org/10.1021/jp507707f.

10. Clapham, K.M., Batsanov, A.S., Bryce, M.R., and Tarbit, B. (2009). Trifluoromethyl-substituted pyridyl- and pyrazolylboronic acids and esters: synthesis and Suzuki–Miyaura cross-coupling reactions. Org. Biomol. Chem. *7*, 2155–2161. https://doi.org/10.1039/B901024F.

11. Lu, J., Man, Y., Zhang, Y., Lin, B., Lin, Q., and Weng, Z. (2019). Copper-catalyzed chemoselective synthesis of 4-trifluoromethyl pyrazoles. RSC Adv. *9*, 30952–30956. https://doi.org/10.1039/C9RA07694H.

12. Xia, T., Hu, Z., Ji, W., Zhang, S., Shi, H., Liu, C., Pang, B., Liu, G., and Liao, X. (2018). Synthesis of withasomnine and pyrazole derivatives via intramolecular dehydrogenative cyclization, as well as

biological evaluation of withasomnine-based scaffolds. Org. Chem. Front. *5*, 850–854. https://doi.org/10.1039/C7QO00847C.

13. Zhang, X., Kang, J., Niu, P., Wu, J., Yu, W., and Chang, J. (2014). I2-Mediated Oxidative C–N Bond Formation for Metal-Free One-Pot Synthesis of Di-, Tri-, and Tetrasubstituted Pyrazoles from α,β-Unsaturated Aldehydes/Ketones and Hydrazines. J. Org. Chem. *79*, 10170–10178. https://doi.org/10.1021/jo501844x.

14. Oberli, M.A., and Buchwald, S.L. (2012). A General Method for Suzuki–Miyaura Coupling Reactions Using Lithium Triisopropyl Borates. Org. Lett. *14*, 4606–4609. https://doi.org/10.1021/ol302063g.

15. Font-Sanchis, E., Céspedes-Guirao, F.J., Sastre-Santos, Á., and Fernández-Lázaro, F. (2007). Indium-Mediated Synthesis of Heterobiaryls. J. Org. Chem. *72*, 3589–3591. https://doi.org/10.1021/jo062638k.

16. Sarkar, K., Kumar, P., Mule, A., and Maji, B. (2024). Divergent Synthesis of Pyrazoles via Manganese Pincer Complex Catalyzed Acceptorless Dehydrogenative Coupling Reactions. Chem. – Eur. J. *30*, e202401105. https://doi.org/10.1002/chem.202401105.

17. West, M.J., and Watson, A.J.B. (2019). Ni vs. Pd in Suzuki–Miyaura sp2–sp2 cross-coupling: a head-to-head study in a comparable precatalyst/ligand system. Org. Biomol. Chem. *17*, 5055–5059. https://doi.org/10.1039/C9OB00561G.

18. Sen, P.P., and Roy, S.R. (2023). Introducing Phenalenyl-Based Organic Lewis Acid as a Photocatalyst to Facilitate Oxidative Azolation of Unactivated Arenes. Org. Lett. *25*, 1895–1900. https://doi.org/10.1021/acs.orglett.3c00409.

19. Xu, Z.-L., Li, H.-X., Ren, Z.-G., Du, W.-Y., Xu, W.-C., and Lang, J.-P. (2011). Cu(OAc)2·H2O-catalyzed N-arylation of nitrogen-containing heterocycles. Tetrahedron *67*, 5282–5288. https://doi.org/10.1016/j.tet.2011.05.025.

20. Majumder, S., Gipson, K.R., Staples, R.J., and Odom, A.L. (2009). Pyrazole Synthesis Using a Titanium-Catalyzed Multicomponent Coupling Reaction and Synthesis of Withasomnine. Adv. Synth. Catal. *351*, 2013–2023. https://doi.org/10.1002/adsc.200900293.

21. Guo, H., Zhang, D., Zhu, C., Li, J., Xu, G., and Sun, J. (2014). When Aryldiazonium Salts Meet Vinyl Diazoacetates: A Cobalt-Catalyzed Regiospecific Synthesis of N-Arylpyrazoles. Org. Lett. *16*, 3110–3113. https://doi.org/10.1021/ol5012339.

22. Surmont, R., Verniest, G., and De Kimpe, N. (2010). New Synthesis of Fluorinated Pyrazoles. Org. Lett. *12*, 4648–4651. https://doi.org/10.1021/ol1019713.

23. Chan, K.Shing., and Wulff, W.D. (1986). 1,3-Dipolar cycloaddition reactions of transition-metal carbene complexes and the formal [3 + 2 + 1] pyridinannulation of the cycloadducts. J. Am. Chem. Soc. *108*, 5229–5236. https://doi.org/10.1021/ja00277a029.

24. Konwar, M., Phukan, P., Chaliha, A.K., Buragohain, A.K., Damarla, K., Gogoi, D., Kumar, A., and Sarma, D. (2019). An Unexplored Lewis Acidic Catalytic System for Synthesis of Pyrazole and its Biaryls

Derivatives with Antimicrobial Activities through Cycloaddition-Iodination-Suzuki Reaction. ChemistrySelect *4*, 10236–10245. https://doi.org/10.1002/slct.201902266.

25. Lakeland, C.P., Watson, D.W., and Harrity, J.P.A. (2020). Exploiting Synergistic Catalysis for an Ambient Temperature Photocycloaddition to Pyrazoles. Chem. – Eur. J. *26*, 155–159. https://doi.org/10.1002/chem.201904210.

26. Cho, C.S., and Patel, D.B. (2006). Palladium-catalyzed C–N bond formation: synthesis of 1-aryl-1*H*-pyrazoles from β-bromovinyl aldehydes and arylhydrazines. Tetrahedron *62*, 6388–6391. https://doi.org/10.1016/j.tet.2006.04.031.

27. Mennie, K.M., Reutershan, M.H., White, C., Adams, B., Becker, B., Deng, J., Katz, J.D., LaBlue, E., Margrey, K., and Saurí, J. (2021). Divergent and Regioselective Synthesis of Pyrazolo[1,5-a]pyridines and Imidazo[1,5-a]pyridines. Org. Lett. *23*, 4694–4698. https://doi.org/10.1021/acs.orglett.1c01431.

28. Baykal, A., and Plietker, B. (2020). A Bu4N[Fe(CO)3(NO)]-Catalyzed Hemetsberger–Knittel Indole Synthesis. Eur. J. Org. Chem. *2020*, 1145–1147. https://doi.org/10.1002/ejoc.201901864.

29. Tear, W.F., Bag, S., Diaz-Gonzalez, R., Ceballos-Pérez, G., Rojas-Barros, D.I., Cordon-Obras, C., Pérez-Moreno, G., García-Hernández, R., Martinez-Martinez, M.S., Ruiz-Perez, L.M., et al. (2020). Selectivity and Physicochemical Optimization of Repurposed Pyrazolo[1,5-b]pyridazines for the Treatment of Human African Trypanosomiasis. J. Med. Chem. *63*, 756–783. https://doi.org/10.1021/acs.jmedchem.9b01741.

30. Rádl, S., Blahovcová, M., Tkadlecová, M., and Havlíček, J. (2010). Synthetic Studies Connected with the Preparation of N-[3-(3-Cyanopyrazolo[1,5-a]pyrimidin-5-yl)phenyl]-N-ethylacetamide, a Zaleplon Regioisomer. HETEROCYCLES *80*, 1359. https://doi.org/10.3987/COM-09-S(S)129.

31. Gair, J.J., Grey, R.L., Giroux, S., and Brodney, M.A. (2019). Palladium Catalyzed Hydrodefluorination of Fluoro-(hetero)arenes. Org. Lett. *21*, 2482–2487. https://doi.org/10.1021/acs.orglett.9b00889.

32. Ferlin, F., Trombettoni, V., Luciani, L., Fusi, S., Piermatti, O., Santoro, S., and Vaccaro, L. (2018). A waste-minimized protocol for copper-catalyzed Ullmann-type reaction in a biomass derived furfuryl alcohol/water azeotrope. Green Chem. *20*, 1634–1639. https://doi.org/10.1039/C8GC00287H.

33. Niknam, E., Panahi, F., and Khalafi-Nezhad, A. (2020). Immobilized Pd on a NHC functionalized metal–organic framework MIL-101(Cr): an efficient heterogeneous catalyst in Suzuki−Miyaura coupling reaction in water. Appl. Organomet. Chem. *34*, e5470. https://doi.org/10.1002/aoc.5470.

34. Abou-Shehada, S., Teasdale, M.C., Bull, S.D., Wade, C.E., and Williams, J.M.J. (2015). Lewis Acid Activation of Pyridines for Nucleophilic Aromatic Substitution and Conjugate Addition. ChemSusChem *8*, 1083–1087. https://doi.org/10.1002/cssc.201403154.

35. Hai, X., Zheng, Y., Yu, Q., Guo, N., Xi, S., Zhao, X., Mitchell, S., Luo, X., Tulus, V., Wang, M., et al. (2023). Geminal-atom catalysis for cross-coupling. Nature *622*, 754–760. https://doi.org/10.1038/s41586-023-06529-z.

36.Zhu, X., Su, L., Huang, L., Chen, G., Wang, J., Song, H., and Wan, Y. (2009). A Facile and Efficient Oxalyldihydrazide/Ketone-Promoted Copper-Catalyzed Amination of Aryl Halides in Water. Eur. J. Org. Chem. *2009*, 635–642. https://doi.org/10.1002/ejoc.200800940.

37.Ueda, S., Su, M., and Buchwald, S.L. (2012). Completely N1-Selective Palladium-Catalyzed Arylation of Unsymmetric Imidazoles: Application to the Synthesis of Nilotinib. J. Am. Chem. Soc. *134*, 700–706. https://doi.org/10.1021/ja2102373.

38.Zhang, C.-P., Wang, Z.-L., Chen, Q.-Y., Zhang, C.-T., Gu, Y.-C., and Xiao, J.-C. (2011). Copper-Mediated Trifluoromethylation of Heteroaromatic Compounds by Trifluoromethyl Sulfonium Salts. Angew. Chem. Int. Ed. *50*, 1896–1900. https://doi.org/10.1002/anie.201006823.

39.Bellina, F., Cauteruccio, S., Mannina, L., Rossi, R., and Viel, S. (2005). Regioselective Synthesis of 1,5-Diaryl-1H-imidazoles by Palladium-Catalyzed Direct Arylation of 1-Aryl-1H-imidazoles. J. Org. Chem. *70*, 3997–4005. https://doi.org/10.1021/jo050274a.

40.Duong, H.A., Wu, W., and Teo, Y.-Y. (2017). Cobalt-Catalyzed Cross-Coupling Reactions of Arylboronic Esters and Aryl Halides. Organometallics *36*, 4363–4366. https://doi.org/10.1021/acs.organomet.7b00726.

41.Bhatt, R., Bhuvanesh, N., Sharma, K.N., and Joshi, H. (2020). Palladium Complexes of Thio/Seleno-Ether Containing N-Heterocyclic Carbenes: Efficient and Reusable Catalyst for Regioselective C-H Bond Arylation. Eur. J. Inorg. Chem. *2020*, 532–540. https://doi.org/10.1002/ejic.201901259.

42.Laroche, C., Li, J., Freyer, M.W., and Kerwin, S.M. (2008). Coupling Reactions of Bromoalkynes with Imidazoles Mediated by Copper Salts: Synthesis of Novel N-Alkynylimidazoles. J. Org. Chem. *73*, 6462–6465. https://doi.org/10.1021/jo801118q.

43.Joo, J.M., Touré, B.B., and Sames, D. (2010). C−H Bonds as Ubiquitous Functionality: A General Approach to Complex Arylated Imidazoles via Regioselective Sequential Arylation of All Three C−H Bonds and Regioselective N-Alkylation Enabled by SEM-Group Transposition. J. Org. Chem. *75*, 4911–4920. https://doi.org/10.1021/jo100727j.

44.Du, Y., Wang, Y., Li, X., Shao, Y., Li, G., Webster, R.D., and Chi, Y.R. (2014). N-Heterocyclic Carbene Organocatalytic Reductive β,β-Coupling Reactions of Nitroalkenes via Radical Intermediates. Org. Lett. *16*, 5678–5681. https://doi.org/10.1021/ol5027415.

45.O’Connell, J.F., Parquette, J., Yelle, W.E., Wang, W., and Rapoport, H. (1988). Convenient Synthesis of Methyl 1-Methyl-2,4-dibromo-5-imidazolecarboxylate. Synthesis *1988*, 767–771. https://doi.org/10.1055/s-1988-27702.

46.Das, A. (2017). Investigation into Sonogashira reaction on 5-iodo-1-(phenyl/p-halophenyl)imidazole-4-carbonitrile compounds. Synth. Commun. *47*, 2254–2267. https://doi.org/10.1080/00397911.2017.1373298.

47.Tang, D., Wu, P., Liu, X., Chen, Y.-X., Guo, S.-B., Chen, W.-L., Li, J.-G., and Chen, B.-H. (2013). Synthesis of Multisubstituted Imidazoles via Copper-Catalyzed [3 + 2] Cycloadditions. J. Org. Chem. *78*, 2746–2750. https://doi.org/10.1021/jo302555z.

48.Chen, C.-Y., Hu, W.-P., Yan, P.-C., Senadi, G.C., and Wang, J.-J. (2013). Metal-Free, Acid-Promoted Synthesis of Imidazole Derivatives via a Multicomponent Reaction. Org. Lett. *15*, 6116–6119. https://doi.org/10.1021/ol402892z.

49.Pardeshi, S.D., Sathe, P.A., Vadagaonkar, K.S., and Chaskar, A.C. (2017). One-Pot Protocol for the Synthesis of Imidazoles and Quinoxalines using N-Bromosuccinimide. Adv. Synth. Catal. *359*, 4217–4226. https://doi.org/10.1002/adsc.201700900.

50.Pews-Davtyan, A., and Beller, M. (2011). A novel Zn-catalyzed hydroamination of propargylamides: a general synthesis of di- and tri-substituted imidazoles. Chem. Commun. *47*, 2152–2154. https://doi.org/10.1039/C0CC04625F.

51.Gómez-Sánchez, A., Hidalgo, F.J., and Chiara, J.L. (1987). Studies on nitroenamines. Part III. Synthesis and spectral properties of 4-Acyl-1-arylimidazoles. J. Heterocycl. Chem. *24*, 1757–1763. https://doi.org/10.1002/jhet.5570240649.

52.Das, A. (2017). Studies on complex π-π and T-stacking features of imidazole and phenyl/*p*-halophenyl units in series of 5-amino-1-(phenyl/*p*-halophenyl)imidazole-4-carboxamides and their carbonitrile derivatives: Role of halogens in tuning of conformation. J. Mol. Struct. *1147*, 520–540. https://doi.org/10.1016/j.molstruc.2017.06.124.

53.Konishi, H., Ueda, T., Muto, T., and Manabe, K. (2012). Remarkable Improvement Achieved by Imidazole Derivatives in Ruthenium-Catalyzed Hydroesterification of Alkenes Using Formates. Org. Lett. *14*, 4722–4725. https://doi.org/10.1021/ol301850y.

54.太田元告, 越克己, and 小畑和永 (1968). Investigations on Steroids. XI. Synthesis of Steroidal Oxazole, Imidazole, and Triazole. Chem. Pharm. Bull. (Tokyo) *16*, 1487–1497. https://doi.org/10.1248/cpb.16.1487.

55.Xiao, Y., and Zhang, L. (2012). Synthesis of Bicyclic Imidazoles via [2 + 3] Cycloaddition between Nitriles and Regioselectively Generated α-Imino Gold Carbene Intermediates. Org. Lett. *14*, 4662–4665. https://doi.org/10.1021/ol302102h.

56.Tran, R.Q., Dinh, L.P., Jacoby, S.A., Harris, N.W., Swann, W.A., Williamson, S.N., Semsey, R.Y., and Yet, L. (2021). Synthesis of 3-aryl-1-phosphinoimidazo[1,5-a]pyridine ligands for use in Suzuki–Miyaura cross-coupling reactions. RSC Adv. *11*, 28347–28351. https://doi.org/10.1039/D1RA05417A.

57.Li, M., Xie, Y., Ye, Y., Zou, Y., Jiang, H., and Zeng, W. (2014). Cu(I)-Catalyzed Transannulation of N-Heteroaryl Aldehydes or Ketones with Alkylamines via C(sp3)–H Amination. Org. Lett. *16*, 6232–6235. https://doi.org/10.1021/ol503165b.

58.Tolkunov, V.S., Tolkunov, A.S., Smirnova, O.V., and Tolkunov, S.V. (2021). Convenient synthesis of imidazo[1,5-a]pyrimidine derivatives and their unusual recyclization into 3H-imidazo[4,5-b]pyridine derivatives. Chem. Heterocycl. Compd. *57*, 554–559. https://doi.org/10.1007/s10593-021-02942-2.

59.Anglada, L., Palomer, A., and Guglietta, A. (2006). N-(3-(imidazo [1,5-a]pyrimidin-4-yl)phenyl]-sulfonamides and n-[3-(imidazo[1,5-a]pyrimidin-4-yl)-phenyl]-carboxamides and their use as gabaa

receptor modulators.

60. Alves, M.J., Fortes, A.G., Lemos, A., and Martins, C. (2005). Ethyl 3-(2-Pyridyl)-2H-azirine-2-carboxylate: Synthesis and Reaction with Dienes. Synthesis *2005*, 555–558. https://doi.org/10.1055/s-2004-837296.

61. Seto, S., Nishigaya, Y., Tanioka, A., Tatani, K., and Kondo, A. (2014). Pyrazolopyridine Derivative or Pharmacologically Acceptable Salt Thereof.

62. Benincori, T., Brenna, E., and Sannicolo, F. (1993). Studies on Wallach's imidazole synthesis. J. Chem. Soc. Perkin 1, 675–679. https://doi.org/10.1039/P19930000675.

63. Roseau, M., Chausset-Boissarie, L., Gremetz, S., Roth, P.M.C., and Penhoat, M. (2022). Multiple wavelength (365–475 nm) complete actinometric characterization of Corning® Lab Photo Reactor using azobenzene as a highly soluble, cheap and robust chemical actinometer. Photochem. Photobiol. Sci. *21*, 421–432. https://doi.org/10.1007/s43630-022-00171-w.

64. Balzani, V., Ceroni, P., and Juris, A. (2014). Photochemistry and Photophysics: Concepts, Research, Applications (John Wiley & Sons).

65. Casida, M.E., Jamorski, C., Casida, K.C., and Salahub, D.R. (1998). Molecular excitation energies to high-lying bound states from time-dependent density-functional response theory: Characterization and correction of the time-dependent local density approximation ionization threshold. J. Chem. Phys. *108*, 4439–4449. https://doi.org/10.1063/1.475855.

66. Yanai, T., Tew, D.P., and Handy, N.C. (2004). A new hybrid exchange–correlation functional using the Coulomb-attenuating method (CAM-B3LYP). Chem. Phys. Lett. *393*, 51–57. https://doi.org/10.1016/j.cplett.2004.06.011.

67. Dunning, T.H., Jr. (1989). Gaussian basis sets for use in correlated molecular calculations. I. The atoms boron through neon and hydrogen. J. Chem. Phys. *90*, 1007–1023. https://doi.org/10.1063/1.456153.

68. Kendall, R.A., Dunning, T.H., Jr., and Harrison, R.J. (1992). Electron affinities of the first-row atoms revisited. Systematic basis sets and wave functions. J. Chem. Phys. *96*, 6796–6806. https://doi.org/10.1063/1.462569.

69. Woon, D.E., and Dunning, T.H., Jr. (1993). Gaussian basis sets for use in correlated molecular calculations. III. The atoms aluminum through argon. J. Chem. Phys. *98*, 1358–1371. https://doi.org/10.1063/1.464303.

70. Peterson, K.A., Woon, D.E., and Dunning, T.H., Jr. (1994). Benchmark calculations with correlated molecular wave functions. IV. The classical barrier height of the H+H2→H2+H reaction. J. Chem. Phys. *100*, 7410–7415. https://doi.org/10.1063/1.466884.

71. Wilson, A.K., van Mourik, T., and Dunning, T.H. (1996). Gaussian basis sets for use in correlated molecular calculations. VI. Sextuple zeta correlation consistent basis sets for boron through neon. J. Mol. Struct. THEOCHEM *388*, 339–349. https://doi.org/10.1016/S0166-1280(96)80048-0.

72.Neese, F. (2012). The ORCA program system. WIREs Comput. Mol. Sci. *2*, 73–78. https://doi.org/10.1002/wcms.81.

73.Neese, F. (2018). Software update: the ORCA program system, version 4.0. WIREs Comput. Mol. Sci. *8*, e1327. https://doi.org/10.1002/wcms.1327.

74.Neese, F., Wennmohs, F., Becker, U., and Riplinger, C. (2020). The ORCA quantum chemistry program package. J. Chem. Phys. *152*, 224108. https://doi.org/10.1063/5.0004608.

75.Hohenberg, P., and Kohn, W. (1964). Inhomogeneous Electron Gas. Phys. Rev. *136*, B864–B871. https://doi.org/10.1103/PhysRev.136.B864.

76.Kohn, W., and Sham, L.J. (1965). Self-Consistent Equations Including Exchange and Correlation Effects. Phys. Rev. *140*, A1133–A1138. https://doi.org/10.1103/PhysRev.140.A1133.

77.Parr, R.G., and Yang, W. (1989). Density-Functional Theory of Atoms and Molecules (Oxford University Press).

78.D. R. Salahub and M. C. Zerner (1989). The Challenge of d and f Electrons. In ACS Symposium Series., M. Joan Comstock, ed. (American Chemical Society). https://doi.org/10.1021/bk-1989-0394.fw001.

79.M. J. Frisch, G. W. Trucks, H. B. Schlegel, G. E. Scuseria, M. A. Robb, J. R. Cheeseman, G. Scalmani, V. Barone, G. A. Petersson, H. Nakatsuji, X. Li, M. Caricato, A. V. Marenich, J. Bloino, B. G. Janesko, R. Gomperts, B. Mennucci, H. P. Hratchian, J. V. OrΘz, A. F. Izmaylov, J. L. Sonnenberg, Williams, F. Ding, F. Lipparini, F. Egidi, J. Goings, B. Peng, A. Petrone, T. Henderson, D. Ranasinghe, V. G. Zakrzewski, J. Gao, N. Rega, G. Zheng, W. Liang, M. Hada, M. Ehara, K. Toyota, R. Fukuda, J. Hasegawa, M. Ishida, T. Nakajima, Y. Honda, O. Kitao, H. Nakai, T. Vreven, K. Throssell, J. A. Montgomery Jr., J. E. Peralta, F. Ogliaro, M. J. Bearpark, J. J. Heyd, E. N. Brothers, K. N. Kudin, V. N. Staroverov, T. A. Keith, R. Kobayashi, J. Normand, K. Raghavachari, A. P. Rendell, J. C. Burant, S. S. Iyengar, J. Tomasi, M. Cossi, J. M. Millam, M. Klene, C. Adamo, R. Cammi, J. W. Ochterski, R. L. MarΘn, K. Morokuma, O. Farkas, J. B. Foresman, D. J. Fox, Wallingford, CT, 2016.

80.Chai, J.-D., and Head-Gordon, M. (2008). Long-range corrected hybrid density functionals with damped atom–atom dispersion corrections. Phys. Chem. Chem. Phys. *10*, 6615–6620. https://doi.org/10.1039/B810189B.

81.Weigend, F., and Ahlrichs, R. (2005). Balanced basis sets of split valence, triple zeta valence and quadruple zeta valence quality for H to Rn: Design and assessment of accuracy. Phys. Chem. Chem. Phys. *7*, 3297–3305. https://doi.org/10.1039/B508541A.

82.Marenich, A.V., Cramer, C.J., and Truhlar, D.G. (2009). Universal Solvation Model Based on Solute Electron Density and on a Continuum Model of the Solvent Defined by the Bulk Dielectric Constant and Atomic Surface Tensions. J. Phys. Chem. B *113*, 6378–6396. https://doi.org/10.1021/jp810292n.

83.Simlandy, A.K., Rodphon, W., Alturaifi, T.M., Mai, B.K., Ni, H.-Q., Gurak, J.A.Jr., Liu, P., and Engle, K.M. (2022). Catalytic Addition of Nitroalkanes to Unactivated Alkenes via Directed Carbopalladation. ACS Catal. *12*, 13755–13762. https://doi.org/10.1021/acscatal.2c04557.

84. Grimme, S. (2012). Supramolecular Binding Thermodynamics by Dispersion-Corrected Density Functional Theory. Chem. – Eur. J. *18*, 9955–9964. https://doi.org/10.1002/chem.201200497.

85. Luchini, G., Alegre-Requena, J.V., Funes-Ardoiz, I., and Paton, R.S. (2020). GoodVibes: automated thermochemistry for heterogeneous computational chemistry data. Preprint at F1000Research, https://doi.org/10.12688/f1000research.22758.1 https://doi.org/10.12688/f1000research.22758.1.

86. Wigner, E. (1932). Über das Überschreiten von Potentialschwellen bei chemischen Reaktionen. Z. Für Phys. Chem. *19B*, 203–216. https://doi.org/10.1515/zpch-1932-1920.

87. Skodje, R.T., and Truhlar, D.G. (1981). Parabolic tunneling calculations. J. Phys. Chem. *85*, 624–628. https://doi.org/10.1021/j150606a003.

88. Eckart, C. (1930). The Penetration of a Potential Barrier by Electrons. Phys. Rev. *35*, 1303–1309. https://doi.org/10.1103/PhysRev.35.1303.

89. Garrett, B.C., and Truhlar, D.G. (1979). Semiclassical tunneling calculations. J. Phys. Chem. *83*, 2921–2926. https://doi.org/10.1021/j100485a023.

90. Ang, S.J. (2024). SJ-Ang/PyTUN.
